# Unconventional semi-solid cultivation enhances cytochalasins production by the Colombian fungus *Xylaria* sp. CM-UDEA-H199

**DOI:** 10.1186/s12896-025-00978-2

**Published:** 2025-07-01

**Authors:** Daniela Valencia-Revelo, Esteban Charria-Girón, Katharina Schmidt, Silke Reinecke, Aida M. Vasco-Palacios, Theresia Stradal, Yasmina Marin-Felix, Nelson H. Caicedo-Ortega, Sherif S. Ebada

**Affiliations:** 1 Department Microbial Drugs, Helmholtz Centre for Infection Research (HZI), Inhoffenstrasse 7, 38124 Braunschweig, Germany; 2 Departamento de Ciencias Biológicas, Bioprocesos y Biotecnología. Facultad de Ingeniería, Diseño y Ciencias Aplicadas, Universidad Icesi, Calle 18 No. 122-135, Cali, Colombia; 3 Institute of Microbiology, Technische Universität Braunschweig, Spielmannstrasse 7, 38106 Braunschweig, Germany; 4 Department of Cell Biology, Helmholtz Centre for Infection Research (HZI), Inhoffenstrasse 7, 38124 Braunschweig, Germany; 5 Grupo de Microbiología Ambiental y Grupo BioMicro, Escuela de Microbiología, Universidad de Antioquia UdeA, Calle 67 No. 53-108, Medellín, Colombia; 6 Centro BioInc, Universidad Icesi, Calle 18 No. 122-135, Cali, Colombia; 7 Department of Pharmacognosy, Faculty of Pharmacy, Ain Shams University, Organization of African Unity Street 1, Cairo, 11566 Egypt

**Keywords:** Actin disruption, Cytochalasins, Metabolomics, Nutrient limitation, Xylariales

## Abstract

Fungal species of the order Xylariales, particularly those from tropical and untapped areas like the Amazon region, denote an intriguing reservoir of biodiversity and chemically varied metabolites. Based on this potential and by implementing the One Strain Many Compounds (OSMAC) approach, herein we have cultivated a Colombian *Xylaria* strain in several liquid, solid or semi-solid media, under different nutrient compositions and culture conditions. Metabolomic studies of *Xylaria* sp. CM-UDEA-H199 across these conditions led to the isolation of diverse metabolites. Six compounds were purified from rice (BRFT) cultures, identified as griseofulvin (**1**), xylaropyrones B/C (**2/3**), akolitserin (**4**), hypoxylin A (**5**), and (-)-(*R*)-5-(methoxycarbonyl)mellein (**6**). Three compounds were isolated from liquid YM cultivation: 2-hexylidene-3-methylsuccinic acid (**7**), its 4-methyl ester (**8**), and akoenic acid (**9**). Notably, cultivation in the newly designed semi-solid (S-BRFT) medium significantly altered the metabolome, leading to the predominant production of cytochalasins, with five derivatives (**10**–**14**) purified and structurally characterized.

Among the isolated cytochalasins, compound **12** was identified as a previously undescribed natural diepoxy derivative of cytochalasin D. Structure elucidation of all isolated compounds was achieved based on their MS and comprehensive 1D/2D NMR analyses in addition to comparisons with the reported literature. Compounds **4**–**6**, **10** and **11** revealed mild antifungal activity, while compounds (**1, 5, 6, 8, 10, 11, 13** and **14**) exhibited cytotoxic activity, with hypoxylin A (**5**) being the most potent, displaying IC_50_ values in the nanomolar range. *In cellulo* studies revealed that the epimerization at C-5 of cytochalasin D (**10**) backbone, as in hypoxylin A (**5**), neither affected its activity nor reversibility on actin dynamics. However, the epoxylated variant of **10**, cytochalasin R (**14**), enhanced actin activity accompanied by reduced cytotoxicity compared to **5** and **10**. The occurrence of diverse epoxy-substituted cytochalasins suggests that specific biosynthetic enzymes were activated in response to the applied fermentation conditions. These findings provide a basis for further bioprocess optimization strategies aimed at enhancing cytochalasan production, a chemical class recognized for its promising bioactivities in recent decades.

**Supplementary Information:**

The online version contains supplementary material available at 10.1186/s12896-025-00978-2.

## Introduction

Filamentous fungi belonging to the Ascomycota are characterized by their ability to produce diverse and structurally complex secondary metabolites (SMs), with some of them displaying a broad spectrum of biological activities [1]. These molecules have been an important source for the development of therapeutic agents, including antimicrobials, antivirals, anticancers, immunosuppressants, and immunomodulators, among others [2]. For instance, the genera belonging to the order Xylariales represent one of the largest groups of secondary metabolites producers [3]. Important pharmaceutical agents such as the antiparasitic nodulisporic acids and the anthelmintic PF-1022 derivatives (aka emodepside) have been isolated from these taxa [4]. Other interesting chemical classes such as the cytochalasans are widely produced by its members [3]. These molecules, identified as products of hybrid polyketide synthase non-ribosomal peptide synthetases (PKS-NRPS), are of general interest due to their various biological properties [5]; particularly, for their capacity to block actin polymerization in eukaryotic cells [6, 7]. Nevertheless, novel carbon skeletons from different biosynthetic pathways continue to be discovered from members of this order as revealed by natural product screening programs [3].

The production of SMs and their chemical diversity is generally dependent on the expression of the so-called biosynthetic gene clusters (BGCs), which represent genes located in high proximity that are often co-regulated and responsible for the biosynthesis of a specific group of molecules [8]. Fungal BGCs might be activated in response to biotic and/or abiotic factors in their environment such as: pH, temperature, light, and nutrient availability [9]; as well as for the interactions held with other organisms, all enclosed by the complex regulatory network governing this process [10]. Despite the high number of BGCs found in different fungal species, relatively few compounds have been elucidated to the date [11]. Most BGCs are silent or not expressed in a sufficient level to identify their products implying an immense spectrum of metabolites that are yet unknown under standard laboratory conditions [12]. Thus, different strategies have been designed to unlock/activate silent BGCs and hence, to induce the production of previously undescribed natural products or rare derivatives [13, 14].

Altering the cultivation conditions proved to be one of the simplest and most effective strategies to study fungal metabolic potential via the so-called One Strain Many Compounds (OSMAC) approach [15]. Commonly, screening campaigns involve cultivation under axenic conditions in different standardized media, aiming to explore the SMs diversity. Nonetheless, if analyzed in detail there are multiple factors playing significant roles beyond media composition, such as nutrient sources complexity and availability during cultivation process, the ratio in which nutrients are formulated, as well as oxygen transfer through the media. Furthermore, when key nutrients like carbon, nitrogen or phosphate reach a state of limitation during the cultivation, some species might start producing different SMs and/or their derivatives [16]. For instance, regarding the complexity of nutrient sources, the production of corymbiferones and the corymbiferan lactones by different *Penicillium* spp. was enhanced when using a plant-derived media from macerated bulb tissues instead of common laboratory media [17]. On the other hand, studies showed that the production of lovastatin, a cholesterol-lowering drug, was affected by the changes in carbon (C)/nitrogen (N) ratio during the fermentation of *Aspergillus terreus* [18]. Altering the same parameter of C/N ratio also affected the production of the mycotoxins, alternariol and tenuazonic acid, by *Alternaria alternata * [19]. Finally, the effect of oxygen supply based on the configuration of the media (solid or liquid; static or dynamic) was explored when growing *Sphaeropsidales* sp. in shaken and static liquid or in static solid media. There, six new spirobisnaphthalenes resulted from the fermentation under static conditions, where the oxygen supply was limited [20].

In addition to the high diversity and the promising potential of taxa belonging to the Xylariales, untapped areas of biodiversity like the Amazon region in Colombia further enhance the chances of finding interesting fungi [21]. In this study, a fungus collected from the Amazon region (Department of Guaviare, Colombia), initially classified in the genus *Xylaria*, was cultivated using a panel of four standardized media to evaluate its SMs production and their biological properties. During this process, the potential production of cytochalasans was observed in both liquid and solid media. Subsequently, a new semi-solid culture medium (S-BRFT) was formulated by adjusting the nutrient concentrations of the rice (BRFT) medium to promote nutrient-limited conditions and mitigate oxygen limitation, leading to enhanced production of cytochalasans.

## Materials and methods

### Fungal material and molecular identification

The fungus was collected in *La Serranía de la Lindosa* (2°28′49″N 72°44′43″O) in San José del Guaviare (Guaviare) located in the Colombian Amazon region in November 2021. The sample was isolated from the sporome, which was found on a decomposing log. A fragment of the tissue was washed for 1 min with 0.01% Tween 80, 1 min with sterile water, 1 min with 70% ethanol, and 2 min with sterile water. Then, the pieces of tissue were placed on 2% water agar (WA) and on potato dextrose agar (PDA) with 0.05% chloramphenicol and incubated at room temperature. The obtained pure cultures were transferred and preserved in PDA plates for further experiments.

The isolated strain was deposited at the Microorganism Collection of the School of Microbiology of the University of Antioquia (CM–EM-UdeA) (Medellín, Colombia) with the collection code CM-UDEA-H199.

The DNA extraction of this fungus and the amplification and sequencing of the internal transcribed spacers and intervening 5.8S nrDNA (ITS), large subunit nuclear 28S rDNA (LSU), partial second largest subunit of the DNA-directed RNA polymerase II (*rpb2*), and β-tubulin (*tub2*) genes were previously reported [22]. ITS and LSU loci were not successfully sequenced. A BLAST search based on *rpb2* and *tub2* was conducted to estimate its affinities to other described species available in GenBank. Sequences are available in the Supplementary Information (Table S8).

### Screening cultivatio

The strain was grown on Yeast Malt Agar medium (YMA) at 23 ºC. Then, five agar plugs of a well-grown culture were cut using a cork borer (7 mm diameter) and added into a 500-mL Erlenmeyer flask containing 100 mL of SMYA medium (40 g maltose, 10 g yeast extract, 10 g meat peptone, and 4 g agar per 1 L deionized water). This seed culture was incubated for six days on a rotatory shaker set at 25 ºC and 220 rpm. To investigate the production of secondary metabolites by *Xylaria* sp. CM-UDEA-H199, four different screening media were chosen: three liquid media (YM, Q6½, ZM½) and a solid-state rice medium (BRFT). The media compositions were as follows, (1) YM: (10 g malt extract, 4 g D-glucose, and 4 g yeast extract per 1 L deionized water; pH 6.3); (2) Q6½: (2.5 g D-glucose, 10 g glycerin, 5 g cotton seed flour per 1 L deionized water; pH 7.2); and (3) ZM½: (5 g molasses (Nordzucker AG), 5 g oatmeal, 4 g saccharose, 4 g mannitol, 1.5 g D-glucose, 1.5 g calcium carbonate CaCO_3_, 0.5 g edamin, 0.5 g ammonium sulphate (NH_4_)_2_SO_4_, per 1 L deionized water; pH 7.2), and BRFT (each flask with 28 g brown rice, 1 g yeast extract, 0.5 g disodium tartrate dihydrate, 0.5 g monopotassium phosphate KH_2_PO_4_ and 100 mL deionized water). To inoculate the screening liquid cultures, 2 mL of the seed culture in SMYA were added into different 500-mL Erlenmeyer flasks each containing 200 mL of the liquid medium. The pH values were set before autoclaving and all the cultures were incubated in a rotatory shaker at 23 ºC and 140 rpm in darkness. Fungal growth was monitored daily by measuring the free glucose in the media using Medi-Test glucose stripes (Macherey-Nagel, Düren, Germany). After three days of glucose depletion, the cultures were extracted. For the solid culture, also 2 mL of the seed culture were added to the medium contained in a 500-mL Erlenmeyer flask and mixed thoroughly. Then, it was incubated statically for 15 days at 23 ºC in darkness.

To enhance the production of secondary metabolites, a new semi-solid medium, denoted as a slurry and derived from solid rice medium (BRFT), was designed with the aim of limiting the nutritional source. This new medium was named S-BRFT. To get it prepared, brown rice was blended until obtaining a flour-like consistency. The medium was prepared by adding 4 g of the blended rice on each flask, 2.66 g/L yeast extract, 0.05 g disodium tartrate dihydrate, 0.05 g monopotassium phosphate in 100 mL deionized water; pH 6.3 adjusted before autoclaving, achieving a slurry-type consistency after autoclaving. To inoculate, 2 mL of seed culture in SMYA were added to a 500-mL Erlenmeyer flask containing the S-BRFT medium. The culture was incubated for 15 days on a rotatory shaker at 23ºC and 240 rpm in darkness.

### Scale-up cultivation

According to the yield of crude extract obtained in each medium and their respective secondary metabolites profile, three media were chosen for scale-up fermentations: one liquid medium, YM; the solid-state rice medium, BRFT; and the semi-solid designed medium, S-BRFT from which twenty, ten, and twenty-five 500-mL flasks were prepared, respectively. The seed culture preparation, incubation conditions and media compositions were the same as described above for the screening cultures.

### Extraction and purification of secondary metabolites

For liquid cultures, the mycelia and the supernatant were separated by filtration using a cellulose filter paper (MN 615 1/4 Ø 185 mm, Macherey-Nagel GmbH & Co. KG, Düren, Germany). The mycelia were covered with acetone (Ace) and extracted on an ultrasonic bath at 40 °C for 40 min. Then, Ace phase was separated from the mycelia by filtration and evaporated at 40 ºC using a rotary evaporator (evaporator: Heidolph Instruments GmbH & Co. KG, Germany; pump: Vacuubrand GmbH & Co. KG, Wertheim am Main, Germany) until only aqueous phase was left, while mycelia were discarded. The aqueous phase obtained was mixed with an equal volume of ethyl acetate (EtOAc) in a separatory funnel and vigorously shaken. The EtOAc phase was recovered, filtered through anhydrous sodium sulphate, and evaporated to dryness in a rotary evaporator. The supernatant was extracted with EtOAc (1:1, v/v). The EtOAc phase was separated, filtered, and evaporated until dryness as previously described. For solid and semi-solid cultures, the mycelia were covered with Ace and the process continued as described above for mycelia extractions. After obtaining the dry EtOAc fraction, it was dispersed in methanol (MeOH) and transferred to a separatory funnel, where *n*-heptane (*n*-Hep) (1:1, v/v) was added to perform defatting through liquid-liquid extraction. Both fractions (*n*-Hep and MeOH) were recovered and evaporated until dryness.

For the scaled-up cultures, the same procedure took place, but all the organic extractions were done twice. From YM cultures, the supernatant and the mycelia afforded 937 and 111 mg as organic crude extracts, respectively. From BRFT and S-BRFT, 2.4 g and 1.6 g of their respective defatted MeOH fractions were obtained.

The crude extracts and defatted MeOH fractions obtained from the cultures in YM, BRFT and S-BRFT were purified using a combination of different chromatographic procedures as described in the supplementary material (Tables S1–S6). Compounds (**1**–**6**) were isolated from BRFT, (**7**–**9**) from YM and (**10**–**14**) from S-BRFT.

### Chromatographic methods and spectral data

The crude extracts and defatted MeOH fractions were dissolved in Ace:MeOH (1:1, v/v) to a concentration of 4.5 mg/mL while pure compounds were prepared at 1 mg/mL, respectively. They were analyzed on an UPLC system (UltiMate 3000 Series, Thermo Fisher Scientific, Waltman, MA, USA) coupled to an amaZon speed ESI-Iontrap-MS (Bruker Daltonics, Bremen, Germany). The stationary phase used was a C_18_ Acquity UPLC BEH column (50 × 2.1 mm, 1.7 μm, Waters, Eschborn, Germany) kept at 25 °C. For the mobile phase, deionized H_2_O + 0.1% formic acid as solvent A and acetonitrile (MeCN) + 0.1% formic acid as solvent B were used. The gradient was set as: 5% B in 0.5 min, 5–100% B in 20 min and 100% B was kept for 4.5 min with a flow of 0.6 mL/min and an injection volume of 2 μL. The UV-Vis spectral data were detected using a Diode-Array Detector (DAD) at 210 and 190–600 nm. Similarly, HR-ESI-MS spectra were recorded with an Agilent 1200 series HPLC-UV system (Agilent Technologies, Böblingen, Germany; conditions as for ESI-MS) combined with ESI-TOF-MS (Maxis, Bruker Daltonics, Bremen, Germany), scan range *m/z* 100–2500, capillary voltage 4500 V, and dry temperature 200 °C. The results were analyzed using the software Data Analysis 6.1 (Bruker).

For the metabolomics studies each sample was analyzed at a concentration of 450 µg/mL using an UPLC system (Dionex Ultimate 3000RS, Thermo Scientific, Dreieich, Germany) equipped with a C_18_ column (150 × 2.1 mm, 1.7 µm, Kinetex, 100 Å; Phenomenex, Aschaffenburg, Germany) with an injection volume of 2 µL. The mobile phase consisted of A (deionized H_2_O + 0.1% formic acid) and solvent B (MeCN + 0.1% formic acid) at a constant flow rate of 0.3 mL/min. The gradient started with 1% B for 0.5 min, increased to 5% B within 1 min, and reached 100% B over 19 min, holding at 100% B for 5 min. The column temperature was maintained at 25 °C, and UV-Vis spectral data were detected using a DAD in the range of 190–600 nm. Mass spectra were acquired using a trapped ion mobility quadrupole Time-Of-Flight mass spectrometer (timsTOF Pro, Bruker Daltonics, Bremen, Germany) with the following settings: tims ramp time 100 ms, spectra rate 9.52 Hz, PASEF on, cycle time 320 ms, MS/MS scans 2, scan range (*m/z* 100–1800 Da). Mass spectra were acquired in positive ion mode, and raw data were pre-processed with MetaboScape 2022 (Bruker Daltonics, Bremen, Germany) within the retention time range of 1.0–20 min. The obtained features were dereplicated against our in-house database comprising MS/MS spectra of standards from characteristic secondary metabolites of xylarilean taxa (e.g. azaphilones, asterriquinones, binaphthalenes, cytochalasans, macrolides and sesquiterpenoids) in MetaboScape. Detailed procedures for the metabolomic analyses are available in the ESI (Pages S108–S109). The FBMN analysis generated during this study can be found under the GNPS2 Task ID: 8061de20d4034ad8b3ca5f405eb15486.

Chemical structures of the isolated compounds were elucidated through mass spectrometric data, comprehensive 1D (^1^H and ^13^C) and 2 D (^1^H–^1^ H COSY, HMBC, HSQC and ROESY) NMR spectral analyses that were recorded on a Bruker Avance III 500 MHz spectrometer equipped with BBGO (Plus) Smartprobe (^1^H: 500 MHz; ^13^C: 125 MHz) and a Bruker Avance III 700 MHz spectrometer utilizing a 5 mm TCI cryoprobe (^1^H: 700 MHz; ^13^C: 175 MHz). Compounds were dissolved in deuterated chloroform-*d* or DMSO-*d*_6_.

### Antimicrobial and cytotoxicity assays

To evaluate the antimicrobial activity of the isolated metabolites, their Minimum Inhibitory Concentration (MIC) was determined using a serial dilution assay method in 96-well microtiter plates [23, 24], starting with a concentration of 66.6 µg/mL and followed by serial dilutions down to 0.52 µg/mL. These concentrations were chosen to cover the positive controls MIC values, enabling a direct comparison with the evaluated compounds. The compounds were tested against four Gram-negative bacteria: *Acinetobacter baumannii* (DSM 30008), *Escherichia coli* (DSM 1116), *Pseudomonas aeruginosa* (PA14), *Chromobacterium violaceum* (DSM 30191); three Gram-positive bacteria: *Bacillus subtilis* (DSM 10), *Mycobacterium smegmatis* (ATCC 700084), *Staphylococcus aureus* (DSM 346) and five fungi: *Schizosaccharomyces* *pombe* (DSM 70572), *Wickerhamomyces anomalus* (DSM 6766), *Mucor hiemalis* (DSM 2656), *Candida albicans* (DSM 1665), and *Rhodotorula glutinis* (DSM 10134).

To evaluate their cytotoxicity, the half-maximal inhibitory concentration (IC_50_) was determined adopting the previously described method [23, 24]. Compounds were first evaluated against two mammalian cell lines: mouse fibroblasts (L929, DSMZ: ACC 2) and human endocervical adenocarcinoma (KB 3.1, DSMZ: ACC 158). The compounds showing cytotoxicity were further evaluated against five more human cancer cell lines: adenocarcinomic alveolar basal epithelial cells (A549, DSMZ: ACC 107), breast adenocarcinoma (MCF-7, DSMZ: ACC 317), prostate cancer cells (PC-3, DSMZ: ACC 465), squamous cell carcinoma (A431, DSMZ: ACC 91) and ovarian carcinoma (SKOV-3, DSMZ: ACC HTB 77). Epothilone B served as a positive control.

### Screening for biological effects on actin cytoskeleton

To carry out the assays on actin cytoskeleton, the human osteosarcoma cell line (U-2OS, ATCC HTB-96) was cultivated and maintained in DMEM (Life Technologies, Carlsbad, CA, USA) supplemented with 10% FBS (Sigma- Aldrich, St. Louis, MO, USA), 1% minimum essential medium non-essential amino acids (MEM NEAA, Life Technologies, Carlsbad, CA, USA), 1% L-glutamine (Life Technologies, Carlsbad, CA, USA), 1% sodium pyruvate (Life Technologies, Carlsbad, CA, USA), and 1% penicillin-streptomycin (10,000 U/ml, Life Technologies, Carlsbad, CA, USA) under cell culture conditions (37 °C and 7.5% CO_2_).

Compounds (**5, 10,** and **14**) were screened for bioactivity exerted on the actin network in a 1-h endpoint actin disruption assay implementing the previously described protocol [25]. Human osteosarcoma cells (U-2OS, ATCC-HTB-96) were seeded on fibronectin-coated (25 µg/mL, Roche, Mannheim, Germany) cover-slips at a density of 20,000 cells/well and allowed to spread overnight under cell culture conditions. Growth medium was supplemented with the tested compounds based on previously determined IC_50_ values in mouse fibroblasts L929 (1 × IC_50_ = low dose; 5 × IC_50_ = high dose) and added to the seeded cells for 1 h [26]. Moreover, high-dose effects of the tested compounds were probed for their reversibility by replacing the treatment with fresh medium, followed by a recovery period of 1 h. Cells were fixed using 4% pre-warmed *p*-formaldehyde (PFA) supplied in phosphate buffered saline (PBS) for 20 min at 37 °C, washed with pre-warmed PBS, and permeabilized with 0.1% Triton X-100 (Bio-Rad Laboratories, Hercules, CA, USA) in PBS buffer for 1 min at room temperature (rt). After additional wash steps, cells were stained for filamentous actin (F-actin) using Atto488-coupled phalloidin (1:100, ATTO-TEC, Siegen, Germany) in PBS for 1 h at room temperature and mounted in ProLong Diamond Antifade Mountant (Invitrogen, Carlsbad, CA, USA) containing DAPI for nuclear DNA staining. Stained cells were visualized by epifluorescence and recorded using an inverted microscope (Nikon eclipse Ti2, Tokio, Japan) equipped with a 60 times Nikon oil immersion objective (Plan Apofluar, 1.4 NA) using a pco.edge back-illuminated sCMOS camera (Excelitas Technologies, Mississauga, ON, Canada), and a pE-4000 (CoolLED, Andover, UK) as a light source. The system was operated by and images acquired with NIS elements (Nikon, Tokio, Japan) and processed with Image J (NIH, Bethesda, MD, USA).

## Results and discussion

### Fungal identification

Colombia is a megadiverse country, with an estimated of 105,600–300,000 fungal species [27]. However, by 2023, only 7,619 species had been reported and less than 100 had been studied for their biotechnological application [21, 28]. It is worth noting that most of the reports are from the Andean region, which constitutes the urban areas and main cities of the country; while regions such as the Amazon, expected to harbor a rich biodiversity, remain largely unexplored [21]. This strain was found to belong to the genus *Xylaria* based on sequence data of a fragment of the second largest subunit of the DNA directed RNA polymerase II (*rpb2*) and β-tubulin (*tub2*) sequences showing 95% and 94% nucleotide similarity, respectively, with different strains of *X. cubensis*. Regrettably, sequencing of the nuclear rDNA internal transcribed spacer (ITS) and the large subunit nuclear 28S rDNA (LSU) was not successful. However, previous studies have proven the utility of *tub2* as the new primary barcoding marker in the related family Hypoxylaceae and even for other taxa in the Xylariales [29]. Therefore, we present here the first report of a species belonging to the order Xylariales from the Department of Guaviare (Amazon region) [30, 31]. In Colombia, there are 49 reports of species belonging to the genus *Xylaria* distributed in numerous collections according to the ColFungi database [32]. However, the knowledge about this group is still underrepresented in Colombia, considering it is highly diverse in tropical areas [27]. In fact, further efforts will be needed to clarify the taxonomic placement of the isolated strain, as the provided data herein is insufficient to abstain its classification at the species level.

### Screening and isolation of secondary metabolites from conventional media

**Fig. 1:**
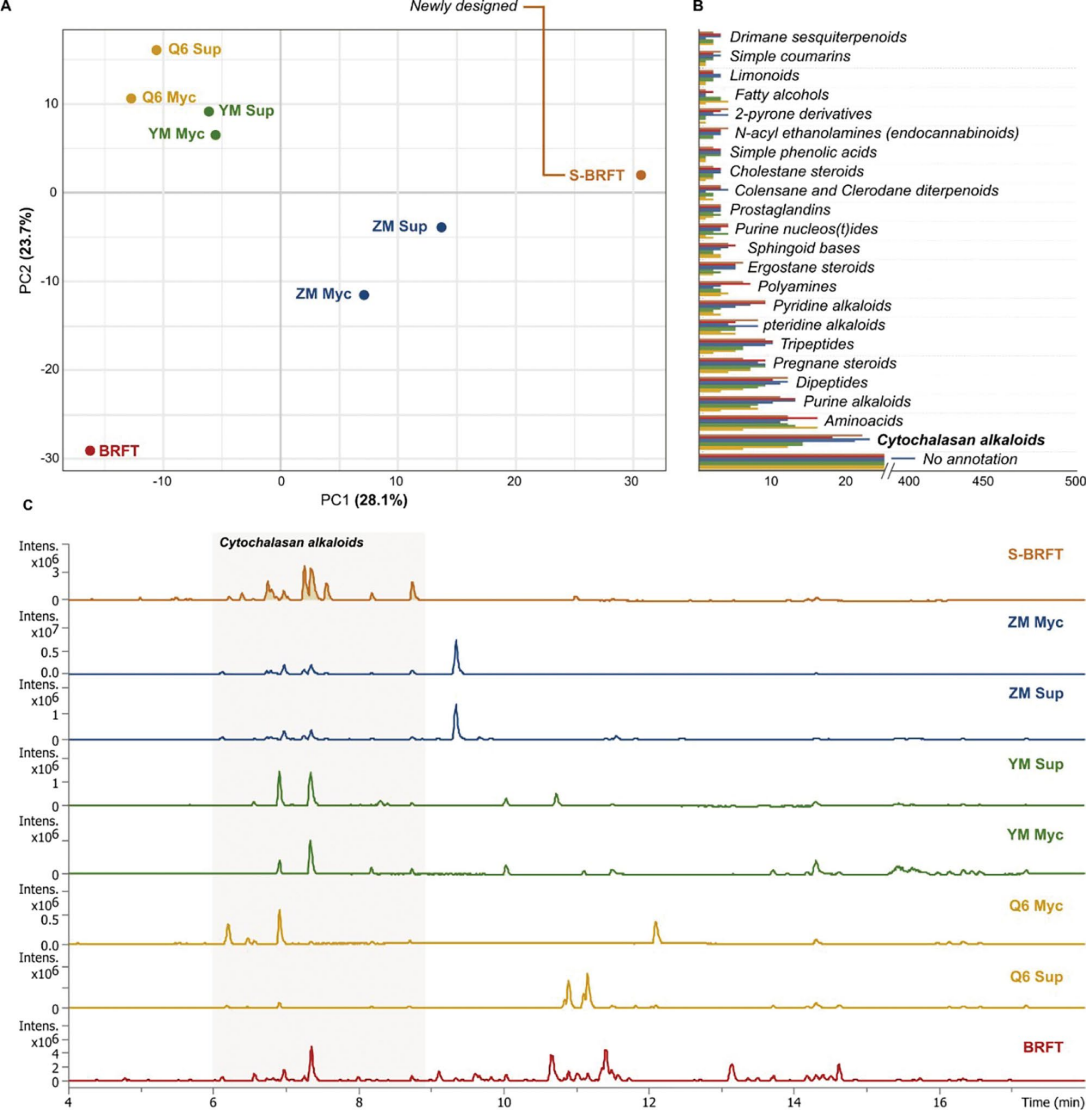
**A** Principal Component Analysis (PCA) score plot of the secondary metabolites produced by *Xylaria* sp. CM-UDEA-H199 when cultured in YM 6.3 (green), Q6½ (yellow), ZM½ (blue), BRFT (red), and the newly designed medium S-BRFT (brown); where Sup and Myc correspond to the supernatant and mycelia crude extract, respectively. **B** Bar plot indicating the number of MS2 features for the most abundant natural product classes (n = 23) within the different crude extracts as predicted from the CANOPUS analysis. The most abundant natural product class, recognized as cytochalasan alkaloids, is highlighted in bold letters. **C** Base peak chromatograms (BPC) from UHPLC-MS analysis of the crude extracts obtained from the cultivation of *Xylaria* sp. CM-UDEA-H199 in the evaluated media. The highlighted section corresponds to the typical retention time frame from cytochalasan alkaloids

As members of the Xylariales represent a great source of diverse chemical entities with promising biological properties, the production of SMs by the fungus *Xylaria* sp. CM-UDEA-H199 was evaluated through its cultivation on three different liquid media (YM 6.3, ZM½, Q6½) and one solid-state rice medium (BRFT). Since as shown in previous research [33–36], these culture media have been frequently used in fungal studies. We used a metabolomics-based approach to gain insights into the chemical diversity observed with different cultivation media. The principal component analysis (PCA) from the 902 MS/MS detected features (Total MS features = 1226) revealed varying similarities among the crude organic extracts obtained from each medium (Fig. 1a).

To further inspect the metabolite diversity within the obtained crude extracts, we used CANOPUS to predict de novo the respective natural product classes based on their MS/MS spectra [37]. Despite more than half of the features were unmatched to any natural product class, the most abundant SMs class corresponded to cytochalasan alkaloids originating from hybrid PKS-NRPS pathways (Fig. 1b). Cytochalasan derivatives were mainly detected in the extracts from ZM½, yet not exclusively, as they were also detected at varying levels in other extracts. By comparing the extracts’ yields, even when ZM½ exhibited a higher number of cytochalasins, it was found that the highest yields were obtained from BRFT and YM media. In fact, it can also be observed in the base peak chromatograms (BPC) from the obtained extracts that the relative intensity of the compounds within the retention time expected for cytochalasan alkaloids is lower in ZM½ when compared to BRFT or YM (Fig. 1c). Therefore, these two media were first selected for scale-up cultivation and purification of their SMs.

**Fig. 2:**
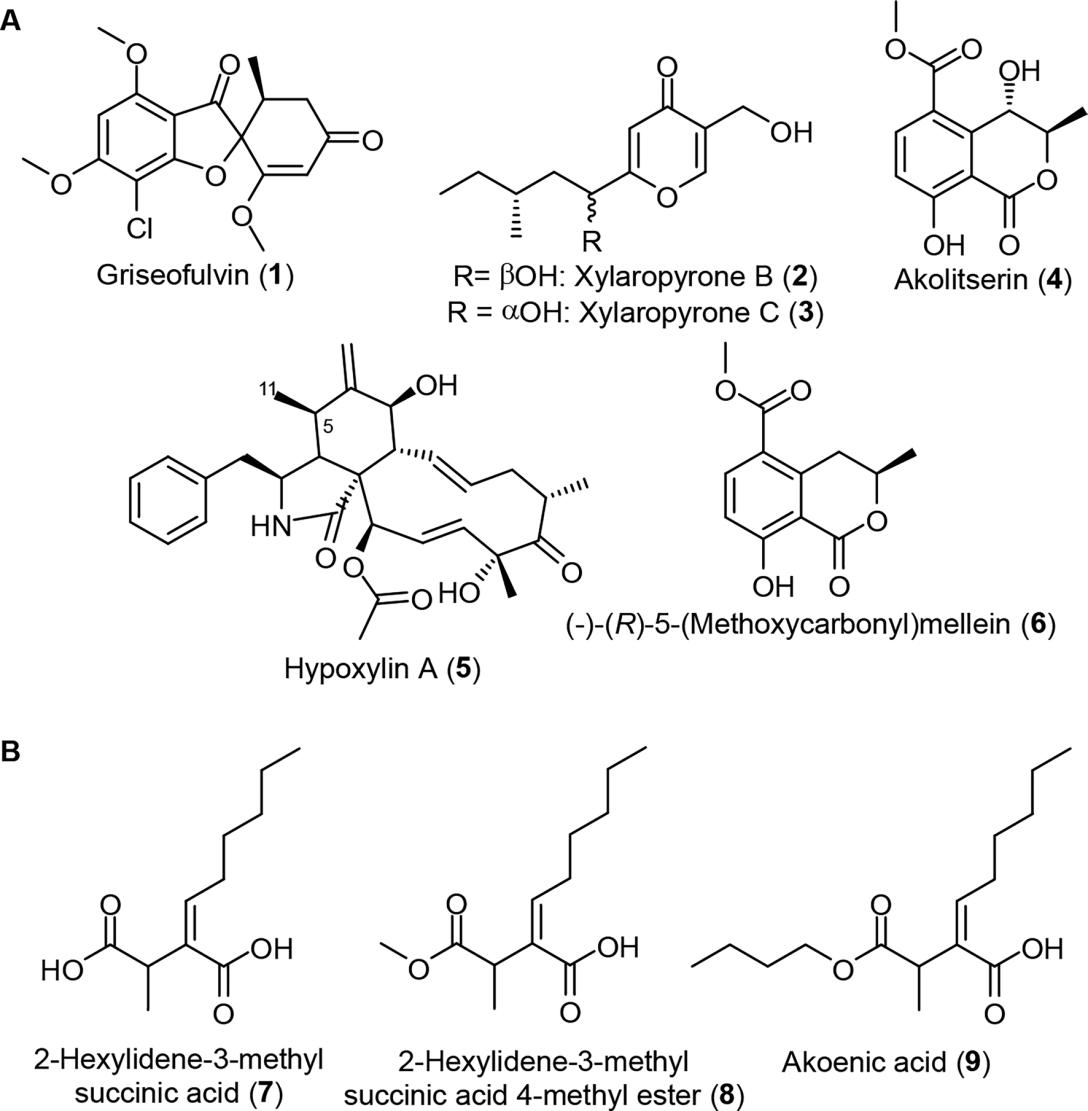
Chemical structures of the isolated compound from *Xylaria* sp. CM-UDEA-H199. **a** BRFT fermentation: Compounds (**1**–**6**). **b** Submerged YM fermentation: Compounds (**7**–**9**)

The crude extracts obtained from the scaled-up cultivation in YM and BRFT media were purified using several preparative HPLC purification steps. These were conducted as described in Tables S1–S6 to obtain compounds (**1**–**9**). Thereafter, chemical structures of the isolated compounds (Fig. 2) were elucidated based on comprehensive spectroscopic analyses including HR-ESI-MS, 1D and 2D NMR spectra in addition to the comparison with reported literature (see supplementary material Table S7-S14). Based on the obtained results, six compounds (Fig. 2) were unambiguously elucidated from the crude extract of the BRFT fermentation.

The isolated compounds were identified as griseofulvin (**1**) (Table S7) [38], a mixture of xylaropyrones B/C (**2/3**) (Table S8) [39], akolitserin (Table S9) (**4**) [40], 5-*epi*-cytochalasin D named hypoxylin A (**5**) (Table S10) [41] and (–)-(*R*)-5-(methoxycarbonyl)mellein (**6**) (Table S11) [40]. The submerged YM fermentation afforded three pure compounds whose identities were determined as 2-hexylidene-3-methyl-succinic acid (**7**) (Table S12) [42], its 4-methyl ester (**8**) (Table S13) [42] and akoenic acid (**9**) (Table S14) [40].

### S-BRFT medium enhances cytochalasins production

Based on the PCA analysis, we identified *Xylaria* sp. CM-UDEA-H199 as a potential producer of diverse SMs, with a notable production of different cytochalasans, bearing a phenylalanine moiety and thus termed as cytochalasins. Therefore, we decided to explore a different approach and modify the BRFT medium to induce nutrient limiting conditions considering that when the availability of key nutrients reach a state of limitation during the cultivation, secondary metabolism in fungi might be tuned to specific SM pathways [16].

Cultivation on a solid-state rice medium is one of the most common practices during fungal natural products screening campaigns. It promotes an increased diversity of secondary metabolites under laboratory conditions; mainly by simulating the natural environment of the fungus [43]. Furthermore, brown rice, specially, also represents a rich nutrient source, as it is constituted by its whole grain, which is a complex composition made of carbohydrates, including polysaccharides such as starch and simple sugars as glucose, xylose and arabinose [44, 45]. This substrate also provides an important source of fiber, antioxidants, proteins and different minerals (i.e. magnesium, potassium, calcium, zinc and copper) and vitamins (i.e. vitamin E and B); among others [46]. Accordingly, we decided to re-formulate the well-known BRFT medium, considering the provided nutrient supply; but in parallel promoting a presumed nutrient limitation state based on the quantity of substrate provided. The modified S-BRFT medium contained 7 times less rice and 2.6 times more yeast extract than the original BRFT medium, while maintaining the same amount of additional micronutrients and the same volume of deionized water (100 mL) before autoclaving. The amount of brown rice was reduced to limit carbon source, while yeast extract was increased aiming for an excess of nitrogen. It might be noted that even when yeast extract and rice are considered as nitrogen and carbon sources, respectively, they both provide each element simultaneously but at different proportions.

During the design of this new medium, additional factors beyond the formulation were also taken into consideration. First, rice grains were ground to obtain rice flour; this to increase the area of contact between the fungus and the substrate. Moreover, brown rice presents an unequal distribution of the nutrients throughout the grain, considering its structure, from outside to inside: bran, endosperm and germ or embryo [47, 48]. Usually, fungal growth occurs mainly on the surface of the grain which means inner nutrients corresponding to the embryo or germ are partially if not consumed. After grinding, the access to these nutrients was less restricted. When using small amounts of the obtained rice flour (4 g in 100 mL), the consistency of the medium changed from solid to semi-solid, resulting in surface enlargement, which enabled a higher oxygen supply transfer into the medium by increasing the agitation speed. The latter also promoted better contact between the fungus and the medium components. These factors have to be considered as they also influence the activation of the metabolism and secondary metabolite production [45].

Conventional studies of media reformulation aim to optimize the production of specific compounds when changing the ratio or concentration of the nutrients [18, 19, 46, 47]. Herein, we studied the complete metabolic response of the fungus to the newly formulated medium; based on the OSMAC concept, which states that different metabolic profiles can be obtained from one strain when cultured under different conditions [15].

**Fig. 3:**
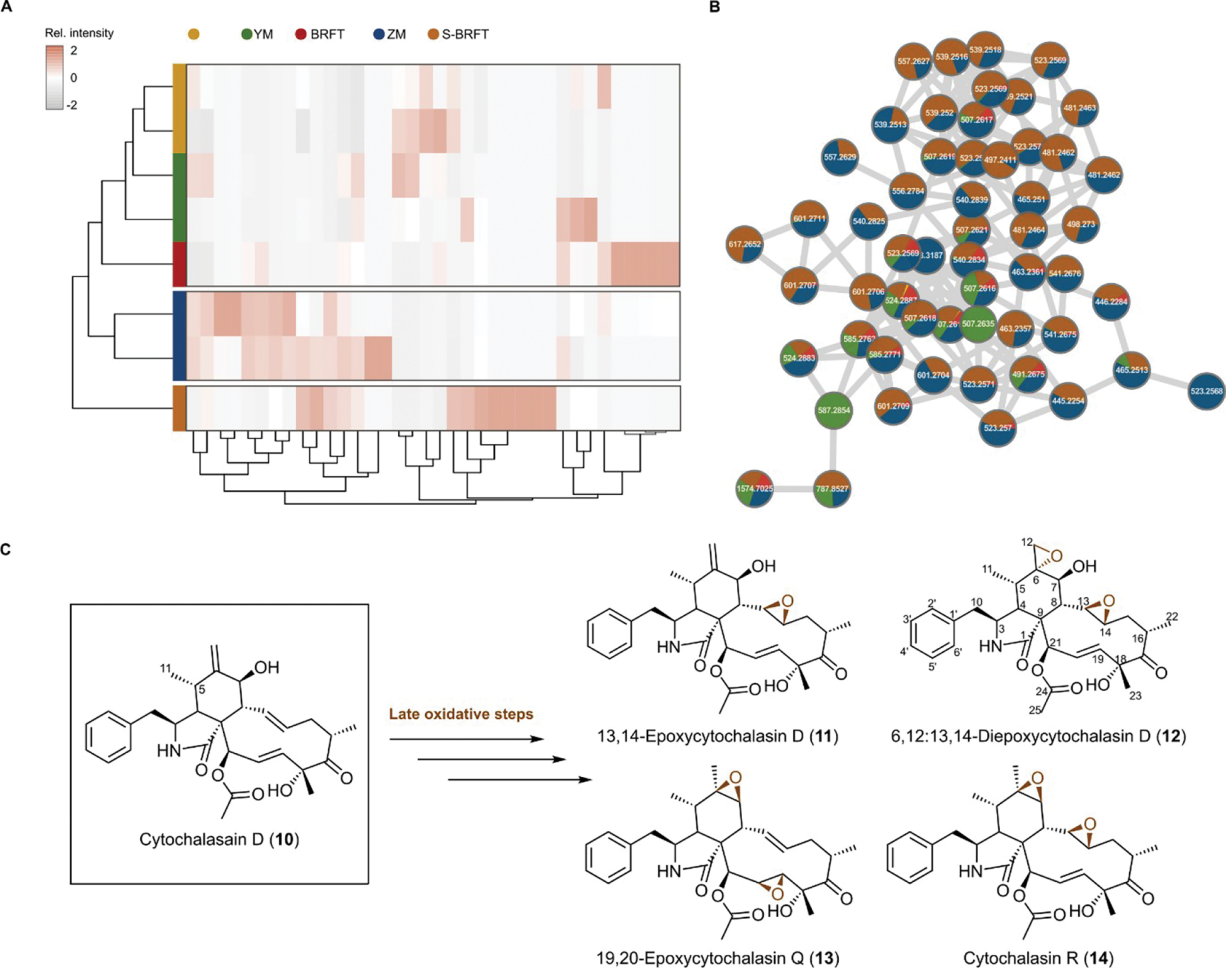
**A** Heatmap following a hierarchical clustering of features classified as cytochalasin alkaloids by CANOPUS. The heatmap displays the feature relative abundance within the crude extracts obtained from the evaluated media. The heatmap with dendrograms was generated by the R package heatmap. **B** Cytochalasins molecular family obtained from the feature-based molecular networking of the crude extracts obtained from the cultures of *Xylaria* sp. CM-UDEA-H199, with pie charts representing each medium where the spectra from the respective feature was detected. **C** Chemical structures of the isolated compounds after the cultivation of *Xylaria* sp. CM-UDEA-H199 in the newly designed semi-solid “Slurry” S-BRFT medium (**10**–**14**)

Metabolomic analysis of the crude extract obtained after fermentation in S-BRFT showed the production of a distinct metabolome compared to those produced in other evaluated media (Fig. 1a). Remarkably, the observed differences correlated with an increased diversity of cytochalasins, as initially suggested by CANOPUS predictions (Fig. 1b), alongside a higher production yield for this class of SMs (Fig. 1c). Similarly, our hierarchical cluster analysis (HCA) revealed a clear distinction between all evaluated media and the newly formulated S-BRFT when referring to the production of putatively annotated cytochalasins (Fig. 3a). Additionally, HCA illustrated how the fermentation setup in S-BRFT influences cytochalasin-associated production patterns across different cultivation media. Interestingly, while compounds produced in BRFT closely resembled those obtained from liquid fermentation media Q6½ and YM, metabolites from ZM½ showed greater similarity to those produced in S-BRFT, despite differences in nutrient composition.

Moreover, while several of the detected cytochalasins were produced as well in ZM½, they were significantly more abundant in the S-BRFT as shown by the feature-based molecular networking analysis (Fig. 3b). Despite that, the cultivation on ZM½ promoted a higher number of cytochalasins, as suggested by de novo annotation using CANOPUS, these were not the major metabolites produced in this medium. In contrast, cultivation on S-BRFT seemed to promote the production of nearly only this class of molecules (Fig. 1c). It is worth noting that despite the advantages offered by current MS/MS-based approaches, it is important to consider that the production yield is a critical parameter beyond the mere detection of potentially interesting candidates. Interestingly, the detected cytochalasins, as shown in their respective molecular family (MF), exhibited a high number of isobaric features and mass differences explained by oxidative modifications within the same carbon skeleton (Figure S83). MS/MS dereplication using our in-house compound library pointed towards the production of cytochalasin-D-like metabolites; however, the different isobars could not be initially identified. Therefore, to discern the chemical nature of the observed cytochalasins in the newly designed S-BRFT, scaled-up cultivation on this medium was conducted.

The chromatographic workups of the semi-solid or “Slurry” fermentation, denoted as S-BRFT, yielded five different cytochalasin derivatives. Their chemical structures were elucidated based on HR-ESI-MS, comprehensive 1D/2D NMR spectroscopic analyses and by comparison with the reported literature. The isolated compounds (Fig. 3) included majorly cytochalasin D (**10**) (Table S15) [49, 50], its 13,14-epoxy derivative (**11**) (Table S16) [51], 19,20-epoxycytochalasin Q (**13**) (Table S18) and cytochalasin R (**14**) (Table S19) [52–54] in addition to 6,12:13,14-diepoxycytochalasin D (**12**) (Table S17) [55]. To the best of our knowledge and according to the reported literature, compound **12** is herein reported for the first time as a natural diepoxycytochalain derivative that was previously described as a synthetic derivative resulting from a chemical derivatization reaction of cytochalasin D using *ter*-butylhydroperoxide [55].

**Fig. 4:**
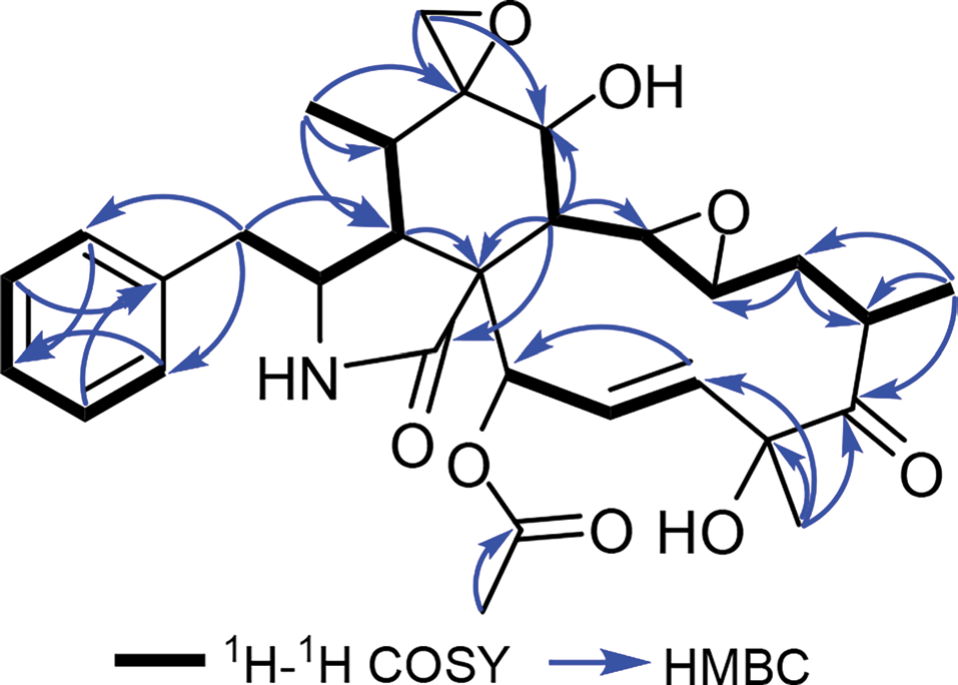
Key ^1^H–^1^ H COSY and HMBC correlations of **12**

Compound **12** was obtained as a colourless amorphous solid. Its molecular formula was determined as C_30_H_37_NO_8_ indicating thirteen degrees of unsaturation. The ^1^H NMR spectral data (Table 2, Figure S62) showed proton signals of a monosubstituted phenyl moiety at δ_H_ 7.14 (d, *J* = 7.0 Hz, H-2’,6’), 7.33 (t, *J* = 7.5 Hz, H-3’,5’) and 7.27 (t, *J* = 7.5 Hz, H-4’). In addition, the ^1^H NMR spectrum also revealed the presence of two doublet methyl groups at δ_H_ 0.63 (d, *J* = 7.0 Hz, H3-11) and 1.19 (d, *J* = 6.8 Hz, H_3_-22) in addition two singlet methyl groups at δH 1.55 (H_3_-23) and 2.29 (H_3_-25). It also unravelled one *trans* double bond moiety at δ_H_ 5.65 (dd, *J* = 15.7, 2.6 Hz, H-19) and 6.36 (dd, *J* = 15.7, 2.4 Hz, H-20). The ^13^C chemical shifts (Table 1) were assigned based on 2D NMR spectra (Figure S5–S6) and they revealed one ketone at δ_C_ 212.3 (C-17) and two carboxyl carbon atoms at δ_C_ 174.0 (C-1) and 169.7 (C-24). Based on the obtained results and by comparing with the reported literature, compound **12** revealed a close resemblance to 6,12:13,14-diepoxycytochalasin D [55] that was previously reported as a synthetic derivative of cytochalasin D produced by epoxidation of cytochalasin D (**10**) using *ter*-butylhydroperoxide [55]. The depicted structure of **12** was further confirmed by the ^1^H–^1^ H COSY spectrum (Fig. 4, S63) that revealed four characteristic spin systems of cytochalasins including: (1) a monosubstituted phenyl moiety H-2’,6’/ H-3’,5’/ H-4’; (2) H_2_-10/H-3/H-4/H-5/H_3_-11; (3) H-7/H-8/H-13/H-14/H_2_-15/H-16/H_3_-22; and (4) H-19/H-20/H-21. The ^1^H NMR and the HSQC spectra of **12** revealed a two geminally coupled protons at δ_H_ 2.68/2.83 (d, *J* = 4.7 Hz, H-12a/H-12b) that were directly correlated to a secondary *sp*^3^ carbon at δ_c_ 49.3 suggesting there presence in an spiroepoxy ring. To confirm the position of the spiroepoxy ring, the HMBC spectrum of **12** was acquired (Fig. 4, S64) and it revealed key correlations from H-12a/H-12b to two carbon signals at δc 72.2 (C-7) and δ_c_ 59.3 (C-6) that in turn was correlated to a doublet methyl signal at δ_H_ 0.63 (d, *J* = 7.0 Hz, H_3_-11).

**Table 1:** ^1^H and ^13^C NMR data of 6,12:13,14-diepoxycytochalasin D (**12**)

pos.	δC, ^a^ type	δH^b^ multi (J in Hz)	pos.	δC, ^a^ type	δH^b^ multi (J in Hz)
1	174.0, CO	–	16	38.0, CH	2.92 m
2-N*H*	–	5.68 br s	17	212.3, CO	–
3	54.5, CH	3.53 m (overlapped)	18	77.2, C	–
4	49.3, CH	2.26 dd (5.7, 2.8)	19	129.6, CH	5.65 dd (15.7, 2.6)
5	29.1, CH	2.45 q (6.6)	20	131.7, CH	6.36 dd (15.7, 2.4)
6	59.3, C	–	21	75.6, CH	5.79 t (2.5)
7	72.7, CH	3.76 dd (8.9, 2.0)	22	20.4, CH_3_	1.19 d (6.8)
8	45.4, CH	1.86 t (8.7)	23	24.2, CH_3_	1.55 s
9	52.5, C	–	24	169.7, CO	–
10	44.8, CH_2_	α 2.82 dd (13.5, 4.4) β 2.87 dd (13.5, 5.5)	25	20.8, CH_3_	2.29 s
11	10.0, CH_3_	0.63 d (7.0)	1’	136.8, C	–
12	49.3, CH_2_	α 2.68 d (4.7) β 2.83 d (4.7)	2’,6’	129.0, CH	7.14 d (7.0)
13	59.6, CH	3.63 dd (8.8, 2.0)	3’,5’	129.1, CH	7.33 t (7.5)
14	59.0, CH	2.64 dt (9.8, 2.3)	4’	127.3, CH	7.27 t (7.5)
15	37.8, CH_2_	α 1.65 dt (14.7, 10.4) β 2.05 d (14.3)			

^a^ Assigned based on HMBC and HSQC spectra. ^b^ Measured in chloroform-*d* at 700 MHz

**Table 2:** Half-maximal inhibitory concentration (IC_50_) of **1**–**14** against the tested cell lines

	IC_50_ (µM)						
Compound	Test cell line						
	L929	KB3.1	PC-3	MCF-7	SKOV-3	A431	A549
**1**	5.11	0.94	n.t.	n.t.	n.t.	n.t.	n.t.
**2**/**3**	n.a.	n.a.	n.t.	n.t.	n.t.	n.t.	n.t.
**4**	n.a.	n.a.	n.t.	n.t.	n.t.	n.t.	n.t.
**5**	0.14	0.05	0.10	0.07	0.04	0.10	0.13
**6**	33.46	11.01	13.13	29.65	6.78	17.79	13.98
**7**	n.a.	n.a.	n.t.	n.t.	n.t.	n.t.	n.t.
**8**	41.64	30.25	35.51	53.60	14.47	39.89	38.14
**9**	n.a.	n.a.	n.a.	n.a.	n.a.	n.a.	n.a.
**10**	0.75	0.12	n.t.	n.t.	0.10	0.24	0.57
11*	1.2	0.1	0.5	0.3	0.2	0.3	0.4
**12**	n.t.	n.t.	n.t.	n.t.	n.t.	n.t.	n.t.
13*	0.5	0.1	0.1	0.6	0.1	0.2	0.2
**14**	3.63	2.10	2.10	5.54	1.47	3.63	4.20
Epothilone B (nM) (positive control)	2.17	0.07	0.24	0.07	0.57	0.26	0.05

n.a.: no activity. n.t.: not tested. *: values reported by Lambert et al. [58]

Accordingly, the spiroepoxy ring was confirmed to be positioned at C-6. Based on the obtained NMR spectral data of **12** compared to those reported for 6,12-epoxy- and 6,12:13,14-diepoxycytochalasin D (Table S17) [49, 55] together with having a common biosynthetic pathway with other cytochalasins, the absolute configuration of **12** was suggested to be (3*S*,4*R*,5*S*,6*R*,7*S*,8*R*,9*R*,13*R*,14*R*,16*S*,18*R*,21*R*). According to the aforementioned results, compound **12** was identified as a previously undescribed natural diepoxide cytochalasin derivative named 6,12:13,14-diepoxycytochalasin D. It is worth mentioning that compound **12** could be traced back in the chromatogram of the crude extract derived from S-BRFT fermentation medium confirming its authenticity as a genuine natural product rather than being an artefact formed during chromatographic workup and separation procedures.

Overall, the solid-state BRFT medium allowed the fungus to mainly synthesize metabolites originating from varied polyketide pathways. However, when the fungus was cultured on the newly designed S-BRFT medium, the main products were cytochalasins (Fig. 1c). In fact, the key difference between the isolated cytochalasins from the S-BRFT medium is attributed to their oxidation patterns within the same core scaffold, indicating that they all had the same number of carbon and nitrogen atoms. This suggests a positive correlation between increased oxygen supply and/or nutrient limitation and the activation of oxidative tailoring enzymes acting within the same carbon skeleton. Generally, several of these oxidative steps are carried out by different cytochrome P450 monooxygenases, whose action depends on the intracellular redox potential, oxygen level, and availability of cofactors such as iron. The high number of isobaric cytochalasins, including the isolated compounds (**11**–**14**), indicates the presence of different tailoring enzymes within the corresponding BGC. This is further illustrated by the presence of compound **12**, a diepoxide-cytochalasin, or **14**, a 13,14-epoxycytochalasin named cytochalasin R, suggesting the occurrence of an unusual specific P450 enzymes acting at diverse positions of the core scaffold or promiscuity within these tailoring steps [56]. In addition, the isolation of hypoxylin A (**5**) only from BRFT presents a rare phenomenon, as this 5-epimer of the cytochalasin D (**10**) was not found in the crude extracts derived from the cultivation on S-BRFT.

From a bioprocess perspective, these results offer key insights into cytochalasins production, particularly highlighting the role of oxygen supply, regulated through agitation and media rheology, in enhancing yield. Moreover, the increased and selective production of these metabolites using a more resource-efficient medium composition underscores the potential for optimizing this bioprocess in terms of both efficiency and scalability.

### Bioassay results

None of the isolated compounds exhibited inhibitory effects against the tested bacteria, while only compounds **4**–**6**, **10** and **11** showed mild inhibition against some tested fungal strains. Compounds **4** and **5** had a MIC value of 66.6 µg/mL against *Mucor hiemalis*; **5** also exhibited a MIC value of 16.6 µg/mL against *Schizosaccharomyces pombe*. Compound **6** presented a MIC value of 66.6 µg/mL against *Rhodotorula glutinis*. In addition, compound **10** showed an MIC value of 33.3 µg/mL against *S. pombe* and compound **11** presented an MIC value of 66.6 µg/mL against *Candida albicans* and *S. pombe* (results shown in supplementary material, Table S20).

Compounds **5**, **6, 8, 10, 11, 13** and **14** exhibited mild cytotoxic activity (Table 2) against all tested cell lines. Compound **1** exhibited cytotoxicity against L929 and KB3.1. Among the isolated compounds, hypoxylin A (**5**) revealed the most potent cytotoxic activity against all cell lines, followed by epoxycytochalasin Q (**13**) and cytochalasin D (**10**). Moreover, **5, 10, 11** and **13** presented significantly higher cytotoxicity against mouse fibroblast (L929) than the positive control. It is worth restating that compounds **5** and **10**–**14** were identified as cytochalasins, which are commonly known for their cytotoxic potential [57]. The cytotoxicity values for compounds **11** and **13** were referenced from Lambert et al. [58], since the experiments were conducted in the same laboratory and following the same experimental procedures and standards. The antimicrobial and cytotoxic activity of compound **12** were not tested, since the amount isolated was not sufficient to carry out the experiments.

Cytochalasans are widely known as actin polymerization inhibitors in eukaryotic cells. Since actin not only plays a decisive role in the metastasis of cancer cells [59] but also in viral and bacterial host-invasion-processes [60], cytochalasans gained interest as potential drug candidates. Although intensively studied for decades, no drug that target the actin cytoskeleton has reached the clinical phase because of severe and non-selective cytotoxic effects in both, cancer and healthy tissue cells [61]. To date, comprehensive data analyzing of chemical features in the cytochalasan backbone along with their bioactivity is still incomplete [7]. However, more and more studies have been devoted in the last five years to shed light on this topic [58]. This was the reason behind our aim to utilize the obtained cytochalasins in particular compounds (**5, 10** and **14**) for advanced structure-activity relationship (SAR) study on actin dynamics.

Being a 5-epimer of the well-studied actin inhibitor cytochalasin D (**10**), hypoxylin A (**5**) was investigated to compare its potency against the actin cytoskeleton. Likewise, we characterized the bioactivity of cytochalasin R (**14**) featuring two epoxy groups attached to C-6/7 and C-13/14 in an otherwise unchanged backbone if compared to **10**. To screen for filamentous actin (F-actin) network disruption activity, human osteosarcoma cells (U-2OS) were treated with low (= 1 × IC_50_) and high (= 5 × IC_50_) dose concentrations of compounds (**5, 10** and **14**), and stained for F-actin using fluorescently-coupled phalloidin as previously described [25].

**Fig. 5:**
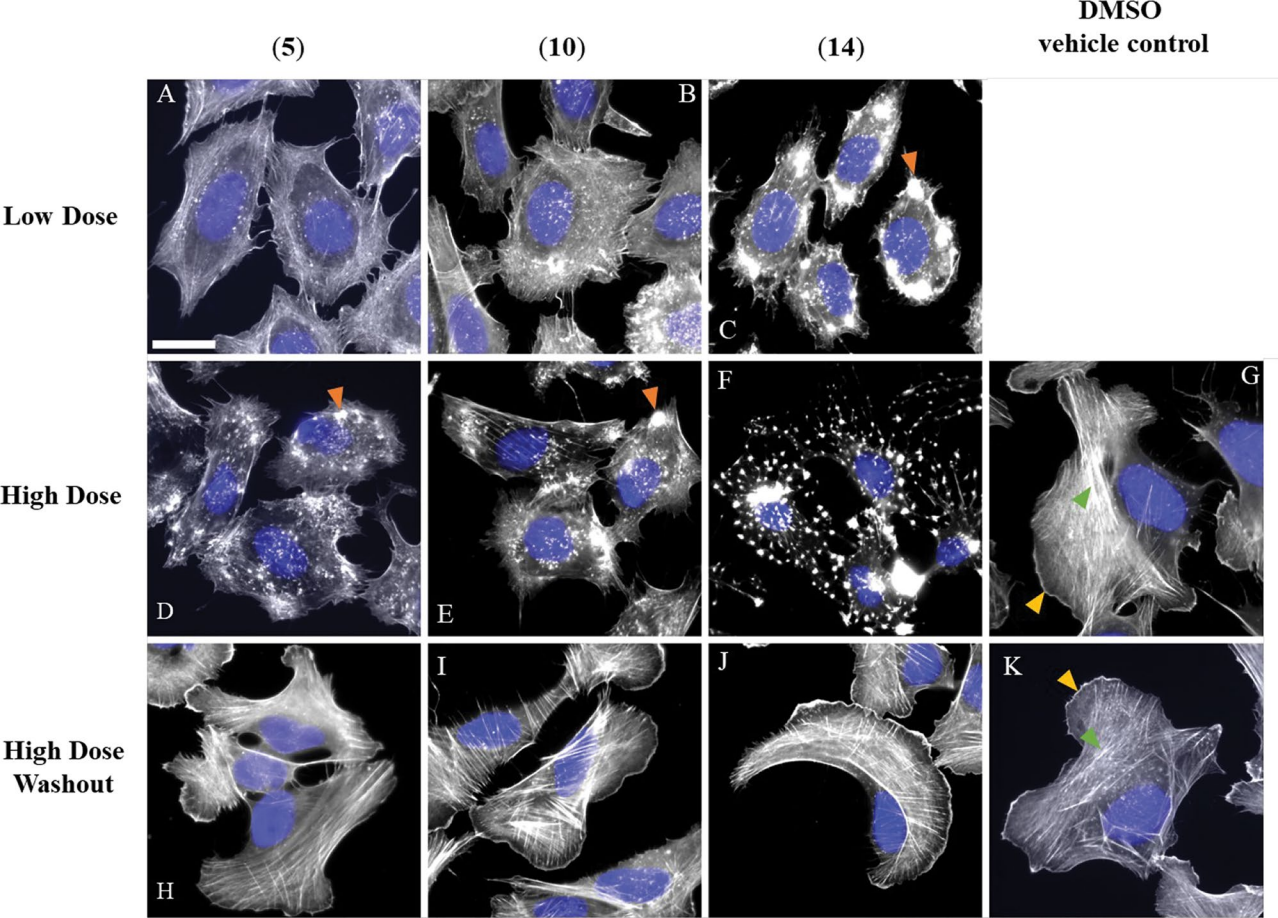
Overlay images of human osteosarcoma cells (U-2OS) treated with **5**, **10** and **14** compared to DMSO as vehicle control (G). Low dose (**a**–**c**) and high dose (**d**–**g**) concentrations correspond to 1 × and 5 × IC_50_ in L929 fibroblasts, respectively. Cells were stained for filamentous actin (F-actin) using AlexaFluor488-coupled phalloidin (greyscale) and DAPI for nuclear DNA (pseudocolored in blue). Orange arrowheads indicate distinct actin accumulations induced by high dose treatment of **5** and **10**. Corresponding high dose washout experiments (**h**–**k**) resulted in F-actin staining patterns indifferent from the DMSO control, exhibiting F-actin-rich structures like lamellipodia (yellow arrowheads) and stress fibers (green arrowheads) that are highlighted in (**g** and **k**). This confirms full reversibility after 1 h recovery time. Representative scale bar in (**a**) corresponds to 25 µm

Reversibility of high dose-induced actin reorganization was assessed by additional wash-out steps followed by a 1-h recovery period in fresh culture medium. Low dose concentration of **5** and **10** (Fig. 5a and b) reduced prominent actin structures (Fig. 5g) such as lamellipodia-F-actin rich protrusions at the cell periphery (yellow arrowheads)—and stress fibers—contractile bundles of antiparallel organized F-actin (green arrowheads), clearly visible in DMSO-treated U-2OS cells. Corresponding high dose concentrations led to massive disruption of the actin network, manifested in distinct large F-actin rich accumulations (Fig. 5d and e, orange arrowheads). In contrast, low concentrations of **14** induced comparable effects on actin if compared with high dose treatments of **5** and **10** (Fig. 5c). Further increasing the treatment concentration of **14** amplified the staining pattern of large, to stellar or knot-like F-actin rich structures (Fig. 5f). Medium exchange following high dose treatment with **5, 10** and **14** resulted in cells indiscernible from the DMSO control, confirming full reversibility. Taken together, (i) we observed unaltered cytotoxicity (**5**: 0.14 µM, **10**: 0.15 µM) and actin disruption activity of **5** compared to **10**, leading to the conclusion that the C-5 methyl stereochemistry is not crucial for bioactivity. (ii) The degree of actin network reorganization strongly increases upon **14** treatment compared to **5** and **10**, although its cytotoxicity was diminished (3.63 µM). Thus, we assume that the implementation of epoxy groups between C-6/7 and C-13/14 as found in **14** might be causative for the observed correlation between cytotoxicity and actin activity. Concurring, Stadler and co-workers recently described that epoxidation of (**10**)—leading to epoxycytochalasin derivatives including cytochalasin R (**14**)—enhanced its actin activity in U-2OS cells on one hand, but diminished cytotoxicity in L929 mouse fibroblasts (from 0.15 µM to 1.9 µM) on the other. Accordingly, it is tempting to hypothesize that the cytotoxicity of cytochalasins might be separated from its activity on actin [58].

## Conclusions

The results presented in this study provide valuable insights into the biotechnological potential of fungal species from the Amazon region of Colombia, encouraging the need for further systematic exploration of the biodiversity in these neglected areas. Particularly, we evidenced that different fermentation configurations and strategies, with an emphasis on nutrient limitation can significantly alter the production profile of bioactive secondary metabolites. For the studied *Xylaria* sp. CM-UDEA-H199, a complete variation in its metabolome was observed when cultured on the newly designed semi-solid “Slurry” S-BRFT medium compared to the conventional solid-state rice (BRFT) medium. Consequently, cytochalasins production was highly promoted through nutrient limitation and increased oxygen availability, resulting in highly epoxidized cytochalasins. Thus, we suggested that in response to the employed fermentation strategy, the activation of diverse enzymes will be an interesting topic for our concurrent studies on the biosynthetic diversity of cytochalasin pathways. In summary, the presented approach represents a first step toward the future bioprocess optimization for sustainable cytochalasins production, promising natural products for developing future therapeutic agents.

## Electronic supplementary material

Below is the link to the electronic supplementary material.

Supplementary Material 1

## Data Availability

All datasets generated during this study are provided in the manuscript or the supplementary information.
