# Supplementary material for: Unconventional semi-solid cultivation enhances cytochalasins production by the Colombian fungus Xylaria sp. CM-UDEA-H199
Source: BMC Biotechnol. 2025 Jul 1;25:57. doi: 10.1186/s12896-025-00978-2 (PMC12220629; doi:10.1186/s12896-025-00978-2)
Supplement: Supplementary file 1 — Supplementary Material 1 [file 12896_2025_978_MOESM1_ESM.pdf]

SUPPLEMENTARY MATERIAL FOR:

Unconventional semi-solid cultivation enhances cytochalasins production by the Colombian fungus *Xylaria* sp. CM-UDEA-H199

Daniela Valencia-Revelo[1,2], Esteban Charria-Girón[1,3], Katharina Schmidt[4], Silke Reinecke[1], Aida M. Vasco-Palacios[5], Theresia Stradal[4], Yasmina Marin-Felix[1,3], Nelson H. Caicedo-Ortega[2,6]* and Sherif S. Ebada[1,7]*

[1] Department Microbial Drugs, Helmholtz Centre for Infection Research (HZI), Inhoffenstrasse 7, 38124 Braunschweig, Germany

[2] Departamento de Ciencias Biológicas, Bioprocesos y Biotecnología. Facultad de Ingeniería, Diseño y Ciencias Aplicadas, Universidad Icesi, Calle 18 No. 122-135, Cali, Colombia

[3] Institute of Microbiology, Technische Universität Braunschweig, Spielmannstrasse 7, 38106 Braunschweig, Germany

[4] Department of Cell Biology, Helmholtz Centre for Infection Research (HZI), Inhoffenstrasse 7, 38124 Braunschweig, Germany

[5] Grupo de Microbiología Ambiental y Grupo BioMicro, Escuela de Microbiología, Universidad de Antioquia UdeA, Calle 67 No. 53-108, Medellín, Colombia

[6] Centro BioInc, Universidad Icesi, Calle 18 No. 122-135, Cali, Colombia

[7] Department of Pharmacognosy, Faculty of Pharmacy, Ain Shams University, 11566 Cairo, Egypt

* Corresponding authors: nhcaicedo@icesi.edu.co (Nelson H. Caicedo-Ortega); Tel: +57 3187548041 sherif.elsayed@helmholtz-hzi.de; sherif_elsayed@pharma.asu.edu.eg (Sherif S. Ebada); Tel.: +49-531-6181-4267; Fax +49-531-6181-9499

TABLE OF CONTENTS

#	Contents	Page
**1**	Purification schemes.	**S5**
**2**	Table S1. Purification method for BRFT cultures.	**S5**
**3**	Table S2. Purification method for YM cultures.	**S6**
**4**	Table S3. Pre-purification method for S-BRFT cultures.	**S6**
**5**	Table S4. Purification method for fractions obtained from flash chromatography (MeOH_S-BRFT).	**S7**
**6**	Table S5. Purification method for fractions obtained from NP-HPLC (MeOH_S-BRFT).	**S8**
**7**	Table S6. Purification method for PSF2.A obtained from RP-HPLC (MeOH_S-BRFT).	**S9**
**8**	Figure S1. LR-ESI-MS of **1**.	**S10**
**9**	Figure S2. HR-ESI-MS of **1**.	**S11**
**10**	Figure S3. ^{1}H NMR spectrum of **1** in DMSO-d_6 at 500 MHz.	**S12**
**11**	Figure S4. ^{1}H–^{1}H COSY spectrum of **1** in DMSO-d_6 at 500 MHz.	**S13**
**12**	Figure S5. HMBC spectrum of **1** in DMSO-d_6 at 500 MHz.	**S14**
**13**	Figure S6. HSQC spectrum of **1** in DMSO-d_6 at 500 MHz.	**S15**
**14**	Table S7. ^{1}H and ^{13}C NMR data of **1** and griseofulvin.	**S16**
**15**	Figure S7. LR-ESI-MS of **2**/**3**.	**S17**
**16**	Figure S8. HR-ESI-MS of **2**/**3**.	**S18**
**17**	Figure S9. ^{1}H NMR spectrum of **2**/**3** in DMSO-d_6 at 500 MHz.	**S19**
**18**	Figure S10. ^{13}C NMR spectrum of **2**/**3** in DMSO-d_6 at 125 MHz.	**S20**
**19**	Figure S11. ^{1}H–^{1}H COSY spectrum of **2**/**3** in DMSO-d_6 at 500 MHz.	**S21**
**20**	Figure S12. HMBC spectrum of **2**/**3** in DMSO-d_6 at 500 MHz.	**S22**
**21**	Figure S13. HSQC spectrum of **2**/**3** in DMSO-d_6 at 500 MHz.	**S23**
**22**	Table S8. ^{1}H and ^{13}C NMR data of **2**/**3** and xylaropyrones B/C.	**S24**
**23**	Figure S14. LR-ESI-MS of **4**.	**S25**
**24**	Figure S15. HR-ESI-MS of **4**.	**S26**
**25**	Figure S16. ^{1}H NMR spectrum of **4** in DMSO-d_6 at 500 MHz.	**S27**
**26**	Figure S17. ^{1}H–^{1}H COSY spectrum of **4** in DMSO-d_6 at 500 MHz.	**S28**
**27**	Figure S18. HMBC spectrum of **4** in DMSO-d_6 at 500 MHz.	**S29**
**28**	Figure S19. HSQC spectrum of **4** in DMSO-d_6 at 500 MHz.	**S30**
**29**	Table S9. ^{1}H and ^{13}C NMR data of **4** and akolitserin.	**S31**
**30**	Figure S20. HR-ESI-MS of **5**.	**S32**
**31**	Figure S21. ^{1}H NMR spectrum of **5** in DMSO-d_6 at 500 MHz.	**S33**
**32**	Figure S22. ^{13}C NMR spectrum of **5** in DMSO-d_6 at 125 MHz.	**S34**
**33**	Figure S23. ^{1}H–^{1}H COSY spectrum of **5** in DMSO-d_6 at 500 MHz.	**S35**
**34**	Figure S24. HMBC spectrum of **5** in DMSO-d_6 at 500 MHz.	**S36**
**35**	Figure S25. HSQC spectrum of **5** in DMSO-d_6 at 500 MHz.	**S37**
**36**	Figure S26. ROESY spectrum of **5** in DMSO-d_6 at 500 MHz.	**S38**
**37**	Table S10. ^{1}H and ^{13}C NMR data of **5** and hypoxylin A.	**S39**
**38**	Figure S27. LR-ESI-MS of **6**.	**S40**
**39**	Figure S28. HR-ESI-MS of **6**.	**S41**
**40**	Figure S29. ^{1}H NMR spectrum of **6** in DMSO-d_6 at 500 MHz.	**S42**
**41**	Figure S30. ^{1}H–^{1}H COSY spectrum of **6** in DMSO-d_6 at 500 MHz.	**S43**
**42**	Table S11. ^{1}H and ^{13}C NMR data of **6** and (-)-(*R*)-5-(methoxycarbonyl)mellein.	**S44**
**43**	Figure S31. LR-ESI-MS of **7**.	**S45**
**44**	Figure S32. HR-ESI-MS of **7**.	**S46**
**45**	Figure S33. ^{1}H NMR spectrum of **7** in DMSO-d_6 at 500 MHz.	**S47**
**46**	Figure S34. ^{1}H–^{1}H COSY spectrum of **7** in DMSO-d_6 at 500 MHz.	**S48**
**47**	Figure S35. HMBC spectrum of **7** in DMSO-d_6 at 500 MHz.	**S49**

**48**	Figure S36. HSQC spectrum of **7** in DMSO-d_6 at 500 MHz.	**S50**
**49**	Table S12. ^{1}H and ^{13}C NMR data of compound **7** and 2-hexylidene-3-methyl succinic acid.	**S51**
**50**	Figure S37. LR-ESI-MS of **8**.	**S52**
**51**	Figure S38. HR-ESI-MS of **8**.	**S53**
**52**	Figure S39. ^{1}H NMR spectrum of **8** in DMSO-d_6 at 500 MHz.	**S54**
**53**	Figure S40. HMBC spectrum of **8** in DMSO-d_6 at 500 MHz.	**S55**
**54**	Figure S41. HSQC spectrum of **8** in DMSO-d_6 at 500 MHz.	**S56**
**55**	Table S13. ^{1}H and ^{13}C NMR data of compound **8** and 2-hexylidene-3-methyl succinic acid methyl ester.	**S57**
**56**	Figure S42. LR-ESI-MS of **9**.	**S58**
**57**	Figure S43. HR-ESI-MS of **9**.	**S59**
**58**	Figure S44. ^{1}H NMR spectrum of **9** in DMSO-d_6 at 500 MHz.	**S60**
**59**	Figure S45. HMBC spectrum of **9** in DMSO-d_6 at 500 MHz.	**S61**
**60**	Figure S46. HSQC spectrum of **9** in DMSO-d_6 at 500 MHz.	**S62**
**61**	Table S14. ^{1}H and ^{13}C NMR data of compound **9** and akoenic acid.	**S63**
**62**	Figure S47. LR-ESI-MS of **10**.	**S64**
**63**	Figure S48. HR-ESI-MS of **10**.	**S65**
**64**	Figure S49. ^{1}H NMR spectrum of **10** in DMSO-d_6 at 500 MHz.	**S66**
**65**	Figure S50. ^{1}H–^{1}H COSY spectrum of **10** in DMSO-d_6 at 500 MHz.	**S67**
**66**	Figure S51. HMBC spectrum of **10** in DMSO-d_6 at 500 MHz.	**S68**
**67**	Figure S52. HSQC spectrum of **10** in DMSO-d_6 at 500 MHz.	**S69**
**68**	Figure S53. ROESY spectrum of **10** in DMSO-d_6 at 500 MHz.	**S70**
**69**	Table S15. ^{1}H and ^{13}C NMR data of compound **10** and cytochalasin D.	**S71**
**70**	Figure S54. LR-ESI-MS of **11**.	**S72**
**71**	Figure S55. HR-ESI-MS of **11**.	**S73**
**72**	Figure S56. ^{1}H NMR spectrum of **11** in chloroform-*d* at 500 MHz.	**S74**
**73**	Figure S57. ^{13}C NMR spectrum of **11** in chloroform-*d* at 125 MHz.	**S75**
**74**	Figure S58. ^{1}H–^{1}H COSY spectrum of **11** in chloroform-*d* at 500 MHz.	**S76**
**75**	Figure S59. HMBC spectrum of **11** in chloroform-*d* at 500 MHz.	**S77**
**76**	Figure S60. HSQC spectrum of **11** in chloroform-*d* at 500 MHz.	**S78**
**77**	Figure S61. ROESY spectrum of **11** in chloroform-*d* at 500 MHz.	**S79**
**78**	Table S16. ^{1}H and ^{13}C NMR data of compound **11** and 13,14-epoxycytochalasin D.	**S80**
**79**	Figure S62. LR-ESI-MS of **12**.	**S81**
**80**	Figure S63. HR-ESI-MS of **12**.	**S82**
**81**	Figure S64. ^{1}H NMR spectrum of **12** in chloroform-*d* at 700 MHz.	**S83**
**82**	Figure S65. ^{1}H–^{1}H COSY spectrum of **12** in chloroform-*d* at 700 MHz.	**S84**
**83**	Figure S66. HMBC spectrum of **12** in chloroform-*d* at 700 MHz.	**S85**
**84**	Figure S67. HSQC spectrum of **12** in chloroform-*d* at 700 MHz.	**S86**
**85**	Table S17. ^{1}H and ^{13}C NMR data of compound **12**, 6,12:13,14-diepoxy- and 6,12-epoxycytochalasins D.	**S87**
**86**	Figure S68. LR-ESI-MS of **13**.	**S88**
**87**	Figure S69. HR-ESI-MS of **13**.	**S89**
**88**	Figure S70. ^{1}H NMR spectrum of **13** in chloroform-*d* at 700 MHz.	**S90**
**89**	Figure S71. ^{1}H–^{1}H COSY spectrum of **13** in chloroform-*d* at 700 MHz.	**S91**
**90**	Figure S72. HMBC spectrum of **13** in chloroform-*d* at 700 MHz.	**S92**
**91**	Figure S73. HSQC spectrum of **13** in chloroform-*d* at 700 MHz.	**S93**
**92**	Figure S74. ROESY spectrum of **13** in chloroform-*d* at 700 MHz.	**S94**
**93**	Table S18. ^{1}H and ^{13}C NMR data of compound **13** and 19,20-epoxycytochalasin D.	**S95**
**94**	Figure S75. LR-ESI-MS of **14**.	**S96**

**95**	Figure S76. HR-ESI-MS of **14**.	**S97**
**96**	Figure S77. ^{1}H NMR spectrum of **14** in chloroform-*d* at 500 MHz.	**S98**
**97**	Figure S78. ^{13}C NMR spectrum of **14** in chloroform-*d* at 125 MHz.	**S99**
**98**	Figure S79. ^{1}H–^{1}H COSY spectrum of **14** in chloroform-*d* at 500 MHz.	**S100**
**99**	Figure S80. HMBC spectrum of **14** in chloroform-*d* at 500 MHz.	**S101**
**100**	Figure S81. HSQC spectrum of **14** in chloroform-*d* at 500 MHz.	**S102**
**102**	Figure S82. ROESY spectrum of **14** in chloroform-*d* at 500 MHz.	**S103**
**103**	Table S19. ^{1}H and ^{13}C NMR data of compound **14** and cytochalasin R.	**S104**
**104**	Table S20. Antimicrobial Activity Results.	**S105**
**105**	Table S21. *Tub2* and *rpb2* sequences of *Xylaria* sp. CM-UDEA-H199.	**S106**
**106**	Figure S83. Base peak chromatogram (BPC) from the crude extract obtained after cultivation in S-BRFT and extracted ion chromatograms (XIC) of isobaric cytochalasins.	**S107**
**107**	Metabolomics Procedures	**S108**

PURIFICATION SCHEMES.

A. Purification of compounds from BRFT culture

Compounds (**1**–**6**) were isolated from the defatted MeOH fraction derived from the extraction of solid-state culture in BRFT medium as described in Table S1. By using the gradient elution (G1), 150 mg of crude extract were purified, resulting in eleven fractions, including compounds **1**–**3** and **6**. On the other hand, 3 × 100 mg of total extract were purified using the gradient method (G2) yielding twelve fractions afforded compounds (**4** and **5**).

Table S1. Purification method for BRFT cultures.

<table>
<tr><td>Purified extract</td><td colspan="5">Defatted MeOH fraction of BRFT total extract</td></tr>
<tr><td>Chromatography equipment</td><td colspan="5">Reversed phase separation using a PLC 2250 preparative HPLC system (Gilson, Middleton, WI, USA)</td></tr>
<tr><td>Stationary pase</td><td colspan="5">Column: Luna C_{18} (250 × 50 mm, 10 µm; Phenomenex, Torrance, CA, USA)</td></tr>
<tr><td>Mobile pase</td><td colspan="5">Solvents:
A: Deionized water + 0.1% formic acid (FA)
B: Acetonitrile (MeCN) + 0.1% formic acid (FA)</td></tr>
<tr><td>Flow rate</td><td colspan="5">30 mL/min</td></tr>
<tr><td>Collection volume</td><td colspan="5">15 mL/tube</td></tr>
<tr><td>Details of the sample injection and amount separated</td><td colspan="5">The total defatted MeOH fraction was dissolved in Acetone:MeOH (1:3, v/v) and then centrifuged for 4 min at 4,000 rpm. The supernatant was separated for further purification.</td></tr>
<tr><td>UV Detection</td><td colspan="5">Channels: 220, 225, 278 and 312 nm
Scan: 200–600 nm</td></tr>
<tr><td rowspan="24">Gradient method(s) and the isolated compounds</td><td rowspan="7">Gradient (G1)</td><td colspan="2">Time (min)</td><td colspan="2">%B</td></tr>
<tr><td colspan="2">0:00</td><td colspan="2">5</td></tr>
<tr><td colspan="2">5:00</td><td colspan="2">5</td></tr>
<tr><td colspan="2">15:00</td><td colspan="2">25</td></tr>
<tr><td colspan="2">1:25:00</td><td colspan="2">95</td></tr>
<tr><td colspan="2">1:30:00</td><td colspan="2">100</td></tr>
<tr><td colspan="2">1:40:00</td><td colspan="2">100</td></tr>
<tr><td rowspan="5">Compounds</td><td>Compound</td><td colspan="2">Retention time (min)</td><td>Yield (mg)*</td></tr>
<tr><td>1</td><td colspan="2">47:39-48:14</td><td>1.3</td></tr>
<tr><td>2 and 3</td><td colspan="2">23:07-23:43</td><td>3.0</td></tr>
<tr><td>6</td><td colspan="2">46:11-47:05</td><td>1.4</td></tr>
<tr><td colspan="4">*The yield is reported in mg of the compound obtained from 150 mg of total fraction.</td></tr>
<tr><td rowspan="8">Gradient (G2)</td><td colspan="2">Time (min)</td><td colspan="2">%B</td></tr>
<tr><td colspan="2">0:00</td><td colspan="2">20</td></tr>
<tr><td colspan="2">5:00</td><td colspan="2">20</td></tr>
<tr><td colspan="2">46:36</td><td colspan="2">94</td></tr>
<tr><td colspan="2">50:00</td><td colspan="2">100</td></tr>
<tr><td colspan="2">1:00:00</td><td colspan="2">100</td></tr>
<tr><td colspan="2">1:05:00</td><td colspan="2">100</td></tr>
<tr><td colspan="2">1:10:00</td><td colspan="2">100</td></tr>
<tr><td rowspan="4">Compounds</td><td>Compound</td><td colspan="2">Retention time (min)</td><td>Yield (mg)*</td></tr>
<tr><td>4</td><td colspan="2">25:58–26:46</td><td>14.0</td></tr>
<tr><td>5</td><td colspan="2">30:59–31:47</td><td>12.0</td></tr>
<tr><td colspan="4">*The yield is reported in mg of the compound obtained from 300 mg of crude extract.</td></tr>
</table>

S1.2. Purification of compounds from YM culture

Compounds (**7**–**9**) were isolated from the supernatant extract of the YM cultures as shown in Table S2. Twelve fractions were obtained from the purification of 6 × 156 mg of the total extract applying the gradient (G3) described below in Table S2. The first and second fractions were combined, yielding compound **7**. Compounds **8** and **9** were also obtained from this separation.

Table S2. Purification method for YM cultures.

<table>
<tr><td>Purified extract</td><td colspan="4">YM supernatant</td></tr>
<tr><td>Chromatography equipment</td><td colspan="4">Reversed phase separation using a PLC 2250 preparative HPLC system (Gilson, Middleton, WI, USA).</td></tr>
<tr><td>Stationary pase</td><td colspan="4">Column: Luna C_{18} (250 × 50 mm, 10µm; Phenomenex, Torrance, CA, USA)</td></tr>
<tr><td>Mobile phase</td><td colspan="4">Solvents:
A: Deionized water + 0.1% formic acid (FA)
B: MeCN + 0.1% formic acid (FA)</td></tr>
<tr><td>Flow rate</td><td colspan="4">30 mL/min</td></tr>
<tr><td>Collection volume</td><td colspan="4">15 mL/tube</td></tr>
<tr><td>Details of the sample injection and amount separated</td><td colspan="4">The total extract was dissolved in Acetone:MeOH (1:1, v/v).</td></tr>
<tr><td>UV detection</td><td colspan="4">Channels: 200, 220, 280, 330 nm
Scan: 200–600 nm</td></tr>
<tr><td rowspan="12">Gradient method(s) and the isolated compounds</td><td rowspan="7">Gradient (G3)</td><td colspan="2">Time (min)</td><td>%B</td></tr>
<tr><td colspan="2">0:00</td><td>35</td></tr>
<tr><td colspan="2">5:00</td><td>35</td></tr>
<tr><td colspan="2">10:00</td><td>45</td></tr>
<tr><td colspan="2">40:00</td><td>75</td></tr>
<tr><td colspan="2">50:00</td><td>100</td></tr>
<tr><td colspan="2">60:00</td><td>100</td></tr>
<tr><td rowspan="5">Compounds</td><td>Compound</td><td>Retention time (min)</td><td>Yield (mg)*</td></tr>
<tr><td>7</td><td>32:30–33:30</td><td>20.0</td></tr>
<tr><td>8</td><td>43:00–43:30</td><td>1.7</td></tr>
<tr><td>9</td><td>55:00–55:30</td><td>1.4</td></tr>
<tr><td colspan="3">*The yield is reported in mg of the compound obtained from 156 mg of crude extract</td></tr>
</table>

S1.3. Purification of compounds from S-BRFT culture

Compounds **10**–**14** were purified from the defatted MeOH fraction of the S-BRFT total extract, for this, three separations were performed consecutively. First, the defatted MeOH fraction (1,588 mg) was pre-separated using flash chromatography according to Table S3 and applying the gradient G4 (Table S3). Thus, twelve fractions (F1–F12) were obtained, and the first three (F1–F3) were combined for further purification as they presented similar LC-MS profiles yielding a total weight of 648 mg. The combined fraction was separated by reversed phase HPLC using two different methods as described in Table S4. With the gradient G5 (Table S4) applied on 96 mg, fourteen subfractions (SF1.1–SF1.14) were obtained including compound **10**. On the other hand, with the gradient G5 (Table S4) 6 × 96 mg of the extract were purified resulting in twelve subfractions (SF2.1–SF2.12) with no pure compounds among them. Then, SF2.1–SF2.12 were sorted into four pooled fractions (PSF2.A-PSF2.D) based on their LC-MS profiles for targeting purifications of compounds (**11**–**14**).

The first pooled fraction (PSF2.A, 53.6 mg) yielded compound **12** as described in Table S5, by applying gradient method G6 (Table S5). The second pooled fraction (PSF2.B, 97.4 mg) yielded compound **14** and

was purified with 9 × 10 mg as described in Table S5, using gradient method G7 (Table S5). The third pooled fraction (PSF2.C, 22 mg) yielded compound **11** and was purified with 2 × 10 mg as described in Table S5, using gradient G8 (Table S5). The fourth pooled fraction (PSF2.D, 4.5 mg) yielded compound **13** and was purified using semipreparative HPLC as described in Table S6 using gradient G9 (Table S6).

Table S3. Pre-purification method for S-BRFT cultures.

Purified fraction	S-BRFT MeOH		
Cromatography equipment	Flash chromatography (Grace Reveleris®, Columbia, MD, USA)		
Stationary pase	To prepare the column, the crude extract was dissolved in MeOH, mixed with silica gel, dried in vacuum at 40 ºC using a rotary evaporator and placed in a silica cartridge of 40 g.		
Mobile phase	Solvents:<br>A: Dichloromethane (DCM)<br>B: MeOH (MeOH)		
Flow rate	30 mL/min		
Collection volume	15 mL		
Details of the sample injection and amount separated	N/A		
UV detection	Channels: 210 nm, 254 nm, 350 nm		
Gradient method	G4	**Time (min)**	**%B**
		0:00	0
		5:00	0
		1:45:00	100
		1:50:00	100

Table S4. Purification method for fractions obtained from flash chromatography (MeOH_S-BRFT).

Purified fraction	Mixture of fractions 1, 2 and 3 obtained from flash chromatography (S-BRFT media cultures - MeOH fraction).		
Cromatography equipment	Reverse phase separation using a PLC 2250 preparative HPLC system (Gilson, Middleton, WI, USA)		
Stationary pase	Column: Gemini C_{18} (250 × 50 mm, 10 µm; Phenomenex®, Torrance, CA, USA)		
Mobile pase	Solvents:<br>A: Deionized water + 0.1% formic acid (FA)<br>B: MeCN + 0.1% formic acid (FA)		
Flow rate	30 mL/min		
Collection volume	15 mL/tube		
Details of the sample injection and amount separated	The crude extract was dissolved in Acetone:MeOH (1:3, v/v) and then centrifuged for 4 min at 4000 rpm. The supernatant was purified, and the precipitant was discarded. On each run approximately 100 mg were purified.		
UV Detection	Channels: 220, 225, 278 and 312 nm<br>Scan: 200–600 nm		
Gradient method(s) and isolated compound	G5	**Time (min)**	**%B**
		0:00	20
		5:00	20
		15:00	40
		1:15:00	100
		1:25:00	100

<table>
<tr><td rowspan="10"></td><td rowspan="3">Compounds</td><td>Compound</td><td colspan="2">Retention time (min)</td><td>Yield (mg)*</td></tr>
<tr><td>10</td><td colspan="2">42:46–43.09</td><td>10</td></tr>
<tr><td colspan="4">*The yield is reported in mg of the compound obtained from 90 mg of the purified fraction</td></tr>
<tr><td rowspan="6">G6</td><td colspan="2">Time (min)</td><td colspan="2">%B</td></tr>
<tr><td colspan="2">0:00</td><td colspan="2">30</td></tr>
<tr><td colspan="2">5:00</td><td colspan="2">30</td></tr>
<tr><td colspan="2">15:00</td><td colspan="2">40</td></tr>
<tr><td colspan="2">1:15:00</td><td colspan="2">100</td></tr>
<tr><td colspan="2">1:25:00</td><td colspan="2">100</td></tr>
</table>

Table S5. Purification method for fractions obtained from NP-HPLC (MeOH_S-BRFT).

<table>
<tr><td>Chromatography equipment</td><td colspan="4">NP-HPLC the Agilent 1100 Serie</td></tr>
<tr><td>Stationary pase</td><td colspan="4">Column: Nucleosil 100-7 (250 × 21 mm, 7 µm; Machery-Nagel™, Düren, Germany)</td></tr>
<tr><td>Mobile pase</td><td colspan="4">Solvents
A: ter-Butyl methyl ether:n-heptane (1:1)
B: ter-Butyl methyl ether:n-heptane:MeOH (15:4:1)</td></tr>
<tr><td>Flow rate</td><td colspan="4">20 mL/min</td></tr>
<tr><td>Collection volume</td><td colspan="4">2 mL</td></tr>
<tr><td>Details of the sample injection and amount separated</td><td colspan="4">The fractions were dissolved in DCM and an aliquot of 10 mg was injected for each run.</td></tr>
<tr><td>UV Detection</td><td colspan="4">Channels: 215, 220, 250 and 285 nm</td></tr>
<tr><td rowspan="23">Gradient method(s) and isolated compound</td><td rowspan="6">G7</td><td colspan="2">Time (min)</td><td>%B</td></tr>
<tr><td colspan="2">0:00</td><td>0</td></tr>
<tr><td colspan="2">1:00</td><td>0</td></tr>
<tr><td colspan="2">3:00</td><td>15</td></tr>
<tr><td colspan="2">40:00</td><td>15</td></tr>
<tr><td colspan="2">40:10</td><td>0</td></tr>
<tr><td rowspan="4">Compounds</td><td>Compound</td><td>Retention time (min)</td><td>Yield (mg)*</td></tr>
<tr><td>11</td><td>25.6702–27.3267</td><td>3.68</td></tr>
<tr><td>12</td><td>37.9975–40.0013</td><td>0.89</td></tr>
<tr><td colspan="3">*The yield is reported in mg of the compound obtained from 20 mg of the purified fraction.</td></tr>
<tr><td rowspan="6">G8</td><td colspan="2">Time (min)</td><td>%B</td></tr>
<tr><td colspan="2">0:00</td><td>0</td></tr>
<tr><td colspan="2">1:00</td><td>0</td></tr>
<tr><td colspan="2">3:00</td><td>10</td></tr>
<tr><td colspan="2">40:00</td><td>10</td></tr>
<tr><td colspan="2">40:10</td><td>0</td></tr>
<tr><td rowspan="3">Compound</td><td>Compound</td><td>Retention time (min)</td><td>Yield (mg)*</td></tr>
<tr><td>14</td><td>23:26–23:50</td><td>1.4</td></tr>
<tr><td colspan="3">*The yield is reported in mg of the compound obtained from 10 mg of the purified fraction.</td></tr>
</table>

Table S6. Purification method for PSF2.A obtained from RP-HPLC (MeOH_S-BRFT).

<table>
<tr><td>Purified fraction</td><td colspan="4">PSF2.A</td></tr>
<tr><td>Chromatography equipment</td><td colspan="4">Agilent Technologies 1200 Infinity Series, semipreparative HPLC (Waldbronn, Germany)</td></tr>
<tr><td>Stationary pase</td><td colspan="4">Column: XBridge BEH C18 (250 mm × 10 mm, 5 µm; Waters, Eschborn, Germany)</td></tr>
<tr><td>Mobile pase</td><td colspan="4">Solvents:
A: Deionized water + 0.1% formic acid (FA)
B: MeCN + 0.1% formic acid (FA)</td></tr>
<tr><td>Flow rate</td><td colspan="4">3.5 mL/min</td></tr>
<tr><td>Collection volume</td><td colspan="4">0.2 mL</td></tr>
<tr><td>Details of the sample injection and amount separated</td><td colspan="4">The total extract was dissolved in 200 µL Acetone:MeOH (1:1, v/v). The injection in one run was of 100 µL.</td></tr>
<tr><td>UV Detection</td><td colspan="4">Channels: 210, 280, 300 and 360 nm.</td></tr>
<tr><td rowspan="9">Gradient method(s) and isolated compound</td><td rowspan="6">G9</td><td colspan="2">Time (min)</td><td>%B</td></tr>
<tr><td colspan="2">0</td><td>15</td></tr>
<tr><td colspan="2">3</td><td>15</td></tr>
<tr><td colspan="2">10</td><td>35</td></tr>
<tr><td colspan="2">50</td><td>70</td></tr>
<tr><td colspan="2">55</td><td>100</td></tr>
<tr><td rowspan="3">Compound</td><td>Compound</td><td>Retention time (min)</td><td>Yield (mg)*</td></tr>
<tr><td>13</td><td>19.50–20.00</td><td>0.1</td></tr>
<tr><td colspan="3">*The yield is reported in mg of the compound obtained from 4.52 mg of the purified fraction.</td></tr>
</table>

Generic Display Report

Analysis Info

Analysis Name	S:\DATA\AmaZon\dva23_Daniela Valencia Revelo\Gymnopus montagnei\Gymnopus Rice MeOH fractions\R1F4_GA4_01_14558.d	Acquisition Date	22.06.2023 13:06:45
Method	r14558m	Operator	lab
Sample Name	R1F4	Instrument	amaZon speed
Comment			

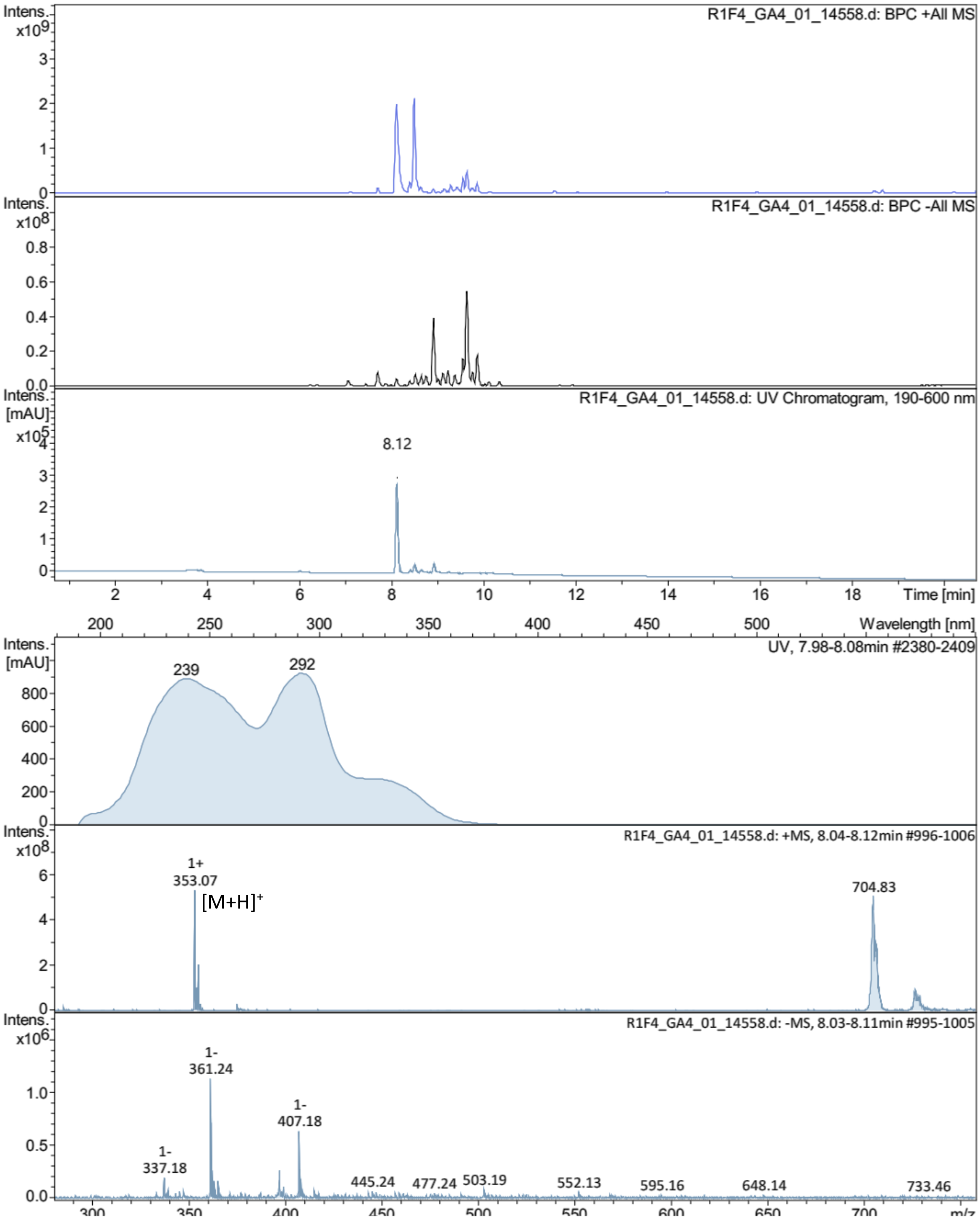

Bruker Compass DataAnalysis 4.4 | printed: 26.06.2023 15:51:30 | by: sel22 | Page 1 of 1

Figure S1. LR-ESI-MS of **1**.

Generic Display Report

Analysis Info

		Acquisition Date	22.06.2023 13:39:13
Analysis Name	S:\DATA\MaXis\dva23_Daniela Valencia Revelo\23_06\23_06_22\23_06_22\Gymnopus-Rice - MeOH_R1_F4_24_01_11900.d		
Method	pos_saure_[illegible]_screening_ms_100_2500_line.m	Operator	ate06
Sample Name	Gymnopus-Rice - MeOH_R1_F4	Instrument	maXis
Comment	Screening01<br>Waters Acquity UPLC BEH C_18 1,7um 2.1x50mm		

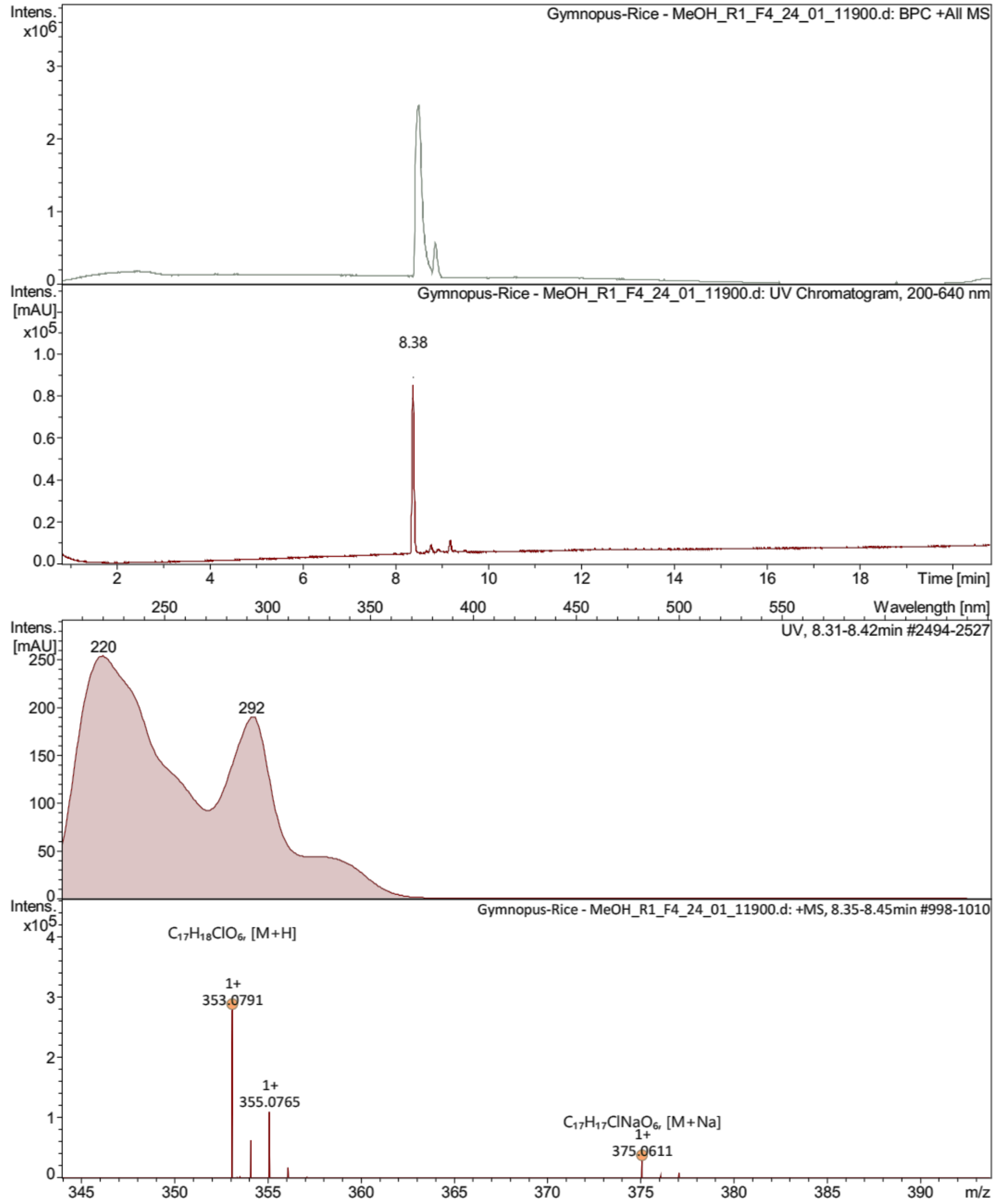

Bruker Compass DataAnalysis 4.4 printed: 26.06.2023 17:55:52 by: sel22 Page 1 of 1

Figure S2. HR-ESI-MS of **1**.

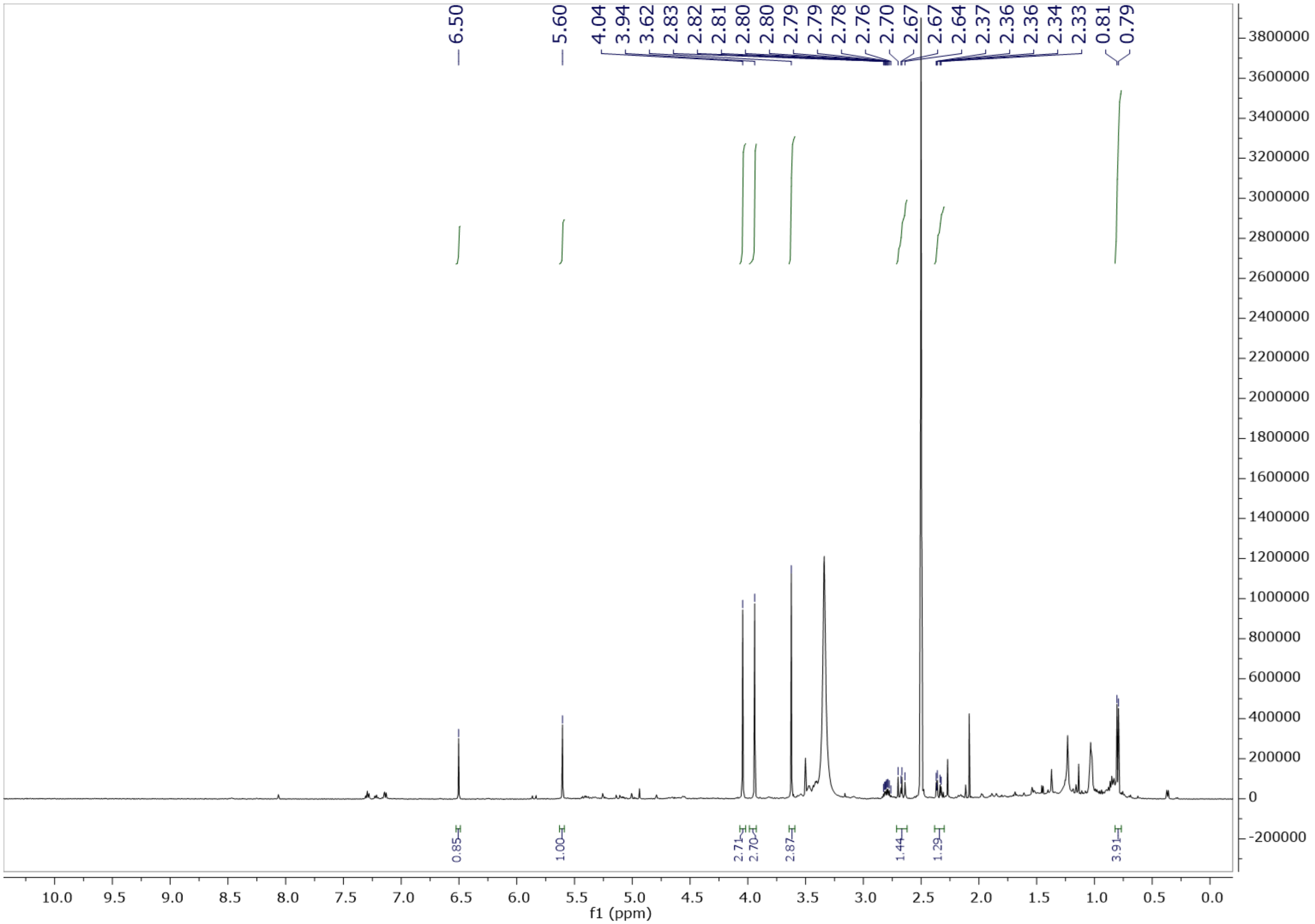

Figure S3. ^{1}H NMR spectrum of **1** in DMSO-d_6 at 500 MHz.

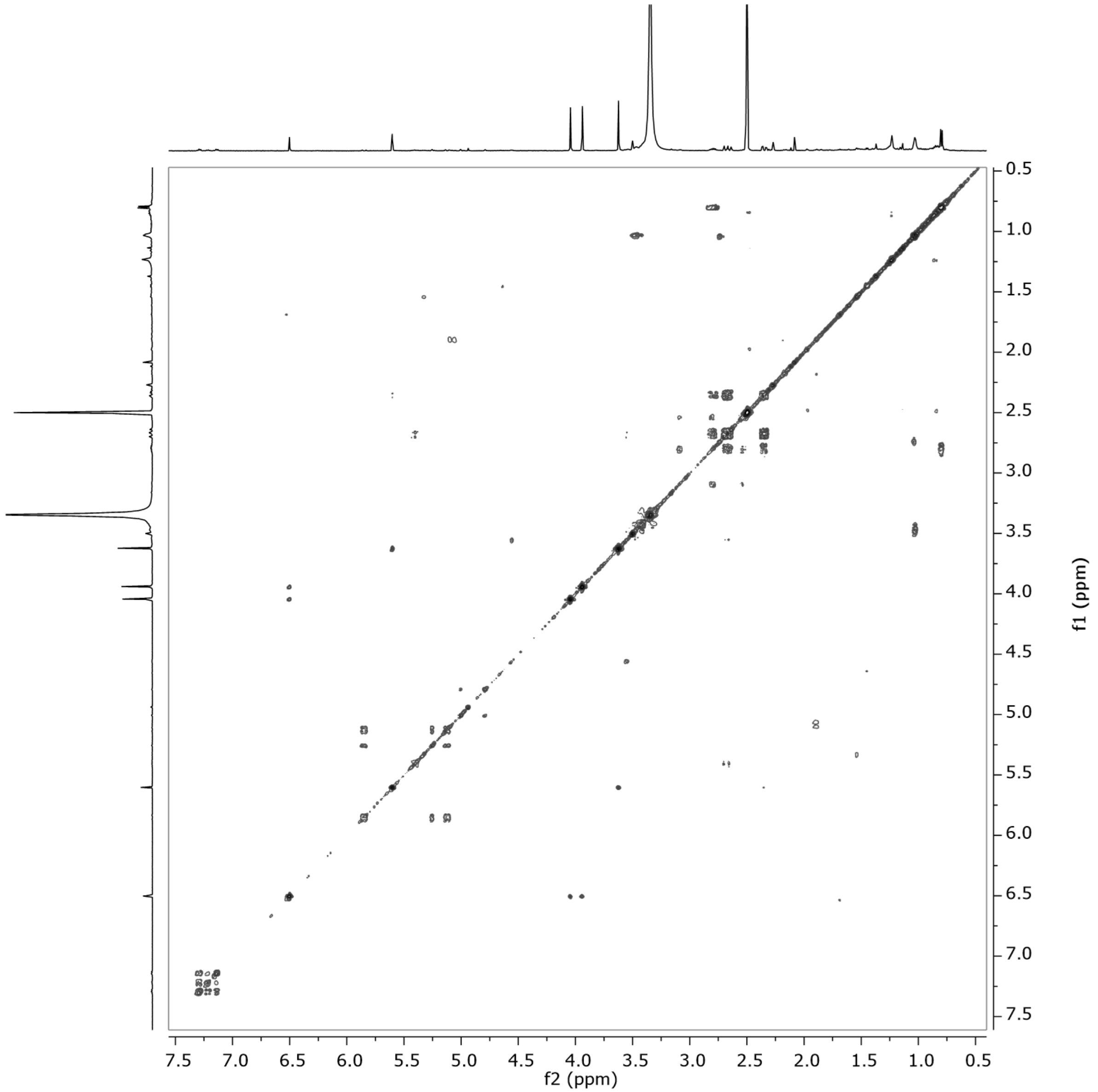

Figure S4. ^{1}H-^{1}H COSY spectrum of **1** in DMSO-d_6 at 500 MHz.

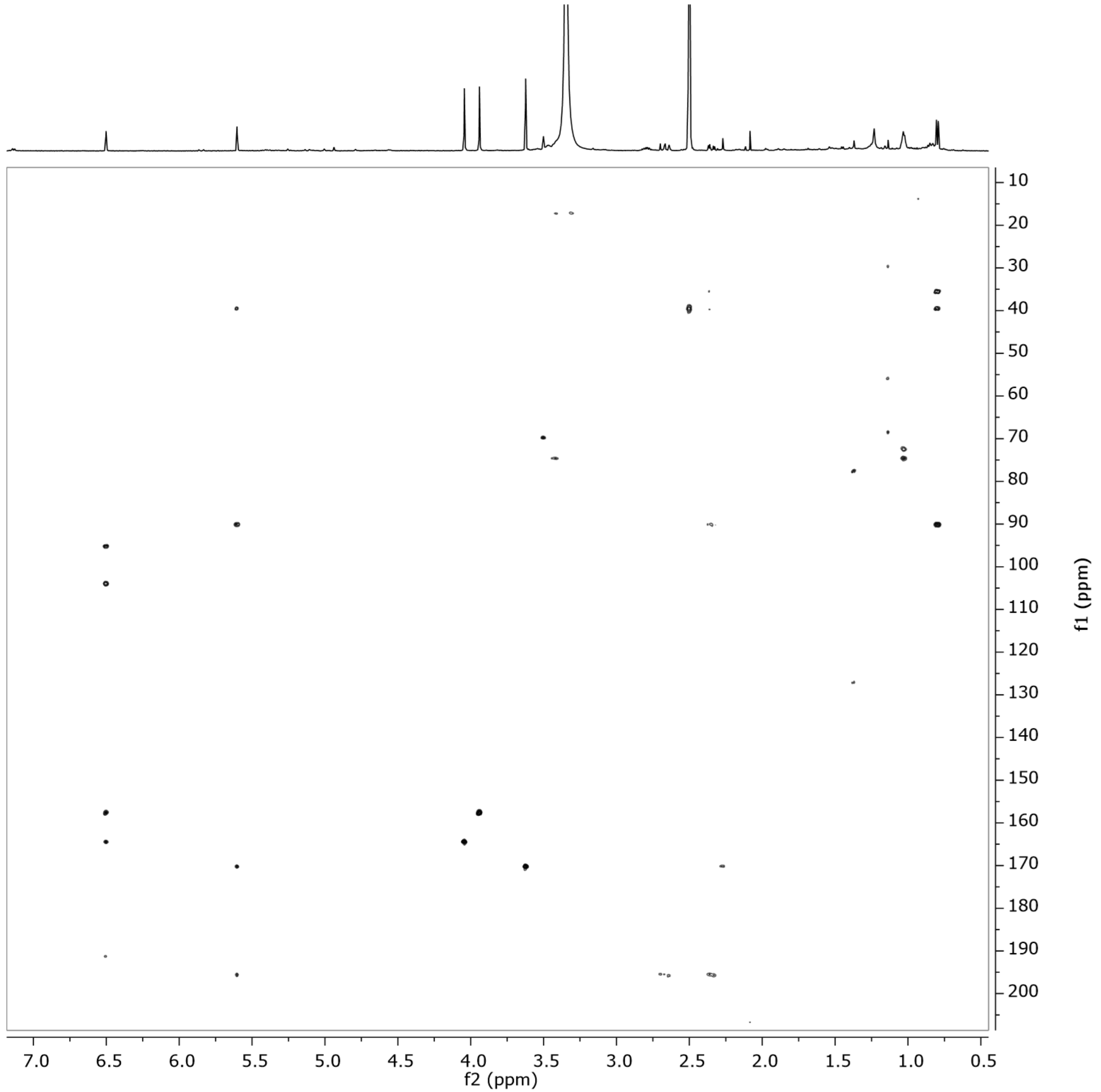

Figure S5. HMBC spectrum of **1** in DMSO-d_6 at 500 MHz.

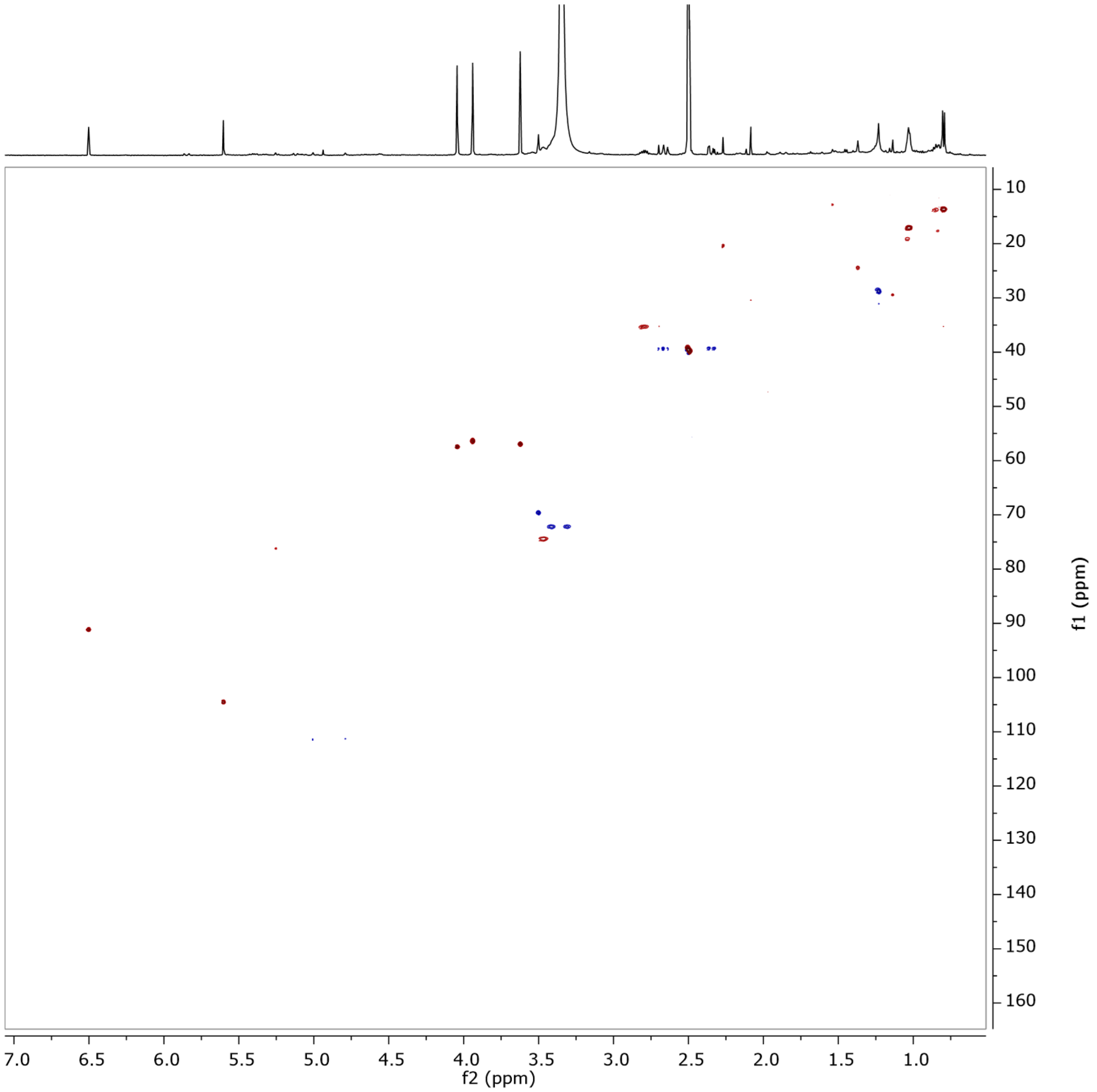

Figure S6. HSQC spectrum of **1** in DMSO-d_6 at 500 MHz.

Table S7. ^{1}H and ^{13}C NMR data of **1** and griseofulvin.

	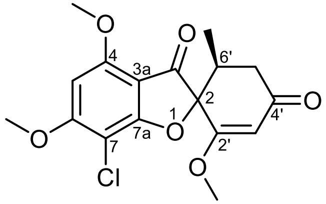<br>Griseofulvin			
	Compound **1**		Griseofulvin	
pos.	δ_C,[a,c] type	δ_H[b] multi (*J* in Hz)	δ_C,[a,c] type	δ_H[b] multi (*J* in Hz)
2	90.1, C		90.1, C	
3	191.2, CO		191.1, CO	
3a	104.0, C		104.0, C	
4	157.5, C		157.5, C	
5	91.2, CH	6.50 s	91.3, CH	6.50 s
6	164.4, C		164.4, C	
7	95.2, C		95.2, C	
7a	n.d.[c]		168.5, C	
2‘	170.2, C		170.2, C	
3‘	104.7, C	5.60 s	104.6, C	5.60 s
4‘	195.5, CO		195.4, CO	
5‘	39.4, CH_2	2.67 dd (16.6, 13.2)<br>2.35 dd (16.6, 4.7)	39.8, CH_2	2.67 dd (16.6, 13.3)<br>2.35 dd (16.6, 4.8)
6‘	35.4, CH	2.80 ddd (13.3, 6.7, 4.8)	35.5, CH	2.76–2.84 m
4-OMe	56.4, CH_3	3.94 s	56.5, CH_3	3.94 s
6-OMe	57.5, CH_3	4.04 s	57.5, CH_3	4.04 s
2‘-OMe	56.9, CH_3	3.62 s	56.9, CH_3	3.62 s
6‘-Me	13.7, CH_3	0.80 d (6.6)	13.7, CH_3	0.80 d (6.6)

Measured in DMSO-d_6 at [a] 125 and [b] 500 MHz. [c] n.d.: not detected.

Generic Display Report

Analysis Info

Analysis Name	S:\DATA\AmaZon\dva23_Daniela Valencia Revelo\Gymnopus montagnei\Gymnopus Rice MeOH fractions\R1F1_GA1_01_14555.d	Acquisition Date	22.06.2023 11:18:08
Method	14550m	Operator	lab
Sample Name	R1F1	Instrument	amaZon speed
Comment			

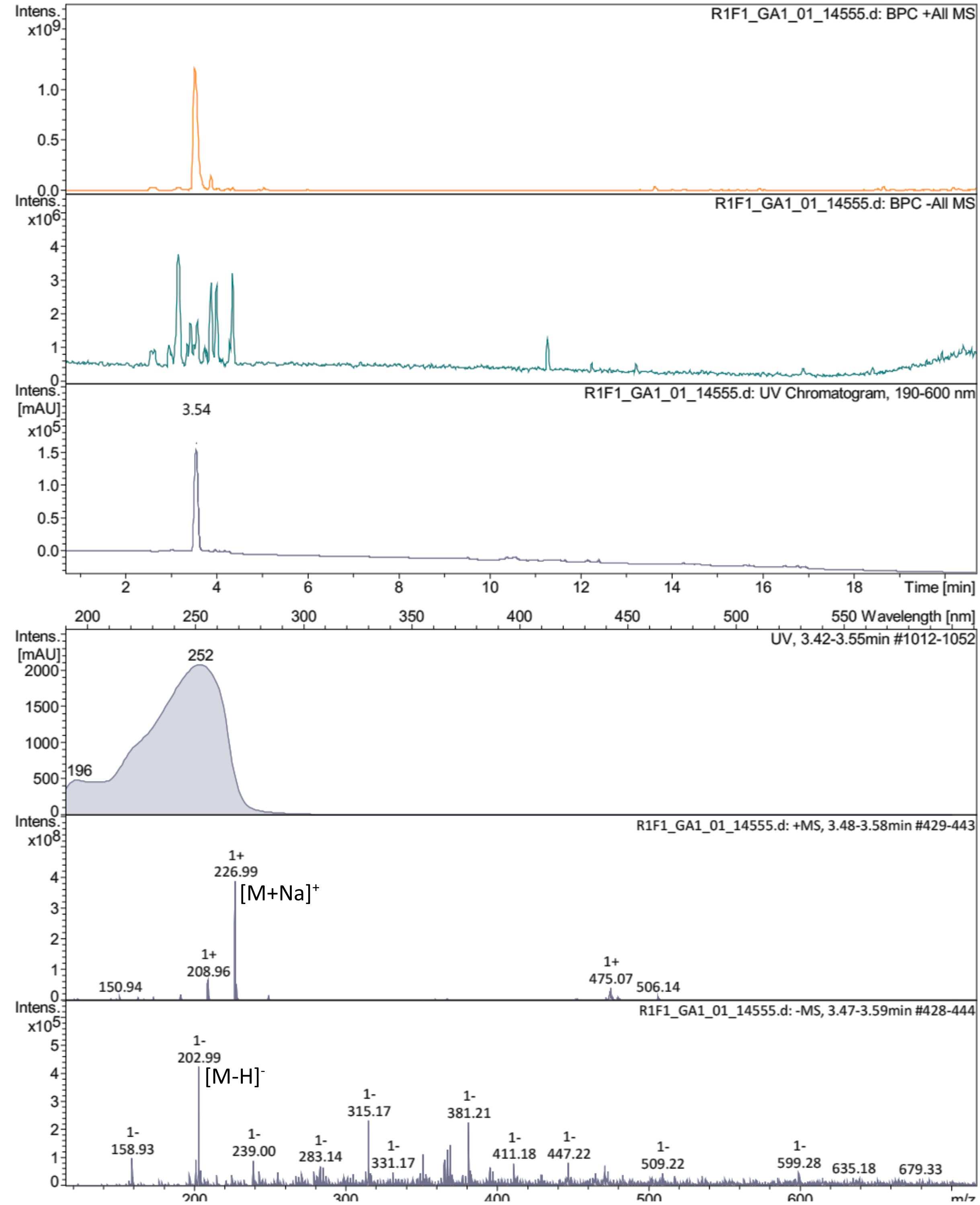

Bruker Compass DataAnalysis 4.4 printed: 26.06.2023 15:39:29 by: sel22 Page 1 of 1

Figure S7. LR-ESI-MS of **2**/**3**.

Generic Display Report

Analysis Info

Analysis Name	S:\DATA\MaXis\dva23_Daniela Valencia Revelo\23_06\23_06_22\23_06_22\Gymnopus-Rice - MeOH_R1_F1_21_01_11897.d	Acquisition Date	22.06.2023 12:06:15
Method	pos_stauro_F0020_screening_ms_100_2500_line.m	Operator	ate06
Sample Name	Gymnopus-Rice - MeOH_R1_F1	Instrument	maXis
Comment	Screening01<br>Waters Acquity UPLC BEH C_18 1,7um 2.1x50mm		

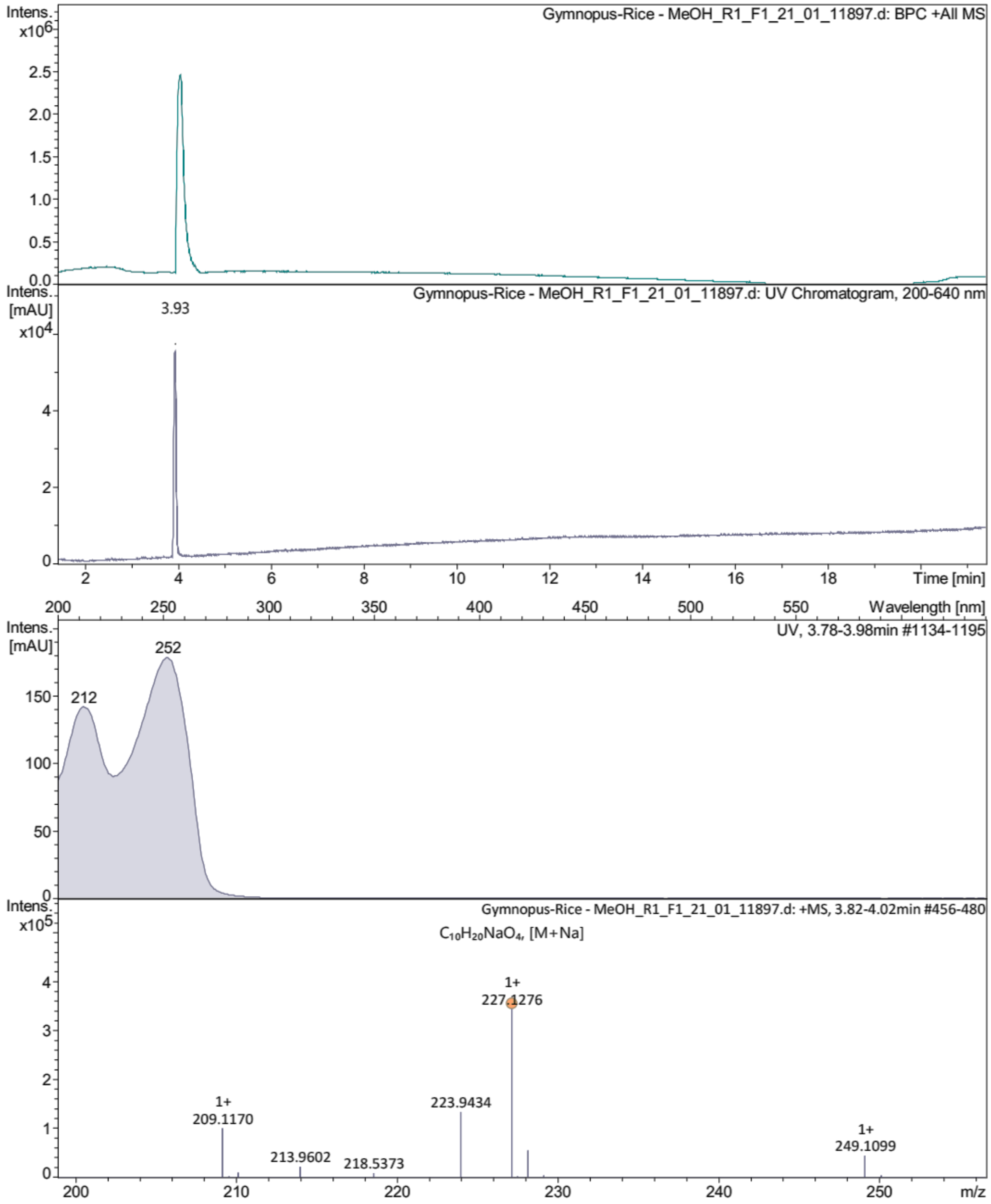

Figure S8. HR-ESI-MS of **2**/**3**.

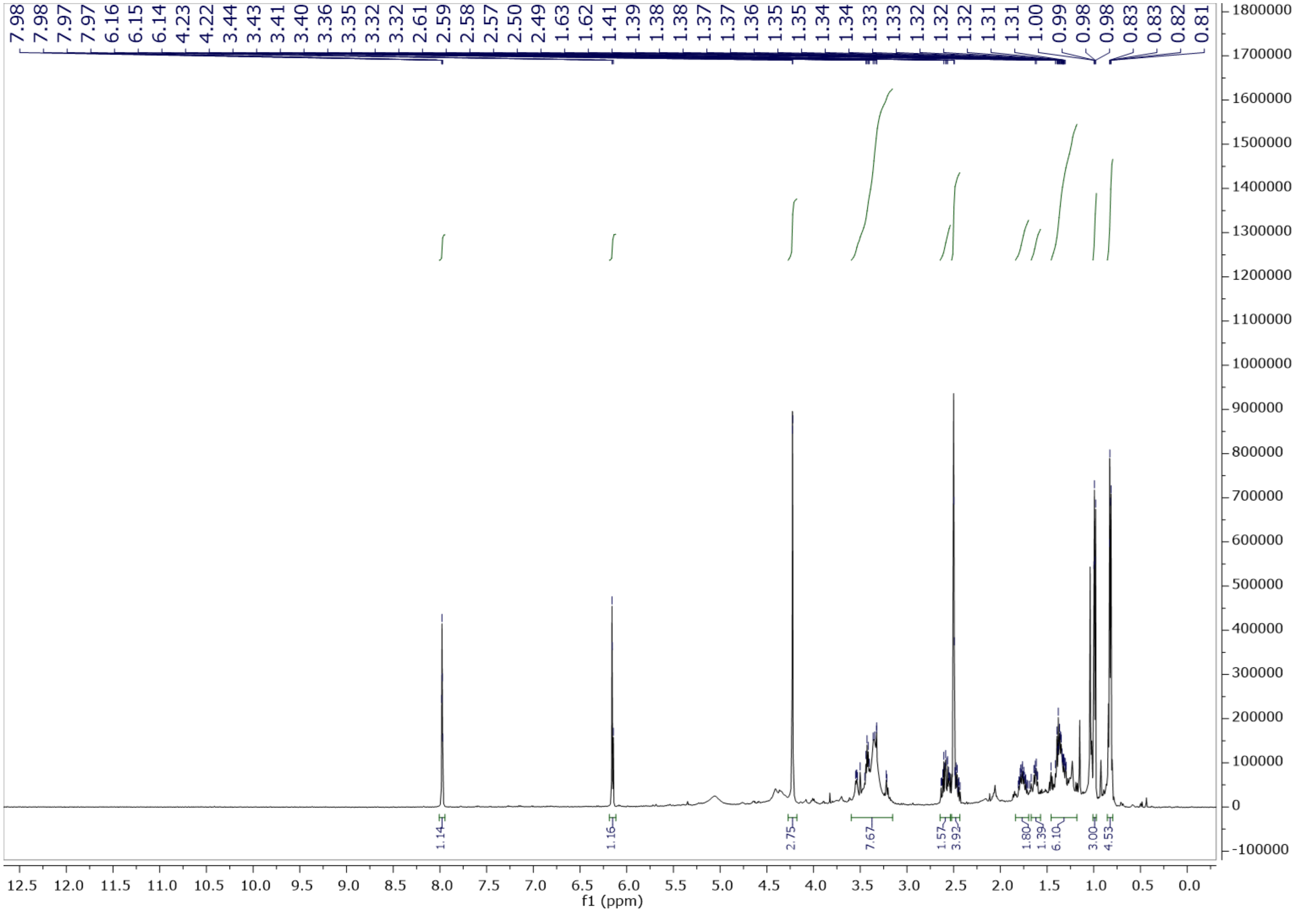

Figure S9. ^{1}H NMR spectrum of **2**/**3** in DMSO-d_6 at 500 MHz.

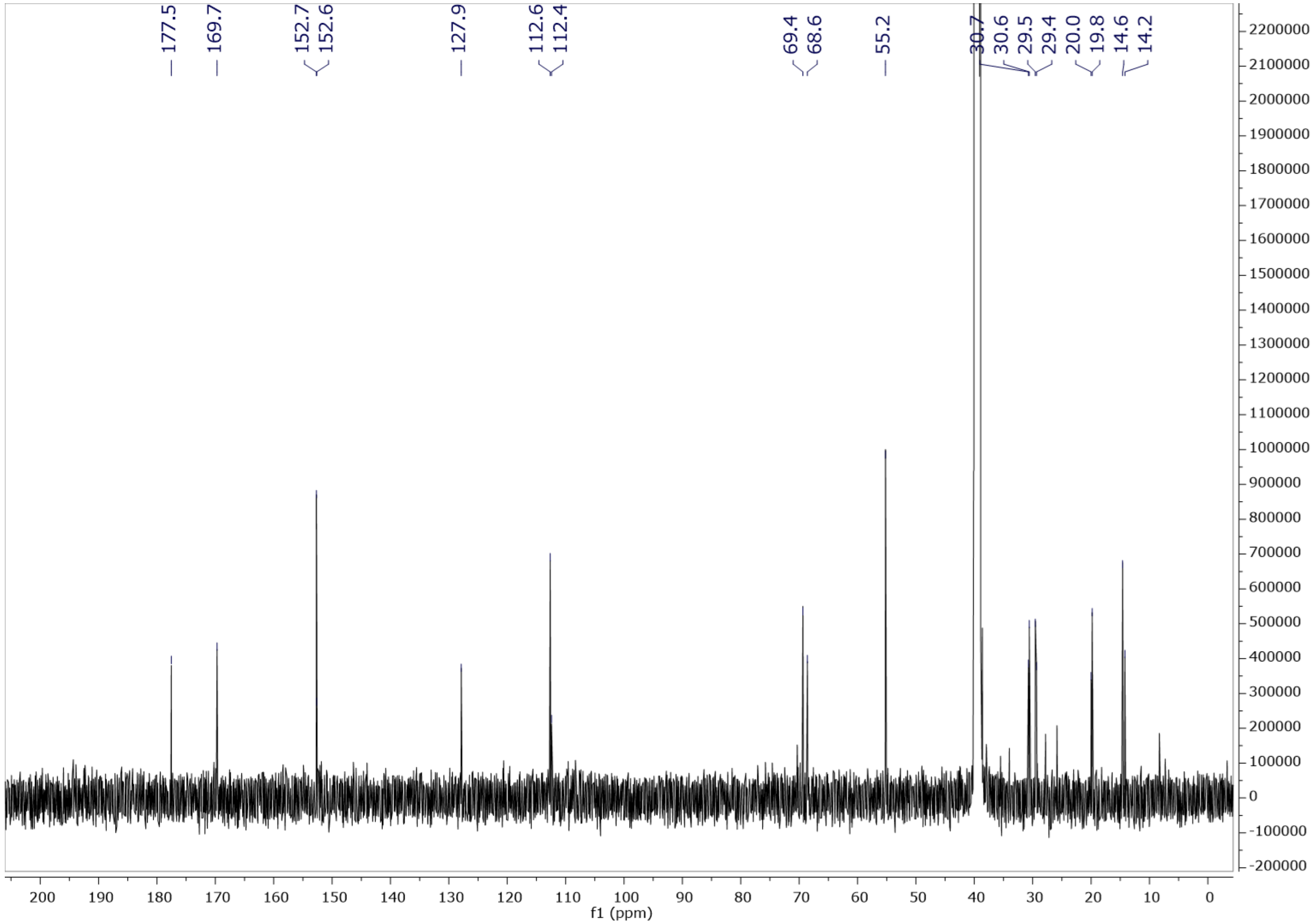

Figure S10. ^{13}C NMR spectrum of **2**/**3** in DMSO-d_6 at 125 MHz.

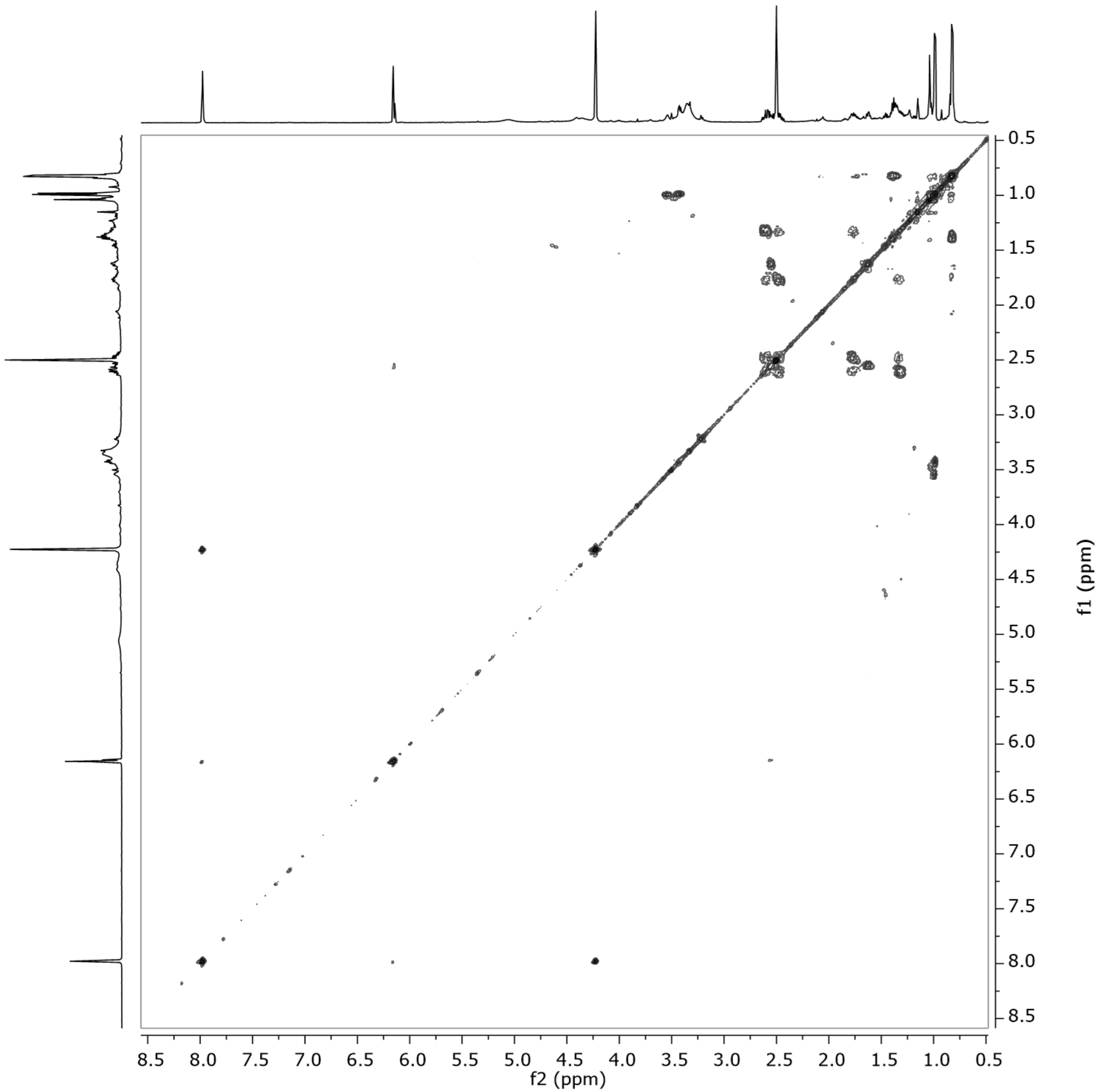

Figure S11. ^{1}H-^{1}H COSY spectrum of **2**/**3** in DMSO-d_6 at 500 MHz.

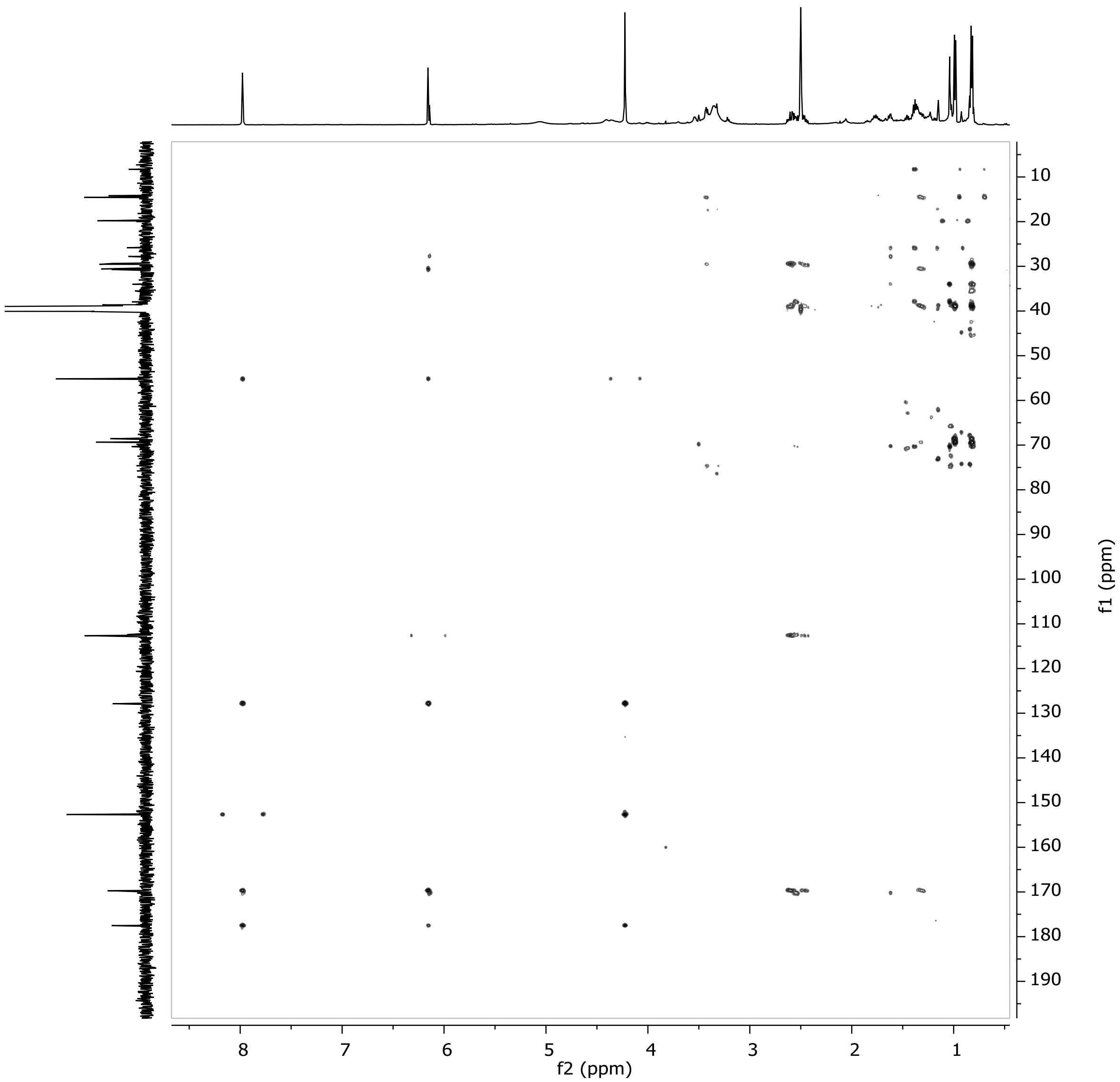

Figure S12. HMBC spectrum of **2**/**3** in DMSO-d_6 at 500 MHz.

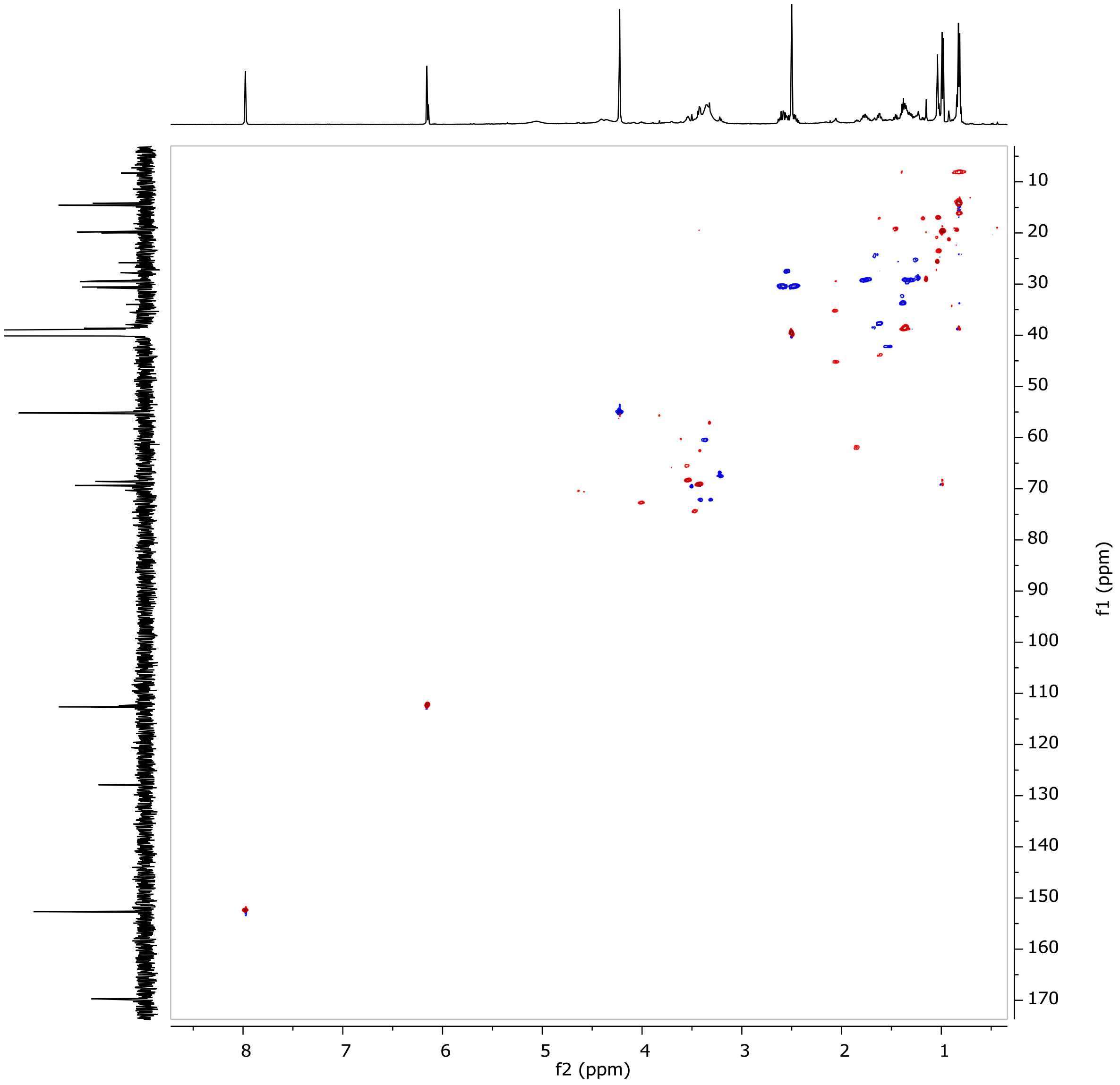

Figure S13. HSQC spectrum of **2**/**3** in DMSO-d_6 at 500 MHz.

Table S8. ^{1}H and ^{13}C NMR data of **2**/**3** and xylaropyrones B/C.

	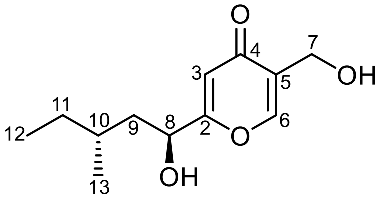<br>Xylaropyrone B				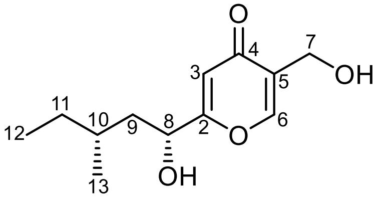<br>Xylaropyrone C			
	Compound **2**		Xylaropyrone B		Compound **3**		Xylaropyrone B	
pos.	δ_C,[a] type	δ_H[b] multi (*J* in Hz)	δ_C,[c] type	δ_H[d] multi (*J* in Hz)	δ_C,[a] type	δ_H[b] multi (*J* in Hz)	δ_C,[c] type	δ_H[d] multi (*J* in Hz)
2	169.7, C		172.4, C		169.7, C		172.1, C	
3	112.4, CH	6.14 s	111.7, CH	6.41 s	112.6, CH	6.16 s	112.0, CH	6.41 s
4	177.5, C		180.3, CO		177.5, C		180.3, CO	
5	127.9, C		127.6, C		127.9, C		127.6, C	
6	152.6, CH	7.97 s	152.5, CH	7.81 s	152.7, CH	7.98 s	152.7, CH	7.83 s
7	55.2, CH_2	4.23 s	58.0, CH_2	4.44 s	55.2, CH_2	4.22 s	57.8, CH_2	4.43 s
8	68.6, CH	3.54 dt (9.9, 5.9)	68.9, CH	4.51 dd (9.7, 3.7)	69.4, CH	3.43 p (6.3)	69.3, CH	4.50 dd (8.3, 5.3)
9	30.7, CH_2	α 2.59 m<br>β 2.48 m	42.3, CH_2	α 1.73 ddd (13.8, 9.7, 4.1)<br>β 1.48 ddd (13.8, 9.4, 3.7)	30.6, CH_2	α 2.59 m<br>β 2.47 m	42.3, CH_2	α 1.71 ddd (13.4, 8.3, 4.9)<br>β 1.58 dd (13.4, 5.3)
10	38.4, CH	1.36 m	30.7, CH	1.64 m	38.7, CH	1.37 m	30.9, CH	1.54 m
11	29.4, CH_2	α 1.75 m<br>β 1.36 m	30.2, CH_2	α 1.35 m<br>β 1.24 m	29.5, CH_2	α 1.78 m<br>β 1.33 m	28.8, CH_2	α 1.44 m<br>β 1.16 m
12	14.1, CH_3	0.82 t (7.1)	11.4, CH_3	0.89 t (7.4)	14.3, CH_3	0.83 t (7.1)	11.1, CH_3	0.87 t (7.4)
13	19.7, CH_3	0.99 d (6.3)	18.6, CH_3	0.94 d (6.6)	19.5, CH_3	0.99 d (6.3)	19.7, CH_3	0.92 d (6.4)

Measured in DMSO-d_6 at [a] 125 and [b] 500 MHz. Measured in chloroform-*d* at [c] 125 and [d] 500 MHz.

Generic Display Report

Analysis Info			
Analysis Name	S:\DATA\AmaZon\dva23_Daniela Valencia Revelo\Gymnopus montagnei\Gymnopus Rice MeOH fractions\R2F3_GB6_01_14570.d	Acquisition Date	22.06.2023 20:21:04
Method	14570.m	Operator	lab
Sample Name	R2F3	Instrument	amaZon speed
Comment			

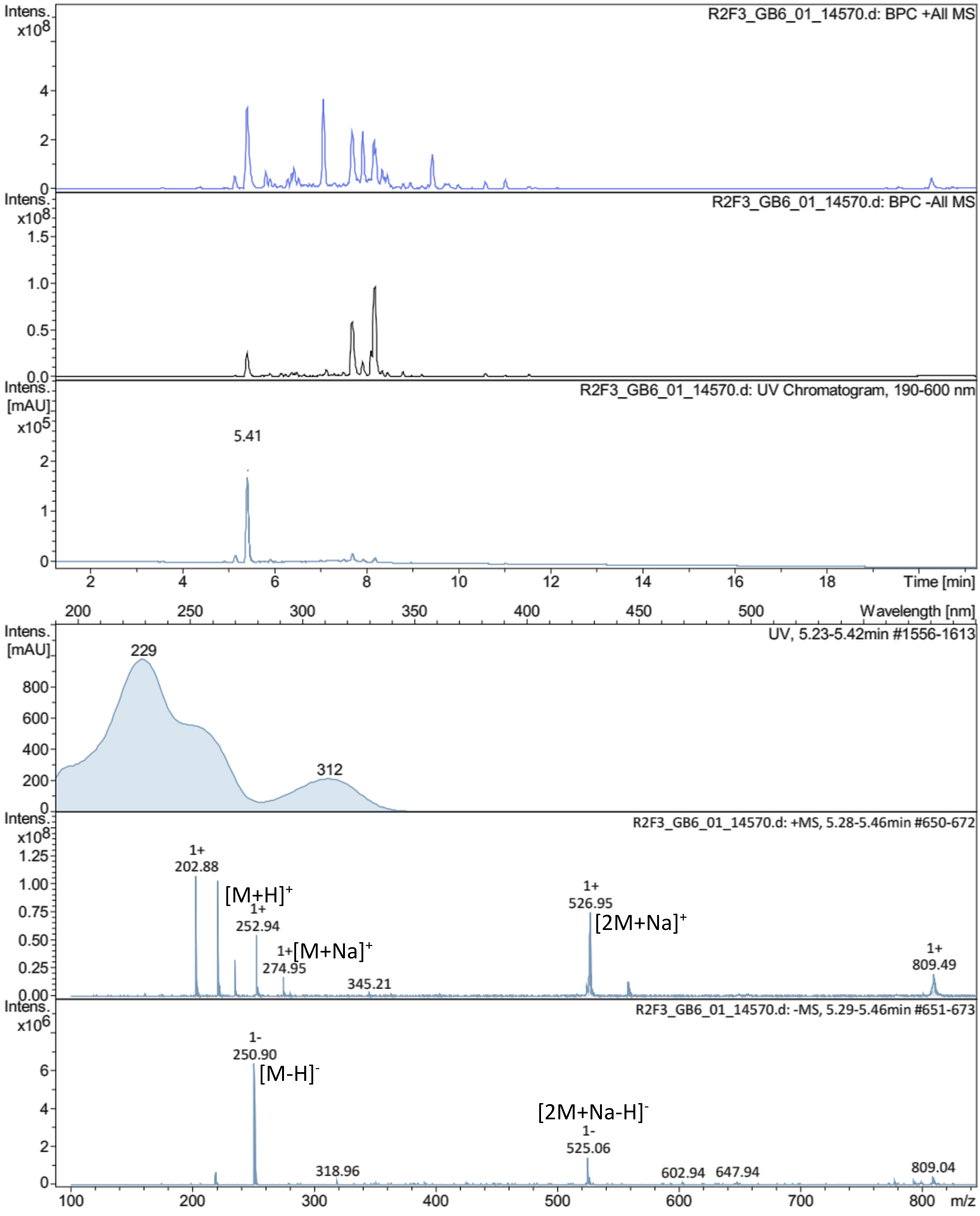

Bruker Compass DataAnalysis 4.4 printed: 26.06.2023 16:13:33 by: sel22 Page 1 of 1

Figure S14. LR-ESI-MS of **4**.

Generic Display Report

Analysis Info

Analysis Name	S:\DATA\MaXis\dva23_Daniela Valencia Revelo\23_06\23_06_22\23_06_22\Gymnopus-Rice - MeOH_R2_F3_34_01_11912.d	Acquisition Date	22.06.2023 19:50:49
Method	pos_saure_T0_08_screening_ms_100_2500_line.m	Operator	ate06
Sample Name	Gymnopus-Rice - MeOH_R2_F3	Instrument	maXis
Comment	Screening01<br>Waters Acquity UPLC BEH C_18 1,7um 2.1x50mm		

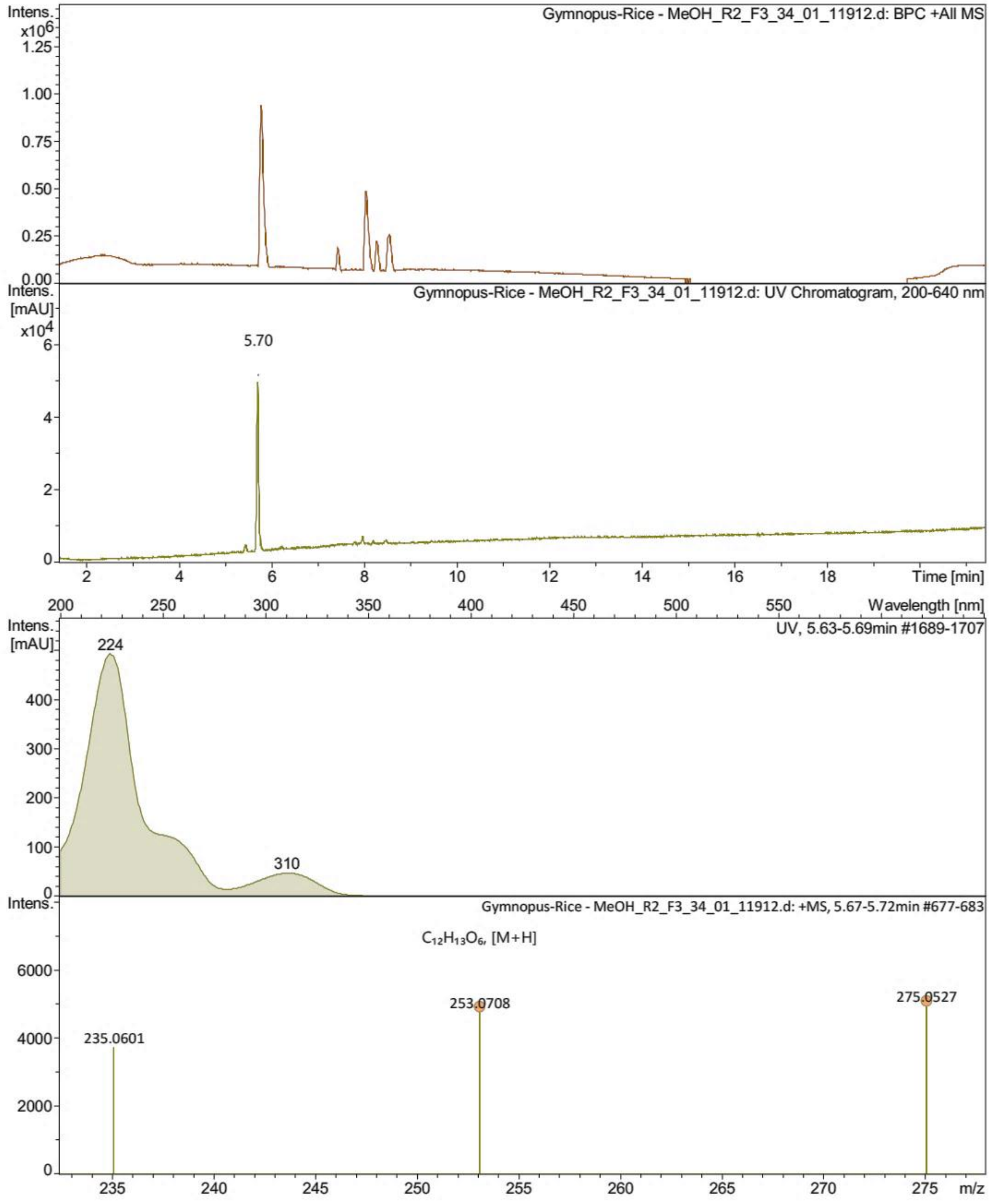

Figure S15. HR-ESI-MS of **4**.

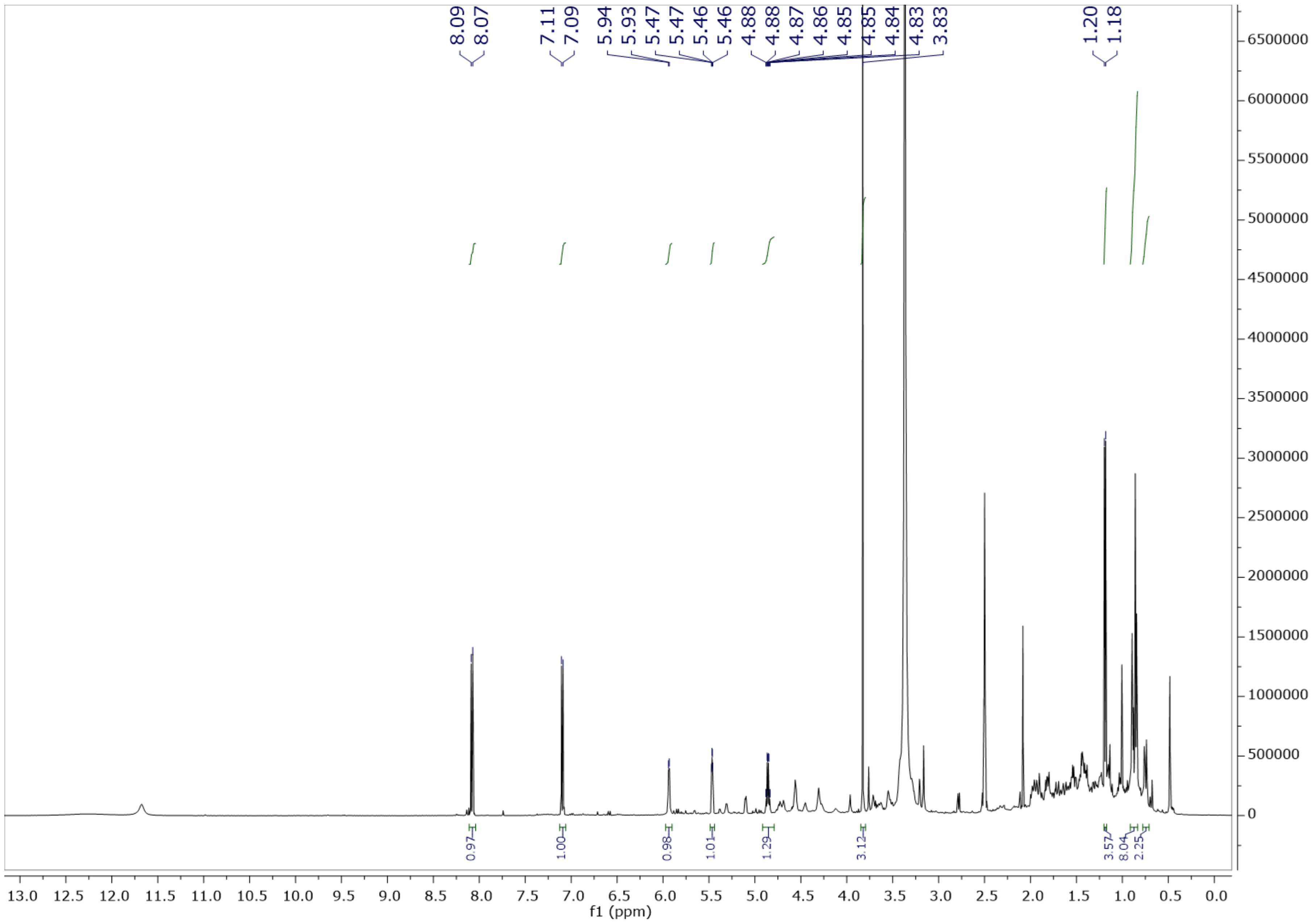

Figure S16. ^{1}H NMR spectrum of **4** in DMSO-d_6 at 500 MHz.

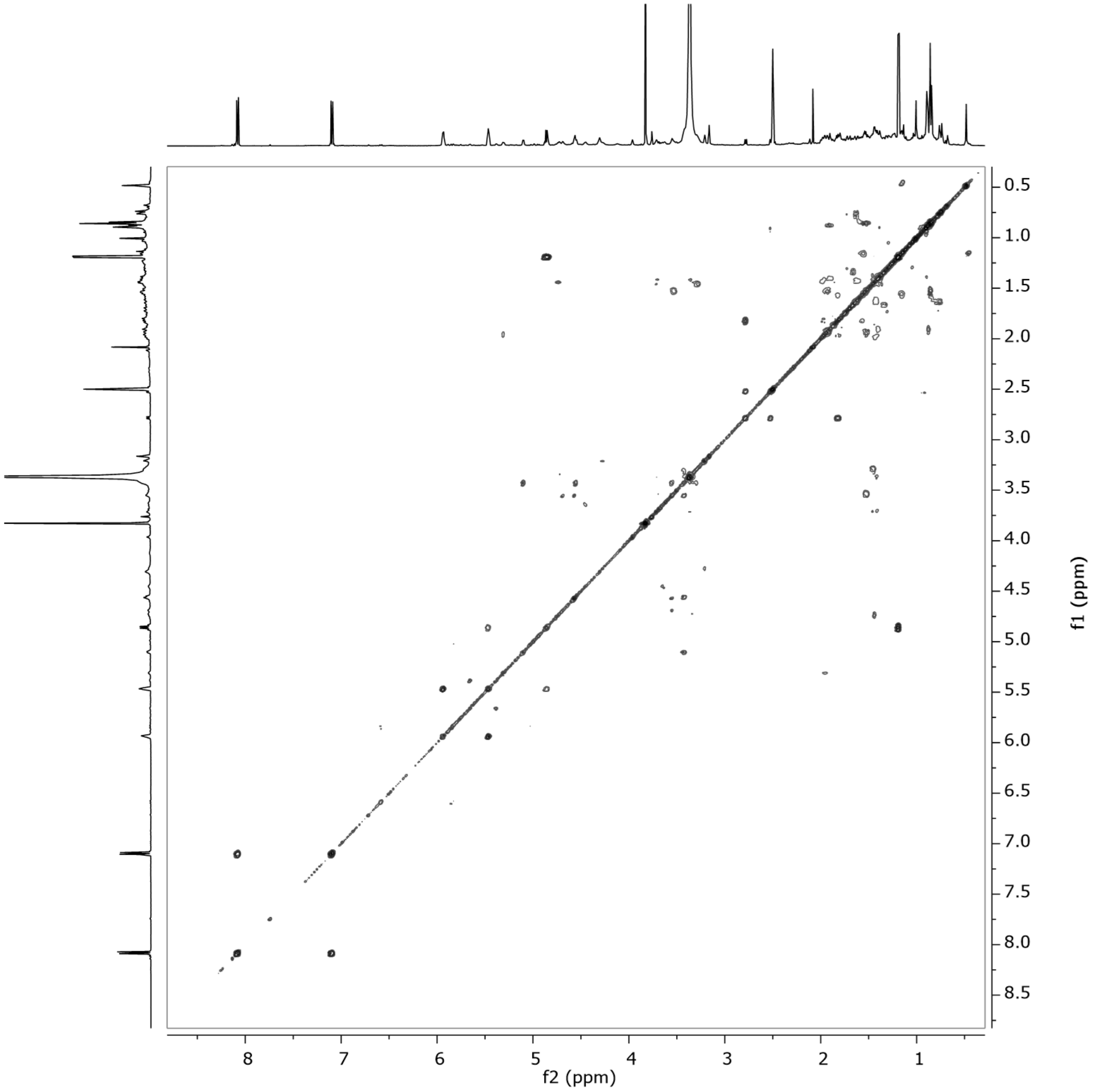

Figure S17. ^1H-^1H COSY spectrum of **4** in DMSO-d_6 at 500 MHz.

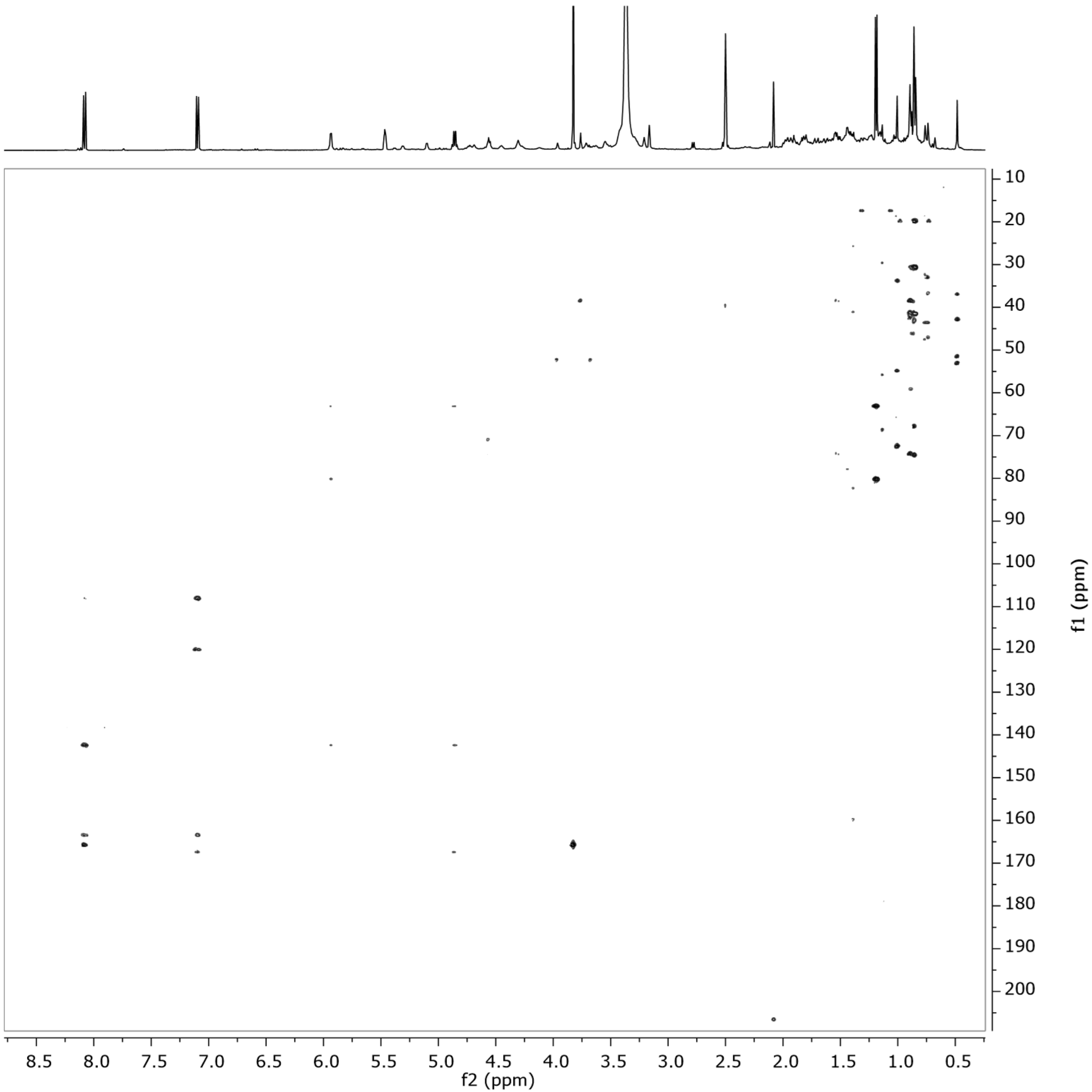

Figure S18. HMBC spectrum of **4** in DMSO-d_6 at 500 MHz.

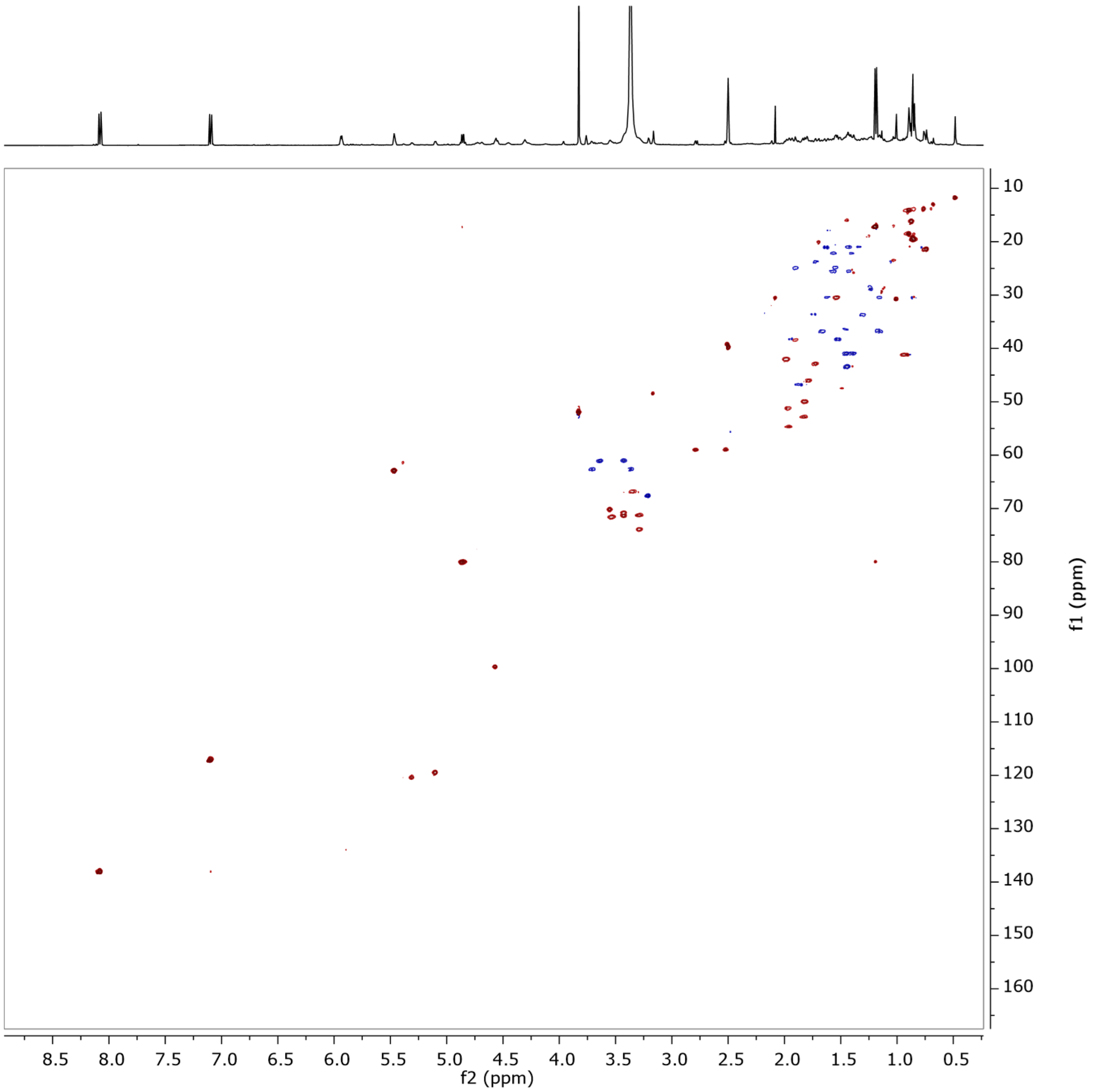

Figure S19. HSQC spectrum of **4** in DMSO-d_6 at 500 MHz.

Table S9. ^{1}H and ^{13}C NMR data of **4** and akolitserin.

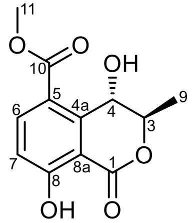

Akolitserin

	Compound **4**		Akolitserin	
pos.	δ_C,[a] type	δ_H[b] multi (*J* in Hz)	δ_C,[c] type	δ_H[d] multi (*J* in Hz)
1	167.3, CO		167.9, CO	
3	80.0, CH	4.86 qd (6.8, 2.5)	79.5, CH	5.05 qd (6.9, 1.5)
4	62.9, CH	5.47 dd (4.6, 2.4)	65.1, C	5.10 dd (3.6, 1.5)
4a	142.4, C		142.1, C	
5	120.0, C		120.0, C	
6	138.0, CH	8.08 d (8.9)	138.5, CH	8.13 d (8.7)
7	117.1, CH	7.10 d (8.9)	118.1, CH	7.06 d (8.7)
8	163.4, C		165.4, C	
8a	108.1, C		107.6, C	
9	17.2, CH_3	1.19 d (6.8)	18.8, CH_3	1.35 d (6.9)
10	165.7, CO		167.0, CO	
11	52.0, CH_3	3.83 s	52.7, CH_3	3.95 s
4-OH	-	5.94 d (5.1)	-	3.91 br d (3.6)
8-OH	-	11.68 br s	-	11.98 s

Measured in DMSO-d_6 at [a] 125 and [b] 500 MHz.
Measured in chloroform-*d* at [c] 150 and [d] 600 MHz.

Generic Display Report

Analysis Info

Analysis Name	S:\DATA\MaXis\dva23_Daniela Valencia Revelo\23_06\23_06_22\Gymnopus-Rice - MeOH_R3_F4_47_01_11927.d	Acquisition Date	23.06.2023 03:35:21
Method	pos_stauro_f0060_screening_ms_100_2500_line.m	Operator	ate06
Sample Name	Gymnopus-Rice - MeOH_R3_F4	Instrument	maXis
Comment	Screening01<br>Waters Acquity UPLC BEH C_18 1,7um 2.1x50mm		

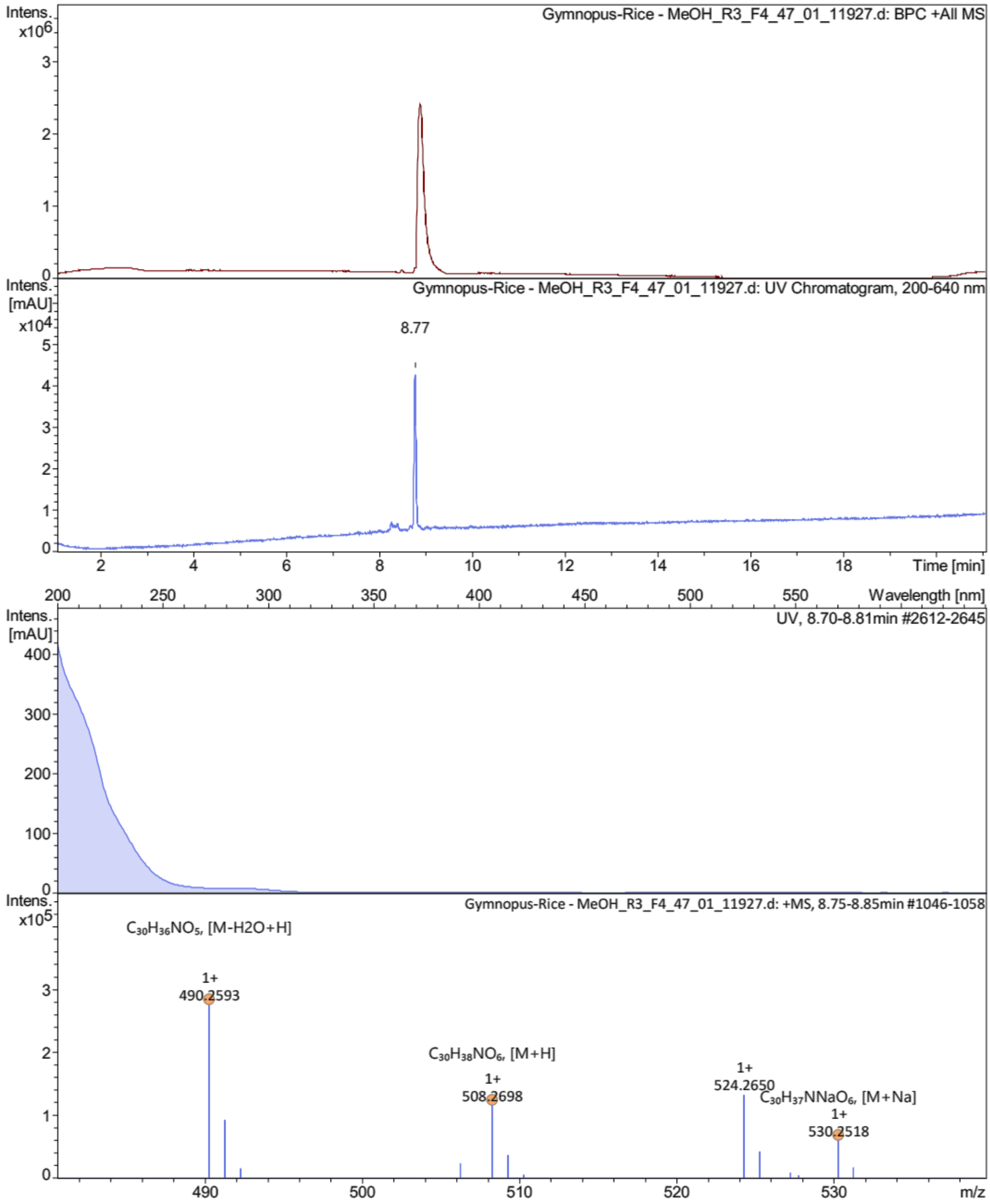

Figure S20. HR-ESI-MS of **5**.

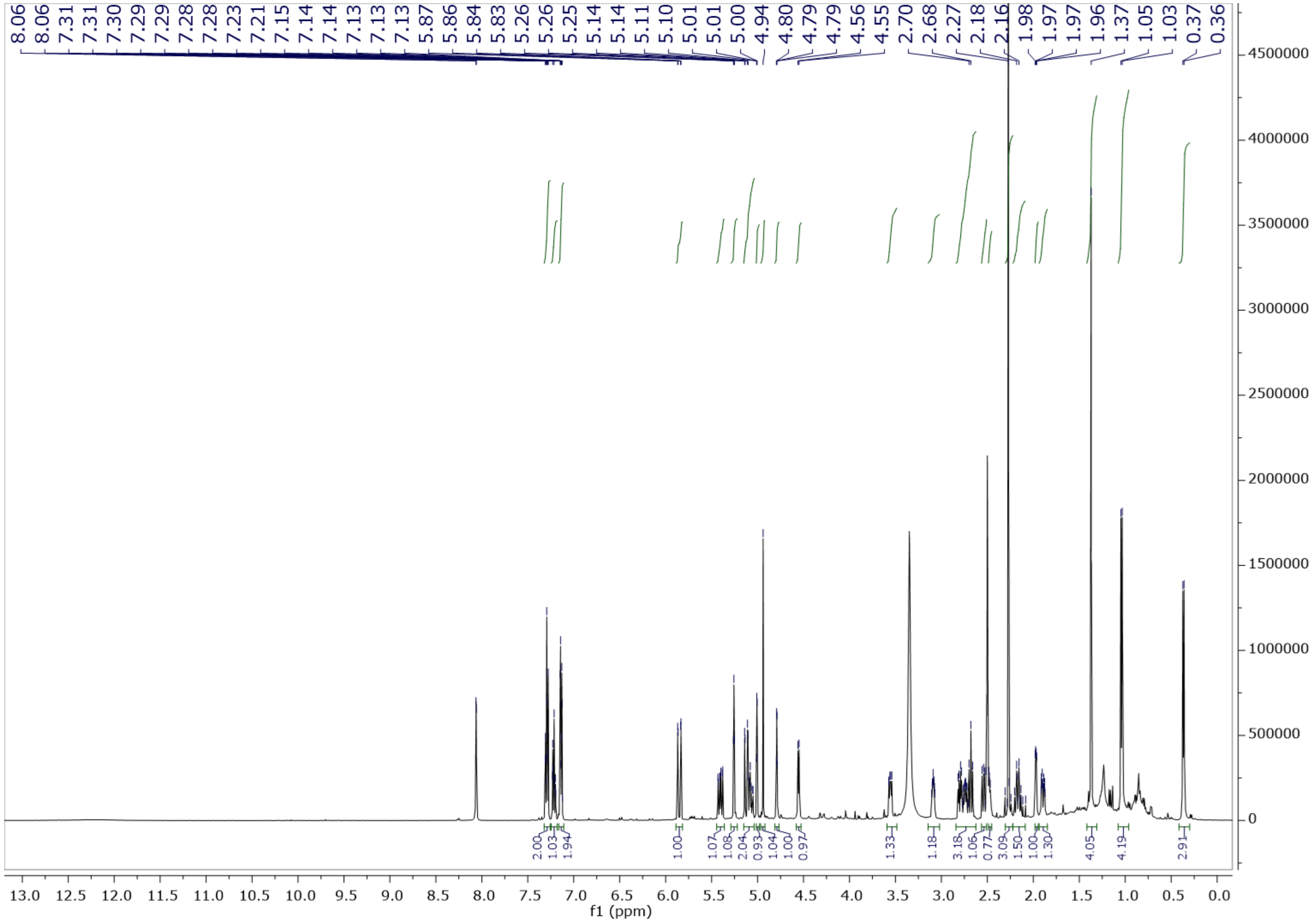

Figure S21. ^{1}H NMR spectrum of **5** in DMSO-d_6 at 500 MHz.

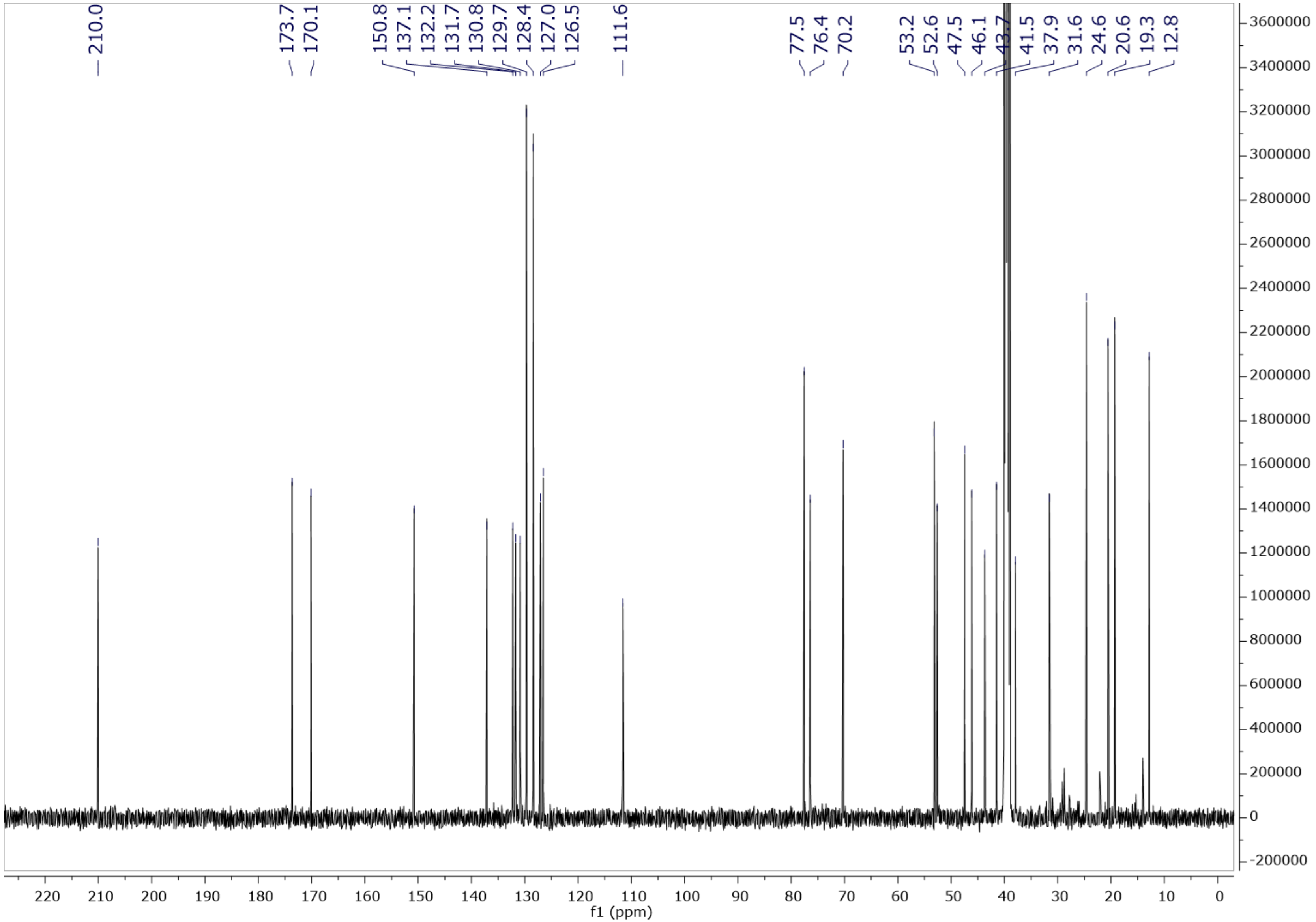

Figure S22. ^{13}C NMR spectrum of **5** in DMSO-d_6 at 125 MHz.

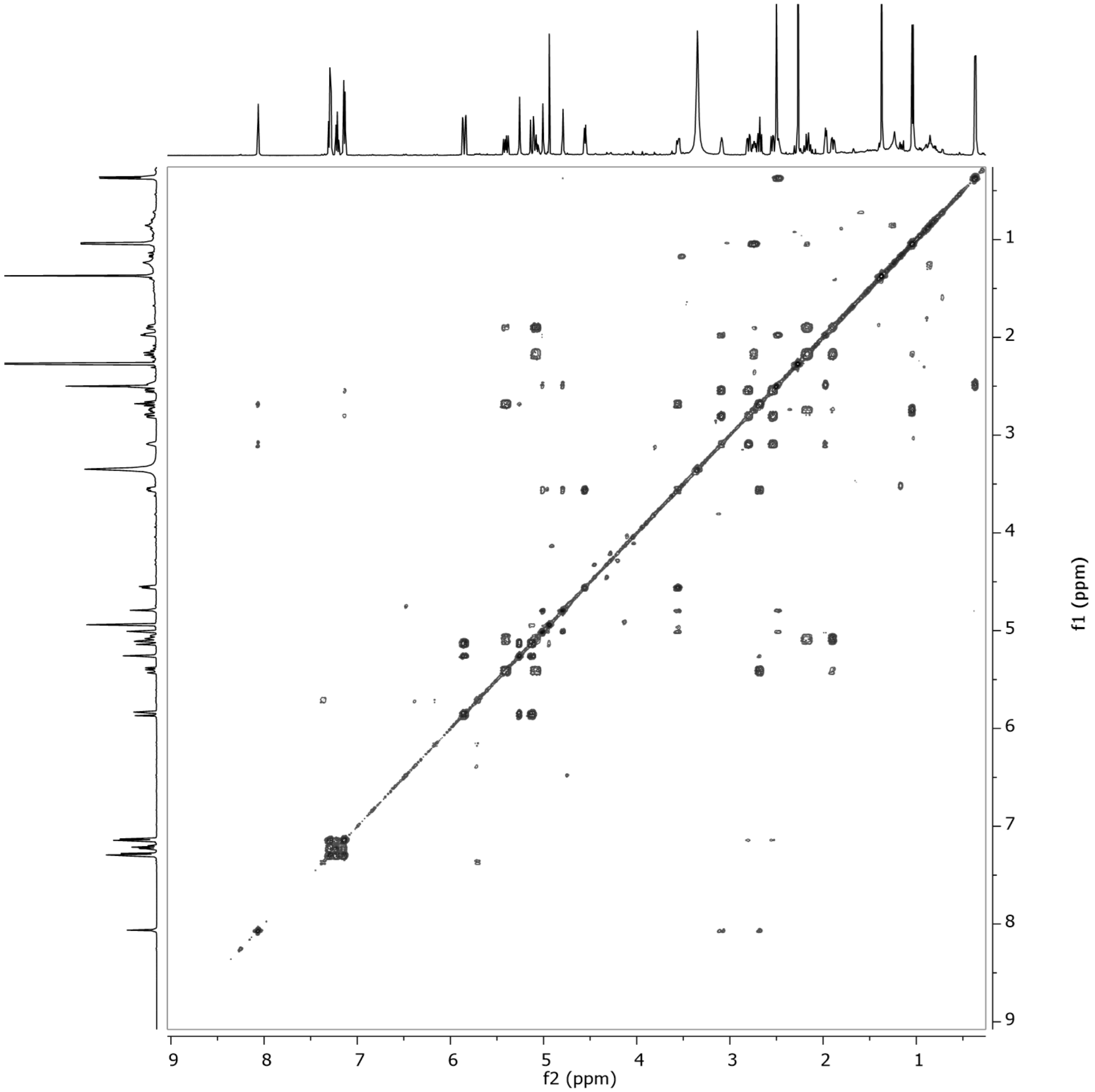

Figure S23. ^{1}H-^{1}H COSY spectrum of **5** in DMSO-d_6 at 500 MHz.

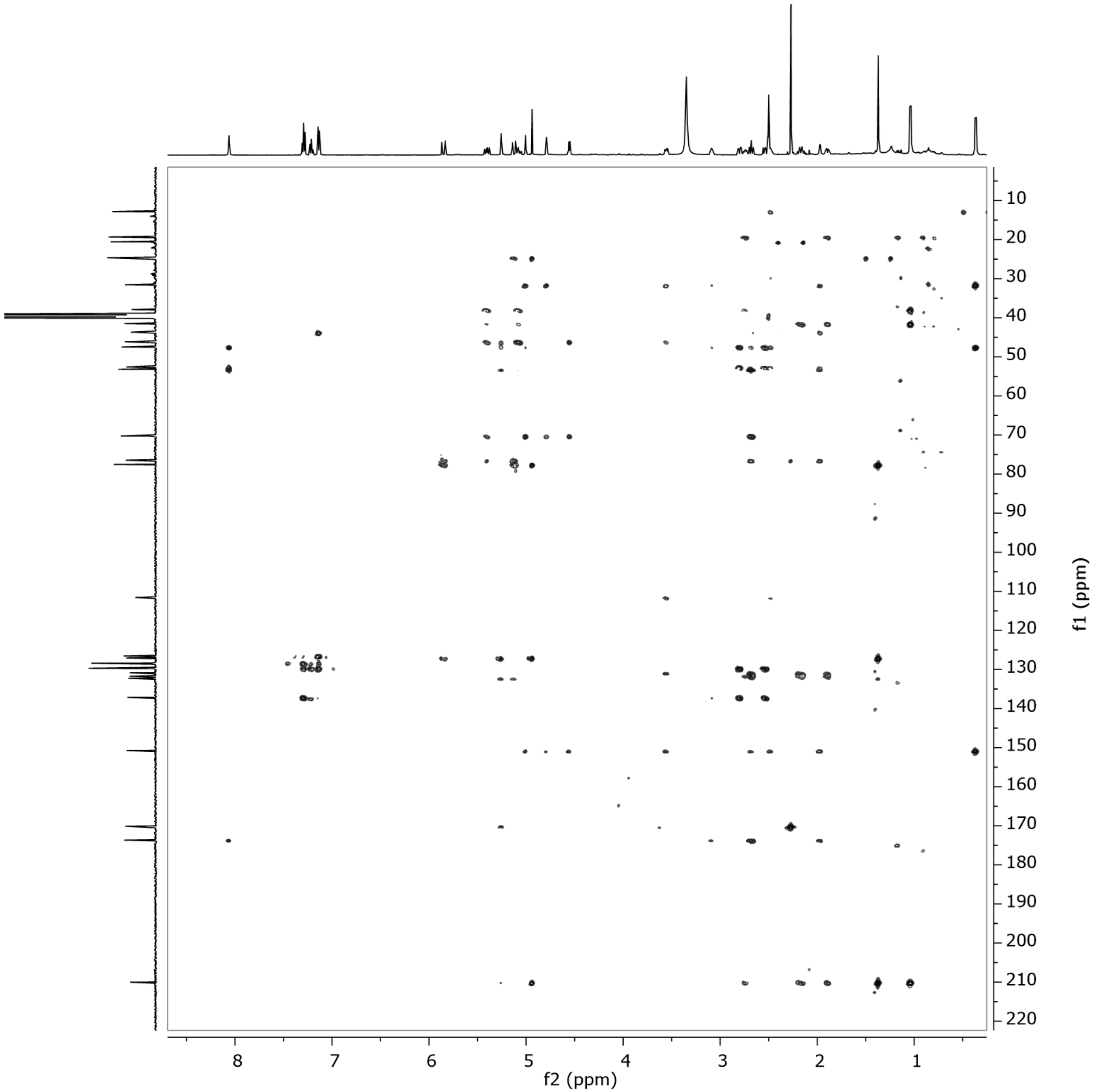

Figure S24. HMBC spectrum of **5** in DMSO-d_6 at 500 MHz.

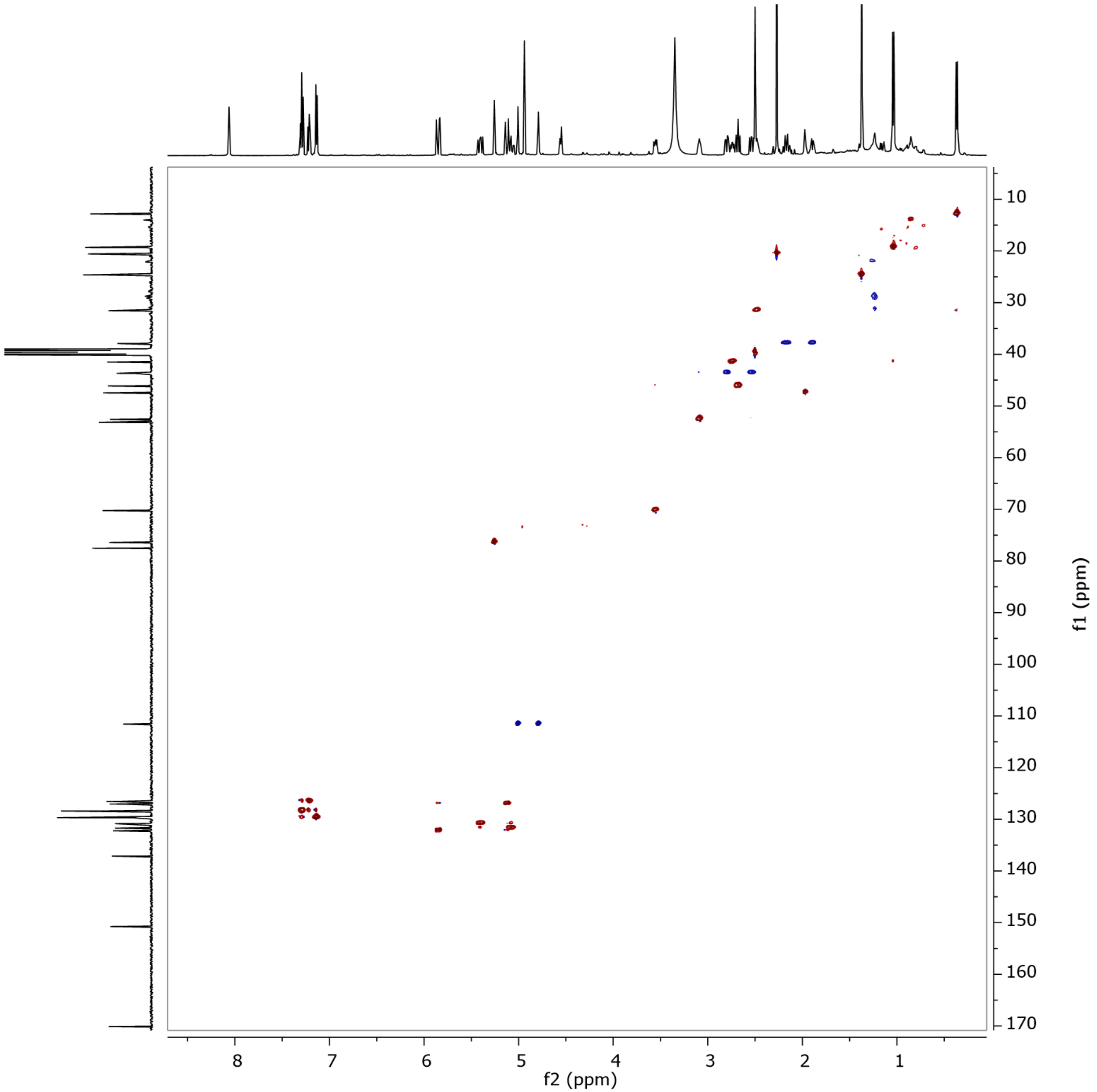

Figure S25. HSQC spectrum of **5** in DMSO-d_6 at 500 MHz.

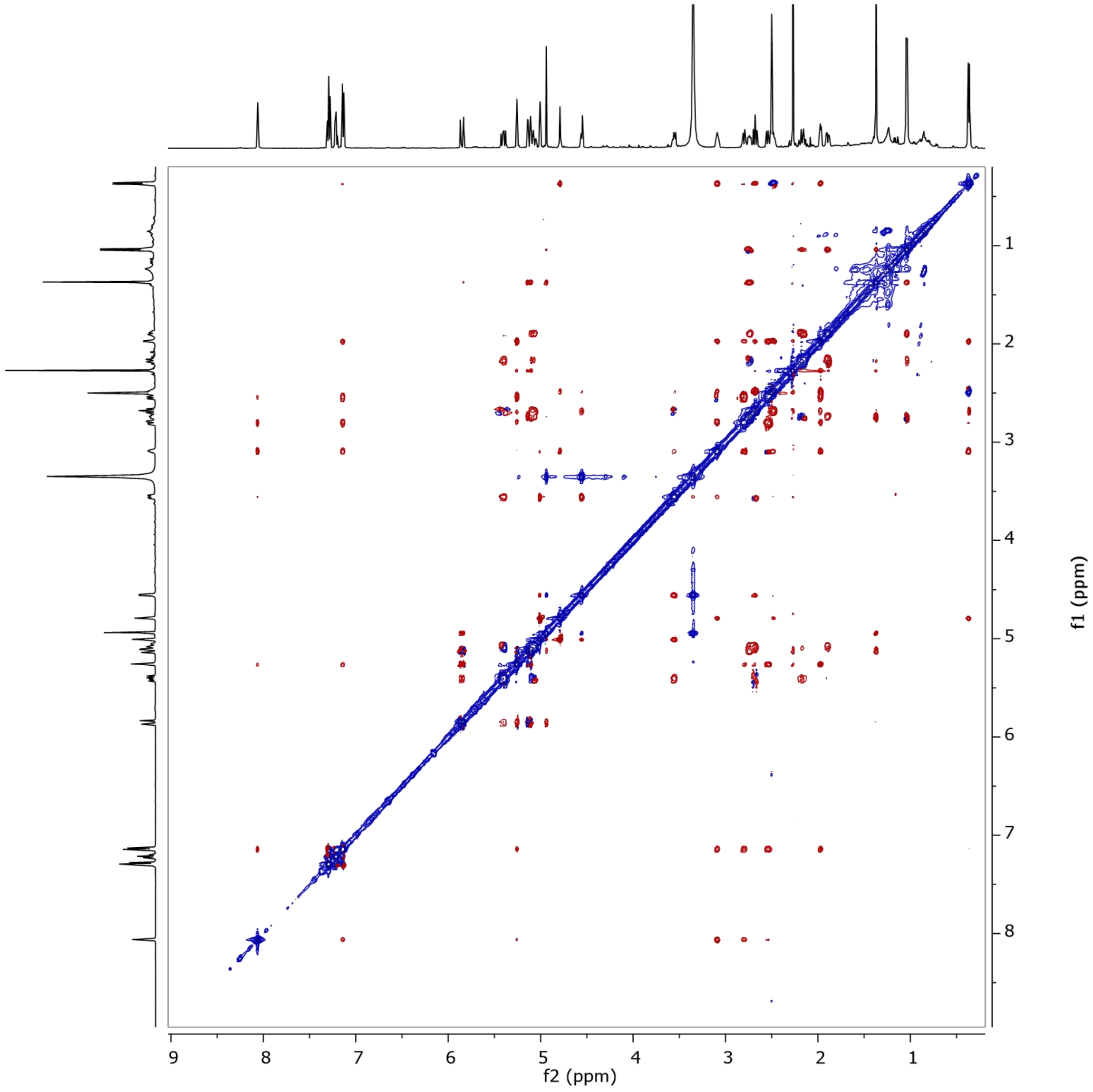

Figure S26. ROESY spectrum of **5** in DMSO-d_6 at 500 MHz.

Table S10. ^{1}H and ^{13}C NMR data of **5** and hypoxylin A.

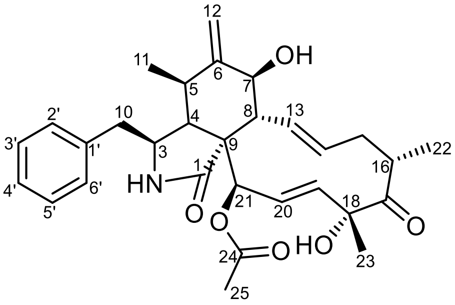

Hypoxylin A

	Compound **5**		Hypoxylin A	
pos.	δ_C,[a] type	δ_H[b] multi (*J* in Hz)	δ_C,[c] type	δ_H[d] multi (*J* in Hz)
1	173.7, CO		173.7, CO	
2-N*H*	-	8.06 d (1.4)	-	-
3	52.6, CH	3.09 m	56.0, CH	4.26 ddd (9.5, 5.0, 3.5)
4	47.5, CH	1.97 dd (5.6, 2.6)	45.2, CH	2.33 dd (5.0, 3.5)
5	31.6, CH	2.48 m	32.6, CH	2.63 m
6	150.8, C		143.5, C	
7	70.2, CH	3.56 dd (10.0, 5.5)	74.1, CH	5.38 d (10.0)
7-O*H*		4.55 d (5.5)		
8	46.1, CH	2.68 t (10.0)	45.1, CH	3.17 t (10.0)
9	53.2, C		56.3, C	
10		α 2.80 dd (13.0, 4.8)<br>β 2.54 dd (13.0, 8.8)	41.2, CH_2	α 3.35 dd (13.5, 5.0)<br>β 2.83 dd (13.5, 9.5)
11	12.8, CH_3	0.37 d (6.7)	12.1, CH_3	0.34 d (7.0)
12	111.6, CH_2	α 5.01 d (1.5)<br>β 4.79 q (1.5)	117.4, CH_2	α 5.38 br s<br>β 5.07 br s
13	130.8, CH	5.40 ddd (15.4, 9.5, 1.2)	130.9, CH	5.49 dd (15.5, 10.0)
14	131.7, CH	5.07 td (10.6, 5.3)	132.9, CH	5.22 ddd (15.5, 10.5, 5.0)
15	37.9, CH_2	α 2.17 q (11.0)<br>β 1.89 dd (12.9, 5.0)	37.7, CH_2	α 2.25 ddd (13.0, 12.5, 10.5)<br>β 1.77 dd (13.0, 5.0)
16	41.5, CH	2.74 ddd (11.0, 6.8, 1.7)	42.3, CH	2.66 dq (12.5, 7.0)
17	210.0, CO		210.2, CO	
18	77.5, C		77.7, C	
19	127.0, CO	5.12 dd (15.9, 2.5)	129.5, CH	5.18 dd (15.5, 2.0)
20	132.2, CH	5.85 dd (15.9, 2.5)	130.7, CH	5.85 dd (15.5, 2.0)
21		5.26 t (2.5)	74.4	5.99 t (2.0)
22	19.3, CH_3	1.04 d (6.8)	19.4, CH_3	1.10 d (7.0)
23	24.6, CH_3	1.37 s	24.1, CH_3	1.48 s
24	170.1, CO		169.2, CO	
25	20.6, CH_3	2.27 s	20.8, CH_3	2.41 s
1'	137.1, C		136.7, C	
2'	129.7, CH	7.14 d (7.4)	129.1, CH	7.29 d (8.0)
3'	128.4, CH	7.29 t (7.4)	128.3, CH	7.37 t (8.0)
4'	126.5, CH	7.21 t (7.4)	127.1, CH	7.31 t (8.0)
5'	128.4, CH	7.29 t (7.4)	128.3, CH	7.37 t (8.0)
6'	129.7, CH	7.14 d (7.4)	129.1, CH	7.29 d (8.0)

Measured in DMSO-d_6 at [a] 125 and [b] 500 MHz.

Measured in chloroform-*d* at [c] 150 and [d] 600 MH.

Generic Display Report

Analysis Info

Analysis Name S:\DATA\AmaZon\dva23_Daniela Valencia Revelo\Gymnopus montagnei\Gymnopus Rice MeOH fractions\R1F6_GA6_01_14560.d

Method 14560.m

Sample Name R1F6

Comment

Acquisition Date 22.06.2023 14:19:08

Operator lab

Instrument amaZon speed

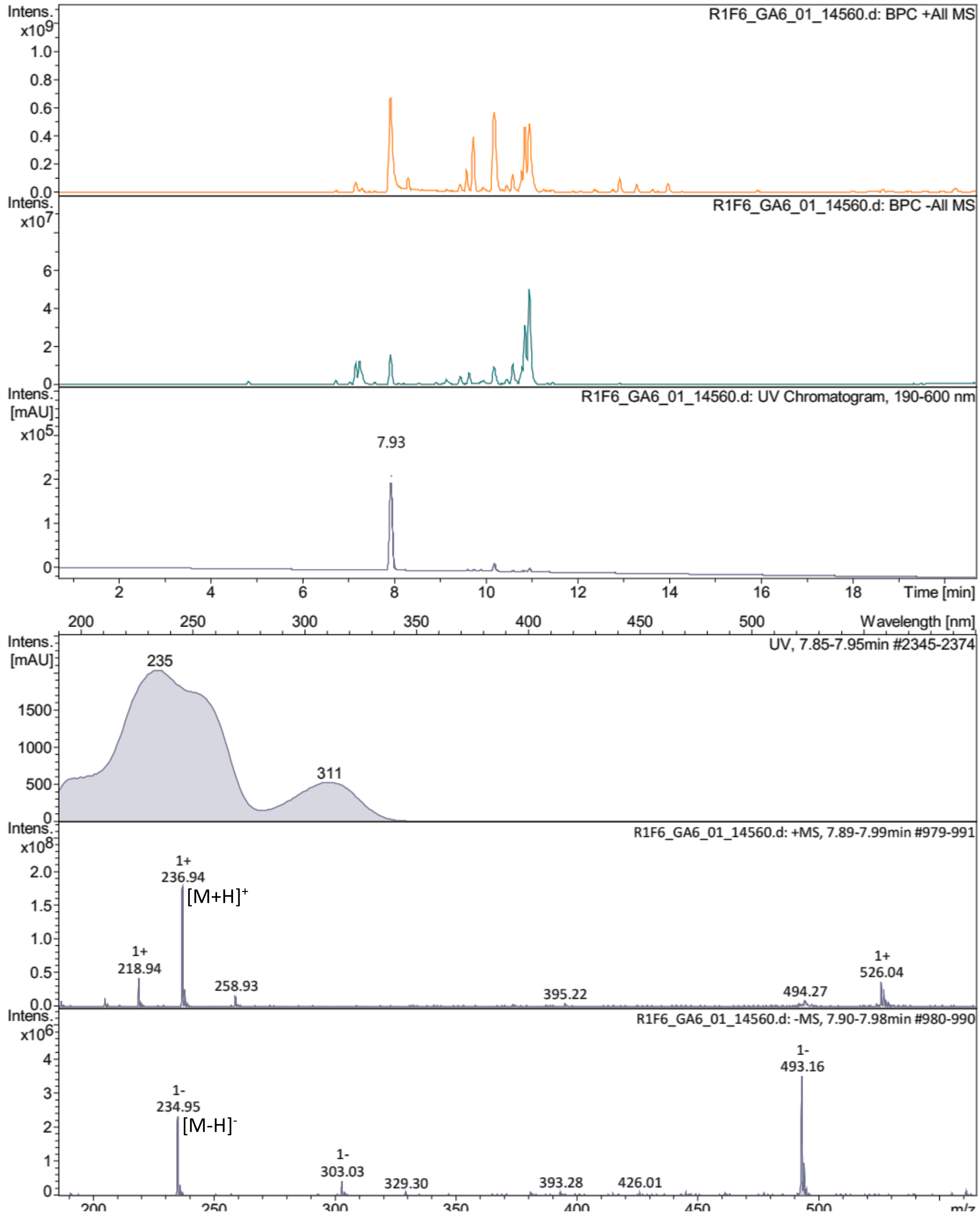

Bruker Compass DataAnalysis 4.4 printed: 26.06.2023 15:59:59 by: sel22 Page 1 of 1

Figure S27. LR-ESI-MS of **6**.

Generic Display Report

Analysis Info

Analysis Name	S:\DATA\MaXis\dva23_Daniela Valencia Revelo\23_06\23_06_22\23_06_22\Gymnopus-Rice - MeOH_R1_F6_26_01_11902.d	Acquisition Date	22.06.2023 14:41:08
Method	pos_saure_f0026_screening_ms_100_2500_line.m	Operator	ate06
Sample Name	Gymnopus-Rice - MeOH_R1_F6	Instrument	maXis
Comment	Screening01<br>Waters Acquity UPLC BEH C_18 1,7um 2.1x50mm		

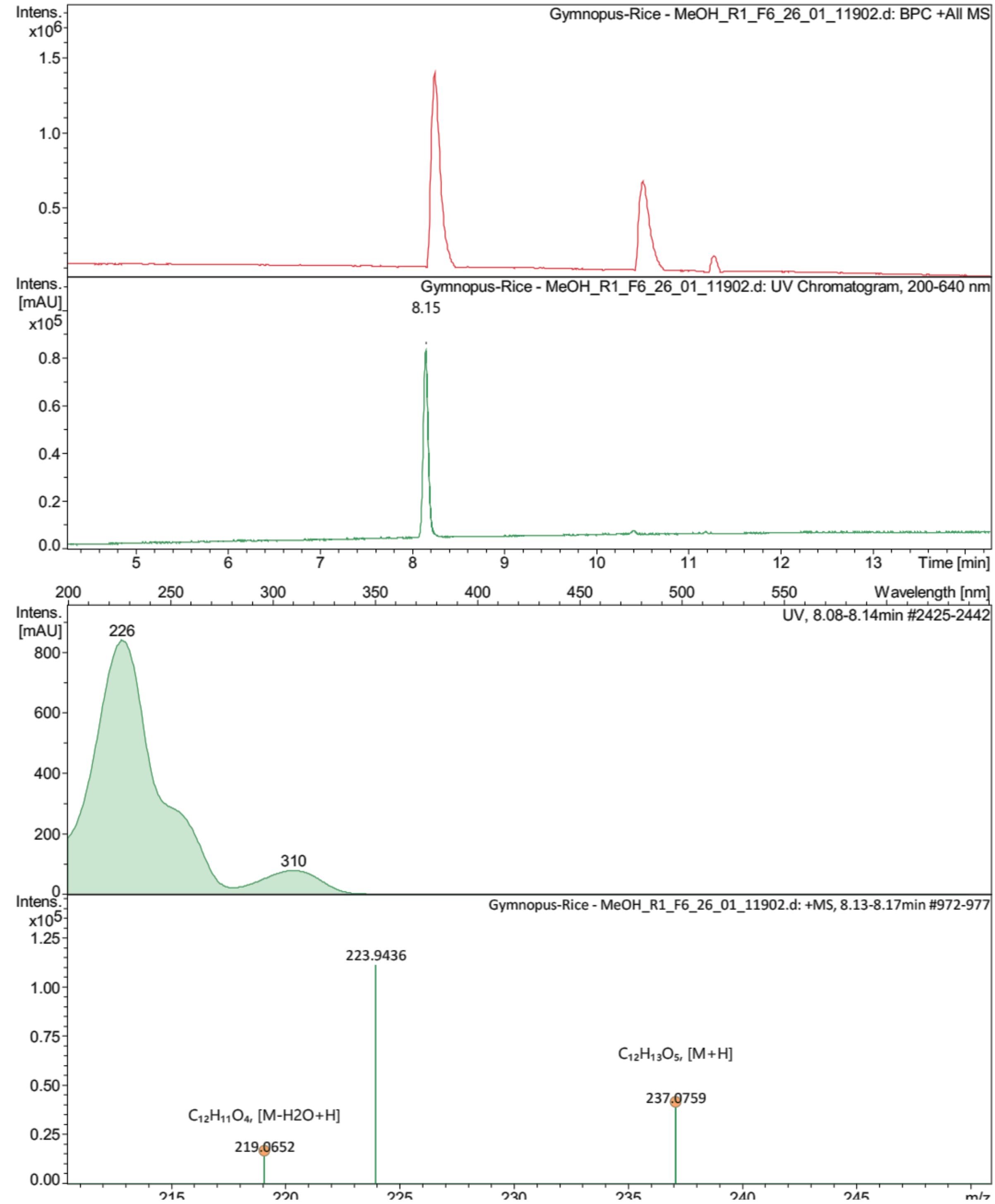

Bruker Compass DataAnalysis 4.4 printed: 26.06.2023 18:00:13 by: sel22 Page 1 of 1

Figure S28. HR-ESI-MS of **6**.

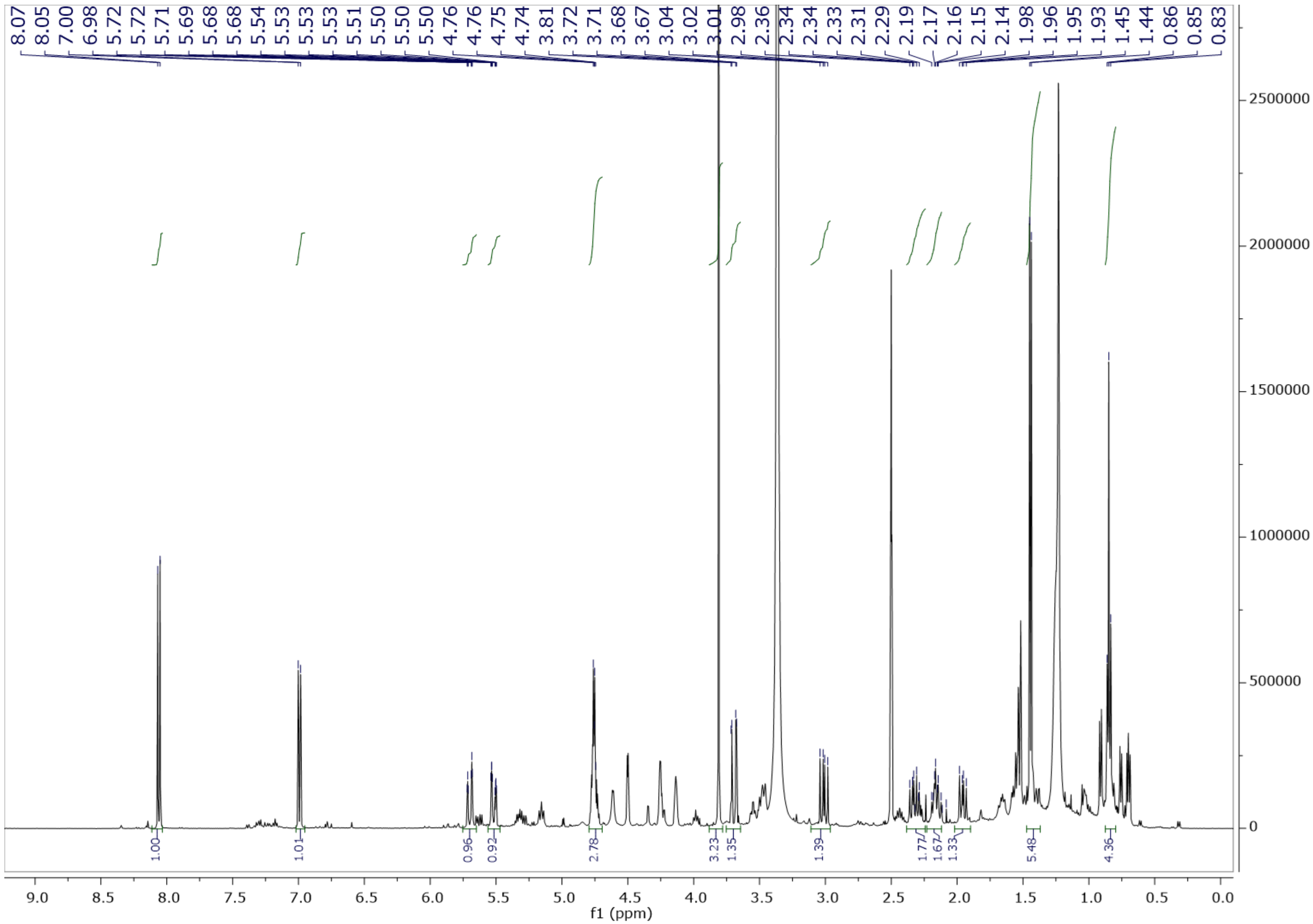

Figure S29. ^{1}H NMR spectrum of **6** in DMSO-d_6 at 500 MHz.

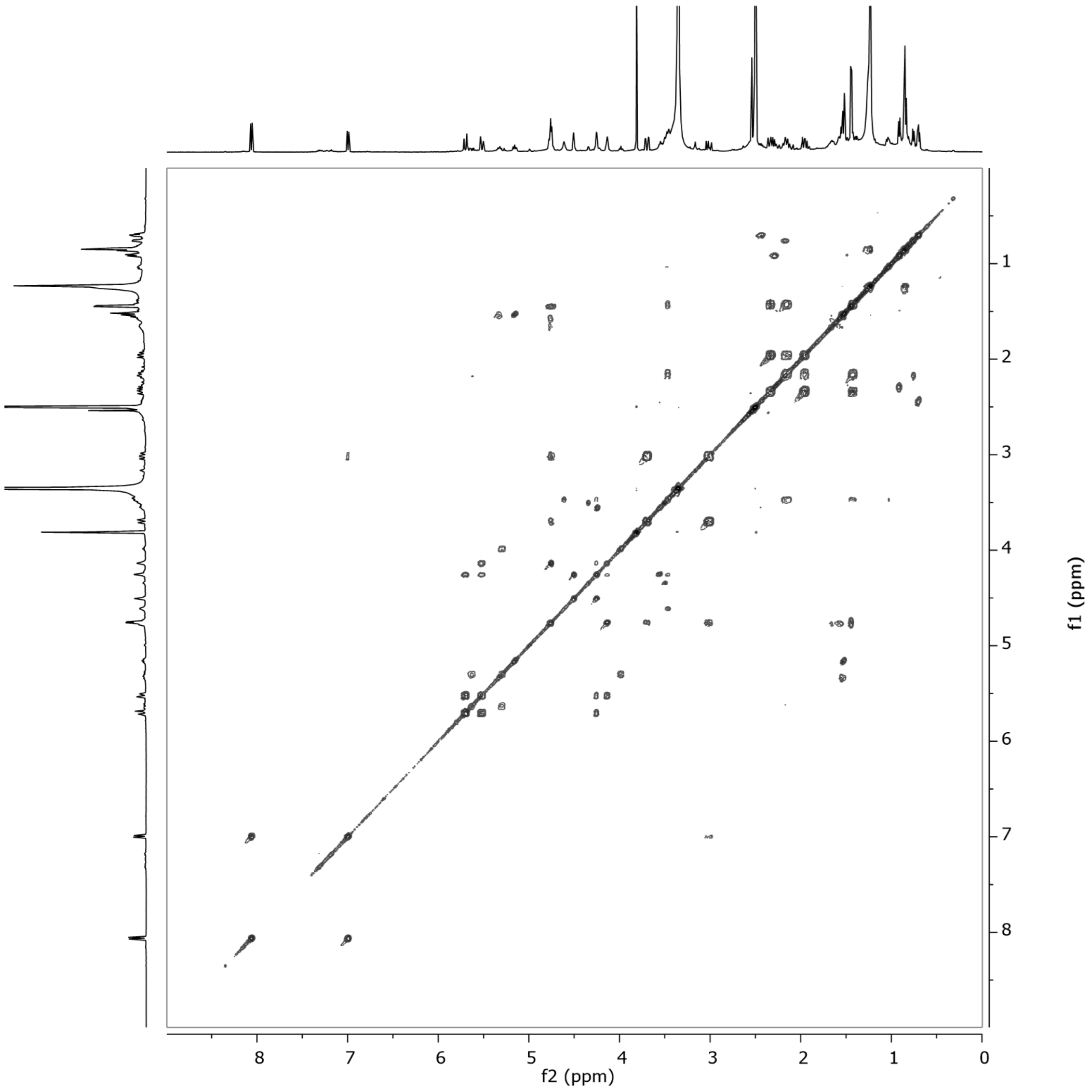

Figure S30. ^1H-^1H COSY spectrum of **6** in DMSO-d_6 at 500 MHz.

Table S11. ^{1}H and ^{13}C NMR data of **6** and (-)-(*R*)-5-(methoxycarbonyl)mellein.

	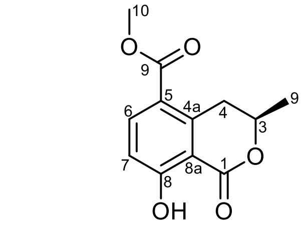 (-)-(*R*)-5-(Methoxycarbonyl)mellein	
	Compound **6**	(-)-(*R*)-5-(Methoxycarbonyl)mellein
pos.	δ_H[a] multi (*J* in Hz)	δ_H[b] multi (*J* in Hz)
1		
3	4.75 dtt (11.9, 6.3, 3.0)	4.60 m
4	α 3.70 dd (17.6, 3.0)<br>β 3.01 dd (17.7, 11.9)	α 3.75 m<br>β 3.15 m
4a		
5		
6	8.06 d (8.9)	8.18 d (8.0)
7	6.99 d (8.9)	6.94 d (8.0)
8		
8a		
9	1.44 d (6.3)	1.45 d (7.0)
10	3.81 s	
8-OH	11.70 br s	11.80 br s

Measured in DMSO-d_6 at [a] 500 MHz.
Measured in acetone-d_6 at [b] 100 MHz.

Generic Display Report

Analysis Info

Analysis Name	S:\DATA\AmaZon\dva23_Daniela Valencia Revelo\Gymnopus montagnei\Gymnopus YM Sup fractions\Gymnopus YM Sup\Gymnopus YM Sup R1F7_BA7_01_48700.d	Acquisition Date	08.07.2023 21:30:46
Method	48700.m	Operator	tti
Sample Name	Gymnopus YM Sup R1F7	Instrument	amaZon speed
Comment			

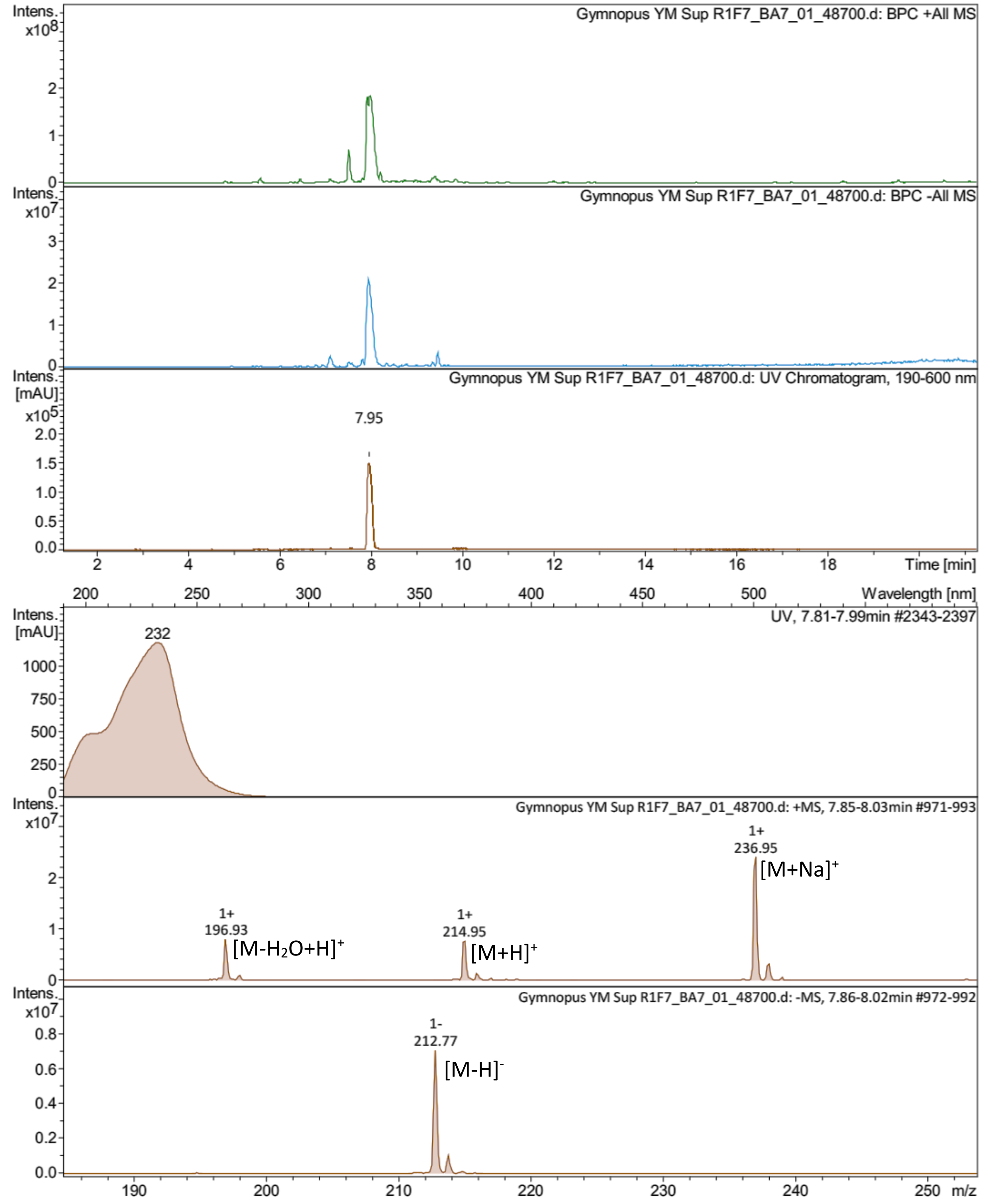

Bruker Compass DataAnalysis 4.4 printed: 18.07.2023 10:15:20 by: sel22 Page 1 of 1

Figure S31. LR-ESI-MS of **7**.

Generic Display Report

**Analysis Info**		Acquisition Date	14.07.2023 20:48:59
Analysis Name	S:\DATA\MaXis\dva23_Daniela Valencia Revelo\23_07\23_07_14\Gymnopus YM Sup_R1_F7_23_01_12418.d		
Method	pos_saure_1000_screening_ms_100_2500_line.m	Operator	ate06
Sample Name	Gymnopus YM Sup_R1_F7	Instrument	maXis
Comment	Screening01<br>Waters Acquity UPLC BEH C_18 1,7um 2.1x50mm		

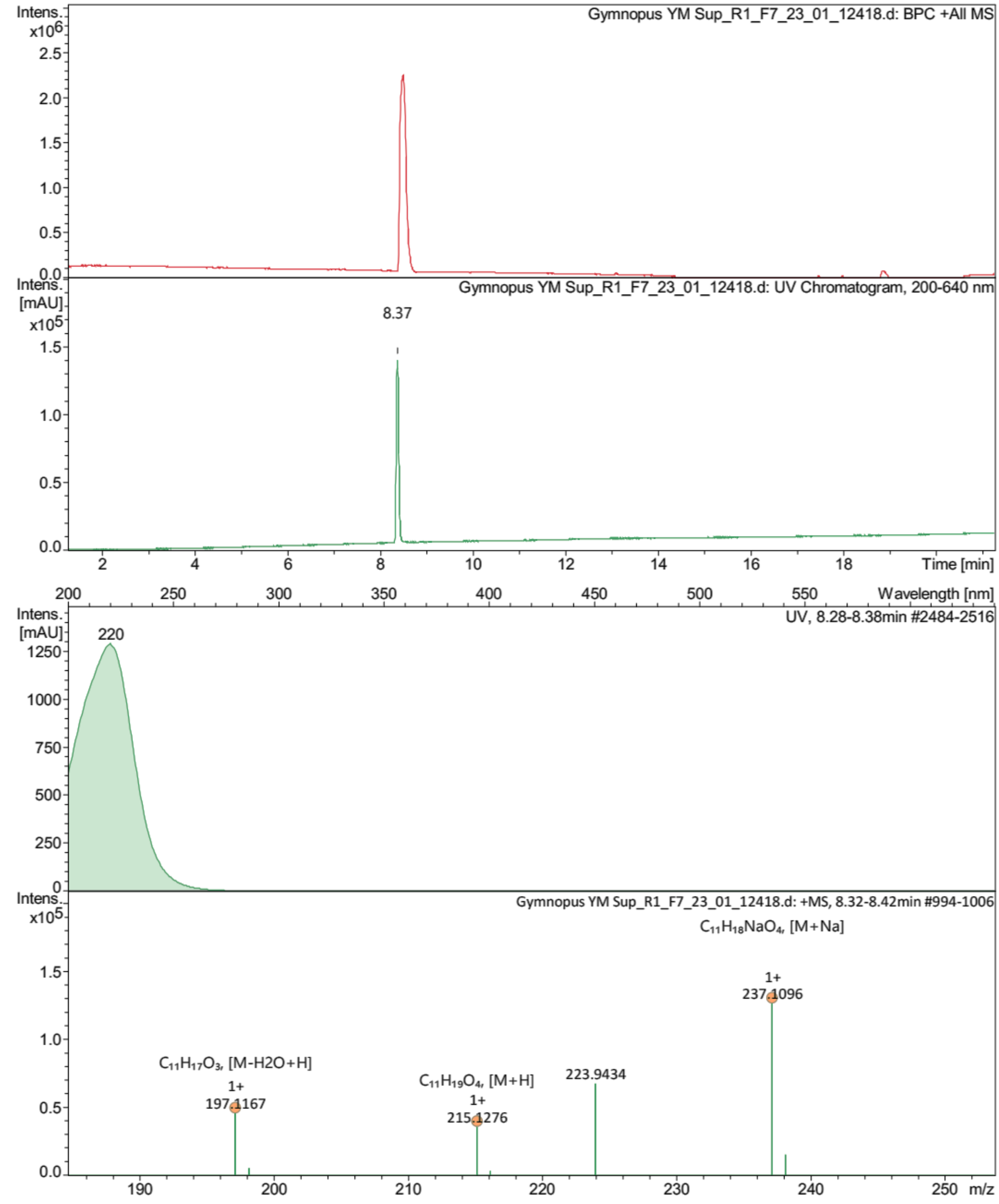

Bruker Compass DataAnalysis 4.4	printed: 18.07.2023 10:14:08	by: sel22	Page 1 of 1

Figure S32. HR-ESI-MS of **7**.

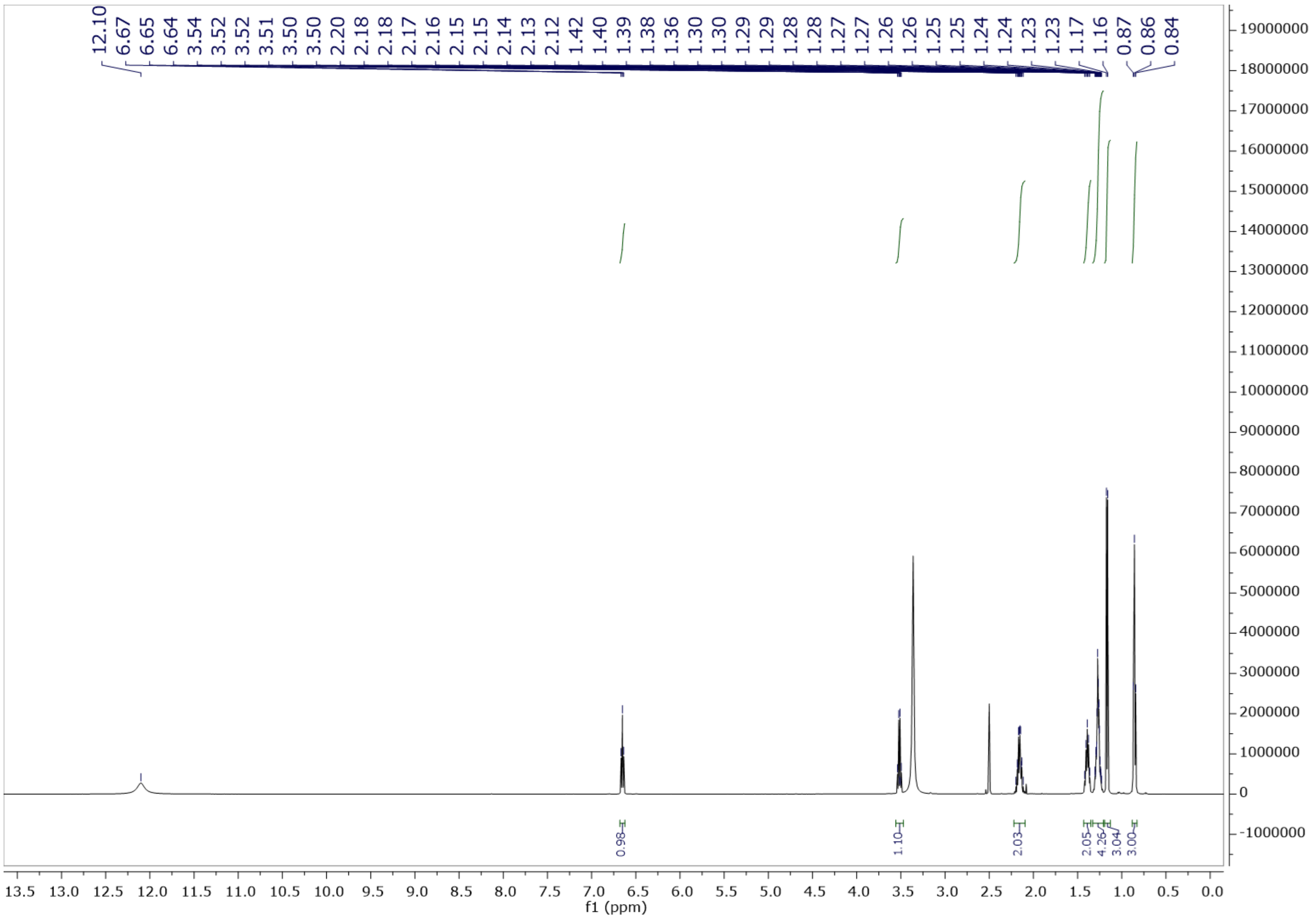

Figure S33. ^{1}H NMR spectrum of **7** in DMSO-d_6 at 500 MHz.

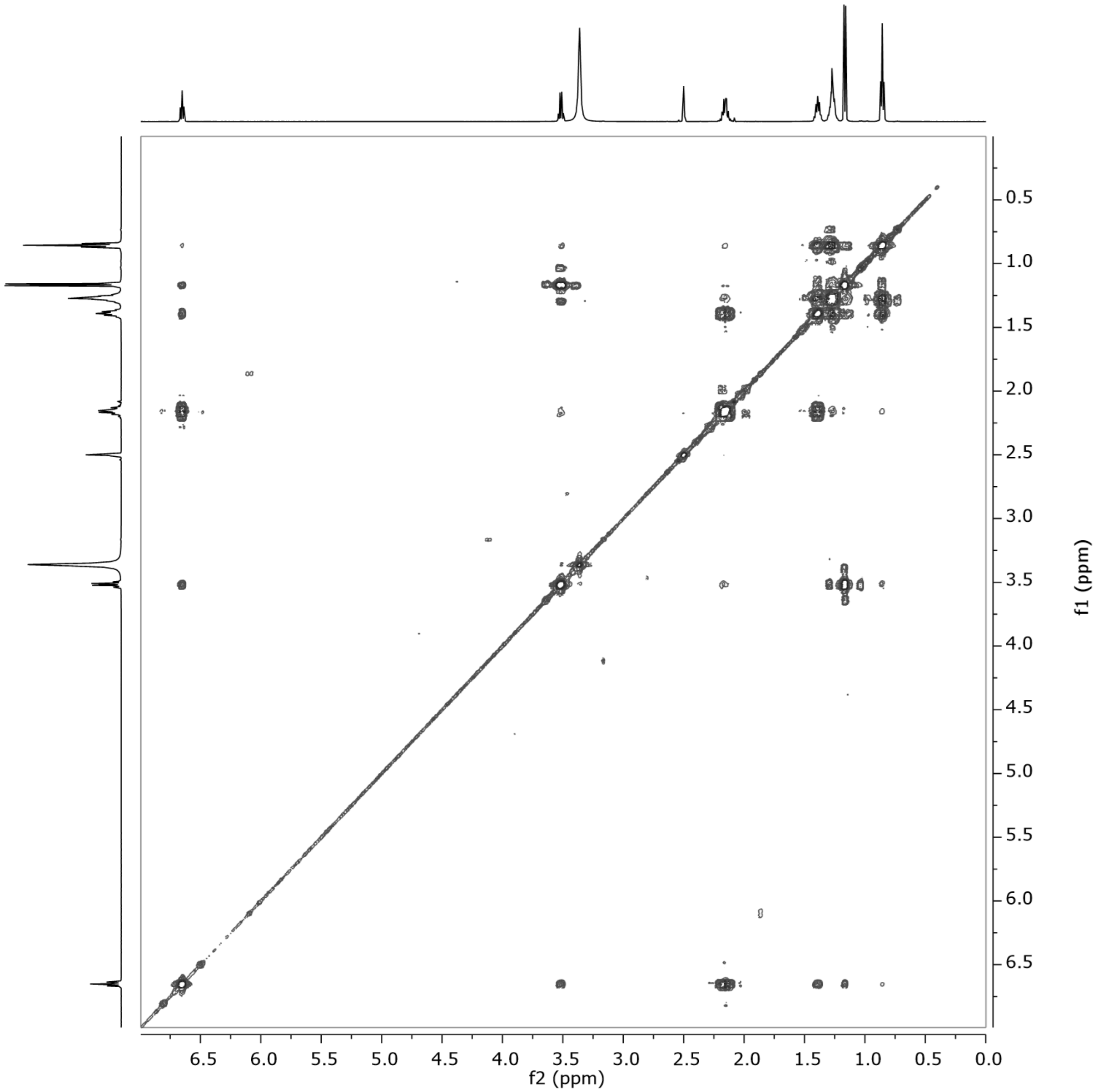

Figure S34. ^{1}H-^{1}H COSY spectrum of **7** in DMSO-d_6 at 500 MHz.

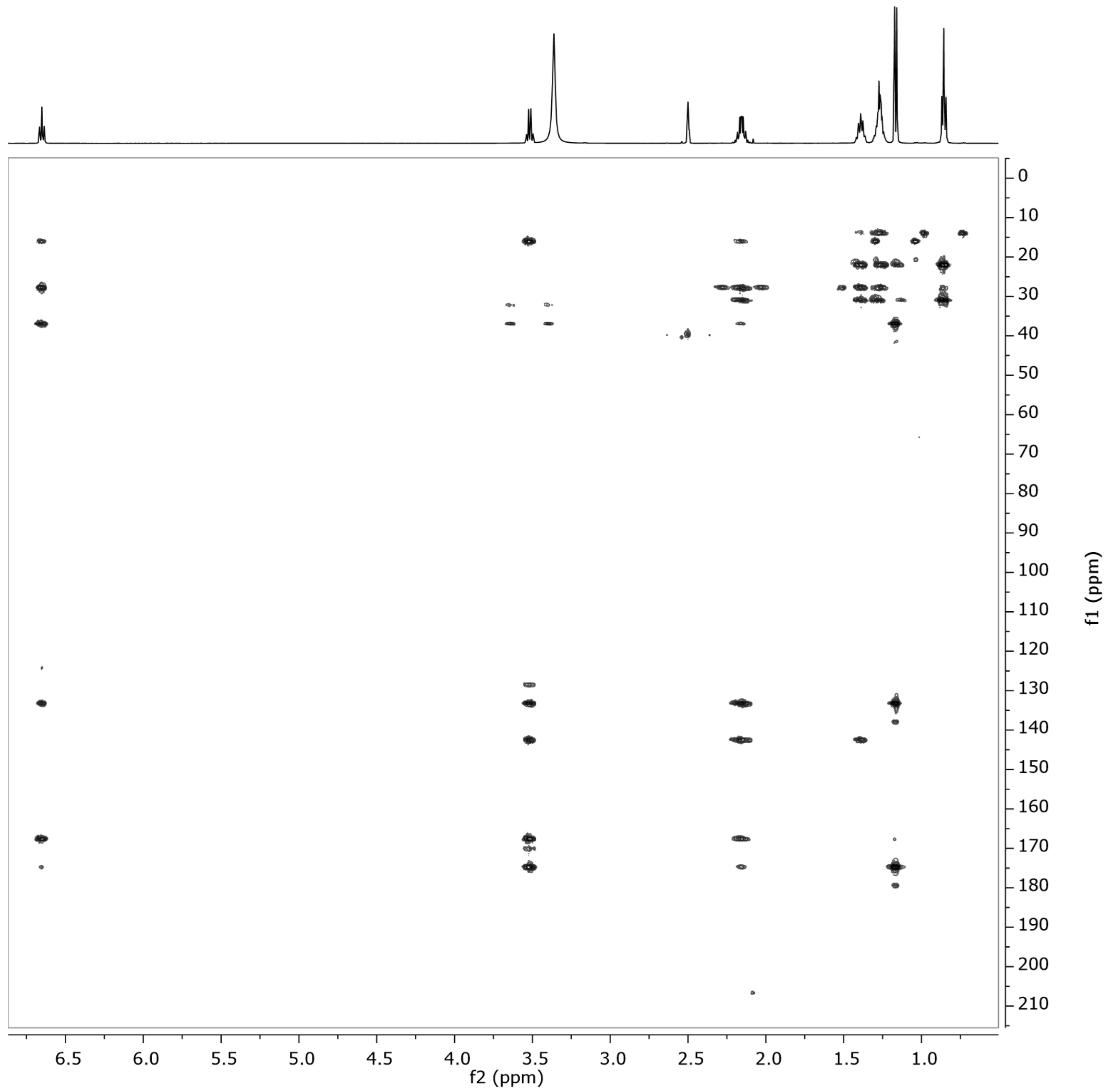

Figure S35. HMBC spectrum of **7** in DMSO-d_6 at 500 MHz.

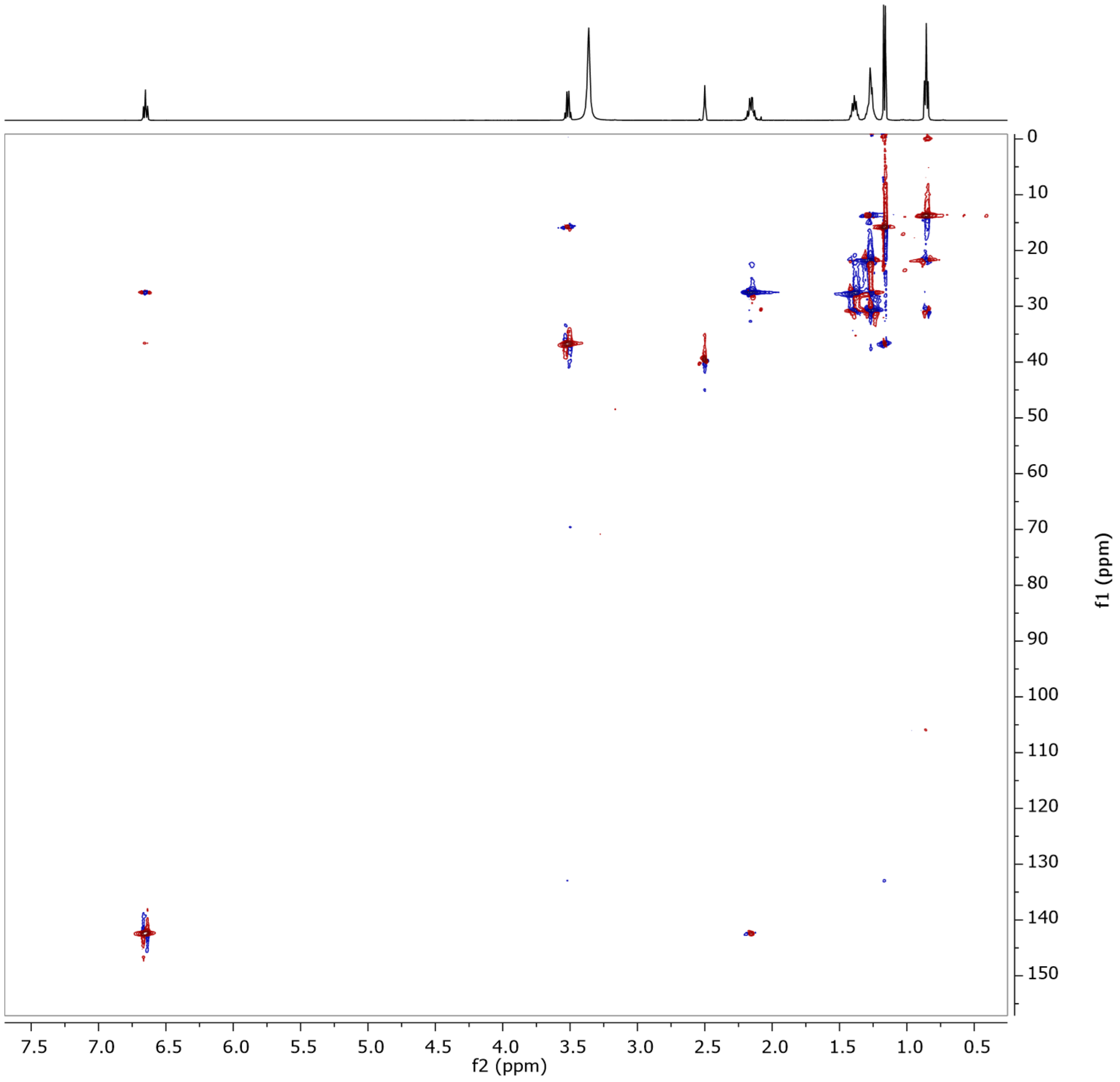

Figure S36. HSQC spectrum of **7** in DMSO-d_6 at 500 MHz.

Table S12. ^{1}H and ^{13}C NMR data of compound **7** and 2-hexylidene-3-methyl succinic acid.

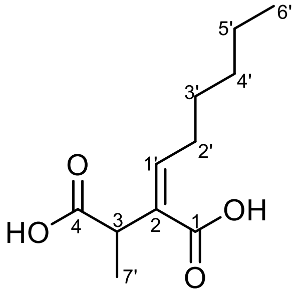

2-Hexylidene-3-methyl succinic acid (**7**)

	Compound **7**		2-Hexylidene-3-methyl succinic acid	
pos.	δ_C,[a] type	δ_H[b] multi (*J* in Hz)	δ_C,[c] type	δ_H[d] multi (*J* in Hz)
1		12.10 br s (O*H*)	171.5, CO	
2			131.5, C	
3	36.6, CH	3.52 q (7.0 Hz)	37.4, CH	3.59 q (7.2)
4		12.10 br s (O*H*)	174.1, CO	
1'	142.2, CH	6.65 t (7.7)	146.5, CH	6.99 t (7.5)
2'	27.4, CH_2	2.16 qd (7.4, 3.8)	28.7, CH_2	2.21 m
3'	27.6, CH_2	1.39 p (7.3)	28.1, CH_2	1.48 m
4'	30.7, CH_3	1.27 qt (6.7, 3.7)	31.5, CH_2	1.31 m
5'	21.6, CH_2	1.27 qt (6.7, 3.7)	22.4, CH_2	1.31 m
6'	13.6, CH_3	0.86 t (6.8)	13.9, CH_3	0.90 t (6.6)
7'	15.7, CH_3	1.17 d (7.0)	15.7, CH_3	1.35 d (7.2)

Measured in DMSO-d_6 at [a] 125 and [b] 500 MHz.
Measured in chloroform-*d* at [c] 100 and [d] 400 MHz.

Generic Display Report

Analysis Info

Analysis Name	S:\DATA\AmaZon\dva23_Daniela Valencia Revelo\Gymnopus montagnei\Gymnopus YM Sup fractions\Gymnopus YM Sup\Gymnopus YM Sup R1F12_BB4_01_48707.d	Acquisition Date	09.07.2023 01:44:23
Method	48707.m	Operator	tti
Sample Name	Gymnopus YM Sup R1F12	Instrument	amaZon speed
Comment			

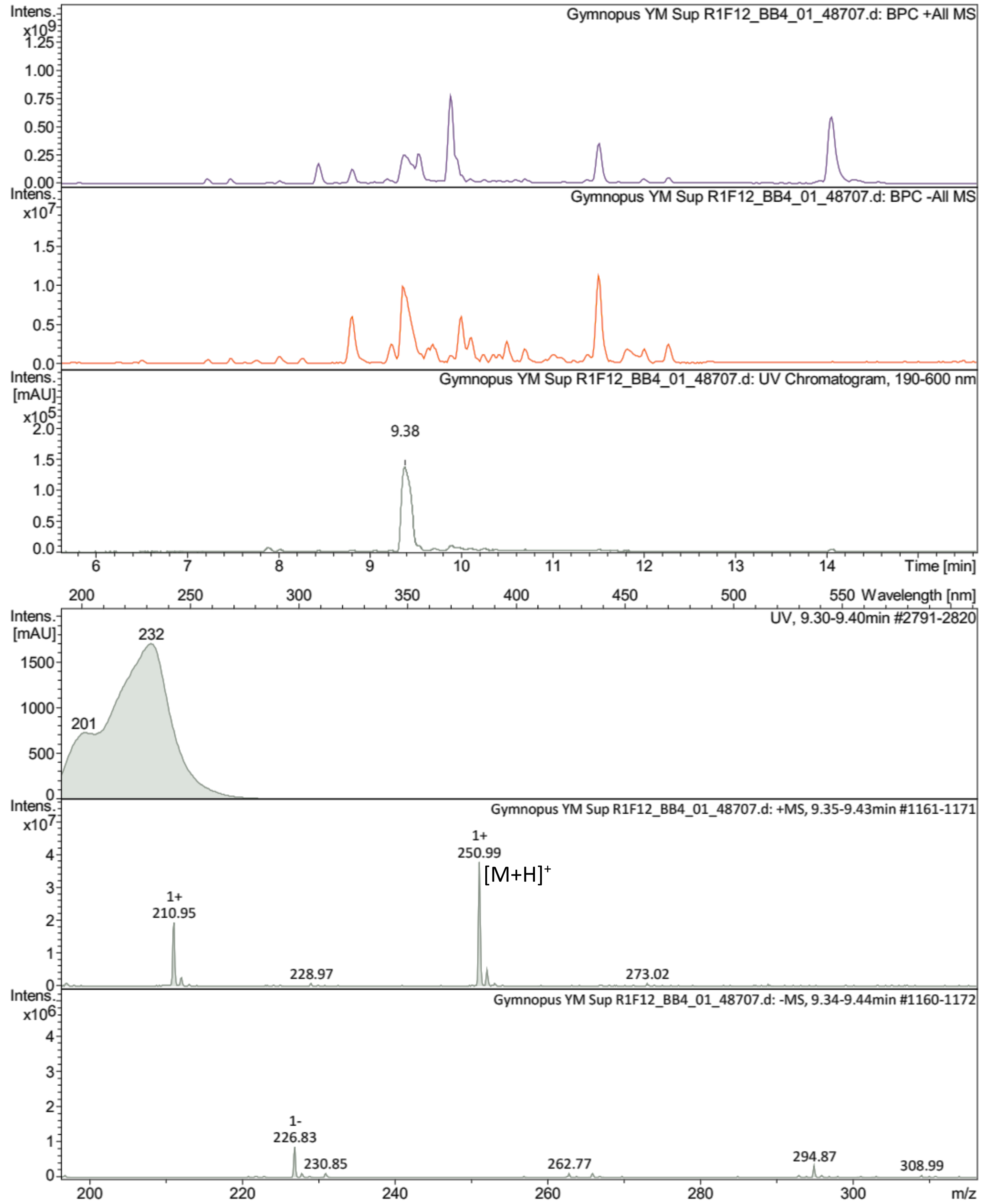

Bruker Compass DataAnalysis 4.4 printed: 18.07.2023 10:28:18 by: sel22 Page 1 of 1

Figure S37. LR-ESI-MS of **8**.

Generic Display Report

Analysis Info

		Acquisition Date	14.07.2023 23:23:46
Analysis Name	S:\DATA\MaXis\dva23_Daniela Valencia Revelo\23_07\Gymnopus YM Sup\Gymnopus YM Sup_R1_F12_28_01_12423.d		
Method	pos_saure_1[illegible]0_screening_ms_100_2500_line.m	Operator	ate06
Sample Name	Gymnopus YM Sup_R1_F12	Instrument	maXis
Comment	Screening01<br>Waters Acquity UPLC BEH C_18 1,7um 2.1x50mm		

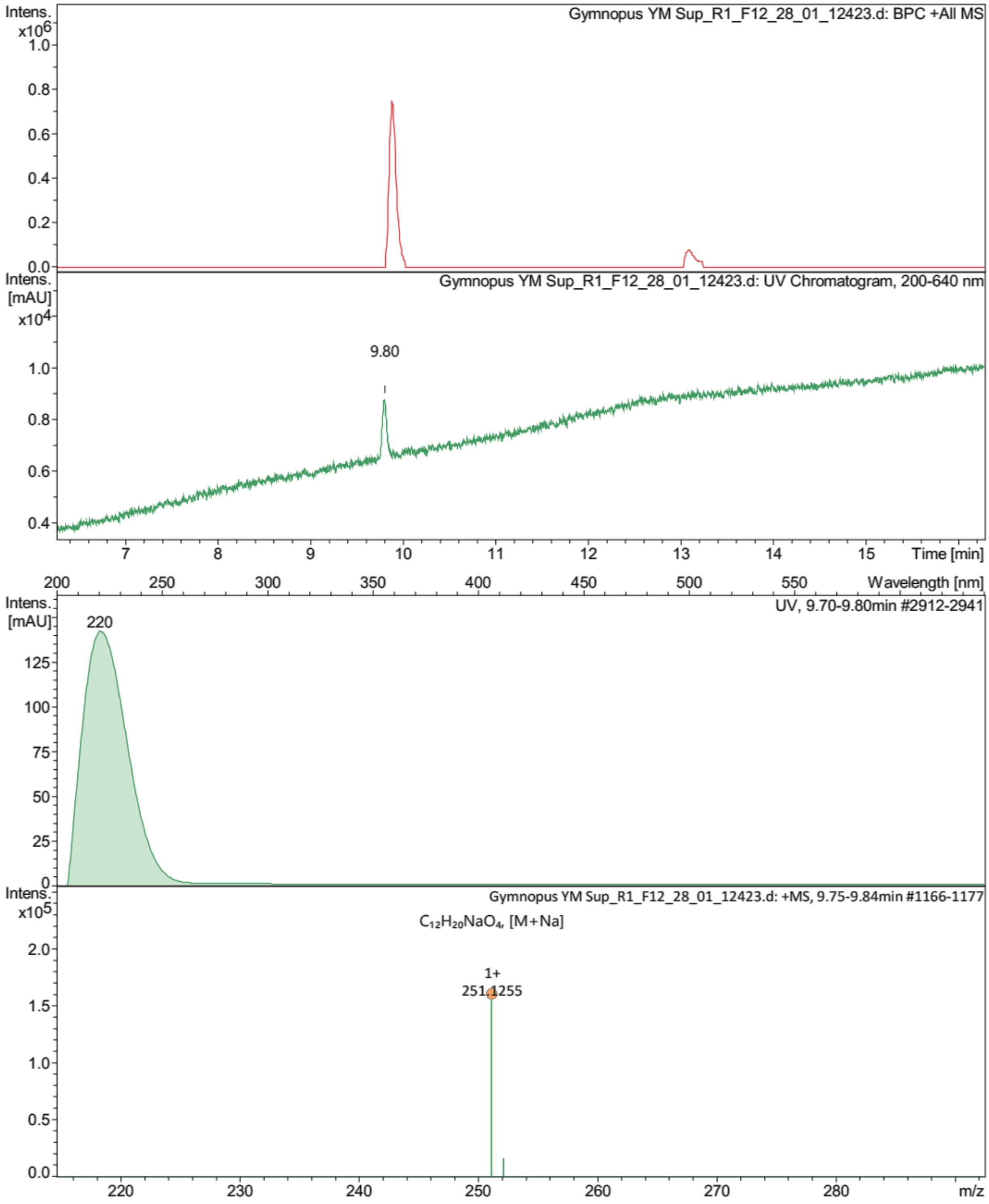

Figure S38. HR-ESI-MS of **8**.

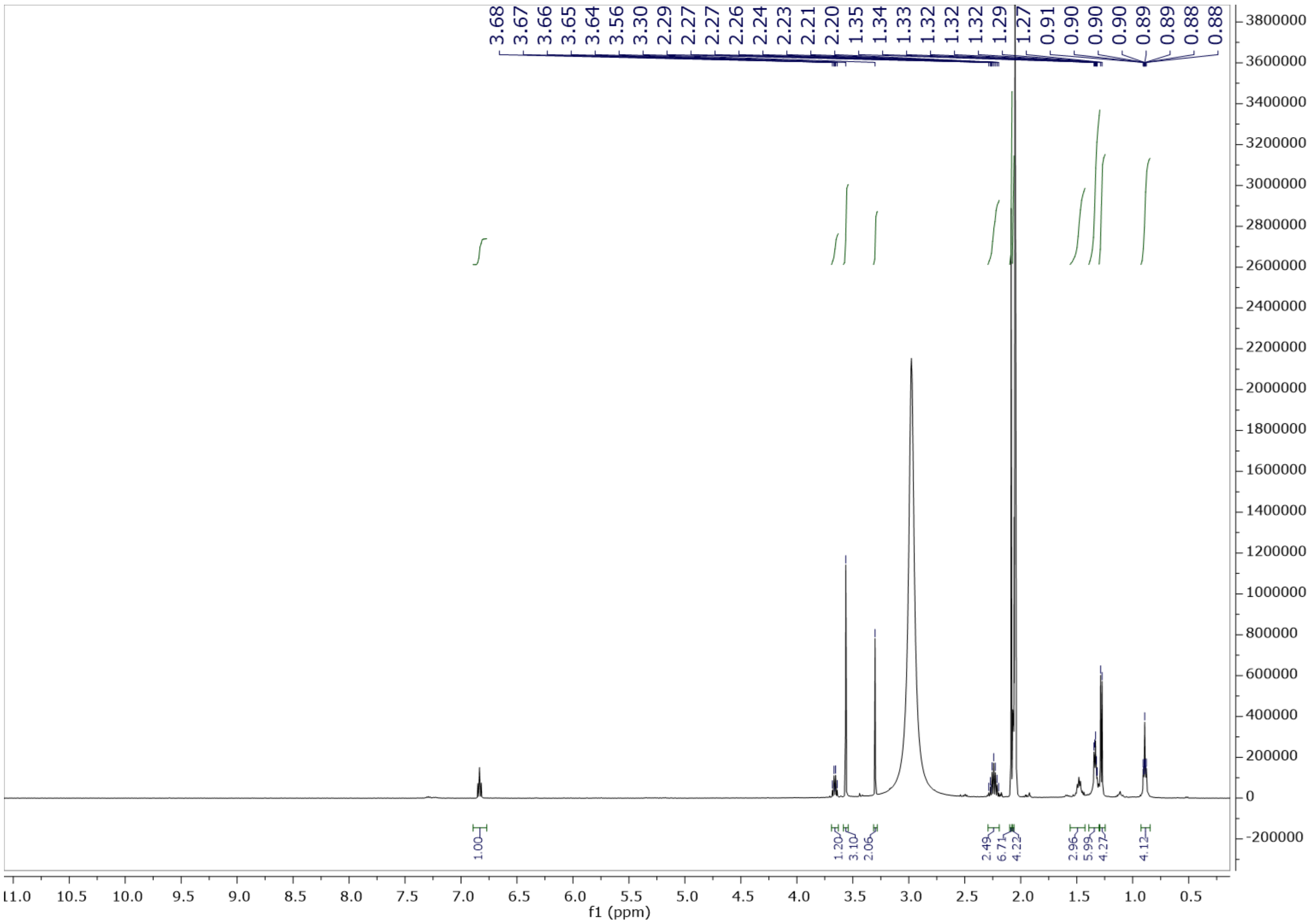

Figure S39. ^{1}H NMR spectrum of **8** in DMSO-d_6 at 500 MHz.

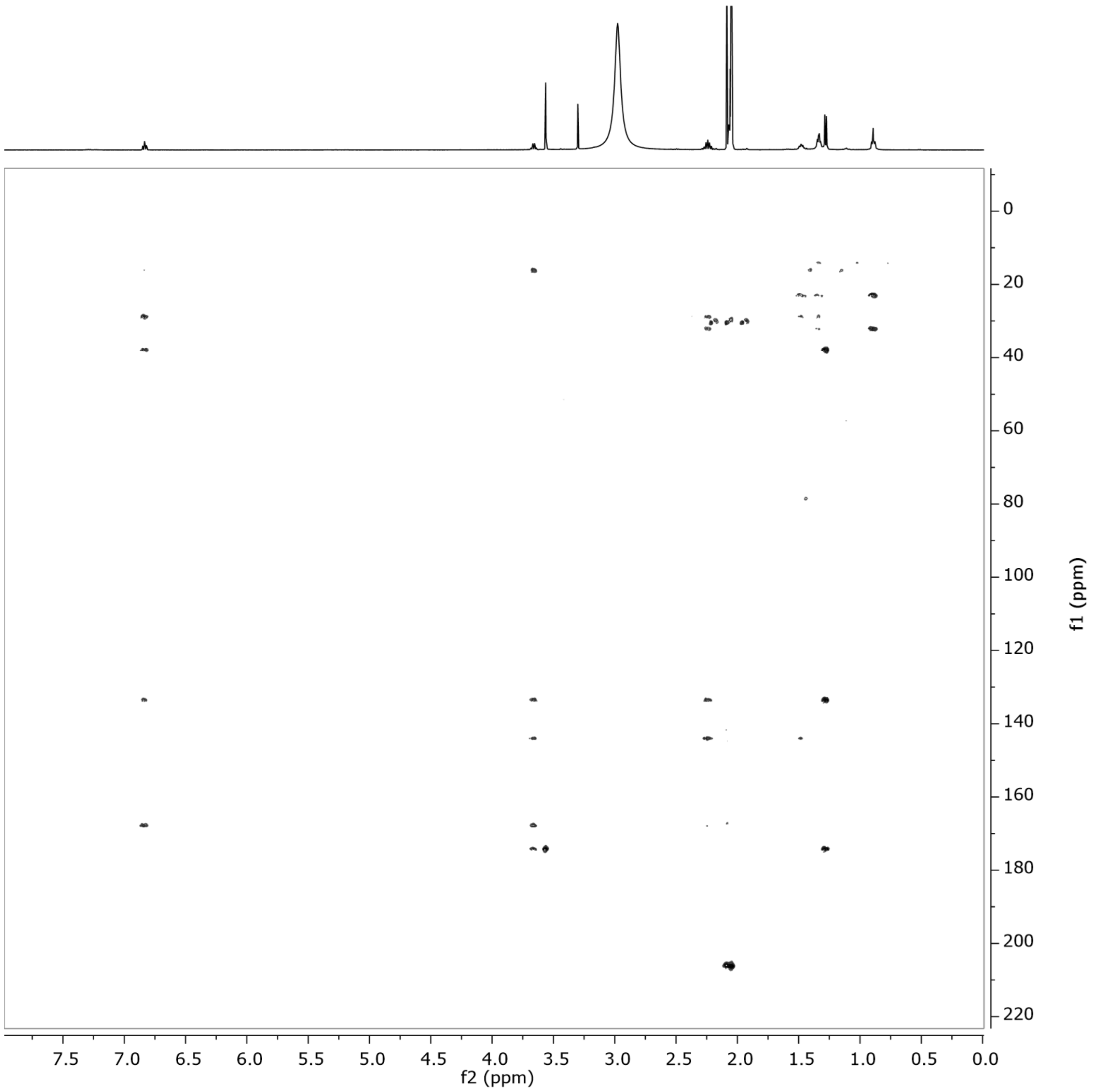

Figure S40. HMBC spectrum of **8** in DMSO-d_6 at 500 MHz.

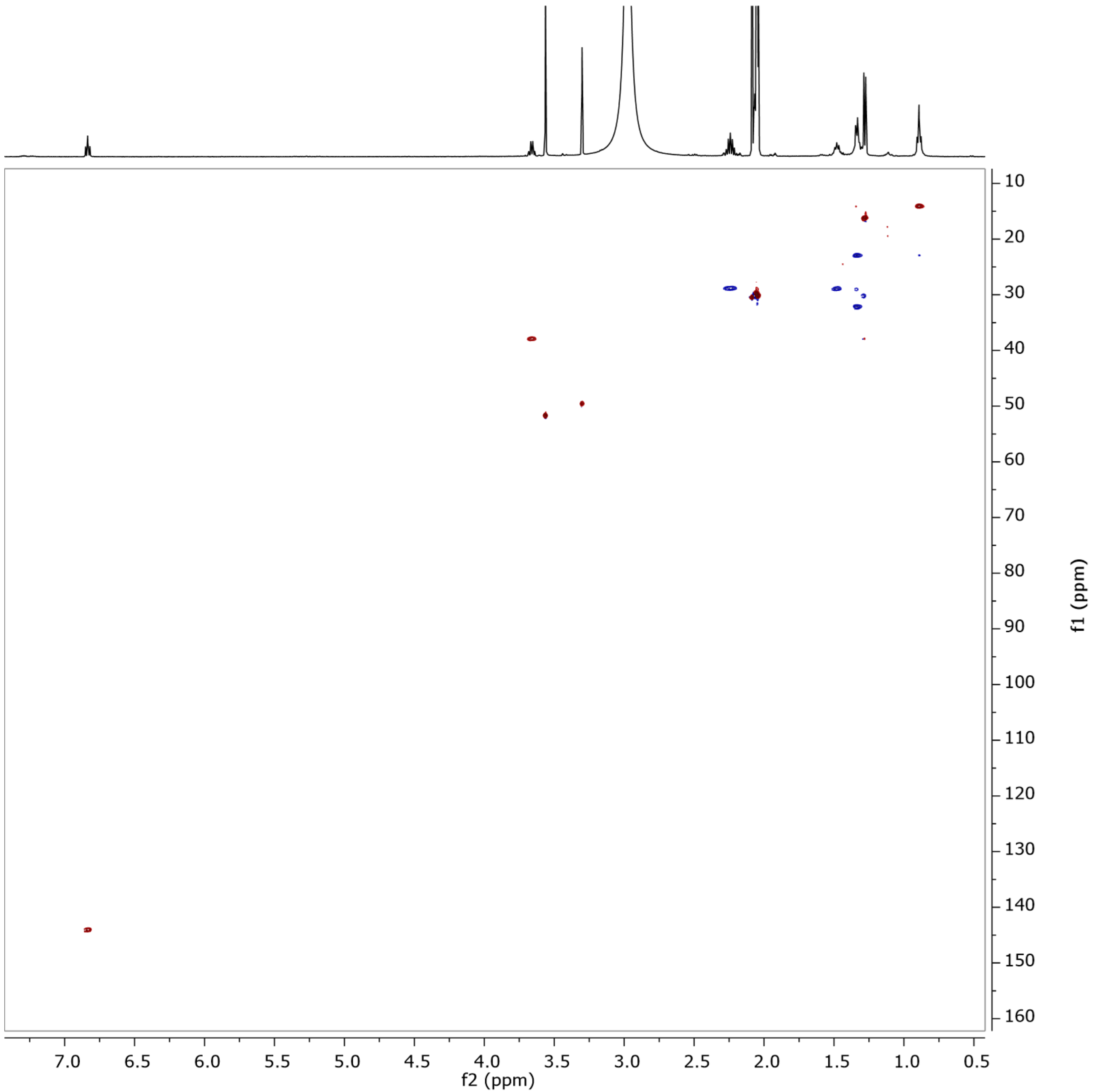

Figure S41. HSQC spectrum of **8** in DMSO-d_6 at 500 MHz.

Table S13. ^{1}H and ^{13}C NMR data of compound **8** and 2-hexylidene-3-methyl succinic acid methyl ester.

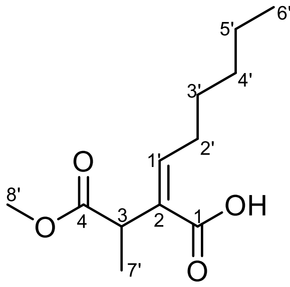

2-Hexylidene-3-methyl
succinic acid 4-methyl ester (**8**)

	Compound **8**		2-Hexylidene-3-methyl succinic acid methyl ester	
pos.	δ_C,[a] type	δ_H[b] multi (J in Hz)	δ_C,[c] type	δ_H[d] multi (J in Hz)
1	167.7, CO		171.5, CO	
2	133.5, C		131.5, C	
3	37.8, CH	3.66 q (7.1)	37.4, CH	3.59 q (7.2)
4	174.2, CO		174.1, CO	
1'	143.9, CH	6.83 t (7.7)	146.5, CH	6.99 t (7.5)
2'	28.7, CH_2	2.24 hept (7.3)	28.7, CH_2	2.21 m
3'	28.8, CH_2	1.42–1.53 m	28.1, CH_2	1.48 m
4'	32.0, CH_2	1.31–1.37 m	31.5, CH_2	1.31 m
5'	22.8, CH_2	1.31–1.37 m	22.4, CH_2	1.31 m
6'	14.0, CH_3	0.89 d (7.1)	13.9, CH_3	0.90 t (6.6)
7'	16.0, CH_3	1.28 d (7.1)	15.7, CH_3	1.35 d (7.2)
8'	51.5, CH_3	3.56 s	52.0, CH_3	3.66 s

Measured in DMSO-d_6 at [a] 125 and [b] 500 MHz.
Measured in acetone-d_6 at [c] 125 and [d] 500 MHz.

Generic Display Report

Analysis Info

Analysis Name	S:\DATA\AmaZon\dva23_Daniela Valencia Revelo\Gymnopus montagnei\Gymnopus YM Sup fractions\Gymnopus YM Sup\Gymnopus YM Sup R1F13_BB5_01_48708.d	Acquisition Date	09.07.2023 02:20:37
Method	48708m	Operator	tti
Sample Name	Gymnopus YM Sup R1F13	Instrument	amaZon speed
Comment			

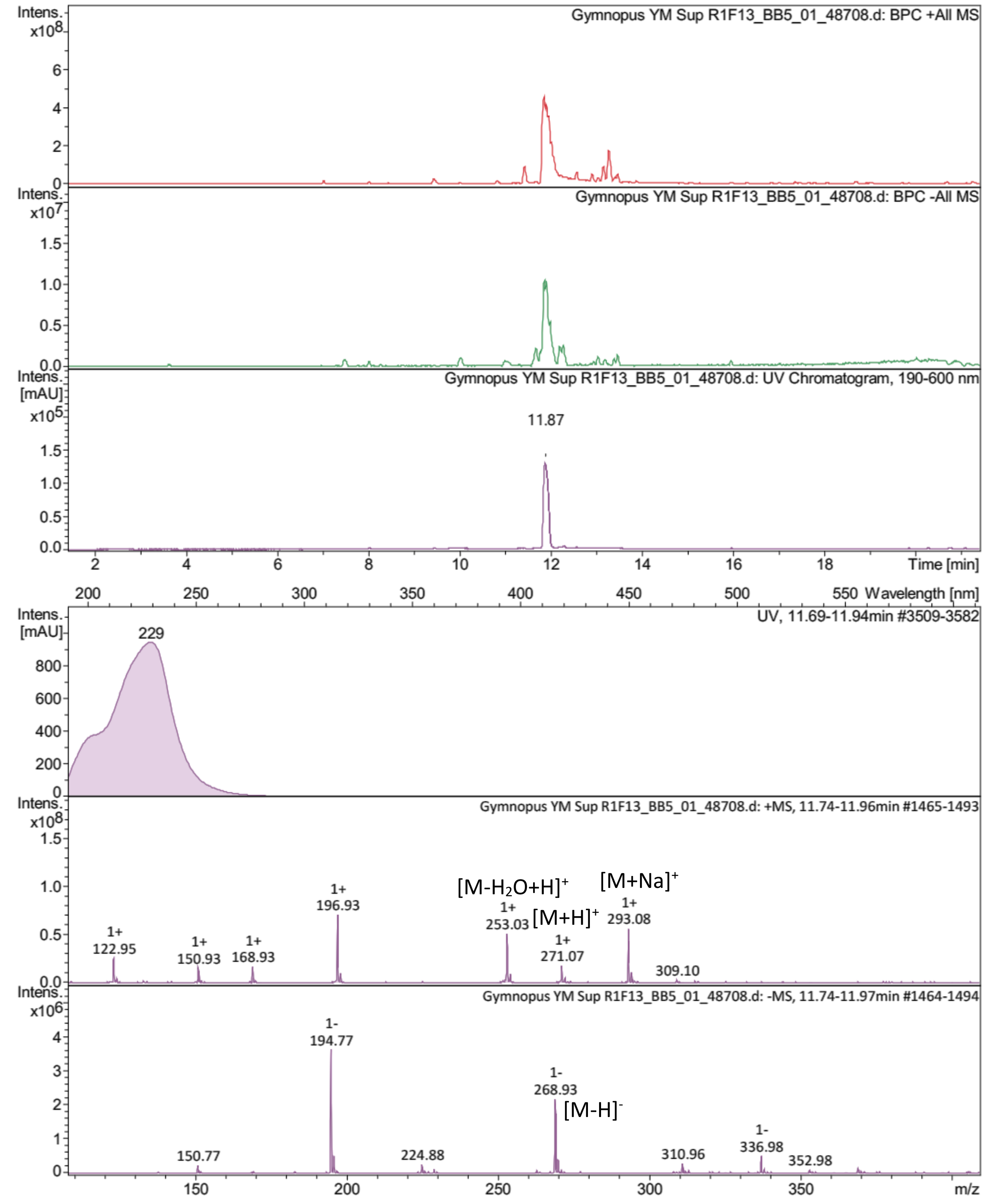

Bruker Compass DataAnalysis 4.4 printed: 18.07.2023 10:17:33 by: sel22 Page 1 of 1

Figure S42. LR-ESI-MS of **9**.

Generic Display Report

Analysis Info

Analysis Name	S:\DATA\MaXis\dva23_Daniela Valencia Revelo\23_07\23_07_14\Gymnopus YM Sup_R1_F13_29_01_12424.d	Acquisition Date	14.07.2023 23:54:44
Method	[illegible]ms_100_2500_line.m	Operator	ate06
Sample Name	Gymnopus YM Sup_R1_F13	Instrument	maXis
Comment	Screening01<br>Waters Acquity UPLC BEH C_18 1,7um 2.1x50mm		

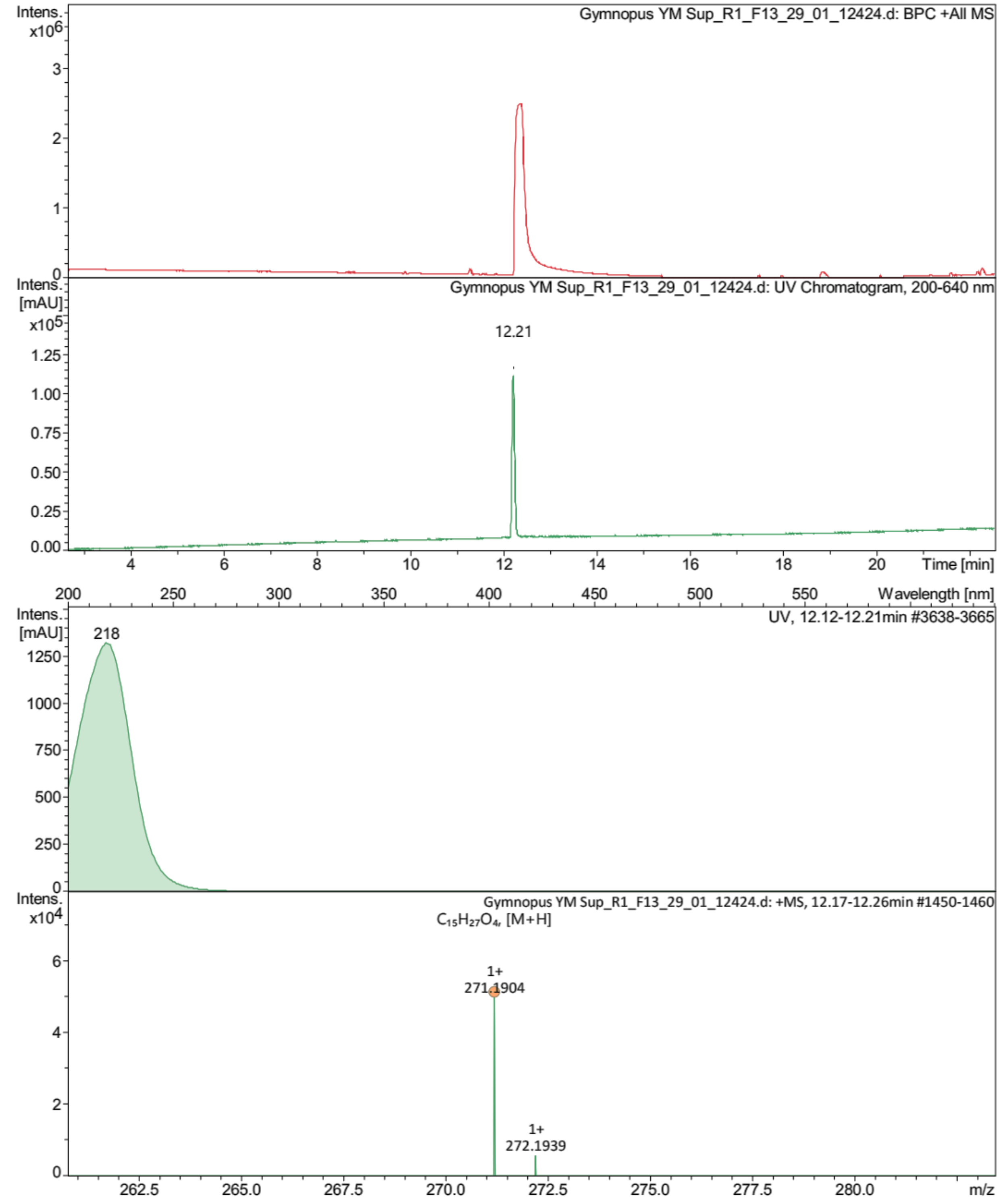

Bruker Compass DataAnalysis 4.4 printed: 18.07.2023 09:56:18 by: sel22 Page 1 of 1

Figure S43. HR-ESI-MS of **9**.

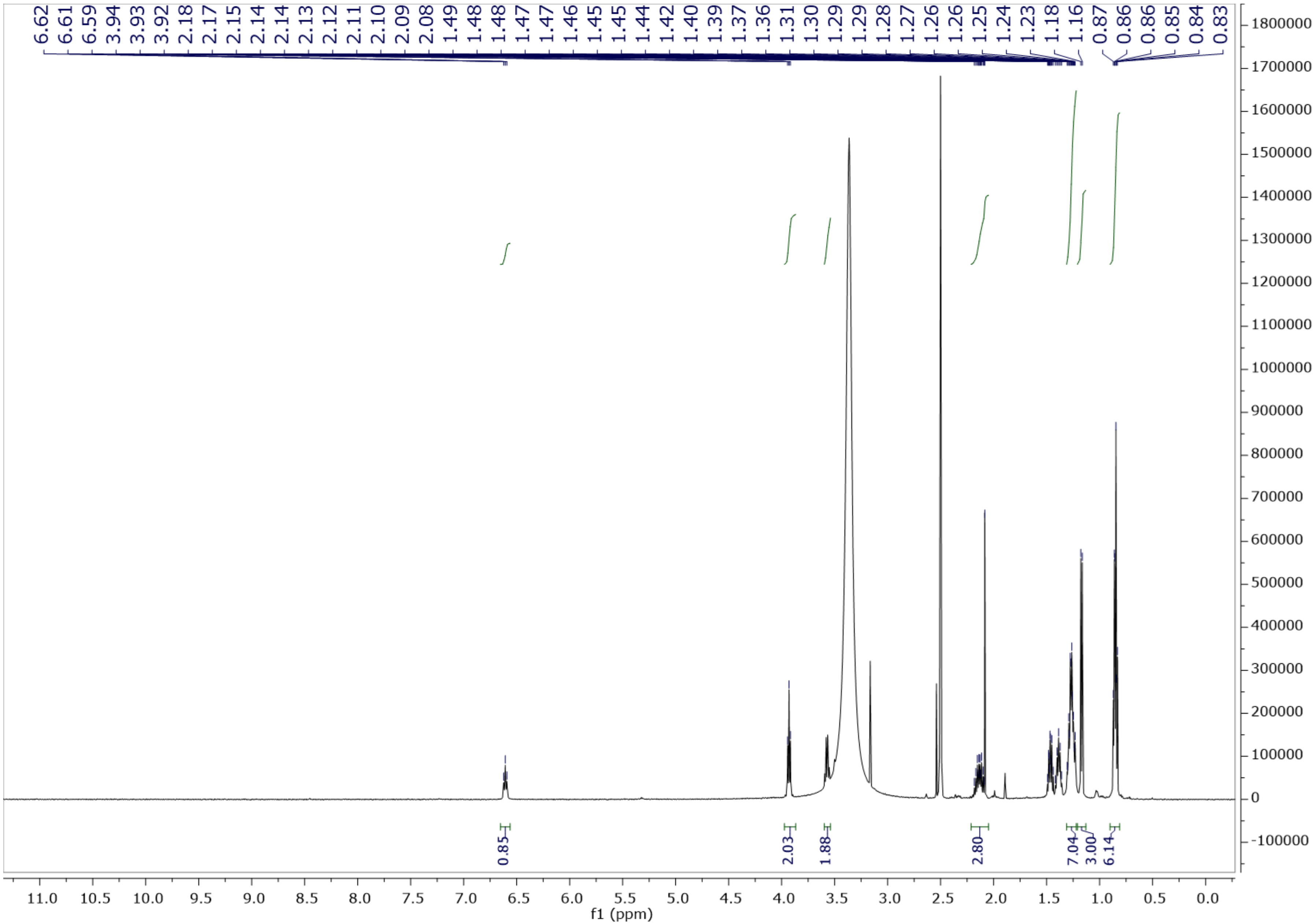

Figure S44. ^{1}H NMR spectrum of **9** in DMSO-d_6 at 500 MHz.

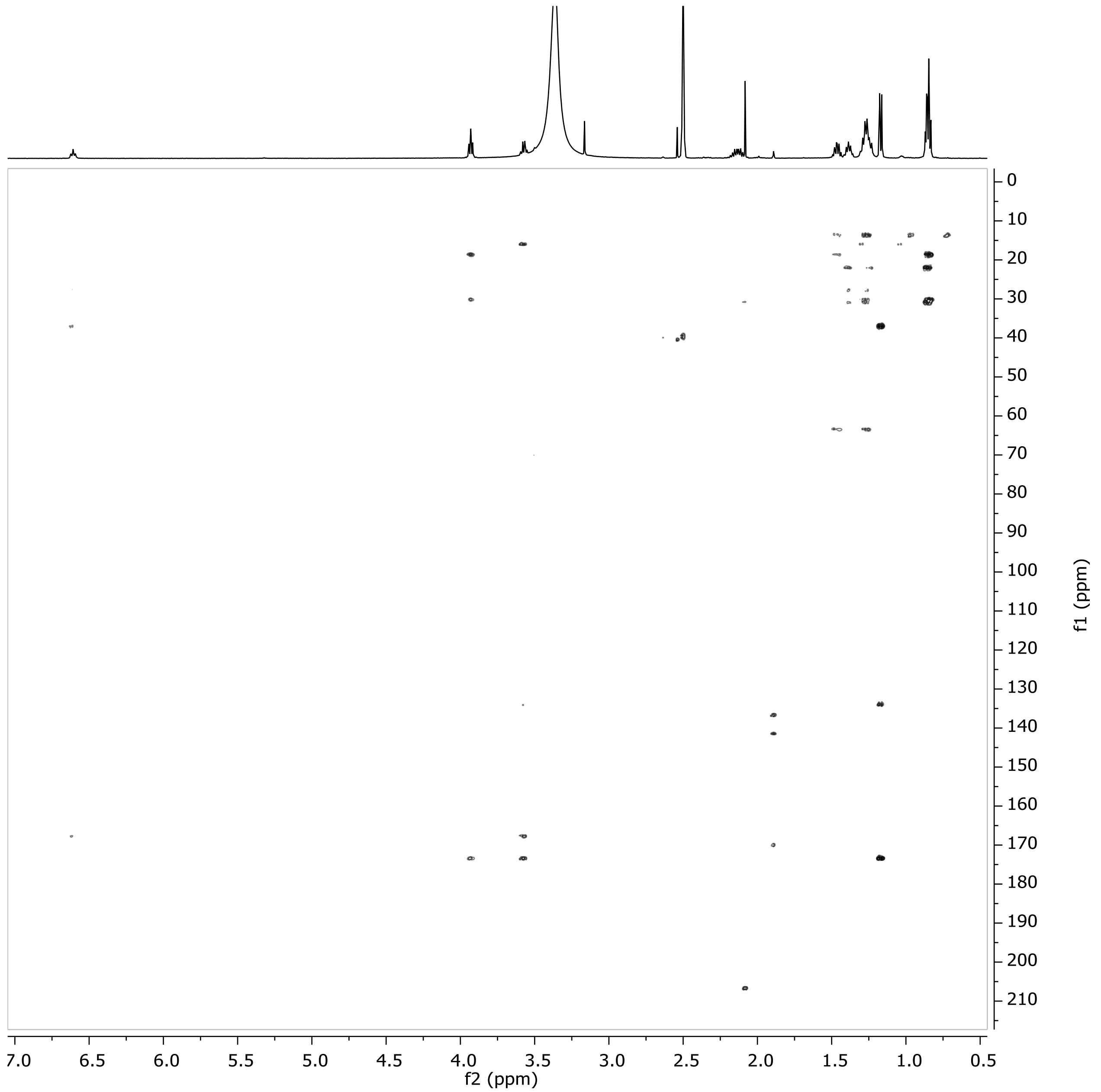

Figure S45. HMBC spectrum of **9** in DMSO-d_6 at 500 MHz.

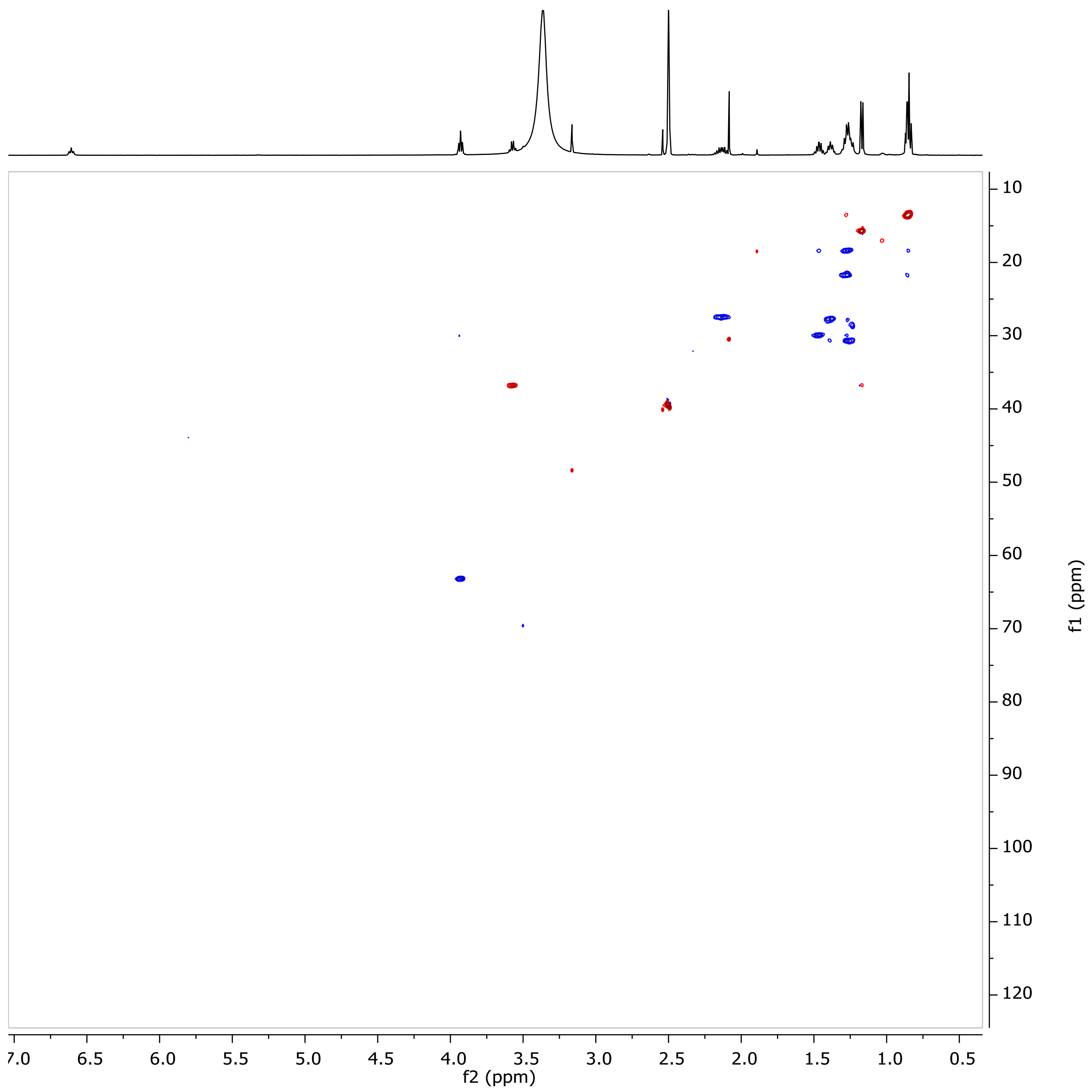

Figure S46. HSQC spectrum of **9** in DMSO-d_6 at 500 MHz.

Table S14. ^{1}H and ^{13}C NMR data of compound **9** and akoenic acid.

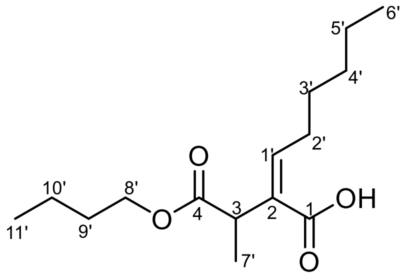

Akoenic acid (**9**)

	Compound **9**		Akoenic acid	
pos.	δ_C,[a] type	δ_H[b] multi (*J* in Hz)	δ_C,[c] type	δ_H[d] multi (*J* in Hz)
1	167.6, CO		171.0, CO	
2	133.9, C		131.5, C	
3	36.7, CH	3.57 q (7.0)	37.5, CH	3.58 q (7.2)
4	173.3, CO		173.7, CO	
1'	n.d.	6.61 t (7.6)	146.5, CH	6.96 t (7.6)
2'	27.4, CH_2	2.03–2.22 m	28.7, CH_2	2.13-2.29 m
3'	27.7, CH_2	1.39 p (7.3)	28.2, CH_2	1.42-1.54 m
4'	30.7, CH_2	1.21–1.31 m	31.5, CH_2	1.29-1.33 m
5'	21.7, CH_2	1.21–1.31 m	22.4, CH_2	1.30-1.34 m
6'	13.6, CH_3	0.86 t (6.9)	13.9, CH_3	0.89 t (7.2)
7'		1.17 d (6.9)	15.7, CH_3	1.33 d (7.2)
8'	63.0, CH_2	3.93 t (6.4)	64.7, CH_2	4.01-4.12 m
9'	29.9, CH_2	1.47 dq (8.9, 6.5, 5.8)	30.5, CH_2	1.49-1.63 m
10'	18.4, CH_2	1.21–1.31 m	19.1, CH_2	1.30-1.36 m
11'	13.4, CH_3	0.85 t (7.3)	13.6, CH_3	0.89 t (7.2)

Measured in DMSO-d_6 at [a] 125 and [b] 500 MHz.
Measured in chloroform-*d* at [c] 100 and [d] 400 MHz. n.d.: not determined.

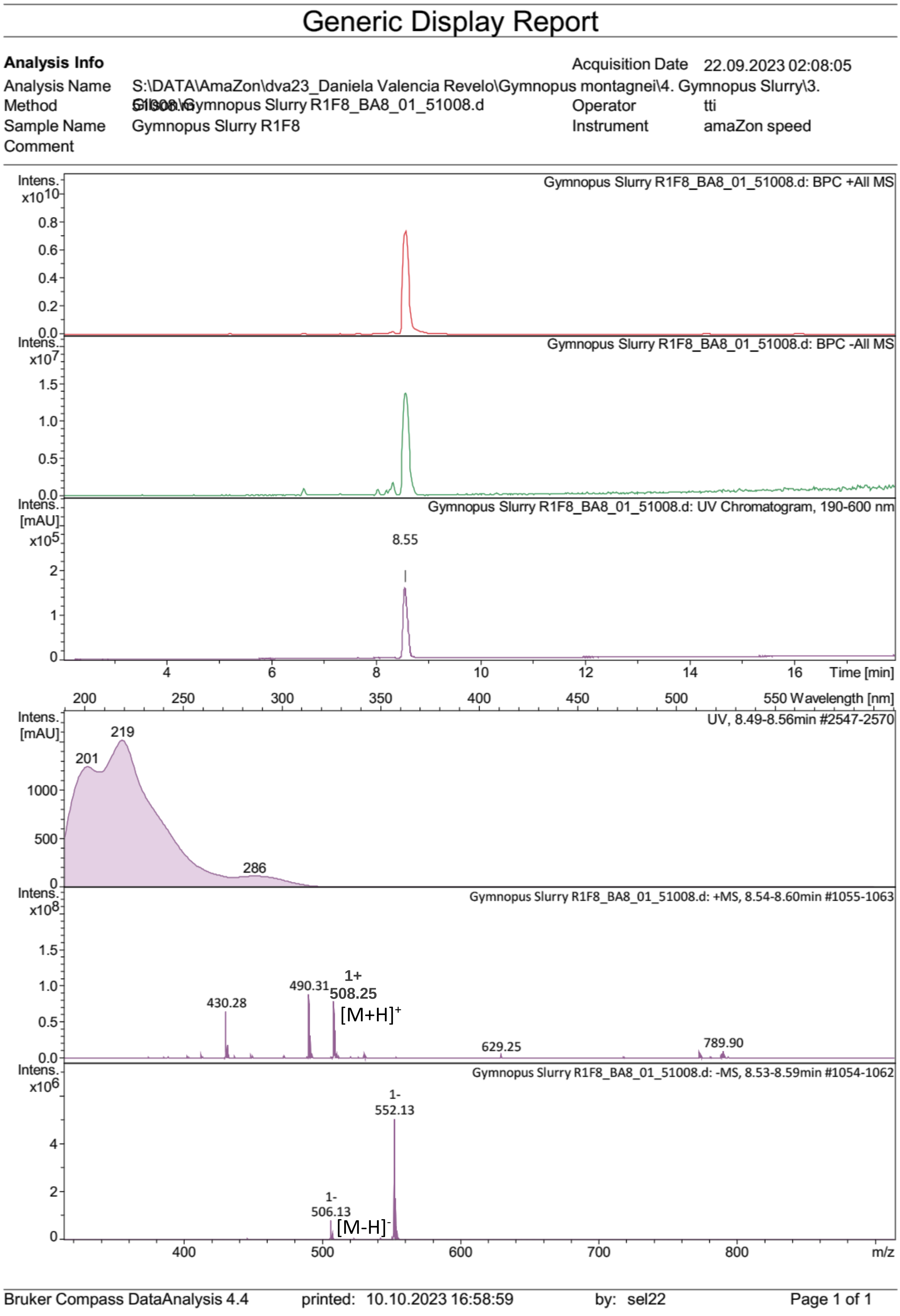

Figure S47. LR-ESI-MS of **10**.

Generic Display Report

**Analysis Info**		Acquisition Date	19.09.2023 17:06:30
Analysis Name	S:\DATA\MaXis\dva23_Daniela Valencia Revelo\23_09_19\Gymnopus Slurry_R1_F8_18_01_13211.d		
Method	pos_säure_10000_screening_ms_100_2500_line.m	Operator	ate06
Sample Name	Gymnopus Slurry_R1_F8	Instrument	maXis
Comment	Screening01<br>Waters Acquity UPLC BEH C_18 1,7um 2.1x50mm		

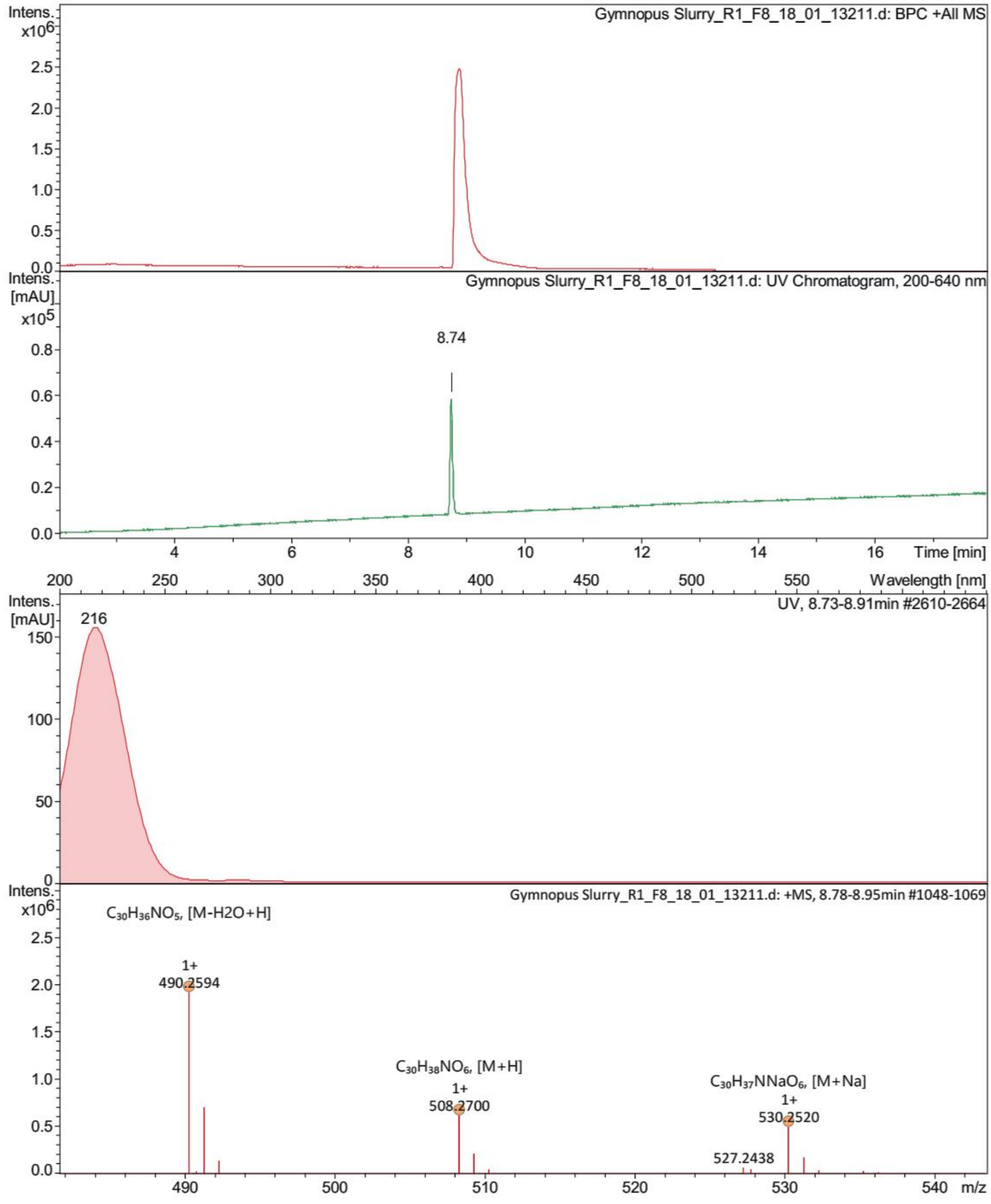

Bruker Compass DataAnalysis 4.4 printed: 10.10.2023 16:57:42 by: sel22 Page 1 of 1

Figure S48. HR-ESI-MS of **10**.

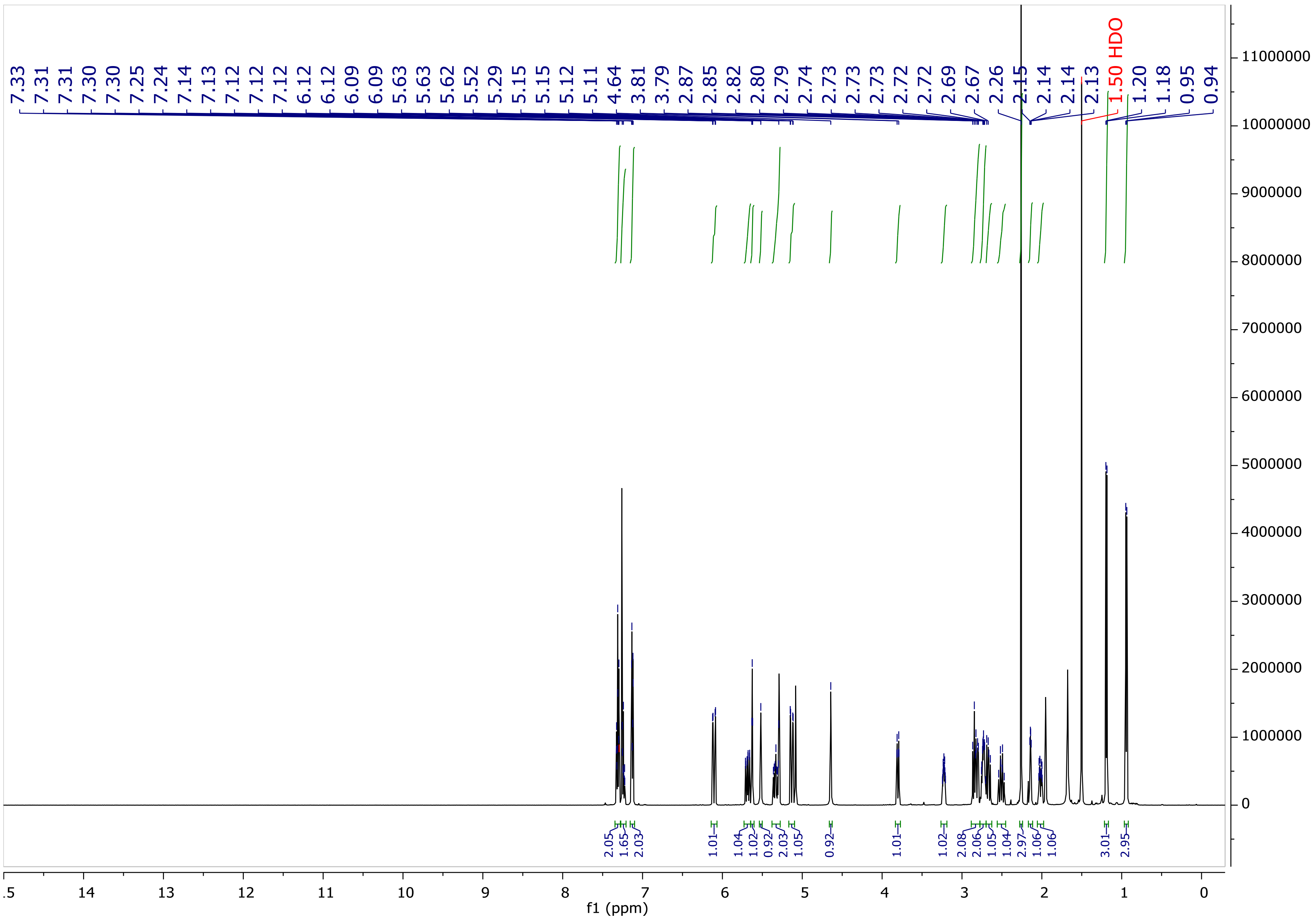

Figure S49. ^{1}H NMR spectrum of **10** in DMSO-d_6 at 500 MHz.

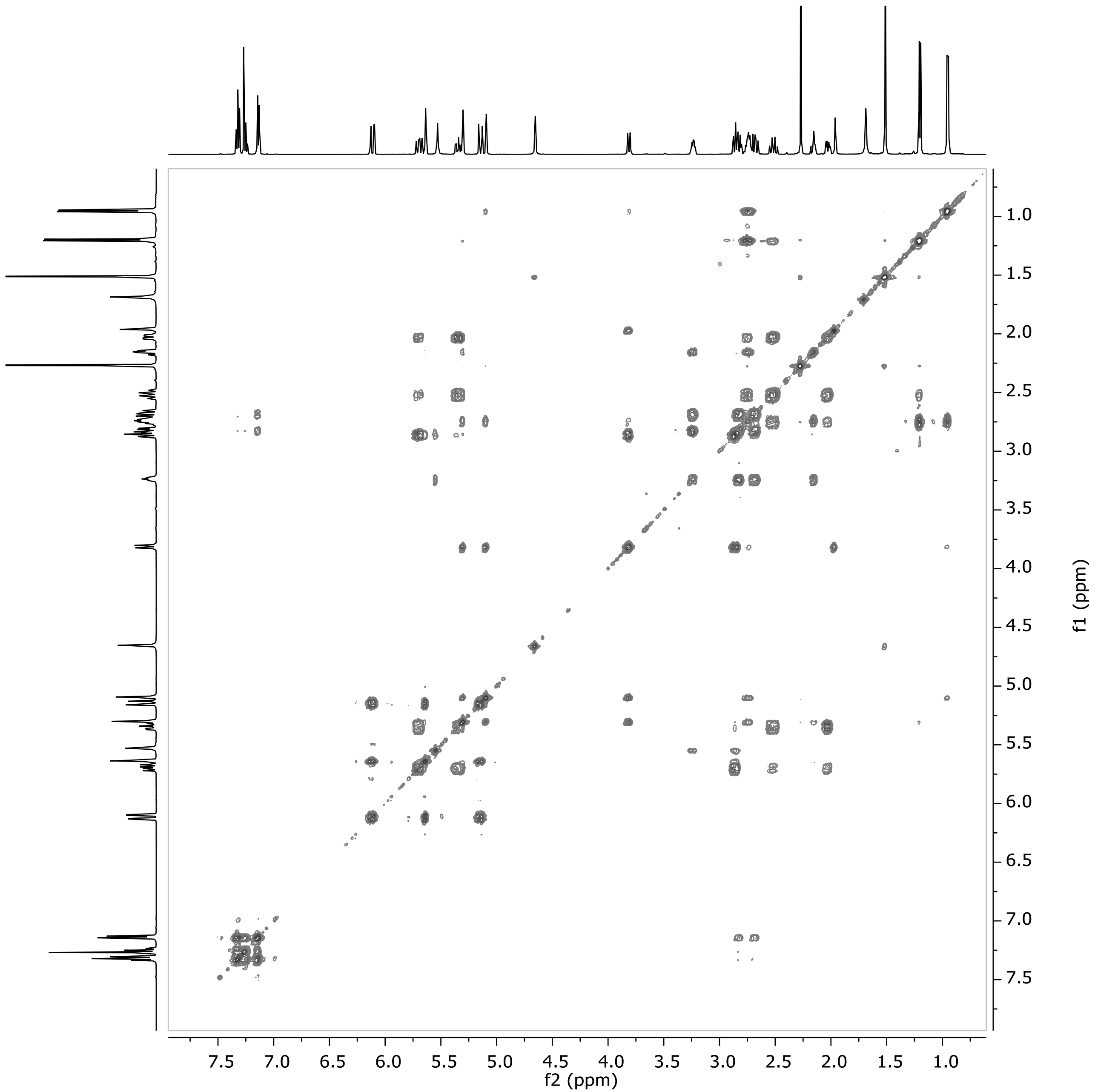

Figure S50. ^{1}H-^{1}H COSY spectrum of **10** in DMSO-d_6 at 500 MHz.

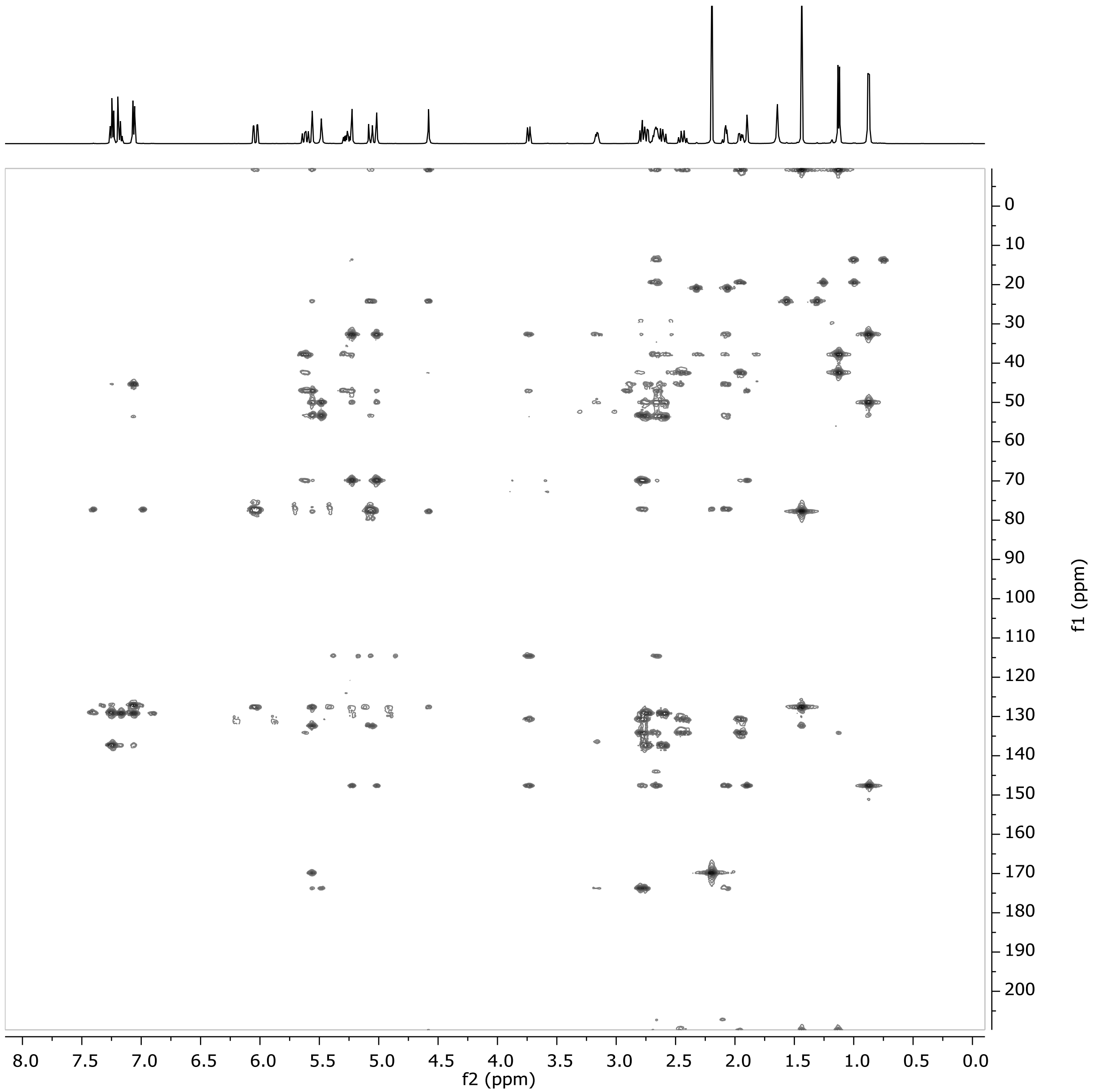

Figure S51. HMBC spectrum of **10** in DMSO-d_6 at 500 MHz.

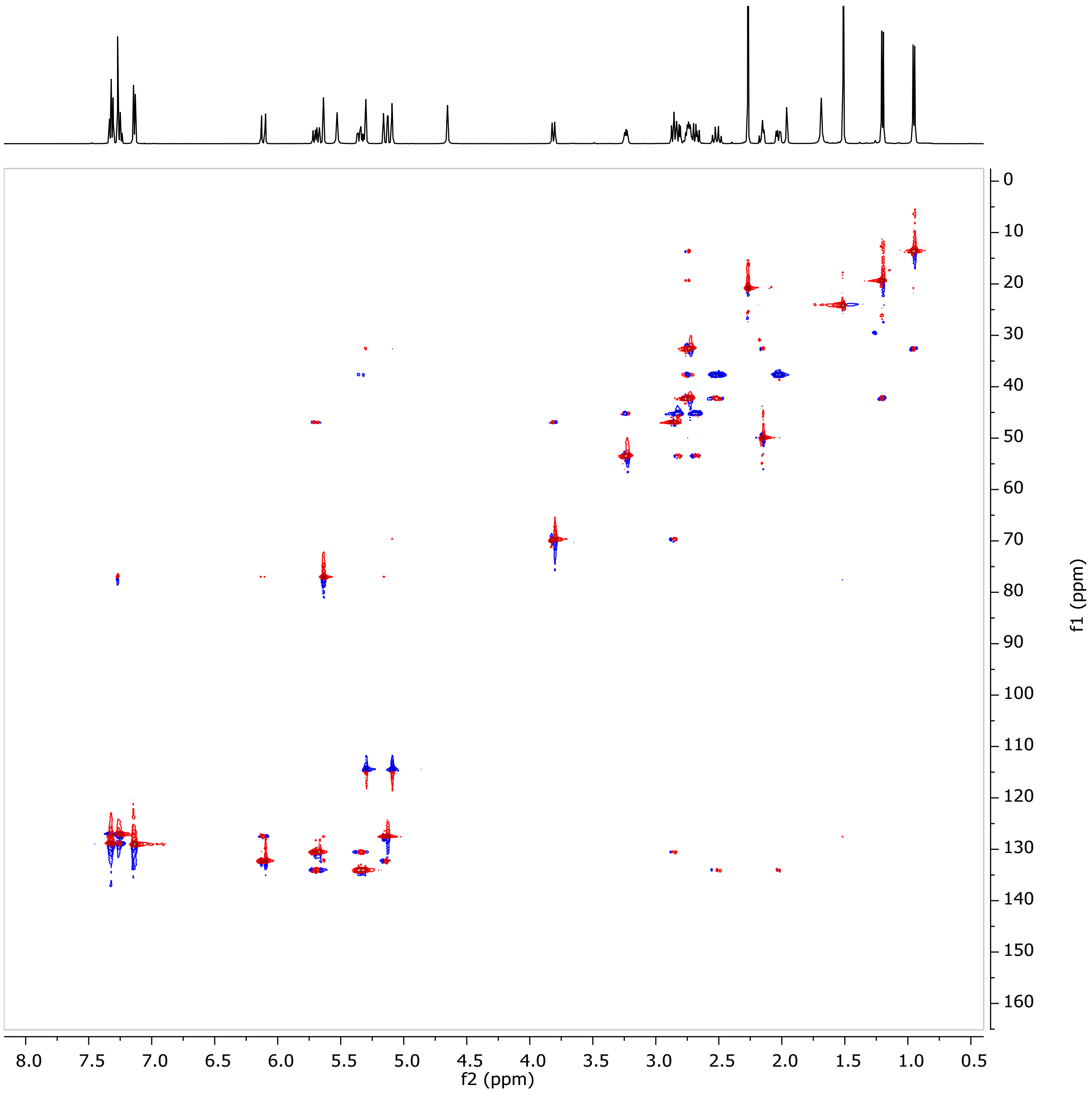

Figure S52. HSQC spectrum of **10** in DMSO-d_6 at 500 MHz.

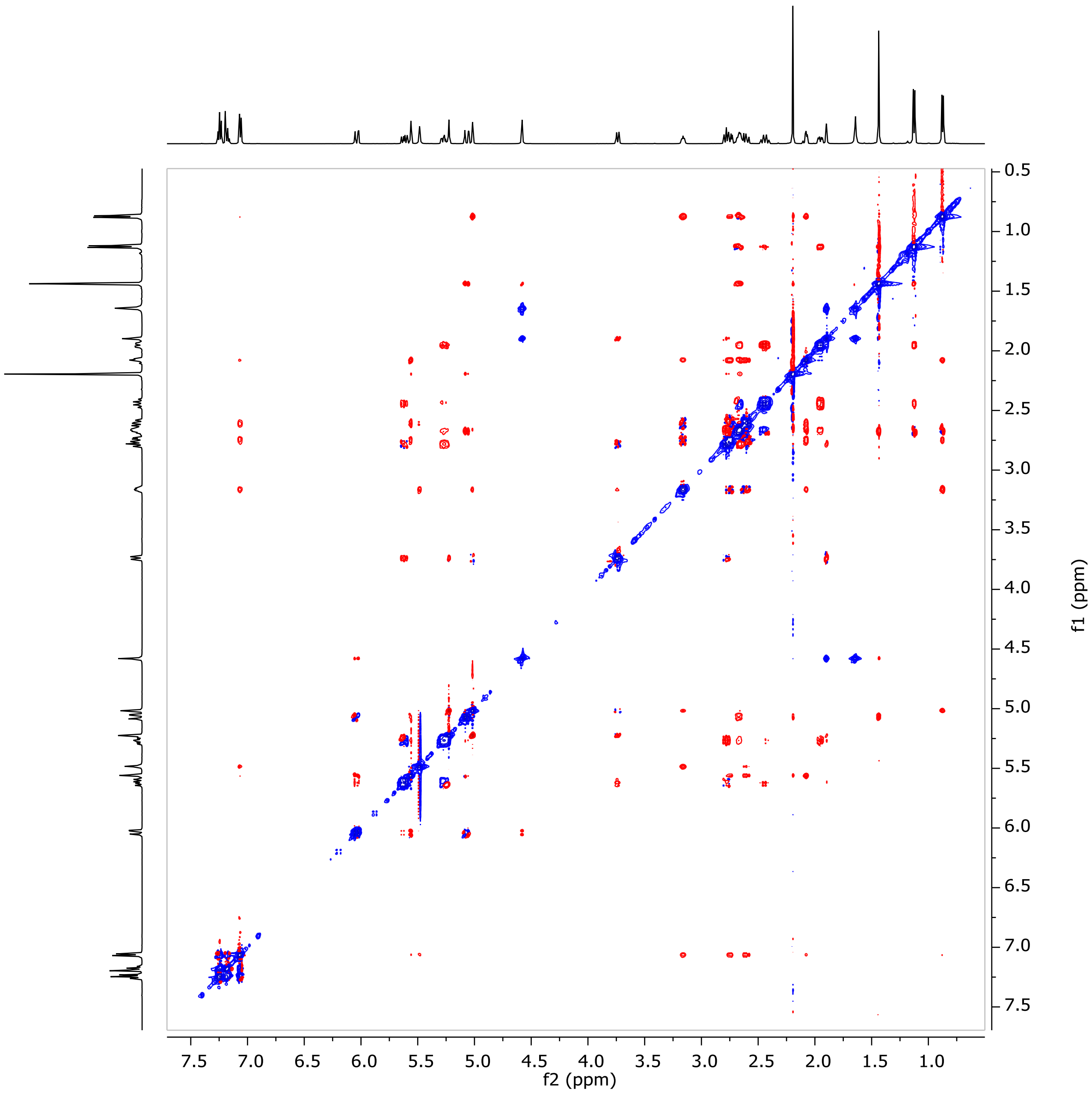

Figure S53. ROESY spectrum of **10** in DMSO-d_6 at 500 MHz.

Table S15. ^{1}H and ^{13}C NMR data of compound **10** and cytochalasin D.

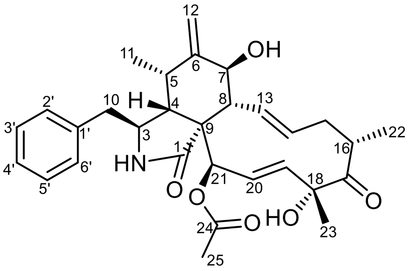

Cytochalasin D

	Compound **10**		Cytochalasin D	
pos.	δ_C,[a] type	δ_H[b] multi (*J* in Hz)	δ_C,[c] type	δ_H[d] multi (*J* in Hz)
1	173.7, CO	-	173.8, CO	-
2-N*H*	-	5.52 s	-	5.43 s
3	53.6, CH	3.23 dddd (8.3, 4.9, 3.4, 1.3)	53.4, CH	3.23 m
4	50.1, CH	2.14 dd (5.2, 3.4)	50.2, CH	2.14 dd (4.4, 3.9)
5	32.7, CH	2.73 m	32.8, CH	2.72 m
6	147.4, C	-	147.7, C	-
7	69.9, CH	3.80 dd (10.6, 1.4)	70.0, CH	3.81 d (10.4)
7-O*H*	-	1.95 br d (1.7)	-	1.91 s
8	47.0, CH	2.85 t (10.3, 10.1)	47.1, CH	2.85 t (10.4, 9.1)
9	53.1, C	-	53.7, C	-
10	45.4, CH_2	α 2.67 dd (13.4, 9.4)<br>β 2.81 dd (13.4, 4.9)	45.5, CH_2	α 2.67 dd (13.3, 9.1)<br>β 2.82 dd (13.6, 5.2)
11	13.8, CH_3	0.94 d (6.7)	13.8, CH_3	0.96 d (6.5)
12	114.6, CH_2	α 5.08 s<br>β 5.29 s	114.7, CH_2	α 5.09 s<br>β 5.29 s
13	127.7, CH	5.13 dd (15.7, 2.3)	127.8, CH	5.14 dd (15.6, 2.6)
14	130.7, CH	5.68 ddd (15.4, 9.8, 1.1)	130.8, CH	5.69 dd (15.6, 9.8)
15	37.9, CH_2	α 2.02 ddt (13.0, 5.2, 1.5)<br>β 2.51 dt (13.0, 10.9)	37.9, CH_2	α 2.02 dd (12.9, 5.2)<br>β 2.51 dd (12.9, 11.0)
16	42.4, CH	2.74 m	42.5, CH	2.74 m
17	210.2, CO	-	210.4, CO	-
18	77.6, C	-	77.8, C	-
18-O*H*	-	4.64 s	-	4.65 s
19	134.3, CH	5.33 ddd (15.8, 10.8, 5.3)	134.3, CH	5.34 ddd (15.5, 10.5, 5.2)
20	132.4, CH	6.10 dd (15.8, 2.7)	132.5, CH	6.11 dd (15.6, 2.6)
21	77.2, CH	5.63 t (2.5)	77.2, CH	5.63 t (2.6)
22	19.5, CH_3	1.19 d (6.8)	19.5, CH_3	1.19 d (7.2)
23	24.3, CH_3	1.50 s	24.3, CH_3	1.51 s
24	169.7, CO	-	169.9, CO	-
25	21.0, CH_3	2.26 s	21.0, CH_3	2.26 s
1'	137.1, C	-	137.4, C	-
2'	129.2, CH	7.13 d (7.0)	129.3, CH	7.12 d (7.1)
3'	129.0, CH	7.31 t (7.3)	129.1, CH	7.31 t (7.1)
4'	127.2, CH	7.24 t (7.4)	127.3, CH	7.25 t (7.8)
5'	129.0, CH	7.31 t (7.3)	129.1, CH	7.31 t (8.0)
6'	129.2, CH	7.13 d (7.0)	129.3, CH	7.12 d (8.0)

Measured in chloroform-*d* at [a] 150 and [b] 600 MHz; [a] 125 and [b] 500 MHz.

Display Report

Analysis Info

Acquisition Date 05.11.2023 00:12:38

Analysis Name	S:\DATA\AmaZon\dva23_Daniela Valencia Revelo\Gymnopus montagnei\4. Gymnopus Slurry\4. Semiprep\Semiprep 10\GymSlurry_SP10_R1F1(good)_RC3_01_52068.d		
Method	52068.m	Operator	tti
Sample Name	GymSlurry_SP10_R1F1(good)	Instrument	amaZon speed
Comment			

Acquisition Parameter

Ion Source Type	ESI	Ion Polarity	Negative	Alternating Ion Polarity	on
Mass Range Mode	UltraScan	Scan Begin	100 m/z	Scan End	2000 m/z
Accumulation Time	4000 µs	RF Level	100 %	Trap Drive	77.7
SPS Target Mass	1000 m/z	Averages	6 Spectra		

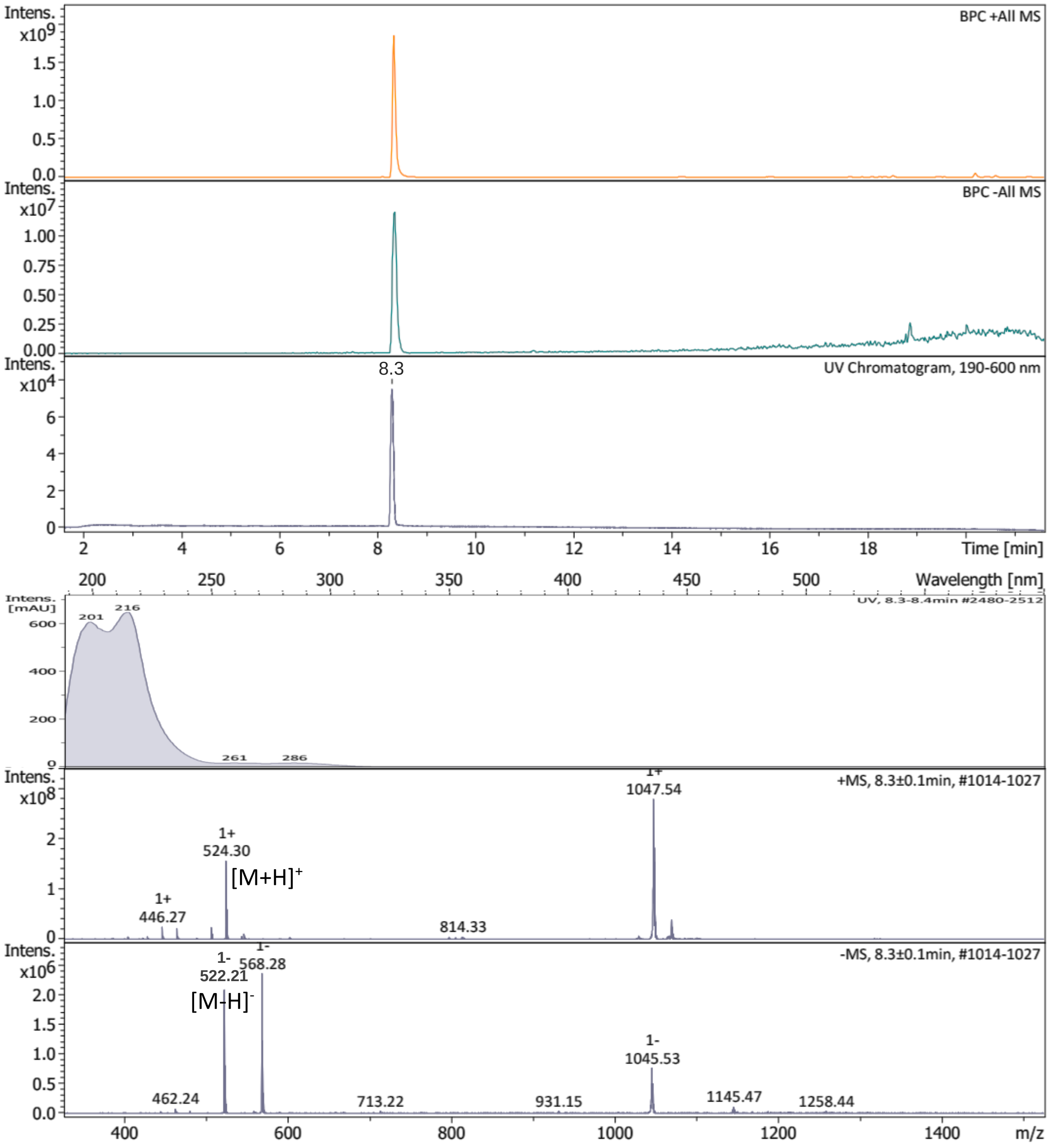

Bruker Compass DataAnalysis 6.1 printed: 20.11.2023 11:44:02 by: sel22 Page 1 of 1

Figure S54. LR-ESI-MS of **11**.

Display Report

Analysis Info Acquisition Date 28.09.2023 15:49:52

Analysis Name	S:\DATA\MaXis\dva23_Daniela Valencia Revelo\23_09_26\Gymnopus Slurry_R7_F6_73_01_13343.d		
Method	pos_säure_10000_screening_ms_100_2500_line.m	Operator	ate06
Sample Name	Gymnopus Slurry_R7_F6	Instrument	maXis
Comment	Screening01<br>Waters Acquity UPLC BEH C_18 1,7um 2.1x50mm		

Acquisition Parameter

Ion Polarity Positive

SPS Target Mass

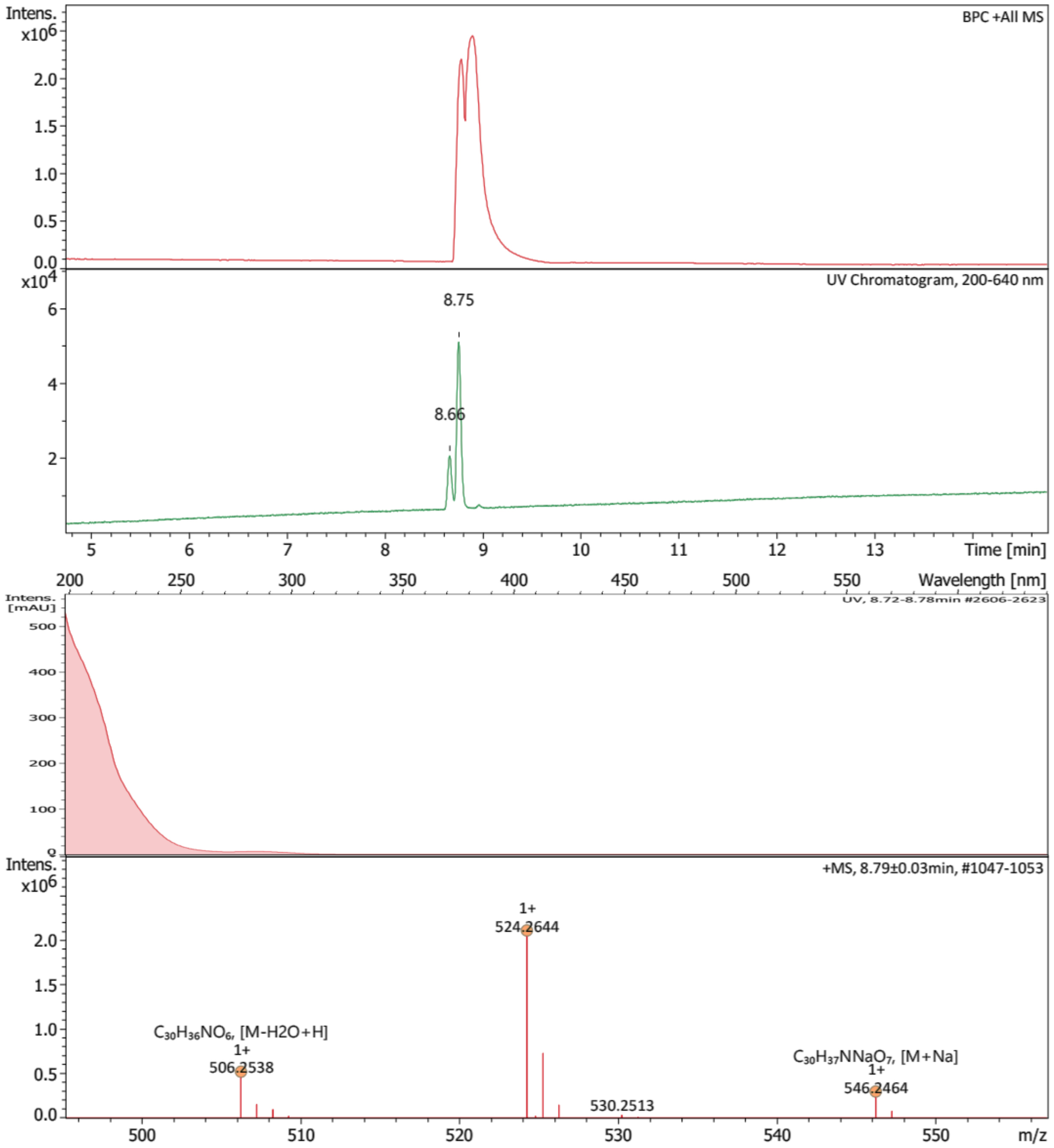

Bruker Compass DataAnalysis 6.1 printed: 23.11.2023 17:51:54 by: sel22 Page 1 of 1

Figure S55. HR-ESI-MS of **11**.

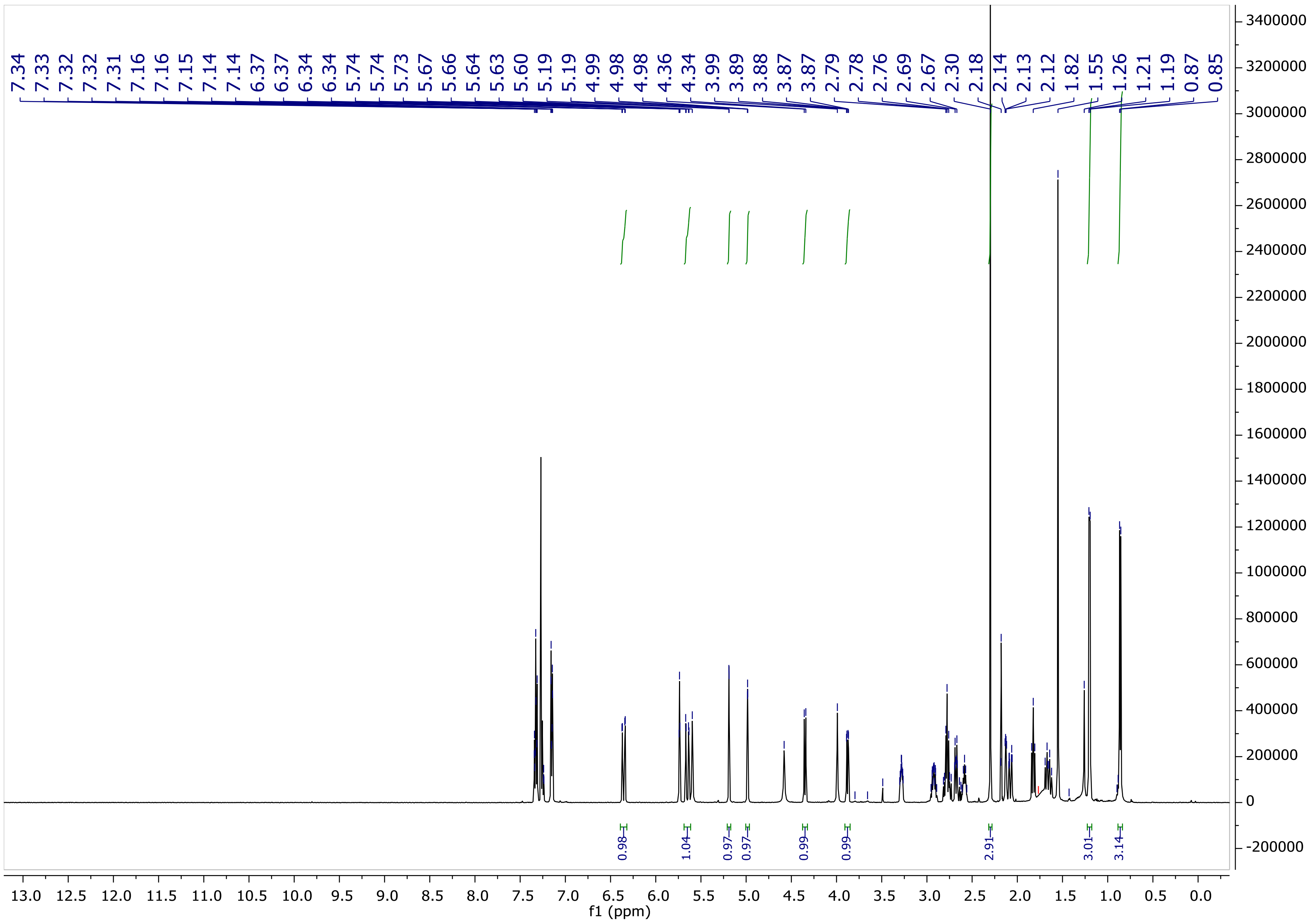

Figure S56. ^{1}H NMR spectrum of **11** in chloroform-*d* at 500 MHz.

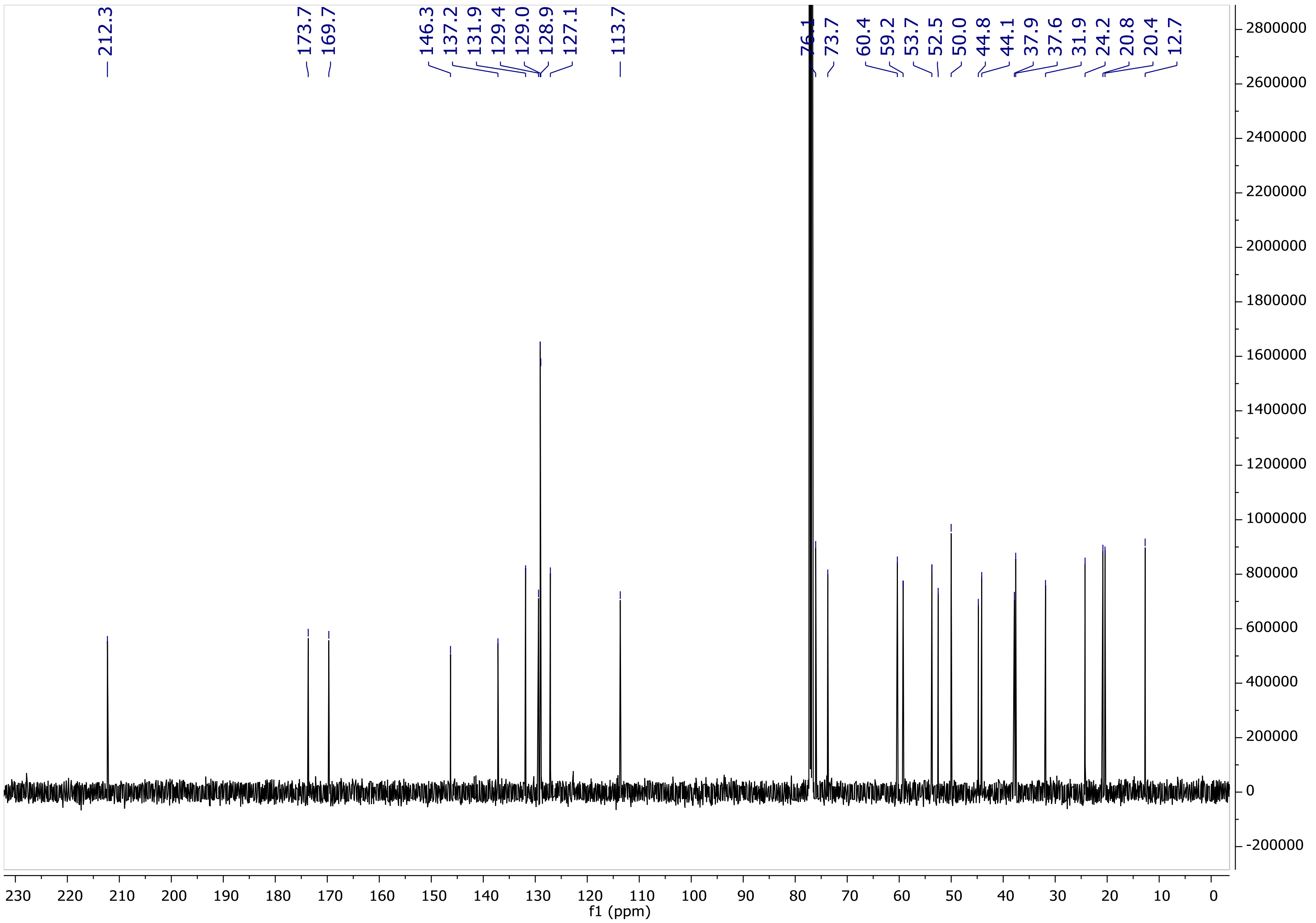

Figure S57. ^{13}C NMR spectrum of **11** in chloroform-*d* at 125 MHz.

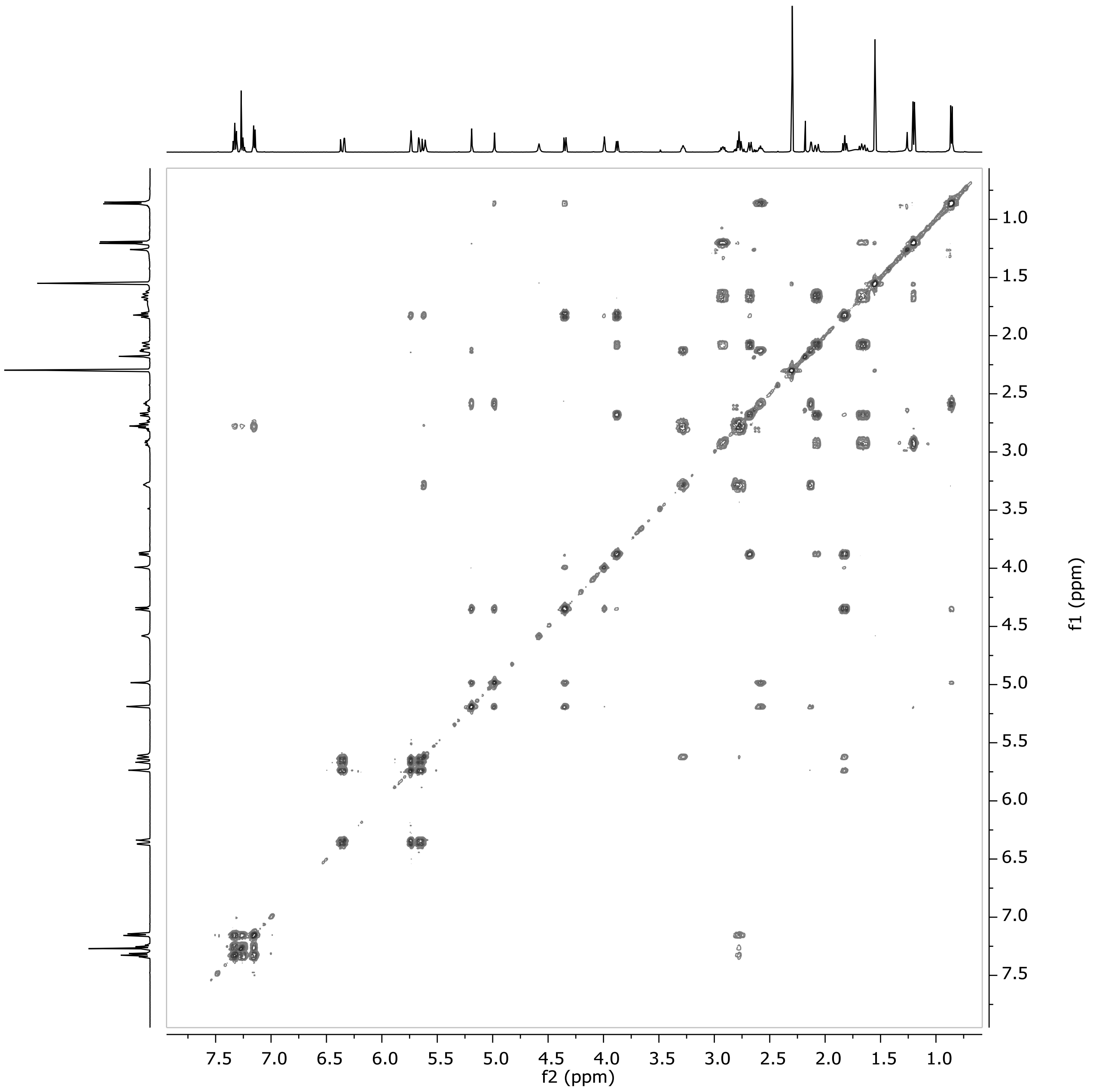

Figure S58. ^{1}H–^{1}H COSY spectrum of **11** in chloroform-*d* at 500 MHz.

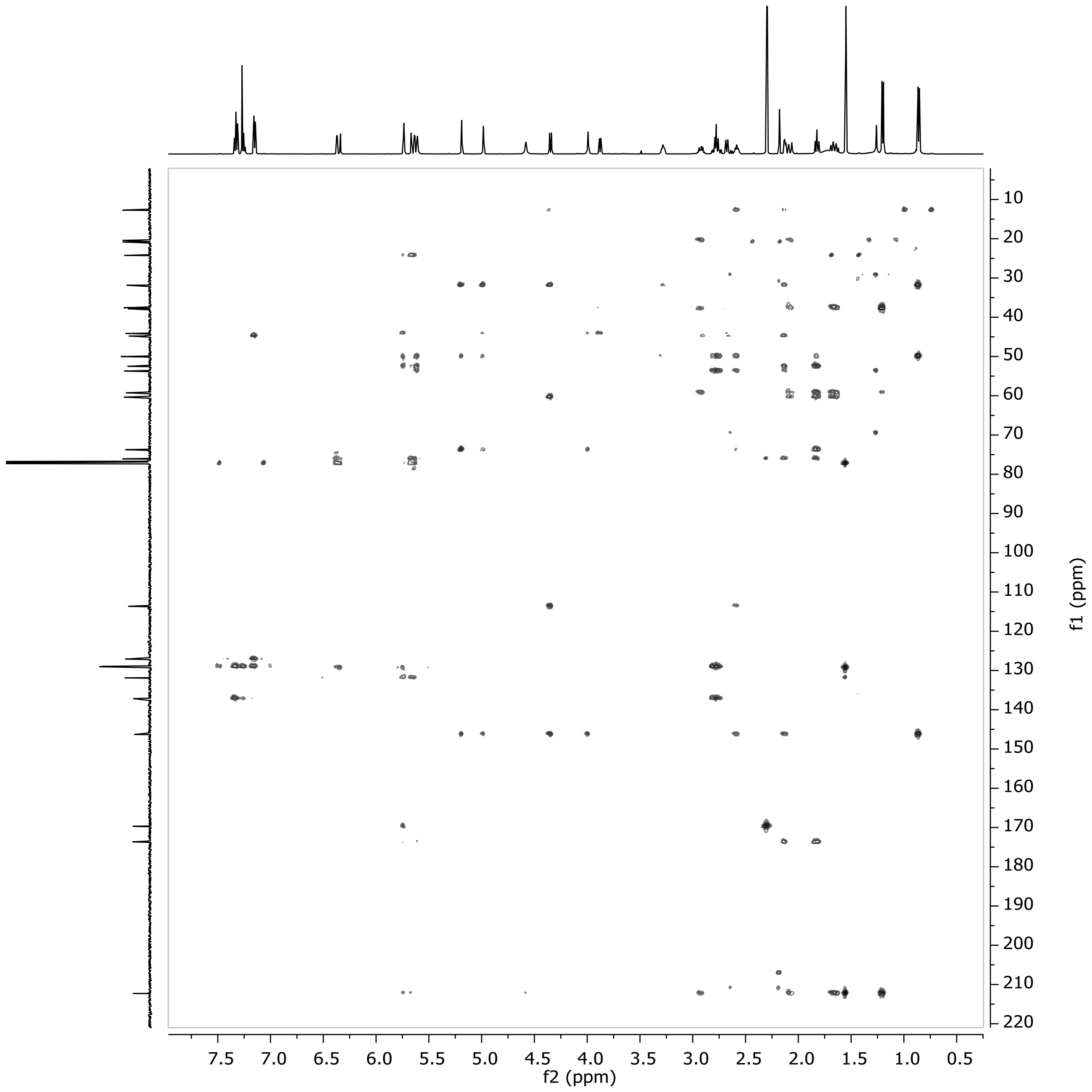

Figure S59. HMBC spectrum of **11** in chloroform-*d* at 500 MHz.

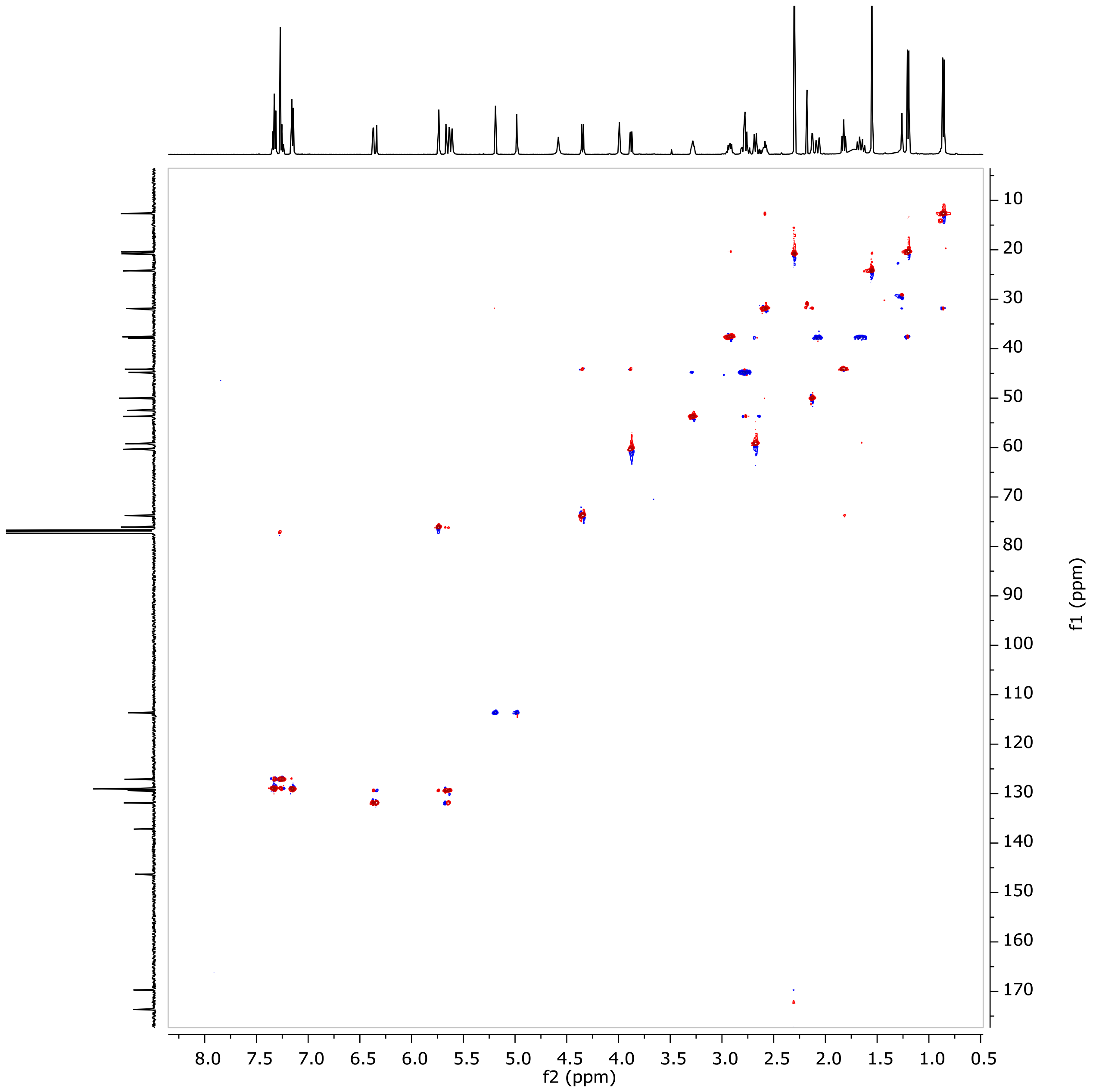

Figure S60. HSQC spectrum of **11** in chloroform-*d* at 500 MHz.

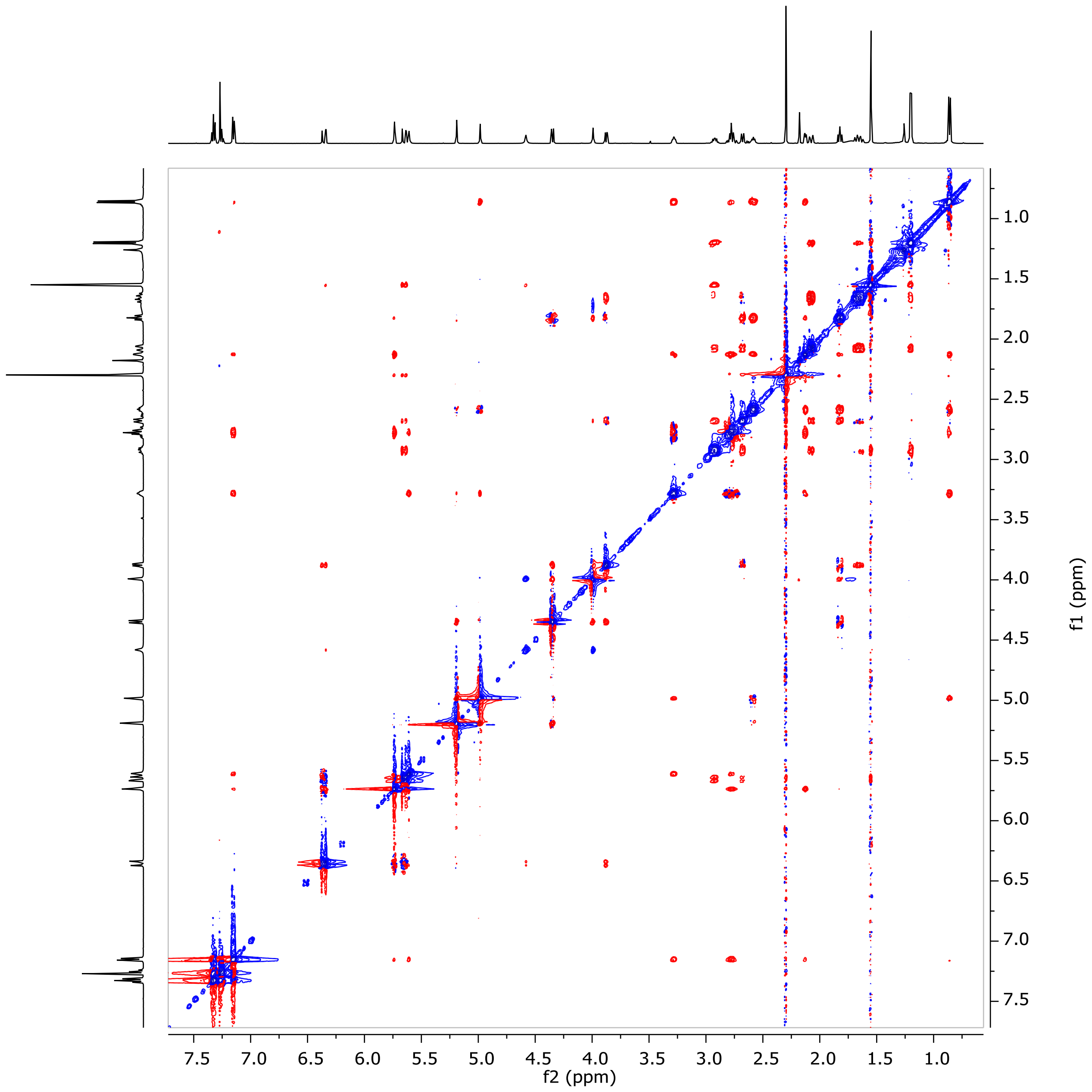

Figure S61. ROESY spectrum of **11** in chloroform-*d* at 500 MHz.

Table S16. ^{1}H and ^{13}C NMR data of compound **11** and 13,14-epoxycytochalasin D.

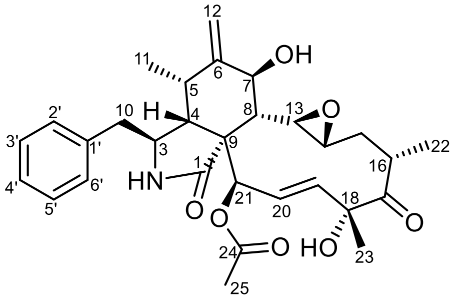

13,14-Epoxycytochalasin D

	Compound **11**		13,14-Epoxycytochalasin D	
pos.	δ_C,[a] type	δ_H[b] multi (*J* in Hz)	δ_C,[c] type	δ_H[d] multi (*J* in Hz)
1	173.8, CO	-	175.06, CO	-
2-N*H*	-	5.59 br s	-	9.35 br s
3	53.9, CH	3.27 m	54.38, CH	3.61 m
4	50.2, CH	2.12 dd (5.5, 2.7)	50.27, CH	2.46 dd (5.5, 2.7)
5	37.7, CH	2.91 dtd (10.6, 7.3, 6.1)	32.68, CH	2.84-2.91 m
6	146.5, C	-	149.31, C	-
7	73.9, CH	4.34 d (8.8)	74.38, CH	4.80 d (9.2)
7-O*H*	-	-	-	5.05 s
8	44.3, CH	1.81 dd (8.7, 8.7)	45.08, CH	2.31 dd (8.8, 8.8)
9	52.7, C	-	53.75, C	-
10	44.9, CH_2	α 2.74 dd (13.4, 9.0)<br>β 2.79 dd (13.4, 5.8)	45.30, CH_2	α 2.84-2.91 m<br>β 2.96-3.10 m
11	12.9, CH_3	0.85 d (6.8)	13.08, CH_3	0.74 d (6.8)
12	113.8, CH_2	α 4.97 d (1.2)<br>β 5.18 d (1.2)	112.23, CH_2	α 5.02 s<br>β 5.35 s
13	60.5, CH	3.87 dd (8.6, 2.3)	61.21, CH	4.44 dd (8.4, 2.2)
14	59.4, CH	2.67 dt (10.1, 2.4)	59.41, CH	2.96-3.10 m
15	38.0, CH_2	α 2.07 d (14.6)<br>β 1.65 dt (14.6, 10.5)	38.52, CH_2	α 1.96-2.15 m<br>β 1.96-2.15 m
16	37.7, CH	2.91 dtd (10.6, 7.3, 6.1)	38.98, CH	2.96-3.10 m
17	212.4, CO	-	213.18, CO	-
18	76.2, C	-	78.68, C	-
18-O*H*	-	-	-	6.62 br s
19	129.5, CH	5.64 dd (15.7, 2.6)	131.00, CH	6.20 dd (15.8, 2.6)
20	132.0, CH	6.35 dd (15.7, 2.4)	132.41, CH	7.18 dd (15.8, 2.4)
21	76.2, CH	5.73 dd (2.5, 2.5)	77.07, CH	6.14 dd (2.6, 2.4)
22	20.6, CH_3	1.19 d (6.9)	20.42, CH_3	1.09 d (6.8)
23	24.4, CH_3	1.54 s	20.56, CH_3	1.63 s
24	169.9, CO	-	170.74, CO	-
25	21.0, CH_3	2.29 s	24.89, CH_3	2.39 s
1'	137.3, C	-	138.44, C	-
2'	129.2, CH	7.14 d (7.0)	130.02, CH	7.22-7.35 m
3'	129.1, CH	7.32 t (7.4)	128.96, CH	7.22-7.35 m
4'	127.2, CH	7.24 t (7.4)	127.00, CH	7.22-7.35 m
5'	129.1, CH	7.32 t (7.4)	128.96, CH	7.22-7.35 m
6'	129.2, CH	7.14 d (7.0)	130.02, CH	7.22-7.35 m

Measured in chloroform-*d* at [a] 150 and [b] 600 MHz. Measured in pyridine-d_5 at [c] 67.8 and [d] 270 MHz.

Generic Display Report

Analysis Info

Analysis Name	S:\DATA\AmaZon\dva23_Daniela Valencia Revelo\Gymnopus montagnei\4. Gymnopus Slurry\4. Semiprep\Semipreps\Semiprep 1\GymSlurry SemiprepF1 R1F8_BB8_01_51357.d	Acquisition Date	03.10.2023 09:52:23
Method	51357.m	Operator	td
Sample Name	GymSlurry SemiprepF1 R1F8	Instrument	amaZon speed
Comment			

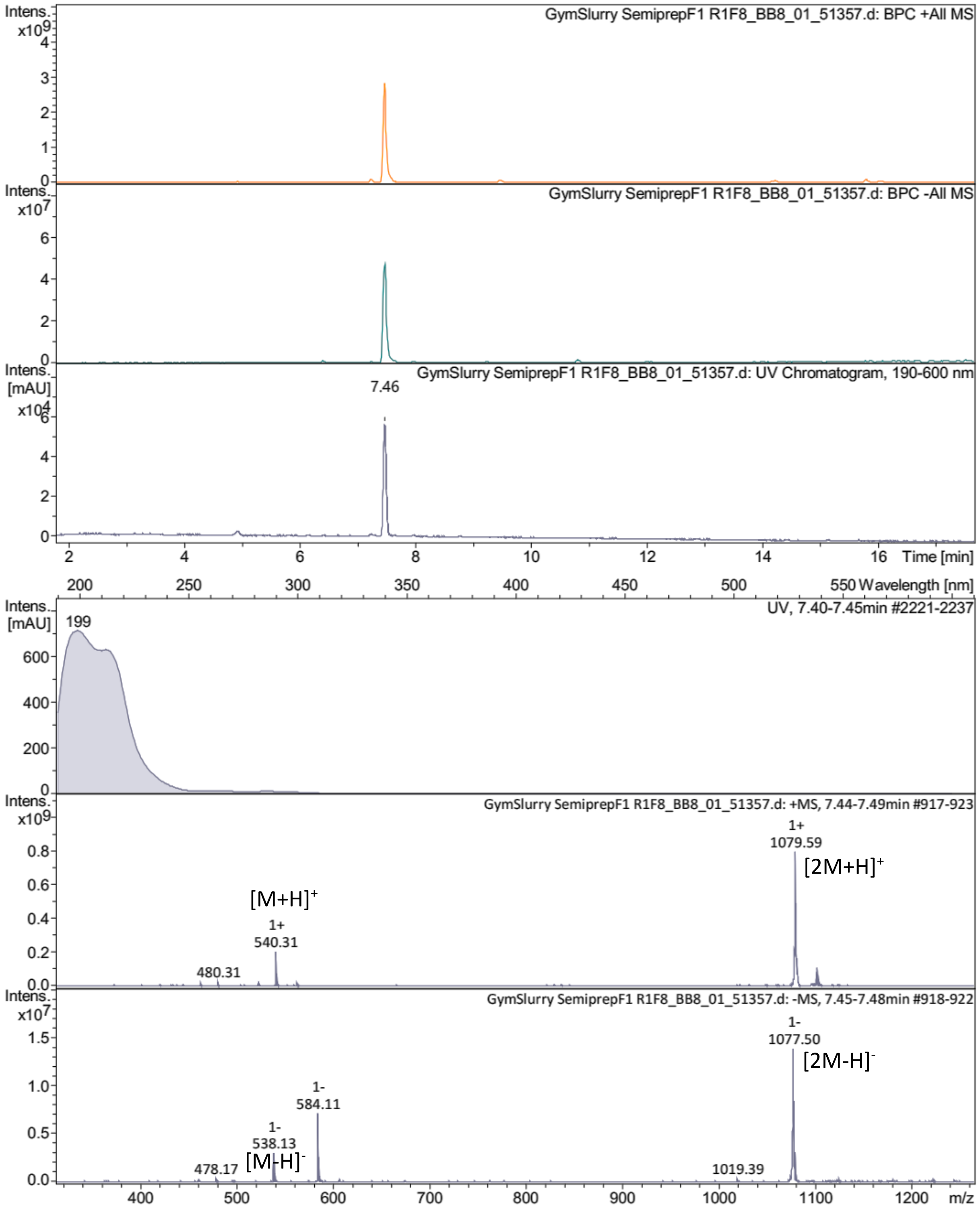

Bruker Compass DataAnalysis 4.4 printed: 10.10.2023 17:31:55 by: sel22 Page 1 of 1

Figure S62. LR-ESI-MS of **12**.

Generic Display Report

**Analysis Info**		Acquisition Date	27.09.2023 17:51:55
Analysis Name	S:\DATA\MaXis\dva23_Daniela Valencia Revelo\23_09_26\Gymnopus Slurry_R6_F1_56_01_13324.d		
Method	pos_säure_10000_screening_ms_100_2500_line.m	Operator	ate06
Sample Name	Gymnopus Slurry_R6_F1	Instrument	maXis
Comment	Screening01<br>Waters Acquity UPLC BEH C_18 1,7um 2.1x50mm		

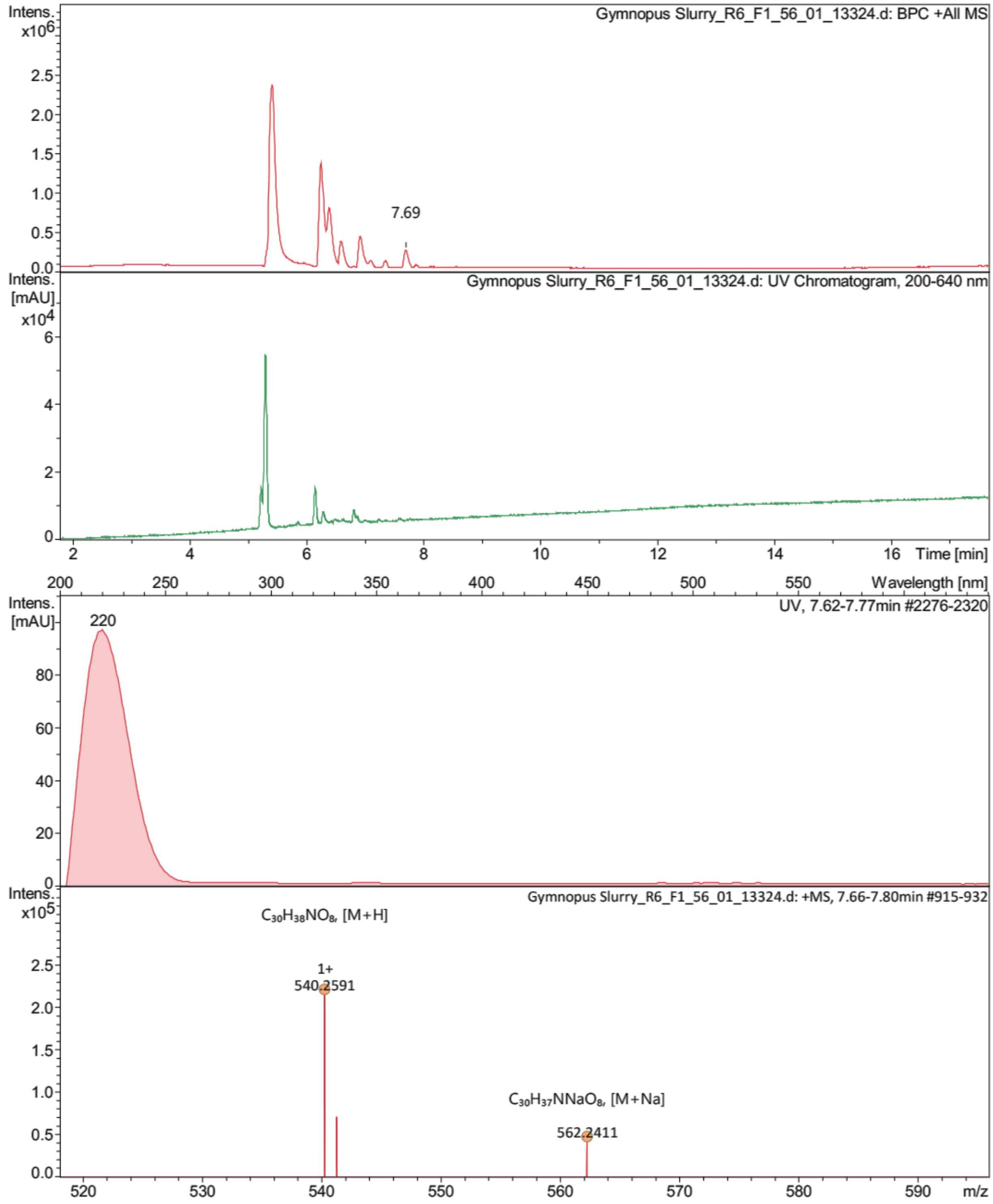

Bruker Compass DataAnalysis 4.4 printed: 10.10.2023 17:35:12 by: sel22 Page 1 of 1

Figure S63. HR-ESI-MS of **12**.

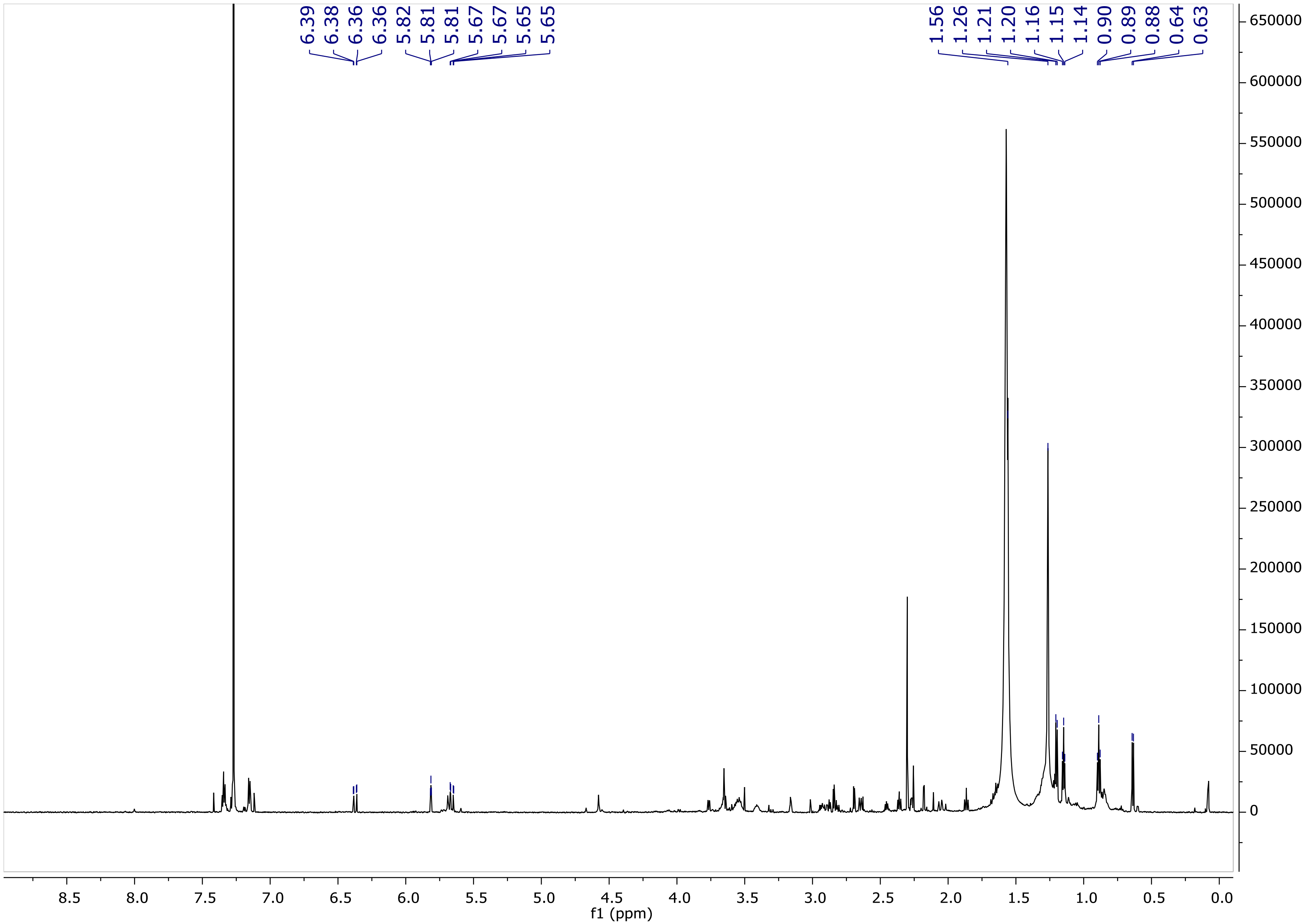

Figure S64. ^{1}H NMR spectrum of **12** in chloroform-*d* at 700 MHz.

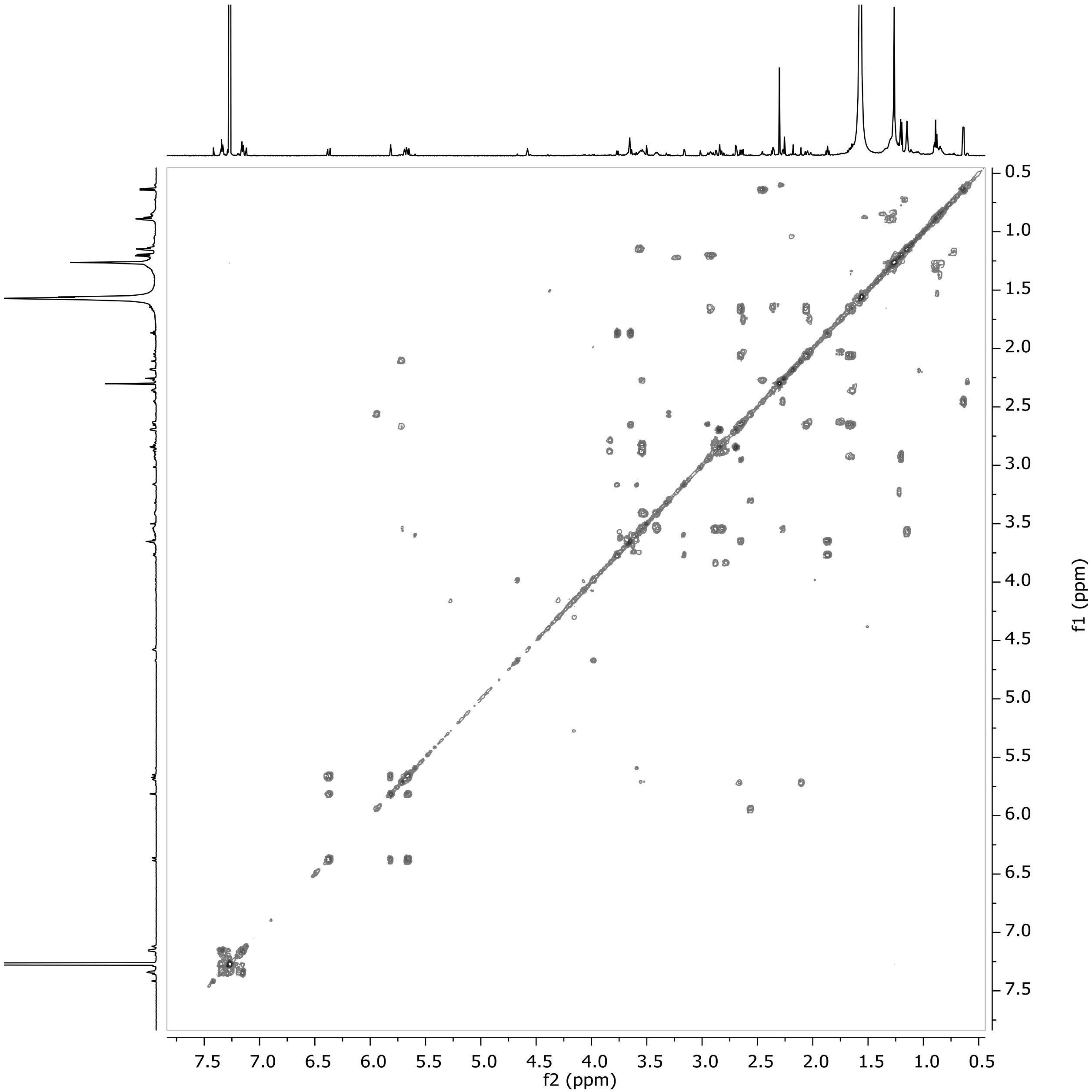

Figure S65. ^{1}H-^{1}H COSY spectrum of **12** in chloroform-*d* at 700 MHz.

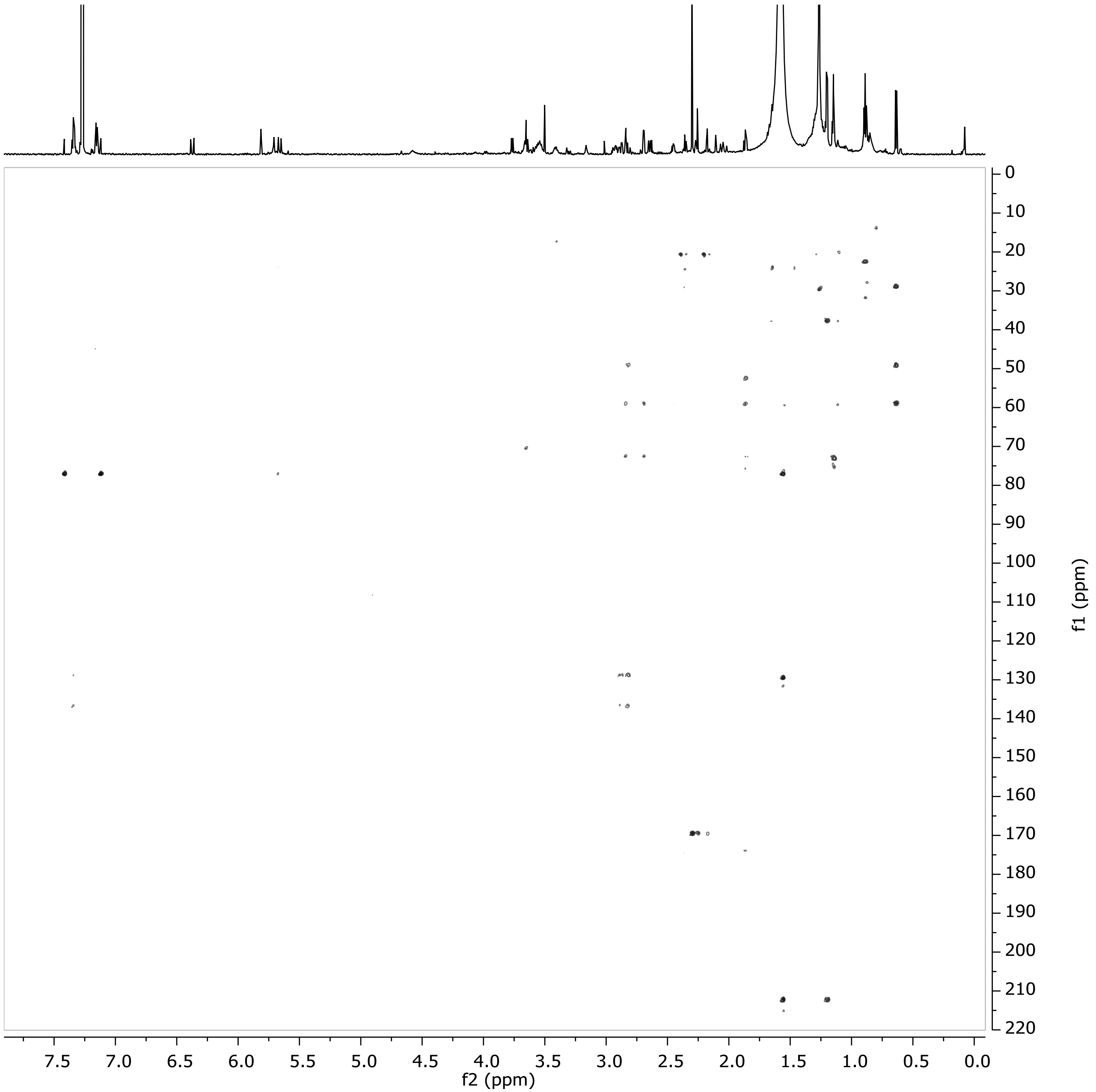

Figure S66. HMBC spectrum of **12** in chloroform-*d* at 700 MHz.

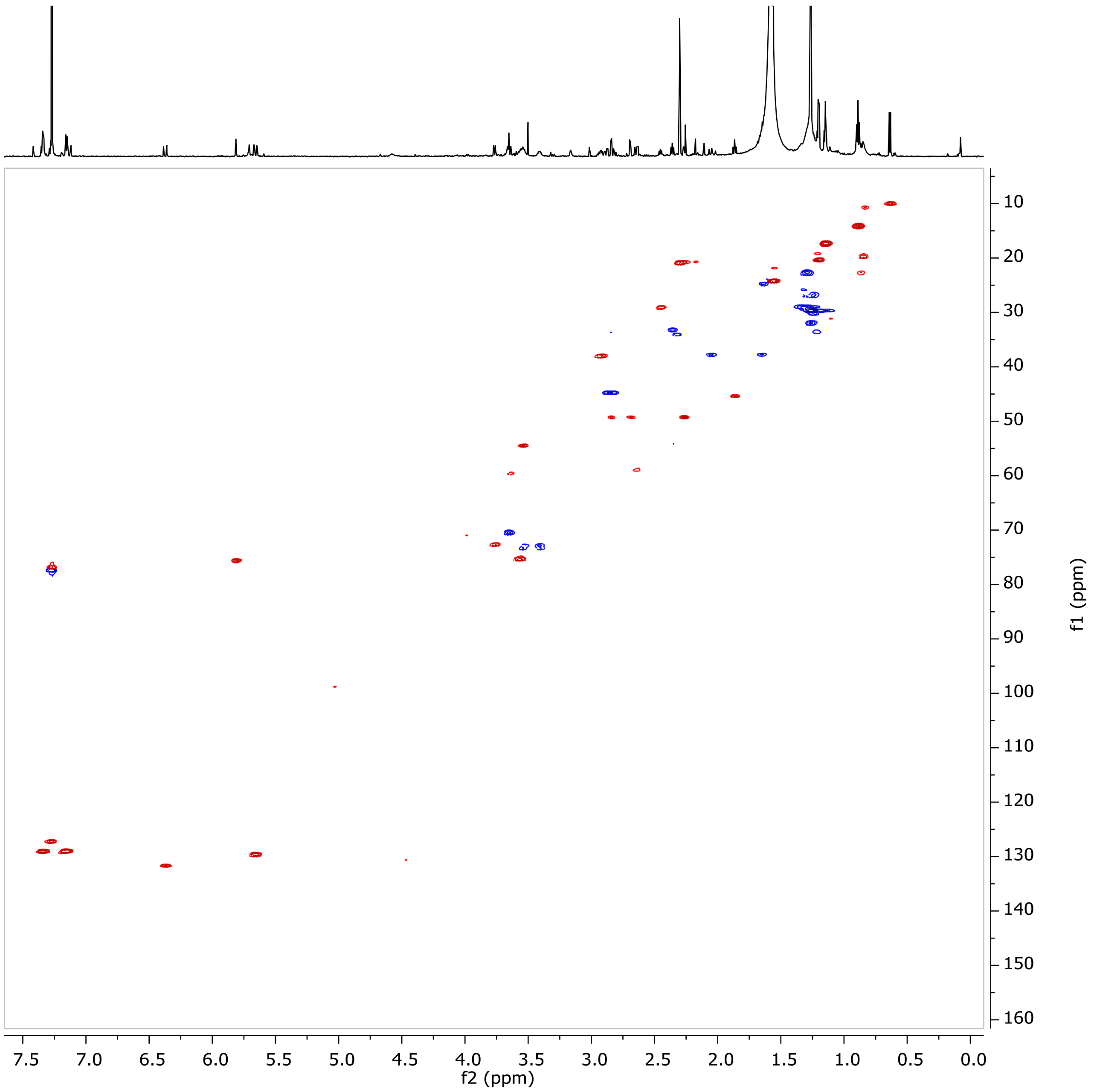

Figure S67. HSQC spectrum of **12** in chloroform-*d* at 700 MHz.

Table S17. ^{1}H and ^{13}C NMR data of compound **12**, 6,12:13,14-diepoxy- and 6,12-epoxycytochalasins D.

	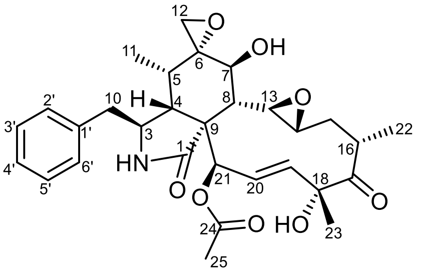 6,12:13,14-Diepoxycytochalasin D			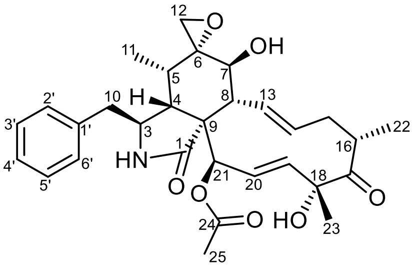 6,12-Epoxycytochalasin D	
	Compound **12**		6,12:13,14-Diepoxycyto D	6,12-Epoxycytochalasin D	
pos.	δ_C,[a] type	δ_H[b] multi (*J* in Hz)	δ_H[b] multi (*J* in Hz)	δ_C,[a] type	δ_H[b] multi (*J* in Hz)
1	174.0, CO	-	-	173.6, CO	-
2-N*H*	-	5.68 br s	-	-	-
3	54.5, CH	3.53 m (overlapped)	n.r.	53.9, CH	3.78 m
4	49.3, CH	2.26 dd (5.7, 2.8)	n.r.	49.7, CH	2.17 dd (4.6, 2.9)
5	29.1, CH	2.45 q (6.6)	n.r.	29.3, CH	2.40 m
6	75.7, C	-	-	61.2, C	-
6-O*H*	-	-	-	-	-
7	59.6, CH	3.63 dd (8.8, 2.0)	4.56 s	59.6, CH	3.27 d (10.7)
7-O*H*	-	-	-	-	-
8	45.4, CH	1.86 t (8.7)	n.r.	47.3, CH	2.75 dd (10.7, 10.7)
9	52.5, C	-	-	53.1, C	-
10	44.8, CH$_2$	α 2.82 dd (13.5, 4.4)<br>β 2.87 dd (13.5, 5.5)	n.r.	45.6, CH$_2$	α 2.69 dd (13.3, 8.9)<br>β 2.82 dd (13.3, 5.4)
11	10.0, CH$_3$	0.63 d (7.0)	0.61 d (7.3)	10.3, CH$_3$	0.64 d (6.9)
12	49.3, CH$_2$	α 2.68 d (4.7)<br>β 2.83 d (4.7)	3.56-3.82 d	45.4, CH$_2$	α 2.64 d (4.7)<br>β 2.97 d (4.7)
13	72.7, CH	3.76 dd (8.9, 2.0)	3.56-3.82 dd	130.5, CH	5.69 overlapped
14	59.0, CH	2.64 dt (9.8, 2.3)	n.r.	134.4, CH	5.32 ddd (15.6, 10.7, 5.0)
15	37.8, CH$_2$	α 1.65 dt (14.7, 10.4)<br>β 2.05 d (14.3)	n.r.	37.6, CH$_2$	α 2.00 br dd (12.6, 5.0)<br>β 2.51 ddd (12.6, 10.9, 10.9)
16	38.0, CH	2.92 m	n.r.	42.3, CH	2.74 m
17	212.3, CO	-	-	210.4, CO	-
18	77.2, C	-	-	77.6, C	-
18-O*H*	-	-	-	-	-
19	129.6, CH	5.65 dd (15.7, 2.6)	5.59 dd (16.0, 2.0)	127.7, CH	5.14 dd (15.5, 2.1)
20	131.7, CH	6.36 dd (15.7, 2.4)	6.37 dd (16.0, 2.0)	132.4, CH	6.14 dd (15.5, 2.5)
21	75.6, CH	5.79 t (2.5)	5.79 m	77.0, CH	5.68 overlapped
22	20.4, CH$_3$	1.19 d (6.8)	1.18 d (6.0)	19.4, CH$_3$	1.20 d (6.9)
23	24.2, CH$_3$	1.55 s	1.54 s	24.2, CH$_3$	1.51 s
24	169.7, CO	-	-	169.6, CO	-
25	20.8, CH$_3$	2.29 s	2.28 s	20.9, CH$_3$	2.27 s
1'	136.8, C	-	-	137.2, C	-
2'	129.0, CH	7.14 d (7.0)	7.00-7.35 m	129.2, CH	7.15 d (7.3)
3'	129.1, CH	7.33 t (7.5)	7.00-7.35 m	128.9, CH	7.31 t (7.3)
4'	127.3, CH	7.27 t (7.5)	7.00-7.35 m	127.0, CH	7.24 t (7.3)
5'	129.1, CH	7.33 t (7.5)	7.00-7.35 m	128.9, CH	7.31 t (7.3)
6'	129.0, CH	7.14 d (7.0)	7.00-7.35 m	129.2, CH	7.15 d (7.3)

Measured in chloroform-*d* at [a] 150 and [b] 600 MHz.

Display Report

Analysis Info

Analysis Name	S:\DATA\AmaZon\dva23_Daniela Valencia Revelo\Gymnopus montagnei\4. Gymnopus Slurry\4. Semiprep\GymSlurry_SP4_R1F3(PreMeasured_RD3_01_52025.d	Acquisition Date	03.11.2023 22:11:05
Method	52025.m	Operator	tti
Sample Name	GymSlurry_SP4_R1F3(PreMeasured	Instrument	amaZon speed
Comment			

Acquisition Parameter

Ion Source Type	ESI	Ion Polarity	Positive	Alternating Ion Polarity	on
Mass Range Mode	UltraScan	Scan Begin	100 m/z	Scan End	2000 m/z
Accumulation Time	4000 µs	RF Level	100 %	Trap Drive	68.6
SPS Target Mass	1000 m/z	Averages	6 Spectra		

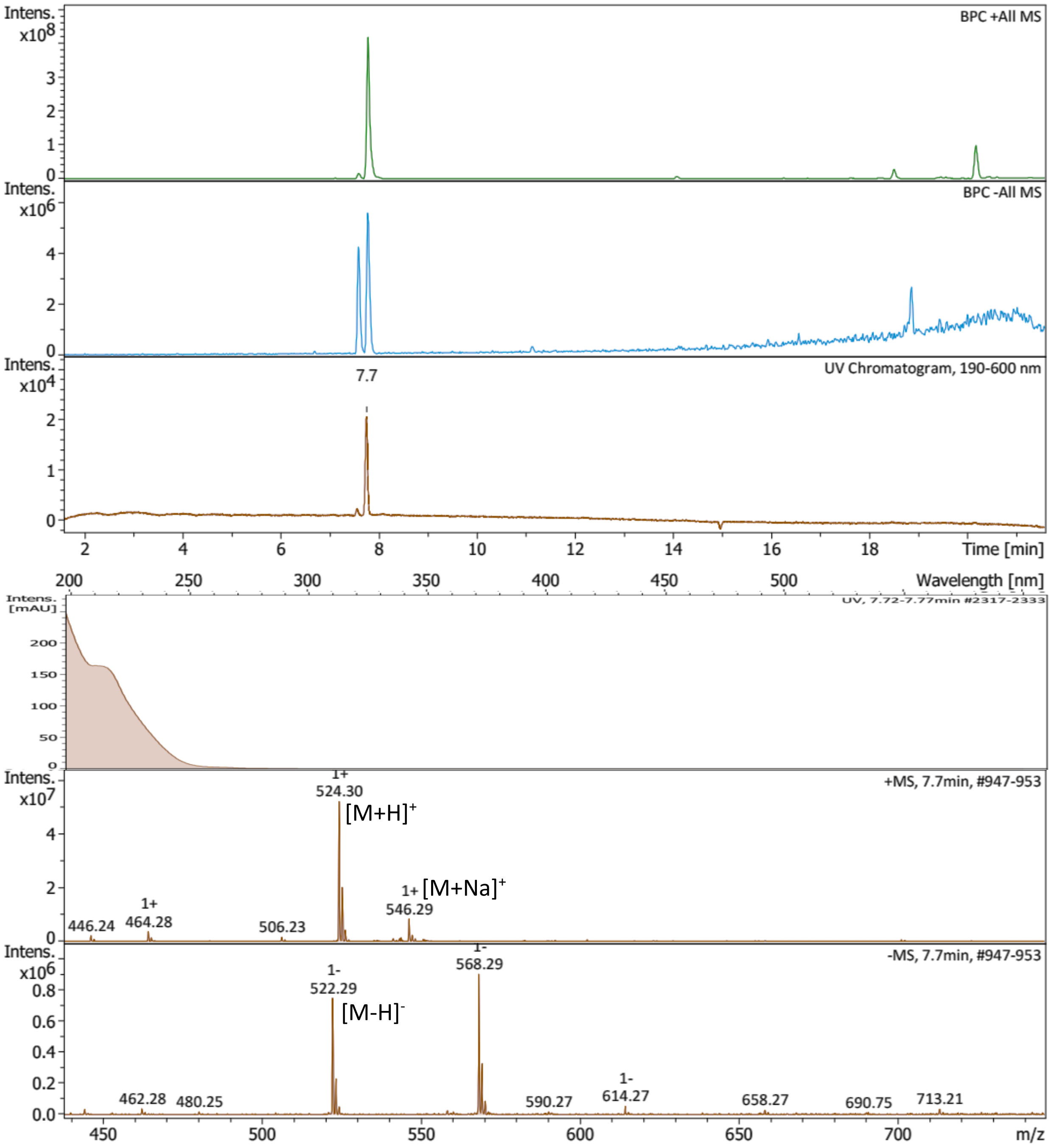

Bruker Compass DataAnalysis 6.1 printed: 09.11.2023 12:08:46 by: sel22 Page 1 of 1

Figure S68. LR-ESI-MS of **13**.

Display Report

Analysis Info

Analysis Name	S:\DATA\MaXis\dva23_Daniela Valencia Revelo\23_09_26\Gymnopus Slurry_R4_F4_14_01_13261.d	Acquisition Date	26.09.2023 09:36:25
Method	pos_säure_10000_screening_ms_100_2500_line.m	Operator	ate06
Sample Name	Gymnopus Slurry_R4_F4	Instrument	maXis
Comment	Screening01<br>Waters Acquity UPLC BEH C_18 1,7um 2.1x50mm		

Acquisition Parameter

Ion Polarity Positive

SPS Target Mass

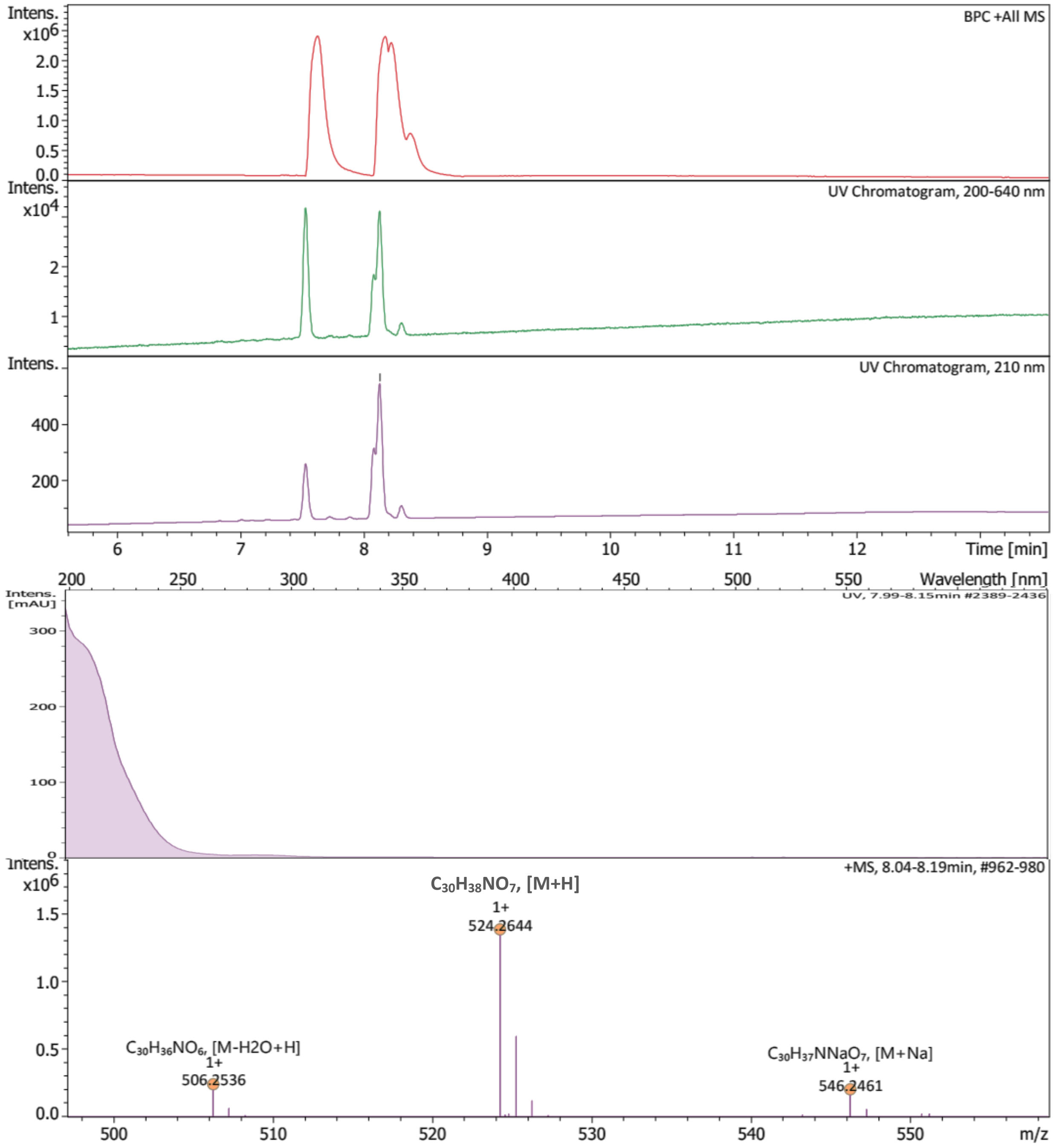

Bruker Compass DataAnalysis 6.1 printed: 09.11.2023 12:16:17 by: sel22 Page 1 of 1

Figure S69. HR-ESI-MS of **13**.

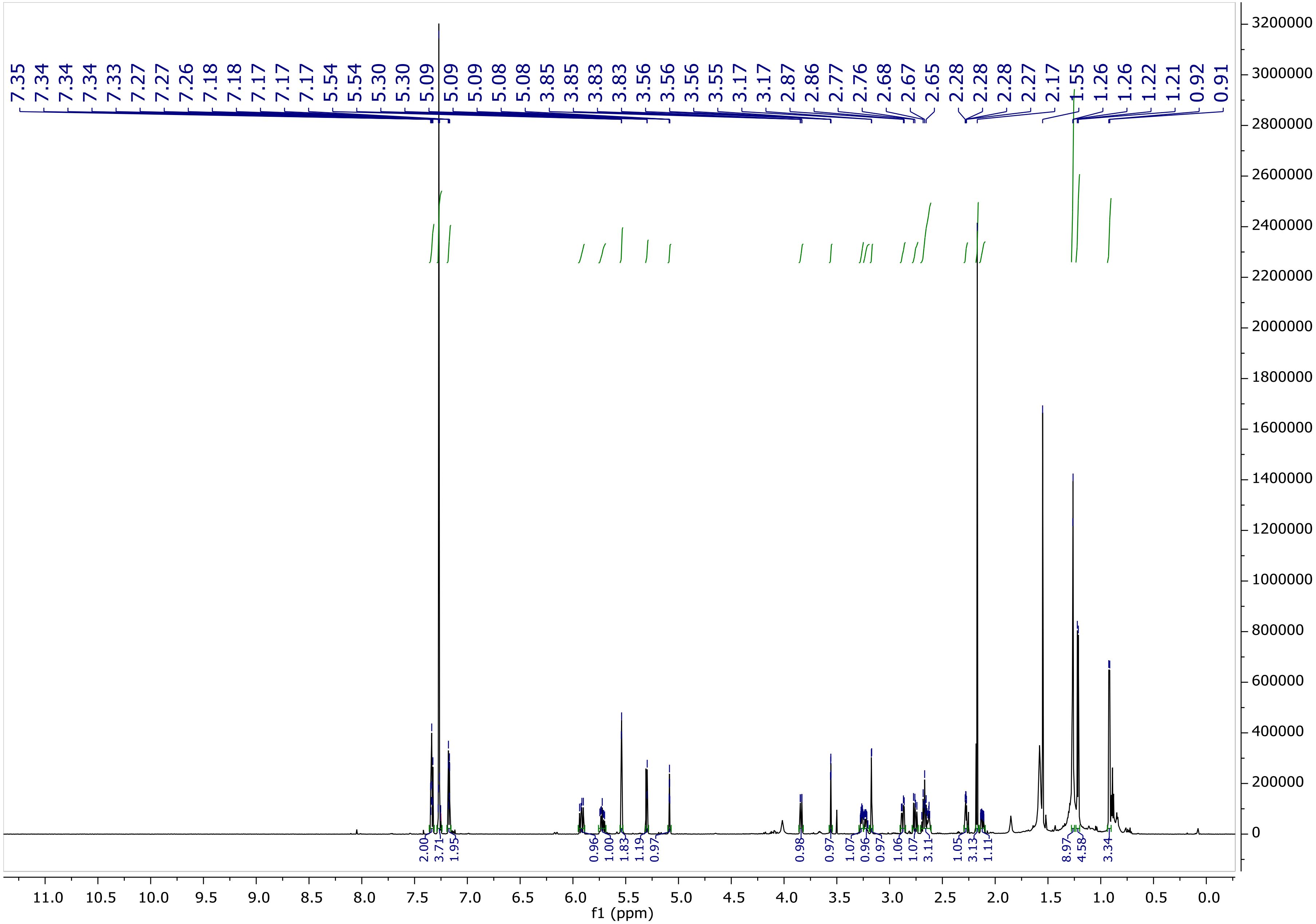

Figure S70. ^{1}H NMR spectrum of **13** in chloroform-*d* at 700 MHz.

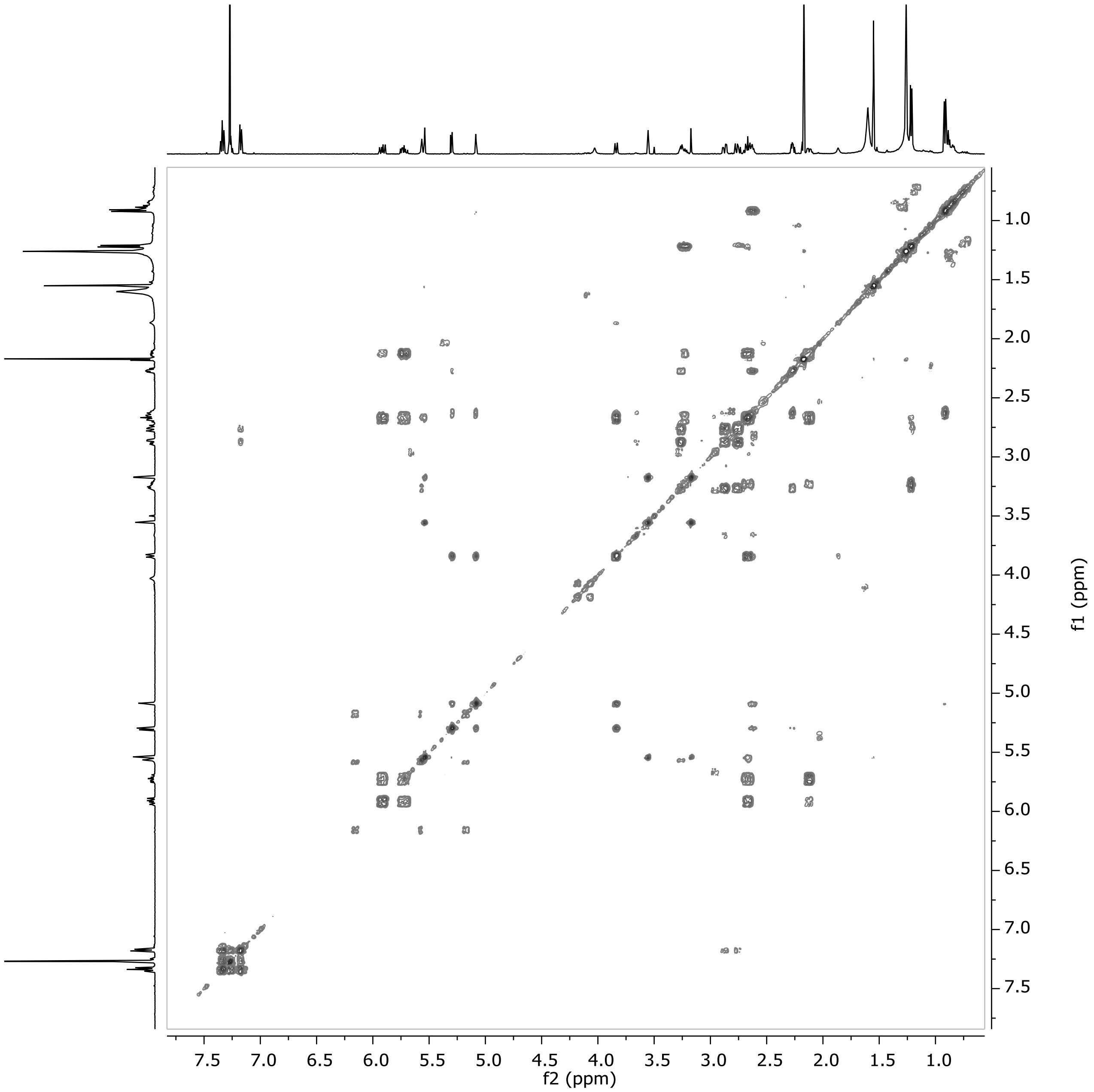

Figure S71. ^{1}H-^{1}H COSY spectrum of **13** in chloroform-*d* at 700 MHz.

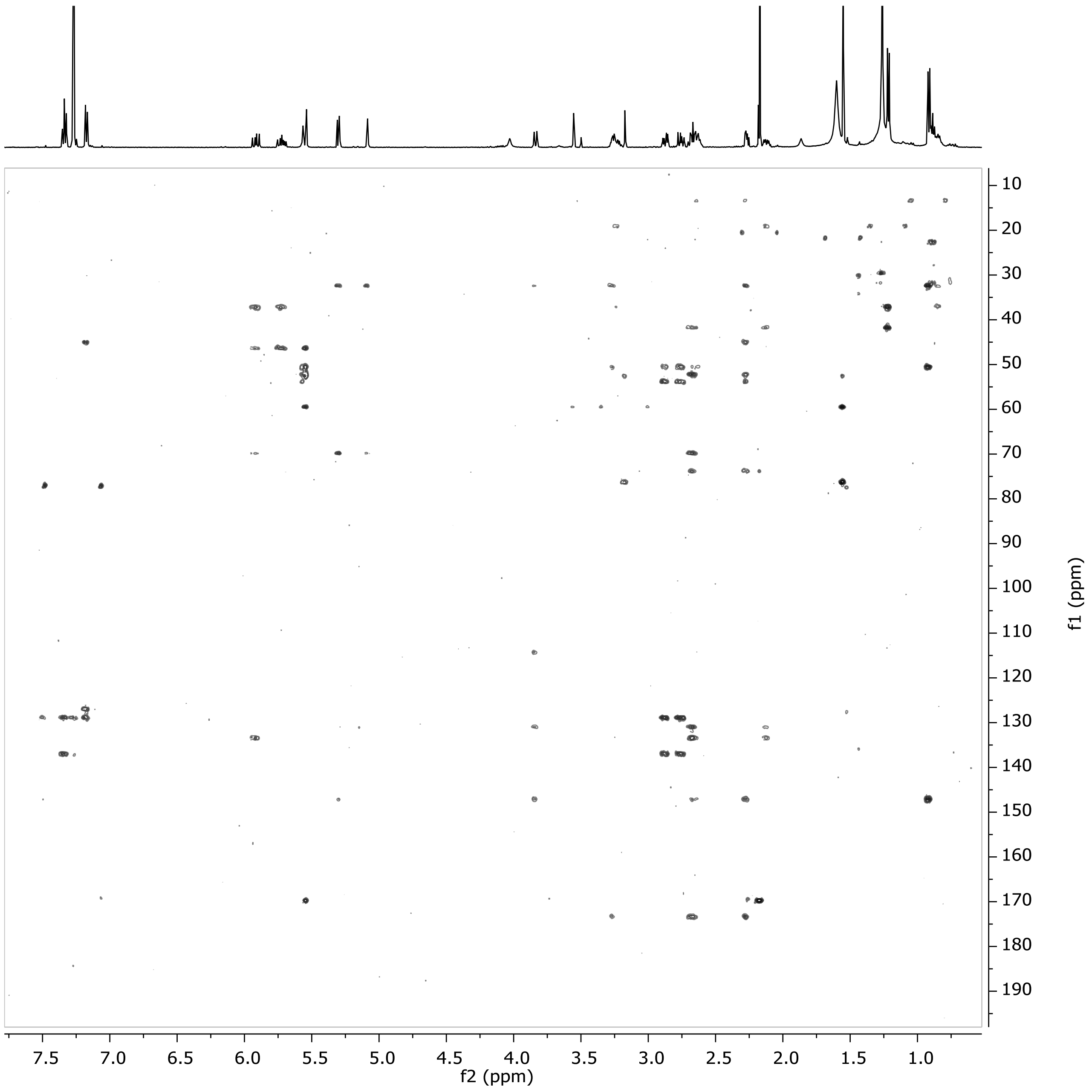

Figure S72. HMBC spectrum of **13** in chloroform-*d* at 700 MHz.

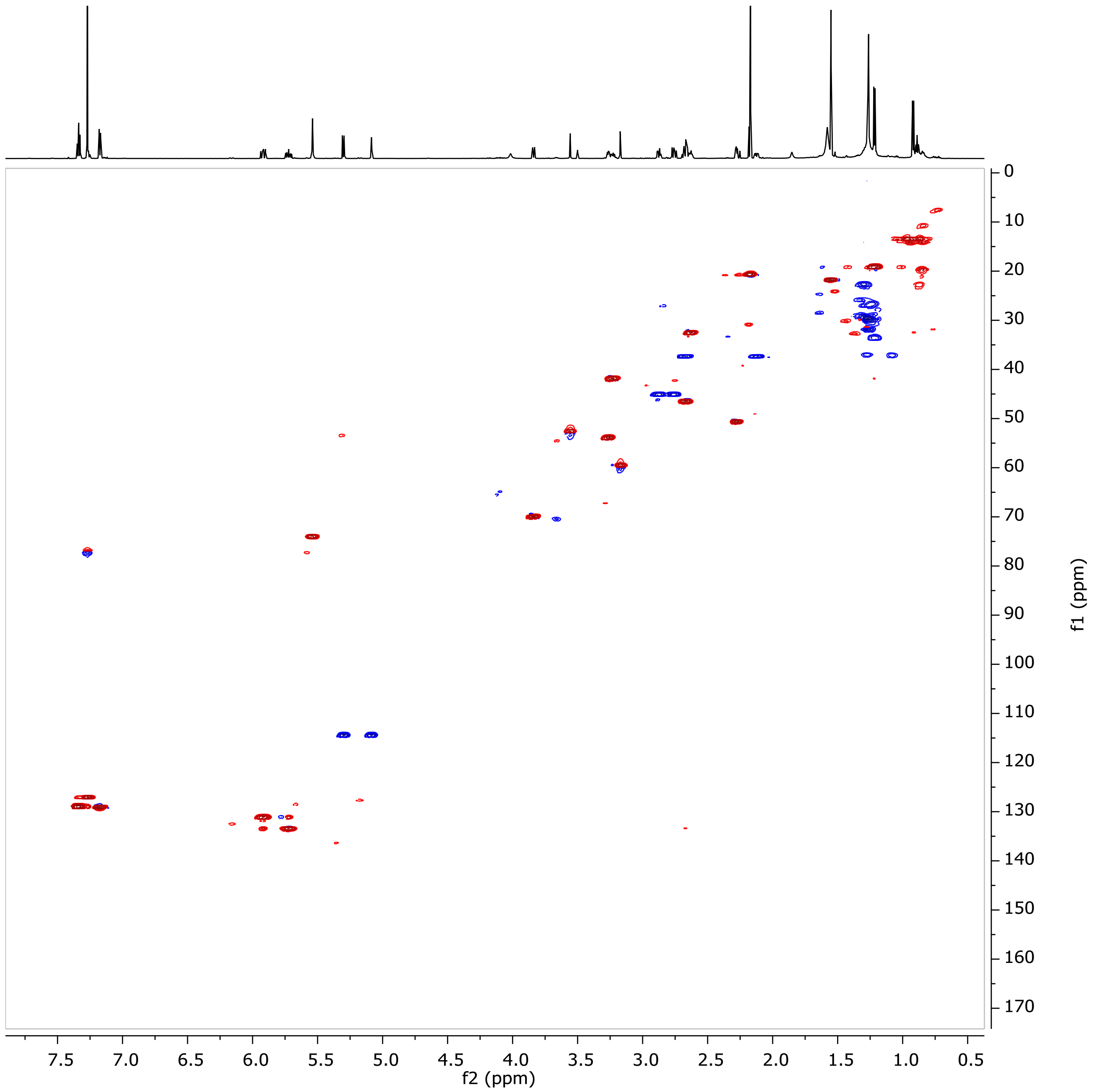

Figure S73. HSQC spectrum of **13** in chloroform-*d* at 700 MHz.

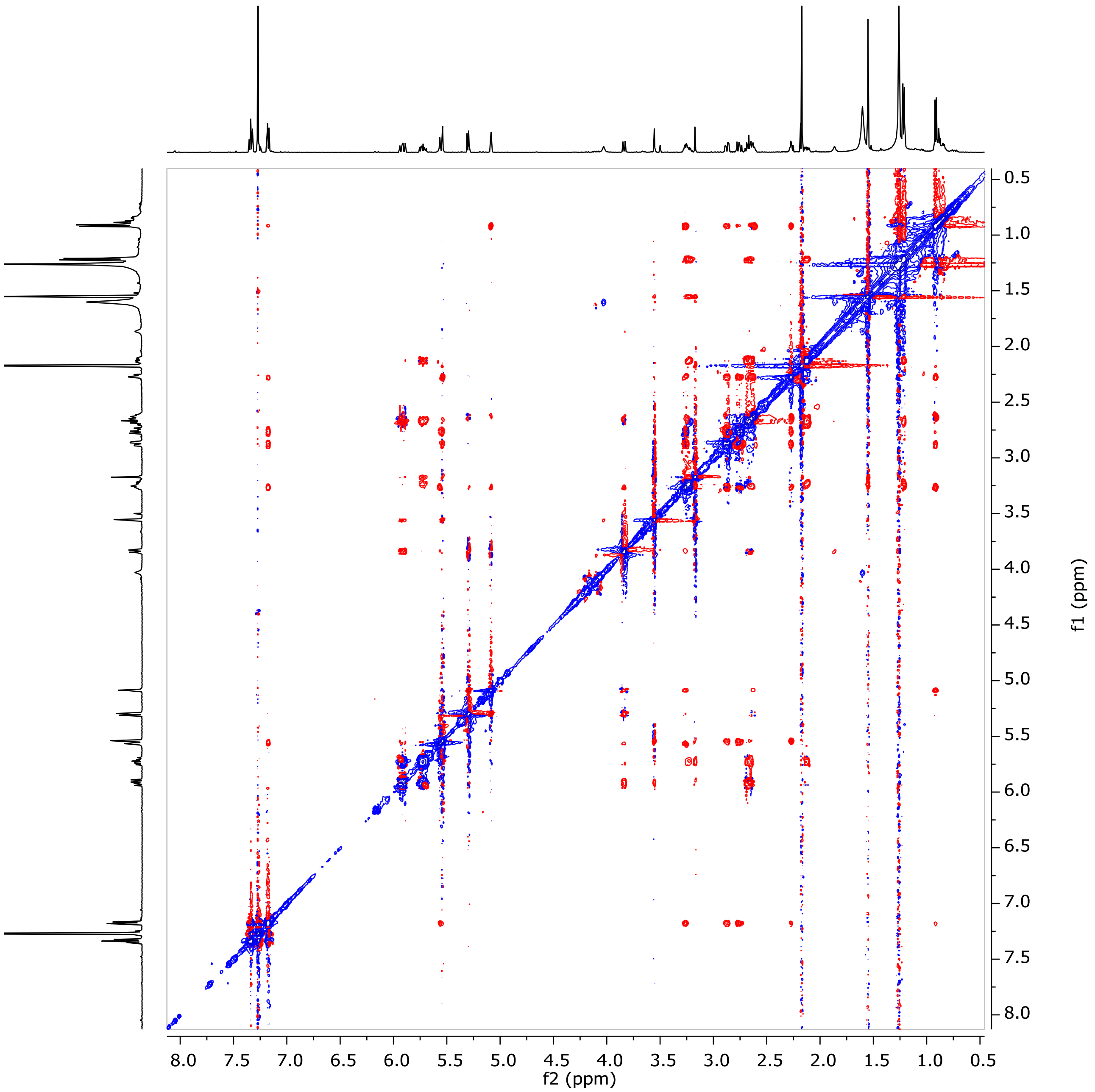

Figure S74. ROESY spectrum of **13** in chloroform-*d* at 700 MHz.

Table S18. ^{1}H and ^{13}C NMR data of compound **13** and 19,20-epoxycytochalasin D.

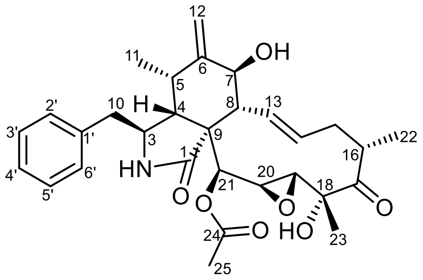

19,20-Epoxycytochalasin D

	Compound **13**		19,20-Epoxycytochalasin D	
pos.	δ_C,[a] type	δ_H[b] multi (*J* in Hz)	δ_C,[c] type	δ_H[d] multi (*J* in Hz)
1	173.3, CO	-	173.43, CO	-
2-N*H*	-	5.54 br s	-	5.46 br s
3	53.8, CH	3.25 m	53.90, CH	3.24 m
4	50.6, CH	2.28 dd (5.3, 3.3)	50.71, CH	2.25 dd (5.2, 3.3)
5	32.6, CH	2.62 m	32.56, CH	2.62 m
6	147.1, C	-	147.35, C	-
6-O*H*				
7	69.9, CH	3.83 dd (10.4, 1.2)	69.98 CH	3.81 br d (10.1)
7-O*H*	-	-	-	-
8	46.4, CH	2.62 m (overlapped)	46.54, CH	2.62 m
9	52.3, C	-	52.45, C	-
10	45.1, CH_2	α 2.87 dd (13.5, 5.1)<br>β 2.75 dd (13.5, 9.2)	45.18, CH_2	α 2.85 dd (13.4, 5.0)<br>β 2.73 dd (13.4, 9.1)
11	13.4, CH_3	0.91 d (6.7)	13.49, CH_3	0.89 d (6.9)
12	114.4, CH_2	α 5.29 d (1.9)<br>β 5.08 p (1.1)	114.44, CH_2	α 5.26 br s<br>β 5.06 br s
13	131.1, CH	5.91 dd (15.5, 9.8)	131.17, CH	5.89 dd (15.5, 9.8)
14	133.4, CH	5.71 ddd (15.8, 10.0, 6.0)	133.47, CH	5.69 ddd (15.5, 9.9, 5.8)
15	37.4, CH_2	α 2.66 m (overlapped)<br>β 2.11 dddd (12.9, 5.9, 2.2, 1.2)	37.40, CH_2	α 2.62 m<br>β 2.09 m
16	41.8, CH	3.22 ddd (11.9, 6.8, 2.2)	41.89, CH	3.22 m
17	215.2, CO	-	215.28, CO	-
18	76.2, C	-	76.32, C	-
18-O*H*	-	-	-	6.54 br s
19	59.5, CH	3.16 d (2.1)	59.63, CH	3.14 d (1.9)
20	52.6, CH	3.55 dd (2.1, 1.4)	52.72, CH	3.53 dd (1.9, 0.8)
21	73.9, CH	5.53 d (1.6)	74.06, CH	5.51 br s
22	19.0, CH_3	1.21 d (6.7)	19.17, CH_3	1.18 d (6.6)
23	21.8, CH_3	1.54 s	21.86, CH_3	1.53 s
24	169.7, CO	-	169.82, CO	-
25	20.6, CH_3	2.16 s	20.68, CH_3	2.14 s
1'	136.9, C	-	137.11, C	-
2'	129.0, CH	7.17 d (7.0)	129.14, CH	7.15 m
3'	128.9, CH	7.33 t (7.5)	128.97, CH	7.32 m
4'	127.0, CH	7.25 t (7.5)	127.13, CH	7.24 m
5'	128.9, CH	7.33 t (7.5)	128.97, CH	7.32 m
6'	129.0, CH	7.17 d (7.0)	129.14, CH	7.15 m

Measured in chloroform-*d* at [a] 150 / [b] 600 MHz and [a] 100 / [b] 400 MHz.

Display Report

Analysis Info Acquisition Date 07.11.2023 11:45:20

Analysis Name	S:\DATA\AmaZon\dva23_Daniela Valencia Revelo\Gymnopus montagnei\4. Gymnopus Slurry\4. Semiprep\Semiprep 7\GymSlurry_SP7_R6F2_BB5_01_52135.d		
Method	52135.m	Operator	tti
Sample Name	GymSlurry_SP7_R6F2	Instrument	amaZon speed
Comment			

Acquisition Parameter

Ion Source Type	ESI	Ion Polarity	Positive	Alternating Ion Polarity	on
Mass Range Mode	UltraScan	Scan Begin	100 m/z	Scan End	2000 m/z
Accumulation Time	4000 µs	RF Level	100 %	Trap Drive	68.6
SPS Target Mass	1000 m/z	Averages	6 Spectra		

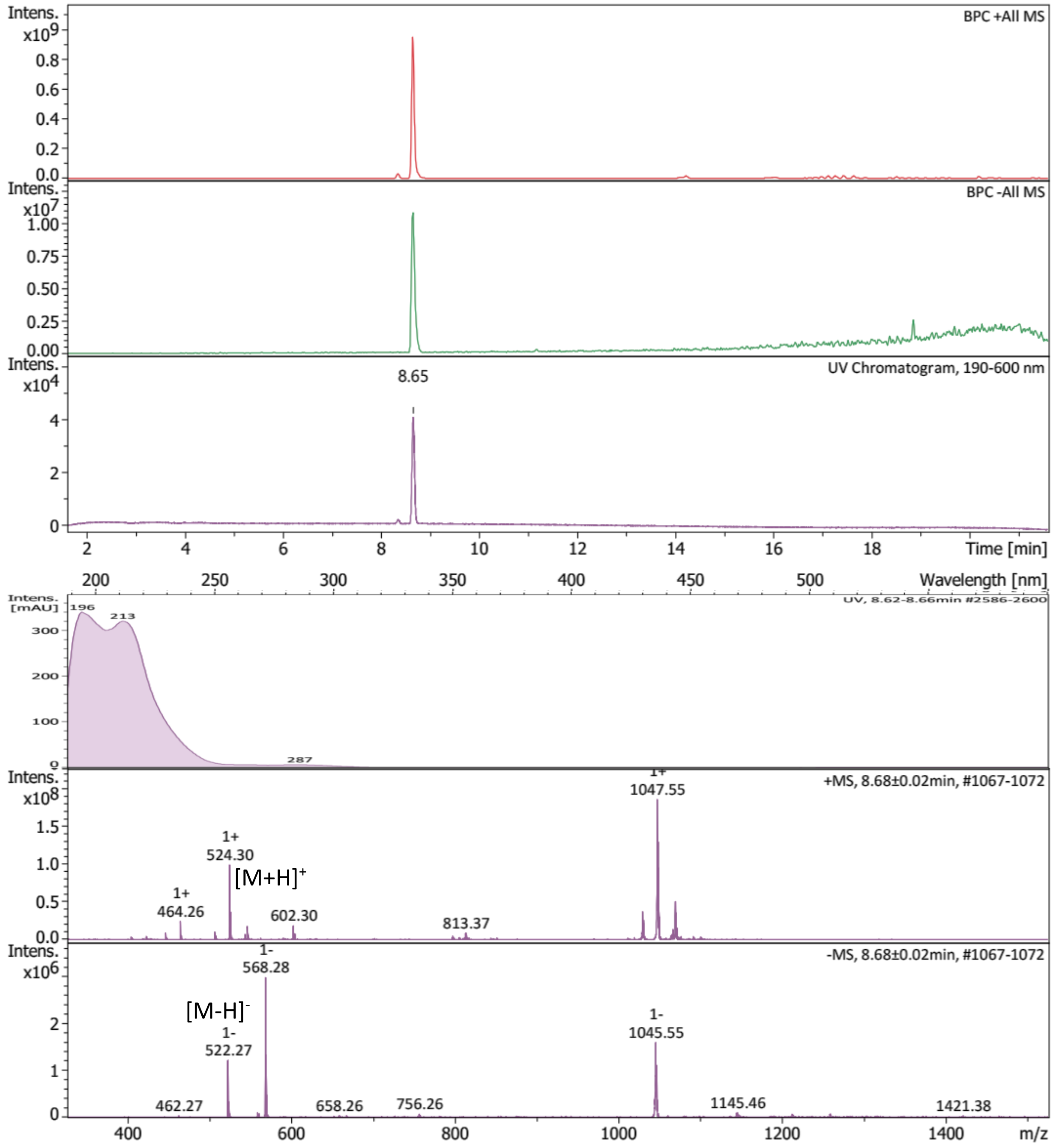

Bruker Compass DataAnalysis 6.1 printed: 20.11.2023 11:46:45 by: sel22 Page 1 of 1

Figure S75. LR-ESI-MS of **14**.

Display Report

Analysis Info

		Acquisition Date	26.09.2023 12:11:28
Analysis Name	S:\DATA\MaXis\dva23_Daniela Valencia Revelo\23_09_26\Gymnopus Slurry_R4_F9_19_01_13266.d		
Method	pos_säure_10000_screening_ms_100_2500_line.m	Operator	ate06
Sample Name	Gymnopus Slurry_R4_F9	Instrument	maXis
Comment	Screening01<br>Waters Acquity UPLC BEH C_18 1,7um 2.1x50mm		

Acquisition Parameter

Ion Polarity Positive

SPS Target Mass

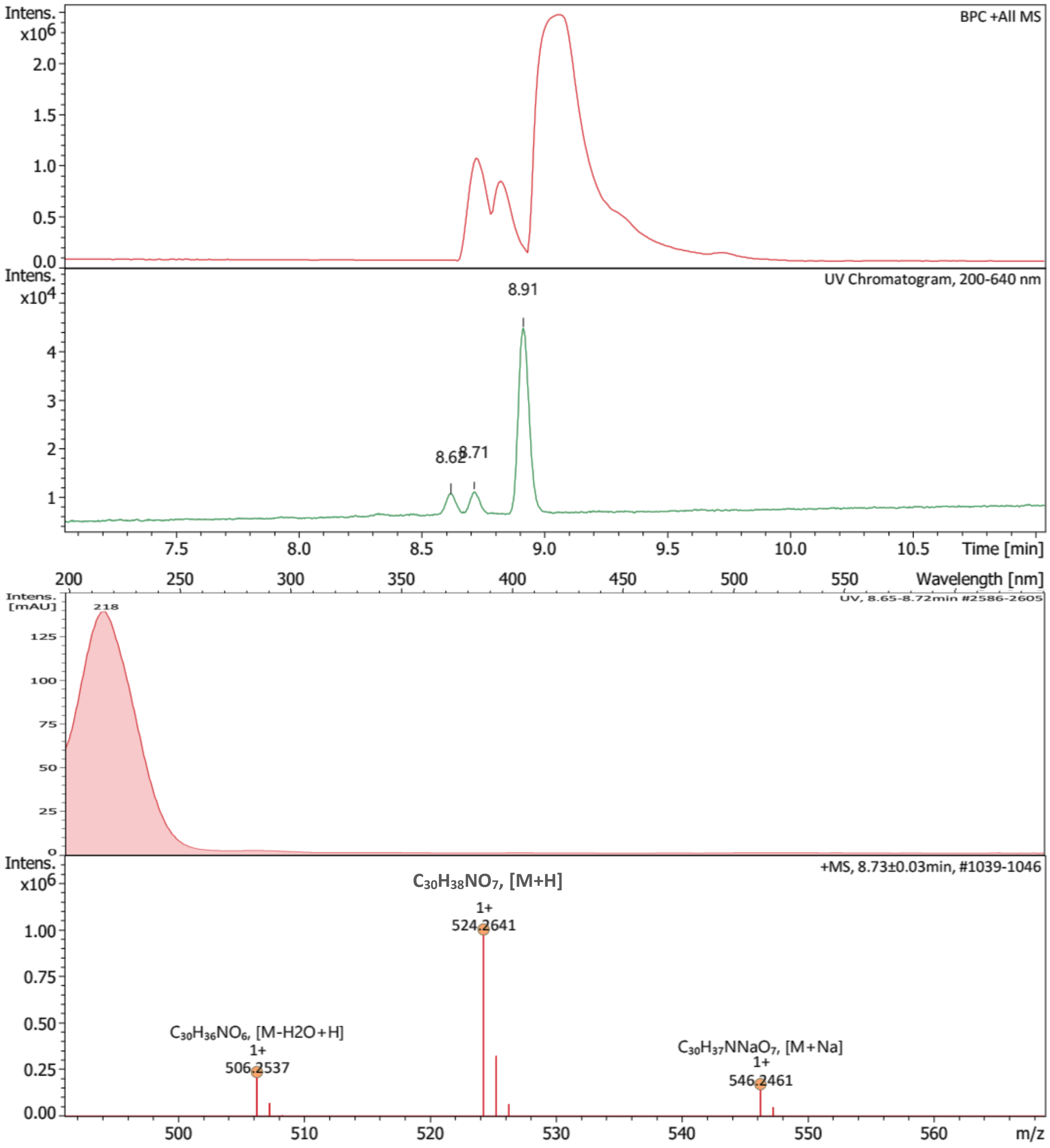

Bruker Compass DataAnalysis 6.1 printed: 23.11.2023 10:15:11 by: sel22 Page 1 of 1

Figure S76. HR-ESI-MS of **14**.

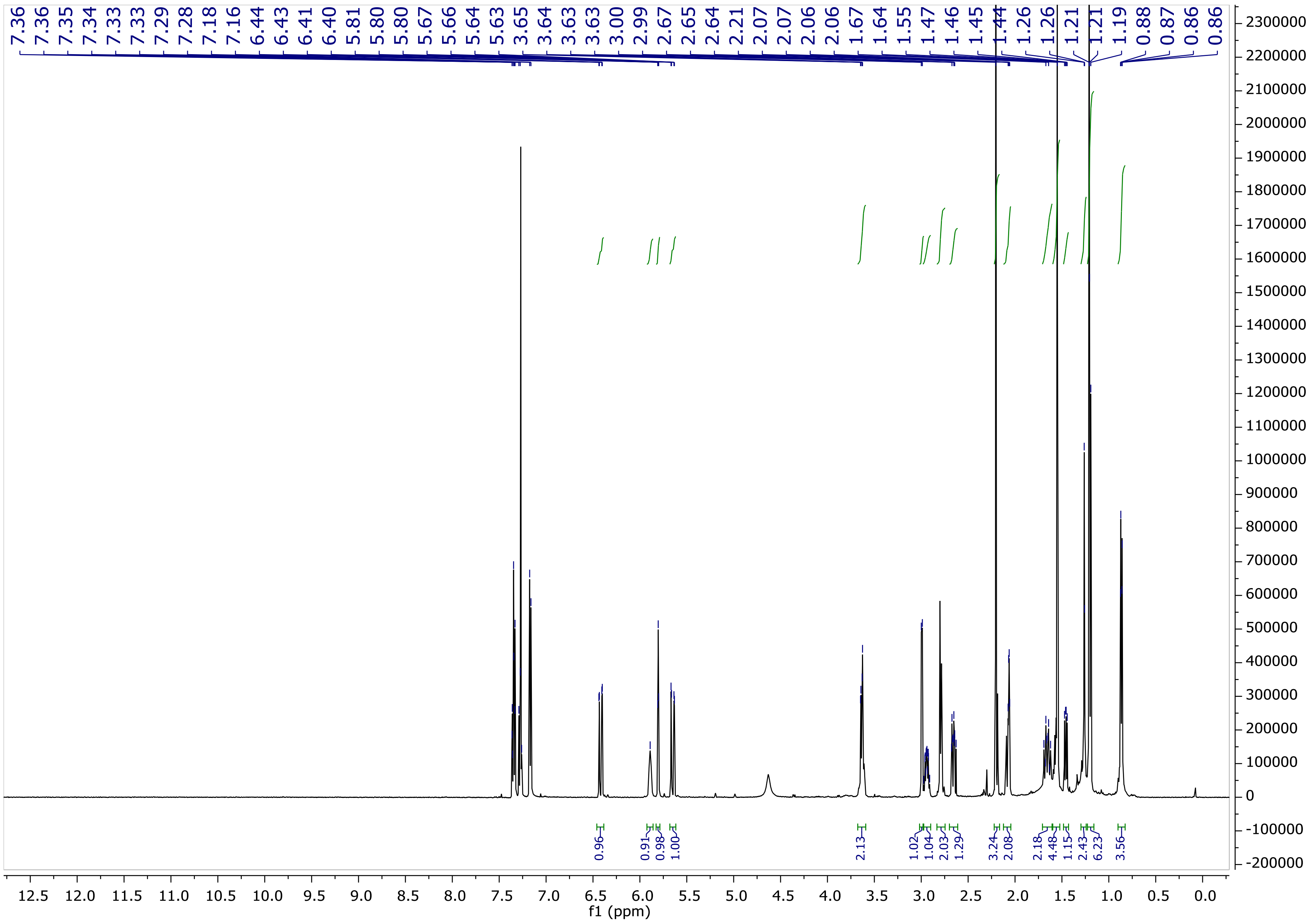

Figure S77. ^{1}H NMR spectrum of **14** in chloroform-*d* at 500 MHz.

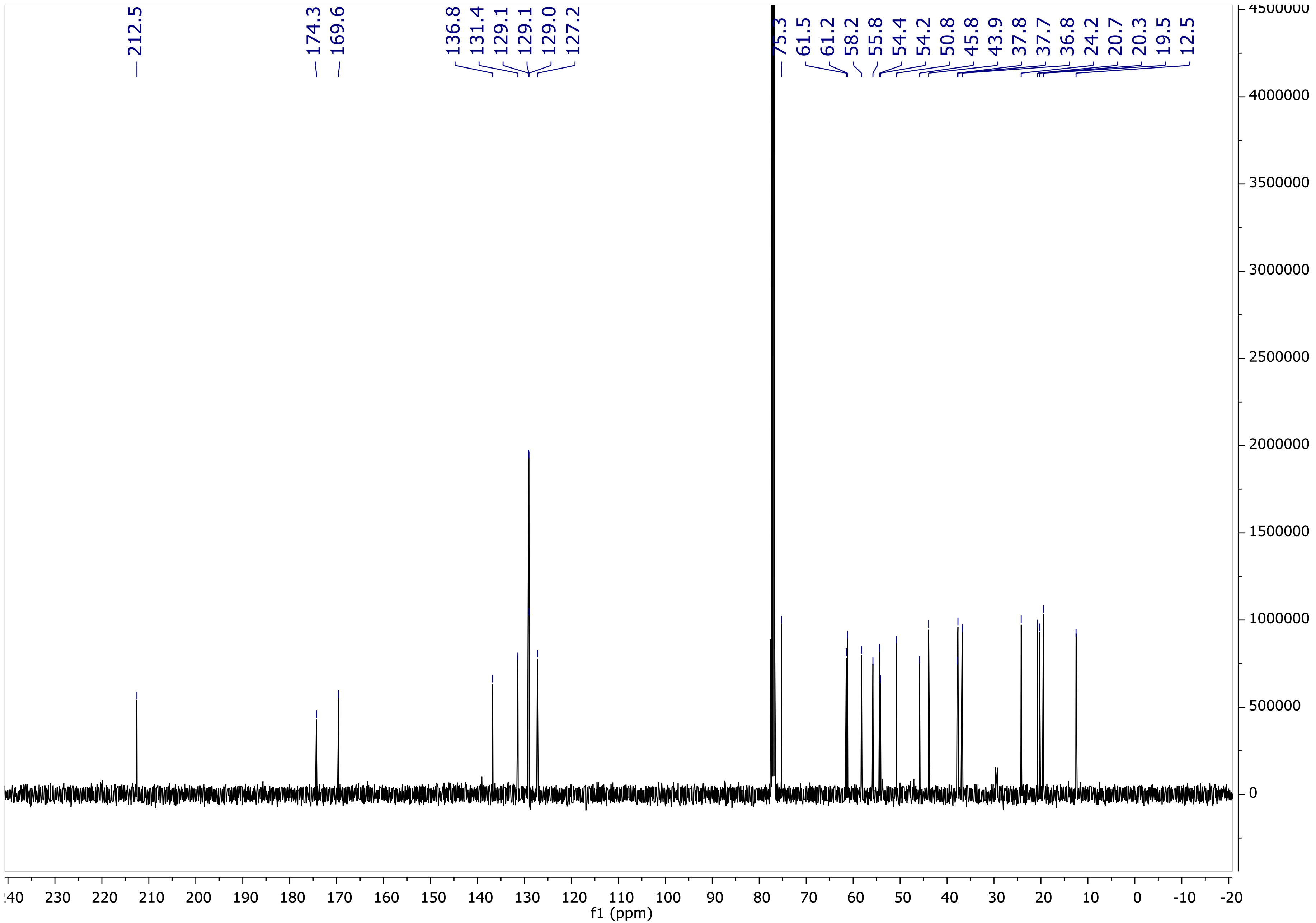

Figure S78. ^{13}C NMR spectrum of **14** in chloroform-*d* at 125 MHz.

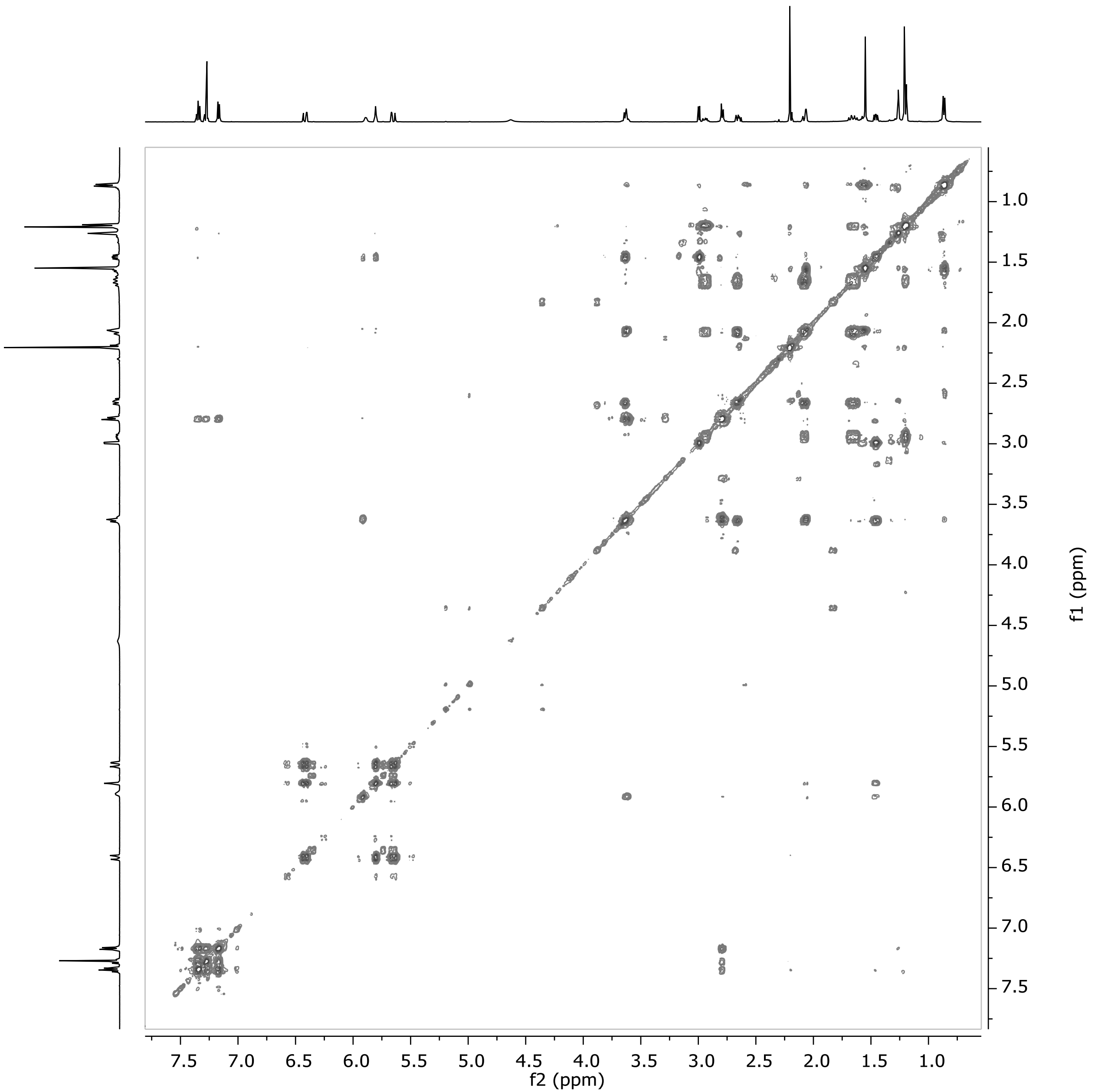

Figure S79. ^{1}H-^{1}H COSY spectrum of **14** in chloroform-*d* at 500 MHz.

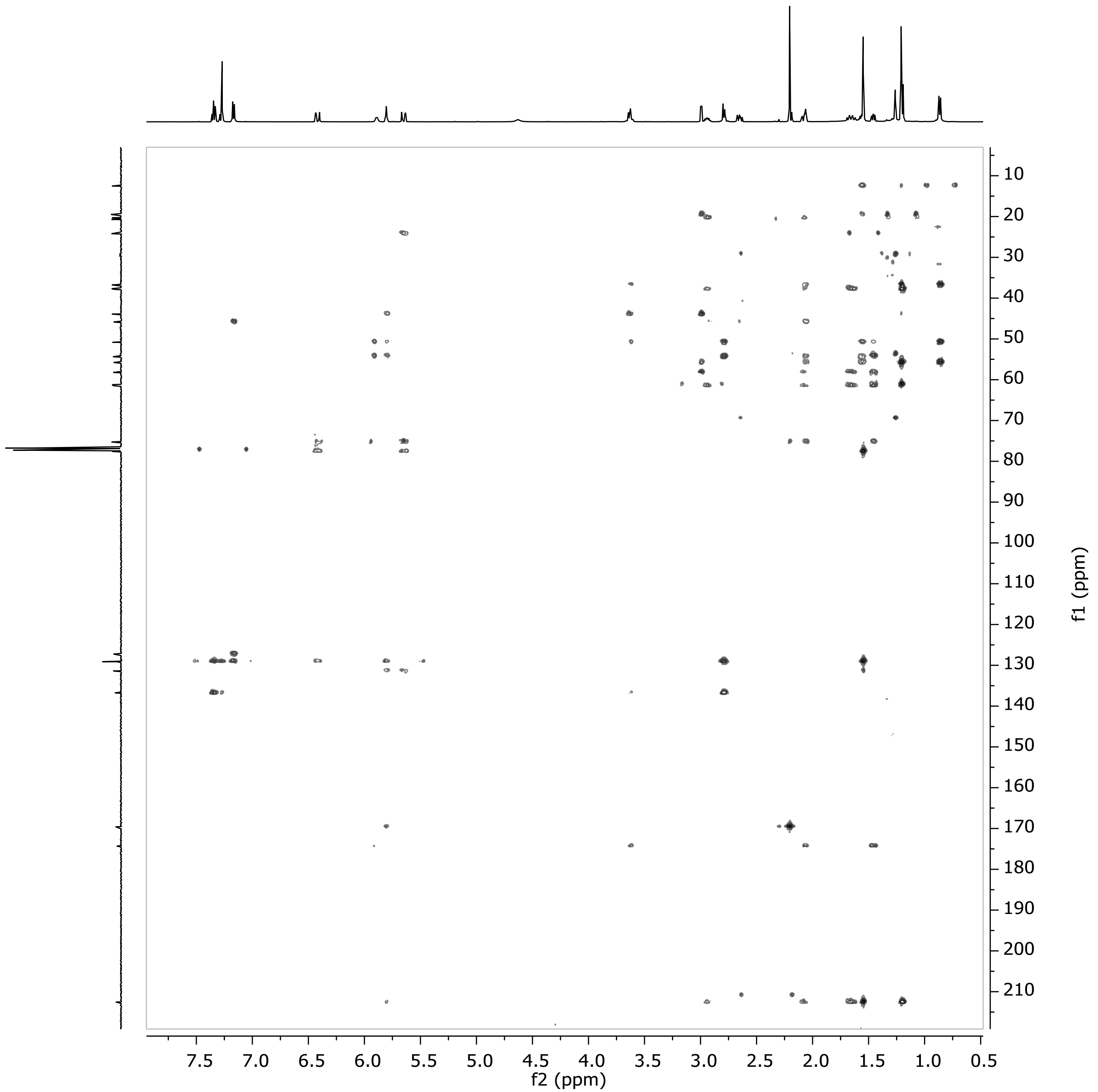

Figure S80. HMBC spectrum of **14** in chloroform-*d* at 500 MHz.

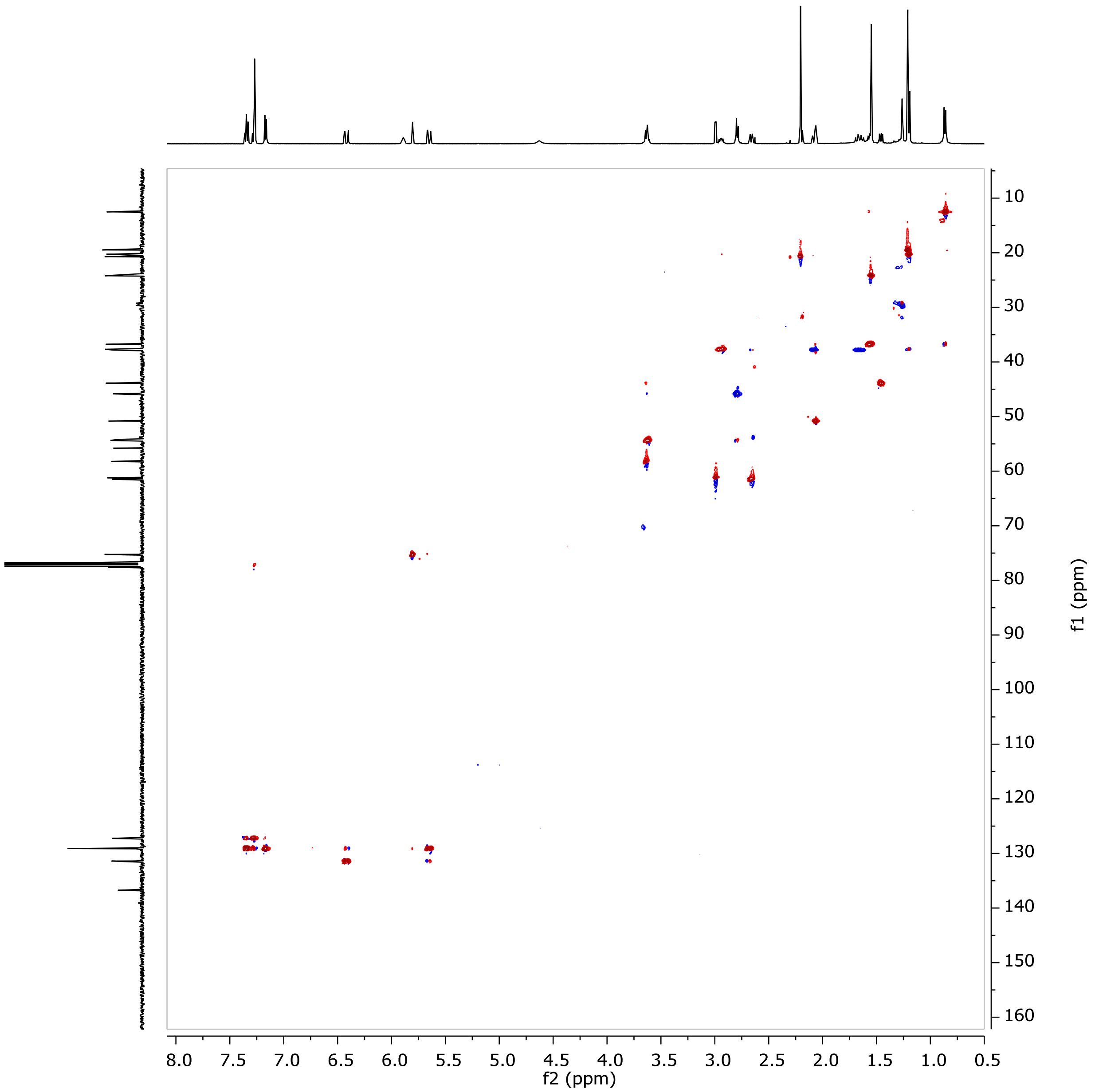

Figure S81. HSQC spectrum of **14** in chloroform-*d* at 500 MHz.

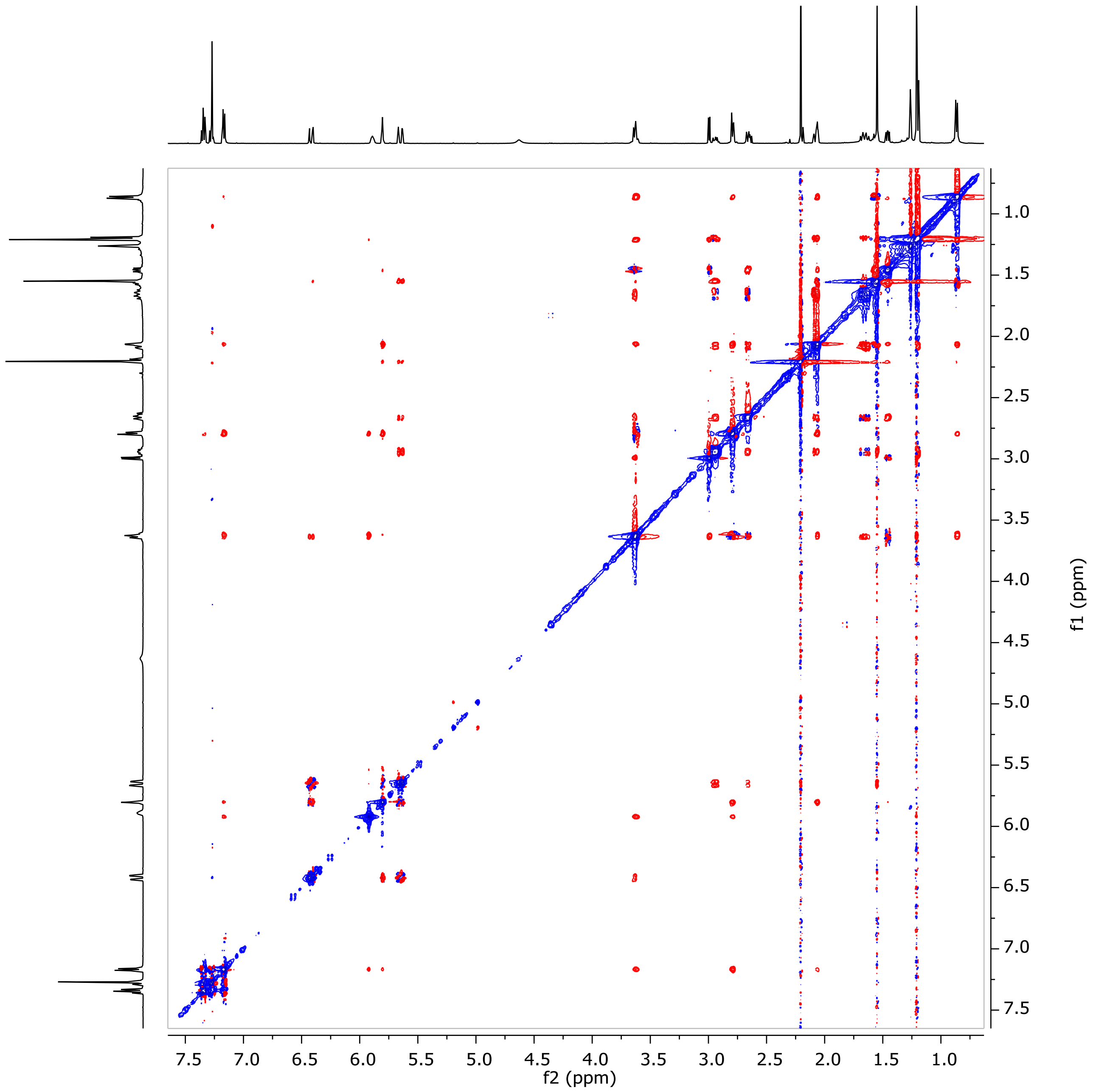

Figure S82. ROESY spectrum of **14** in chloroform-*d* at 500 MHz.

Table S19. ^{1}H and ^{13}C NMR data of compound **14** and cytochalasin R.

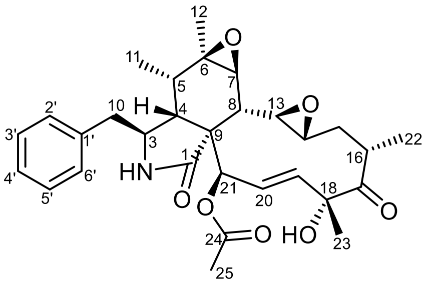

Cytochalasin R

	Compound **14**		Cytochalasin R	
pos.	δ_C,[a] type	δ_H[b] multi (*J* in Hz)	δ_C,[c] type	δ_H[d] multi (*J* in Hz)
1	174.5, CO	-	175.45, CO	-
2-N*H*	-	5.88 br s	-	9.70 br s
3	54.5, CH	3.62 m (overlapped)	54.75, CH	3.96 ddd (7.0, 7.1, 2.0)
4	51.0, CH	2.06 dd (5.6, 2.7)	50.79, CH	2.40 dd (5.7, 2.0)
5	36.9, CH	1.55 m (overlapped)	37.45, CH	1.85 dq (5.7, 7.3)
6	77.2, C	-	76.16, C	-
6-O*H*				
7	61.4, CH	2.98 d (5.7)	62.20, CH	3.39 d (5.7)
7-O*H*	-	-	-	-
8	44.1, CH	1.45 dd (8.7, 5.6)	44.78, CH	1.98 dd (8.6, 5.7)
9	54.3, C	-	55.20, C	-
10	46.0, CH_2	α 2.77 dd (13.4, 8.4)<br>β 2.80 dd (13.4, 6.7)	46.16, CH_2	α 2.84 dd (13.2, 7.7)<br>β 3.11 dd (13.2, 6.4)
11	12.7, CH_3	0.86 d (7.4, 1.7)	12.5, CH_3	0.71 d (7.3)
12	19.6, CH_3	1.20 s	19.55, CH_3	1.20 s
13	58.4, CH	3.63 dd (8.7, 2.1)	56.11, CH	4.37 dd (8.6, 2.0)
14	61.6, CH	2.65 dt (10.2, 2.4)	61.79, CH	3.00-3.12 m
15	38.0, CH_2	α 2.07 d (14.6)<br>β 1.65 dt (14.6, 10.7)	38.79, CH_2	α 1.95-2.09 m<br>β 2.12 dd (12.4, 1.5)
16	37.8, CH	2.93 dtd (10.9, 7.6, 6.2)	38.23, CH	3.00-3.12 m
17	212.7, CO	-	213.21, CO	-
18	77.6, C	-	78.76, C	-
18-O*H*	-	-	-	6.54 br s
19	129.2, CH	5.64 dd (15.6, 2.5)	130.56, CH	6.22 dd (12.5, 2.6)
20	131.5, CH	6.41 dd (15.6, 2.5)	132.22, CH	7.18 dd (12.5, 2.6)
21	75.4, CH	5.79 dd (2.5, 2.5)	77.07, CH	6.25 s
22	20.4, CH_3	1.19 d (7.0)	20.39, CH_3	1.03 d (6.8)
23	24.4, CH_3	1.54 s	20.48, CH_3	1.62 s
24	169.7, CO	-	170.61, CO	-
25	20.9, CH_3	2.20 s	24.77, CH_3	2.30 s
1'	136.9, C	-	138.15, C	-
2'	129.3, CH	7.16 d (7.0)	130.10, CH	7.20-7.38 m
3'	129.2, CH	7.34 t (7.4)	129.03, CH	7.20-7.38 m
4'	127.4, CH	7.27 t (7.4)	127.11, CH	7.20-7.38 m
5'	129.2, CH	7.34 t (7.4)	129.03, CH	7.20-7.38 m
6'	129.3, CH	7.16 d (7.0)	130.10, CH	7.20-7.38 m

Measured in chloroform-*d* at [a] 150 and [b] 600 MHz. Measured in pyridine-d_5 at [c] 67.8 and [d] 270 MHz.

Table S20. Antimicrobial activity (MIC) of compounds **1**–**14** against tested microorganisms.

	**MIC (µg/mL)**													
	**Compound**													
**Test Microorganism**	**1**	**2/3**	**4**	**5**	**6**	**7**	**8**	**9**	**10**	**11***	**12**	**13***	**14**	**Positive Control**
Staphylococcus aureus (DSM 346)	n.t.	n.i.	n.i.	n.i.	n.i.	n.i.	n.i.	n.i.	n.i.	n.i.	n.t.	n.i.	n.i.	0.42^{G}
Escherichia coli (DSM 1116)	n.i.	n.i.	n.i.	n.i.	n.i.	n.i.	n.i.	n.i.	n.i.	n.i.	n.t.	n.i.	n.i.	0.83^{G}
Bacillus subtilis (DSM 10)	n.i.	n.i.	n.i.	n.i.	n.i.	n.i.	n.i.	n.i.	n.i.	n.i.	n.t.	n.i.	n.i.	16.6^{O}
Pseudomonas aeruginosa (PA 14)	n.i.	n.i.	n.i.	n.i.	n.i.	n.i.	n.i.	n.i.	n.i.	n.i.	n.t.	n.i.	n.i.	0.42^{G}
Wickerhamomyces anomalus (DSM 6766)	n.t.	n.i.	n.i.	n.i.	n.i.	n.i.	n.i.	n.i.	n.i.	n.i.	n.t.	n.i.	n.i.	8.30^{N}
Candida albicans (DSM 1665)	n.t.	n.i.	n.i.	n.i.	n.i.	n.i.	n.i.	n.i.	n.i.	66.6	n.t.	n.i.	n.i.	8.30^{N}
Acinetobacter baumannii (DSM 30008)	n.t.	n.i.	n.i.	n.i.	n.i.	n.i.	n.i.	n.i.	n.i.	n.i.	n.t.	n.i.	n.i.	1.06^{C}
Chromobacterium violaceum (DSM 30191)	n.t.	n.i.	n.i.	n.i.	n.i.	n.i.	n.i.	n.i.	n.i.	n.i.	n.t.	n.i.	n.i.	1.67^{G}
Schizosaccharomyces pombe (DSM 70572)	n.t.	n.i.	n.i.	16.6	n.i.	n.i.	n.i.	n.i.	33.3	66.6	n.t.	n.i.	n.i.	8.30^{N}
Mucor hiemails (DSM 2656)	n.i.	n.i.	66.6	66.6	n.i.	n.i.	n.i.	n.i.	n.i.	n.i.	n.t.	n.i.	n.i.	8.30^{N}
Rhodotorula glutinis (DSM 10134)	n.t.	n.i.	n.i.	n.i.	66.6	n.i.	n.i.	n.i.	n.i.	n.i.	n.t.	n.i.	n.i.	4.20^{N}
Mycobacterium smegmatis (ATCC 700084)	n.i.	n.i.	n.i.	n.i.	n.i.	n.i.	n.i.	n.i.	n.i.	n.i.	n.t.	n.i.	n.i.	1.70^{K}

n.a.: No activity. n.i.: No inhibition up to 67 µg/mL. n.t.: Not tested. *: values reported by Lambert et al. [57]
G: Gentamicin; O: Oxytetracycline; N: Nystatin; C: Ciprofloxacin; K: Kanymycin.

Table S21. *Tub2* and *rpb2* sequences of *Xylaria* sp. CM-UDEA-H199.

>Xylaria_rpb2_CM-UDEA-H199

TCTTCCGCAATATTGCGCGTCGGATGACCCAGGAGGTTCTATCCCATCTCAAACGGAGTAT
CGAGCAAGGCAAGCAGTTCAATATTGCCCTCGCCGTTAAGTCAAATATTATTACGAGCGGG
TTGAAATATTCTCTCGCTACAGGCAATTGGGGTGATCAGAAGAAGGCCATGAGCTCTACTG
CTGGTGTCTCGCAGGTCTTGAATAGATACACATTCGCATCTACTTTATCACATTTGCGAAGA
ACAAATACTCCGGTTGGTAGAGATGGTAAGCTTGCCAAGCCACGGCAGCTTCACAACACCC
ACTGGGGTCTGGTCTGTCCGGCCGAGACGCCTGAAGGTCAGGCTTGTGGCTTAGTCAAGAA
CCTTTCCCTTATGTGCTCCATCAGCGTGGGTACCTCGACGGAACCTATTATAGAATATATGA
TTTCGCGAAATATGGAGGTTCTGGAAGAGTATGAACCTCAAAGGTACCCACATGCTACGAA
GATCTTTCTCAATGGATCGTGGATTGGTATCCACCAAGATCCAAAAGCCCTCGTCAGAGAT
GTTCAACAATTACGCCGGACAAACCAGATTCCAGCTGAAGTATCCTTGATTCGAGATATAC
GTGATCGCGAATTCAAGATCTTCTCAGATGCCGGCCGTGTTATGCGGCCCTTGTTTGTGGTC
CACCAAGAAGATGATCCTGATAACAATATCGAAAAGGGCTCATTAGTCTTGACAAAAGAC
ATGGTCCGGCGGCTTGAAATTGATCAGACCCTTCCACCTGGAAGCGACGAATATTTCGGAT
GGCAGGGCCTGGTTAATGCCGGTGTCATCGAATATATGGACGCTGAGGAAGAGGAAACTG
CCATGATTTGCATGACTCCCGAAGATCTAGAGGCTTTCAGACTGACCAAGTTGGGCCTGCC
GGATCCCGACGCGGAATCCAGCTTGAACGCGCCCAATAAACGATTAAAGACGAGAATGAA
TCCGACAACACATACGTACACCCACTGTGAAATTCACCCCAGTATGCTTCTTGGTATTTGTG
CCAGCATCATCCCCTTCCCTGATCATAACCAGGTAAGTGTCACCACGGTCCATTACGTCGC
AGTCACTGACACTACAG

>Xylaria_tub2_CM-UDEA-H199

ATCGCGCTAACCATGCTTCCCCATTCTAGGTTCACCTCCAAACCGGCCAATGCGTAGGTTGC
TCTCCAGCTCTCACAGCGGCATCGAGAAGCTCCCGCGACTCACATGGGATAATAGGGTAAC
CAAATTGGTGCTGCTTTCTGGCAACAAATTTCTGGCGAGCACGGTCTCGACGGCAATGGAG
TGTATGTCTTTGGATCTGGAACGTGGCAACCGCATTGGTGGACTGACAGGGATGAAACAGC
TACAACGGAACTTCCGAGCTCCAGCTAGAGCGCATGAGCGTTTACTTCAACGAGGTAGATA
CCGCCAAATATCCTCTGTTGCATATACGAACCTTCTAACACGATGGGGATTTTAGGGTGCC
AACAACAAGTATGTCCCTCGCGCCGTGCTCGTCGACTTGGAACCCGGTACCATGGACGCTG
TCCGTGCCGGTCCTTTCGGCCAGCTTTTCCGTCCCGACAACTTCGTCTTCGGTCAGTCCGGT
GCCGGCAACAACTGGGCCAAGGGCCACTACACCGAGGGTGCTGAGCTGGTTGACAACGTC
CTCGATGTCGTCCGTCGCGAGGCTGAGGGCTGTGACTGCCTTCAGGGTTTCCAGATTACCC
ACTCGCTTGGTGGTGGTACTGGTGCCGGTATGGGTACGCTGCTGATCTCCAAAATCCGCGA
GGAATTCCCTGACCGCATGATGGCTACCTTCTCCGTCATGCCCTCTCCCAAGGTCTCCGACA
CCGTCGTCGAGCCCTACAACGCCACTCTCTCCGTCCATCAGCTGGTCGAGAACTCGGACGA
GACCTTCTGTATCGATAACGAGGCTCTGTACGACATCTGCATGCGTACCCTGAAGCTATCC
AACCCGTCATACGGTGACTTGAACCACCTTGTCTCCGCCGTCATGTCTGGTGTTACTACCTG
CTTGCGTTTCCCTGGTCAGCTTAACTCTGATCTGCGCAAGTTGGCTGTCAACATGGTGCCAT
TCCCTCGTCTGCACTTCTTCATGGTCGGCTTCGCGCCTCTCACTAGTCGTGGTGCTCACTCTT
TCCGTGCTGTCACCGTTCCCGAGCTGACCCAGCAAATGTTTGACCCCAAGAACATGATGGC
TGCTGCTGATTTCCGCAACGGTCGTTACCTGACATGCTCTGCAATCTTGTAAGAAAACACCT
CCCCTATAGTGCAACCCATAGCTAACCGCACCCCCCACCAGCCGTGGCAAGGTTTCCATGA
AGGAGGT

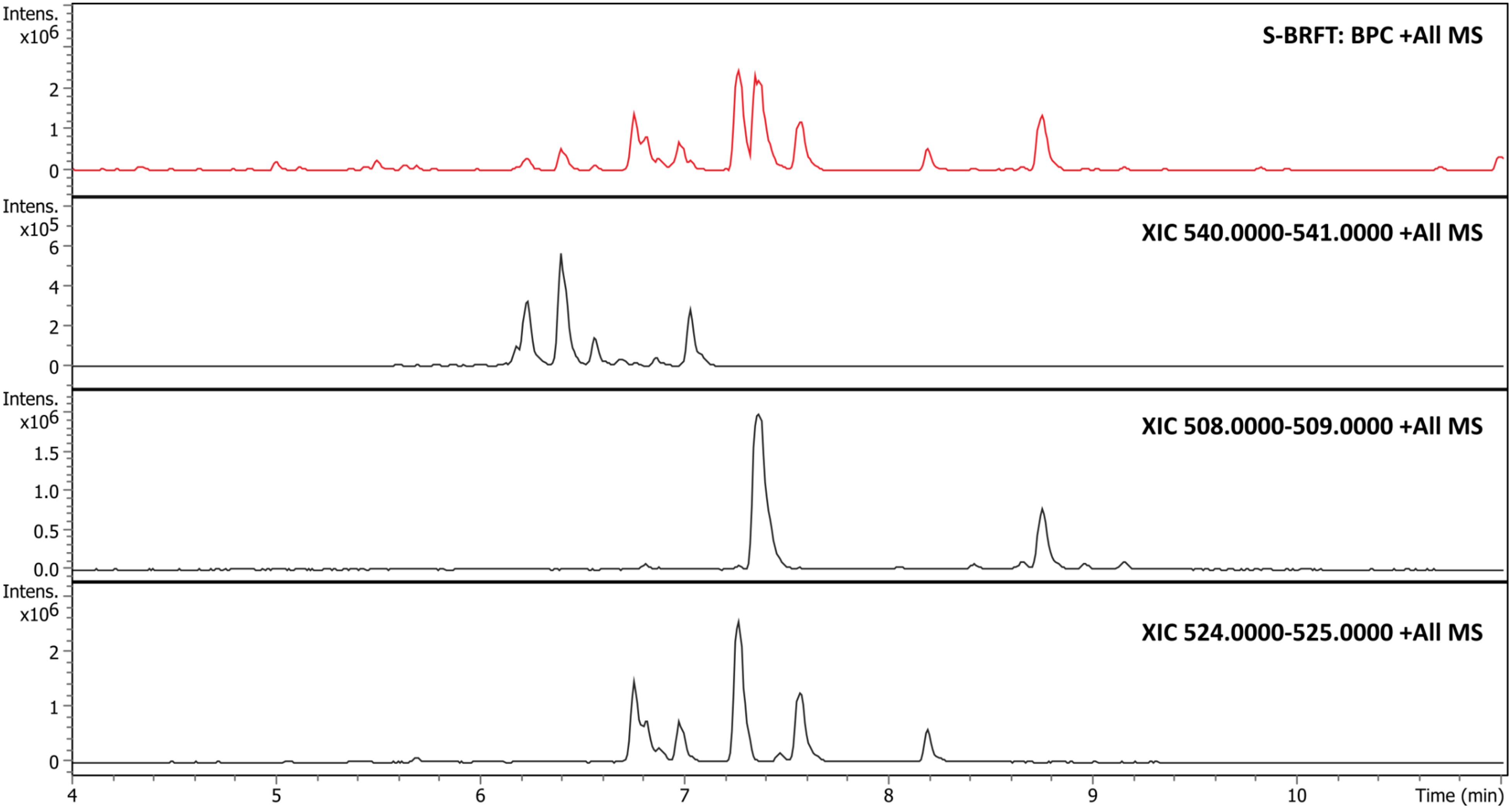

Figure S83. Base peak chromatogram (BPC) from the crude extract obtained after cultivation in S-BRFT and extracted ion chromatograms (XIC) of isobaric cytochalasins.

METABOLOMICS PROCEDURES:

The preprocessing parameters used within MetaboScape are shown below:

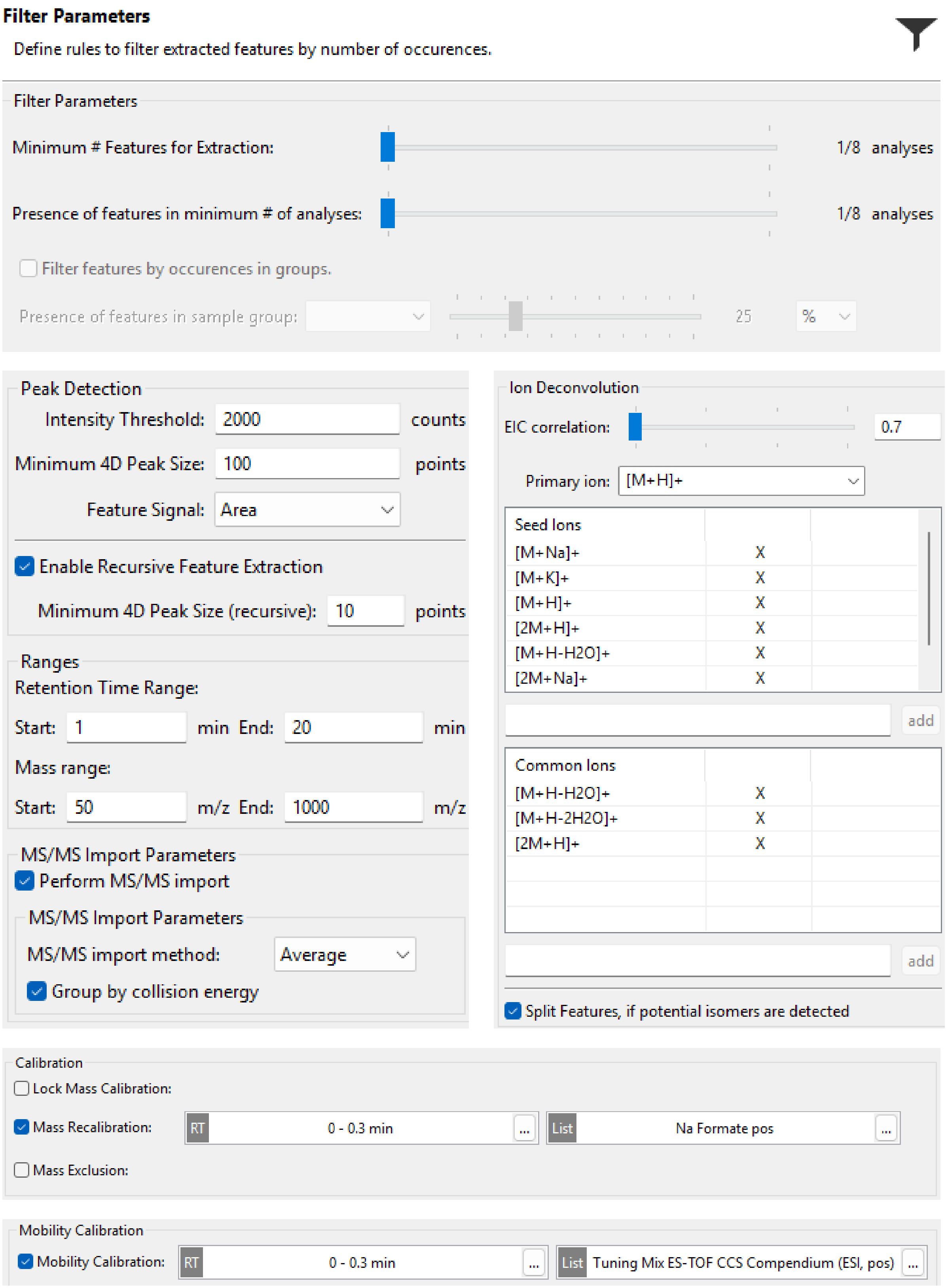

It is noting that CANOPUS is a paramater-free tool, which can be used within SIRIUS:

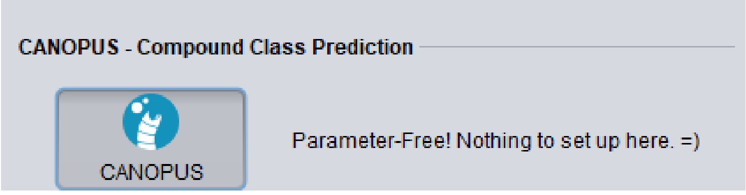

The parameters used for the FBMN within the GNPS2 infrastructure can be accessed by looking into the publicly available job (Task ID: 8061de20d4034ad8b3ca5f405eb15486).

The R script used for the heatmap generation and PCA analysis is shown below:

```
#Read data
features_names <- Dataset$FEATURE_ID
abundance_values <-Dataset[, -1]  # Exclude the first column (compound names)


# Create heatmap
pheatmap(abundance_values,
         labels_row = features_names,  # Use compound_names as row labels
         #color = colorRampPalette(c("blue", "white", "red"))(100),  Choose your color palette
         #main = "Abundance Heatmap",
         color = colorRampPalette(c("skyblue4", "white", "coral2"))(100),  # Choose your color palette
         cluster_rows = TRUE,  # Cluster rows
         cluster_cols = TRUE,  # Cluster columns
         scale = "row",  # Scale rows, if needed)
         clustering_distance_cols = "canberra",show_rownames = FALSE,cutree_cols = 7)
#treeheight_row =0)
```

```
# Create PCA
library(ggplot2)
scaled_data <- scale(abundance_values)
pca_result <- prcomp(scaled_data, center = TRUE, scale. = TRUE)
pca_data <- as.data.frame(pca_result$x)
pca_data$sample <- rownames(pca_data)
sample_labels <- colnames(abundance_values)
pca_data$group <- sample_labels

ggplot(pca_data, aes(x = PC1, y = PC2, color = group, label = sample)) +
  geom_point(size = 2) +
  geom_text(aes(label = sample), vjust = -1, hjust = 1) +
  xlab(paste0("PC1 (", round(summary(pca_result)$importance[2, 1] * 100, 1), "%)")) +
  ylab(paste0("PC2 (", round(summary(pca_result)$importance[2, 2] * 100, 1), "%)")) +
  ggtitle("PCA of Abundance Values") +
  theme_minimal()
```
